# Supplementary material for: Pyruvate Dehydrogenase Kinase 1 inhibition mediated oxidative phosphorylation enhancement in cartilage promotes osteoarthritis progression
Source: BMC Musculoskelet Disord. 2023 Jul 20;24:597. doi: 10.1186/s12891-023-06585-6 (PMC10357736; doi:10.1186/s12891-023-06585-6)
Supplement: Supplementary file 1 — Additional file 1. [file 12891_2023_6585_MOESM1_ESM.zip › BP.Gsea.1653623667859/gsea_report_for_normal_1653623667859.html]

Report for normal 1653623667859 [GSEA]

| GS  follow link to MSigDB | GS DETAILS | SIZE | ES | NES | NOM p-val | FDR q-val | FWER p-val | RANK AT MAX | LEADING EDGE || 1 | GOBP\_CYTOPLASMIC\_TRANSLATION | Details ... | 129 | -0.55 | -2.44 | 0.000 | 0.000 | 0.000 | 2701 | tags=53%, list=21%, signal=66% |
| 2 | GOBP\_REGULATION\_OF\_RNA\_SPLICING | Details ... | 131 | -0.51 | -2.29 | 0.000 | 0.001 | 0.002 | 2643 | tags=47%, list=20%, signal=58% |
| 3 | GOBP\_RIBOSOMAL\_LARGE\_SUBUNIT\_ASSEMBLY | Details ... | 24 | -0.71 | -2.25 | 0.000 | 0.001 | 0.002 | 2701 | tags=67%, list=21%, signal=84% |
| 4 | GOBP\_REGULATION\_OF\_MRNA\_PROCESSING | Details ... | 116 | -0.50 | -2.24 | 0.000 | 0.001 | 0.003 | 2403 | tags=44%, list=19%, signal=54% |
| 5 | GOBP\_REGULATION\_OF\_MIRNA\_TRANSCRIPTION | Details ... | 58 | -0.56 | -2.19 | 0.000 | 0.003 | 0.011 | 3107 | tags=53%, list=24%, signal=70% |
| 6 | GOBP\_REGULATION\_OF\_MRNA\_SPLICING\_VIA\_SPLICEOSOME | Details ... | 86 | -0.51 | -2.17 | 0.000 | 0.004 | 0.018 | 2403 | tags=45%, list=19%, signal=55% |
| 7 | GOBP\_GLOMERULAR\_MESANGIUM\_DEVELOPMENT | Details ... | 16 | -0.73 | -2.14 | 0.000 | 0.006 | 0.032 | 1287 | tags=50%, list=10%, signal=55% |
| 8 | GOBP\_CELL\_FATE\_DETERMINATION | Details ... | 37 | -0.57 | -2.09 | 0.000 | 0.012 | 0.073 | 2196 | tags=46%, list=17%, signal=55% |
| 9 | GOBP\_REGULATION\_OF\_ASTROCYTE\_DIFFERENTIATION | Details ... | 25 | -0.64 | -2.07 | 0.002 | 0.017 | 0.115 | 1983 | tags=60%, list=15%, signal=71% |
| 10 | GOBP\_POSITIVE\_REGULATION\_OF\_RNA\_SPLICING | Details ... | 29 | -0.59 | -2.03 | 0.000 | 0.025 | 0.191 | 4043 | tags=69%, list=31%, signal=100% |
| 11 | GOBP\_RIBOSOMAL\_LARGE\_SUBUNIT\_BIOGENESIS | Details ... | 61 | -0.51 | -2.01 | 0.000 | 0.028 | 0.227 | 3127 | tags=54%, list=24%, signal=71% |
| 12 | GOBP\_POSITIVE\_REGULATION\_OF\_MIRNA\_TRANSCRIPTION | Details ... | 43 | -0.54 | -2.01 | 0.000 | 0.027 | 0.231 | 3107 | tags=51%, list=24%, signal=67% |
| 13 | GOBP\_REGULATION\_OF\_MRNA\_METABOLIC\_PROCESS | Details ... | 234 | -0.41 | -1.99 | 0.000 | 0.035 | 0.311 | 2525 | tags=36%, list=20%, signal=44% |
| 14 | GOBP\_POSITIVE\_REGULATION\_OF\_SIGNAL\_TRANSDUCTION\_BY\_P53\_CLASS\_MEDIATOR | Details ... | 24 | -0.61 | -1.98 | 0.000 | 0.036 | 0.338 | 2294 | tags=50%, list=18%, signal=61% |
| 15 | GOBP\_MRNA\_TRANSPORT | Details ... | 108 | -0.45 | -1.97 | 0.000 | 0.038 | 0.378 | 2712 | tags=41%, list=21%, signal=51% |
| 16 | GOBP\_SPROUTING\_ANGIOGENESIS | Details ... | 109 | -0.45 | -1.97 | 0.000 | 0.039 | 0.400 | 2997 | tags=40%, list=23%, signal=52% |
| 17 | GOBP\_NEGATIVE\_REGULATION\_OF\_MRNA\_METABOLIC\_PROCESS | Details ... | 78 | -0.48 | -1.97 | 0.000 | 0.039 | 0.411 | 2490 | tags=38%, list=19%, signal=47% |
| 18 | GOBP\_RNA\_STABILIZATION | Details ... | 47 | -0.52 | -1.97 | 0.000 | 0.037 | 0.415 | 3279 | tags=49%, list=25%, signal=65% |
| 19 | GOBP\_POSITIVE\_REGULATION\_OF\_TRANSCRIPTION\_FROM\_RNA\_POLYMERASE\_II\_PROMOTER\_IN\_RESPONSE\_TO\_STRESS | Details ... | 20 | -0.64 | -1.95 | 0.002 | 0.041 | 0.466 | 2082 | tags=55%, list=16%, signal=65% |
| 20 | GOBP\_NEGATIVE\_REGULATION\_OF\_MRNA\_CATABOLIC\_PROCESS | Details ... | 53 | -0.51 | -1.95 | 0.000 | 0.040 | 0.476 | 3279 | tags=47%, list=25%, signal=63% |
| 21 | GOBP\_RIBOSOME\_ASSEMBLY | Details ... | 53 | -0.51 | -1.94 | 0.000 | 0.042 | 0.512 | 2701 | tags=49%, list=21%, signal=62% |
| 22 | GOBP\_BRANCHING\_INVOLVED\_IN\_BLOOD\_VESSEL\_MORPHOGENESIS | Details ... | 29 | -0.58 | -1.94 | 0.002 | 0.044 | 0.538 | 2403 | tags=45%, list=19%, signal=55% |
| 23 | GOBP\_RNA\_EXPORT\_FROM\_NUCLEUS | Details ... | 69 | -0.47 | -1.92 | 0.000 | 0.050 | 0.611 | 2275 | tags=36%, list=18%, signal=44% |
| 24 | GOBP\_NEGATIVE\_REGULATION\_OF\_RNA\_CATABOLIC\_PROCESS | Details ... | 59 | -0.48 | -1.91 | 0.000 | 0.058 | 0.686 | 3279 | tags=44%, list=25%, signal=59% |
| 25 | GOBP\_MRNA\_SPLICE\_SITE\_SELECTION | Details ... | 25 | -0.58 | -1.91 | 0.002 | 0.056 | 0.689 | 2905 | tags=68%, list=23%, signal=88% |
| 26 | GOBP\_ENDODERM\_FORMATION | Details ... | 46 | -0.50 | -1.90 | 0.000 | 0.058 | 0.714 | 2059 | tags=39%, list=16%, signal=46% |
| 27 | GOBP\_REGULATION\_OF\_SIGNAL\_TRANSDUCTION\_BY\_P53\_CLASS\_MEDIATOR | Details ... | 88 | -0.45 | -1.90 | 0.000 | 0.057 | 0.718 | 1857 | tags=34%, list=14%, signal=40% |
| 28 | GOBP\_NEURONAL\_STEM\_CELL\_POPULATION\_MAINTENANCE | Details ... | 17 | -0.65 | -1.90 | 0.000 | 0.057 | 0.733 | 2051 | tags=47%, list=16%, signal=56% |
| 29 | GOBP\_NUCLEOLUS\_ORGANIZATION | Details ... | 15 | -0.67 | -1.90 | 0.000 | 0.056 | 0.735 | 1960 | tags=47%, list=15%, signal=55% |
| 30 | GOBP\_POSITIVE\_REGULATION\_BY\_HOST\_OF\_VIRAL\_TRANSCRIPTION | Details ... | 17 | -0.65 | -1.89 | 0.000 | 0.054 | 0.736 | 2893 | tags=59%, list=22%, signal=76% |
| 31 | GOBP\_RIBOSOME\_BIOGENESIS | Details ... | 243 | -0.38 | -1.89 | 0.000 | 0.056 | 0.759 | 2952 | tags=40%, list=23%, signal=51% |
| 32 | GOBP\_REGULATION\_OF\_SPROUTING\_ANGIOGENESIS | Details ... | 33 | -0.54 | -1.88 | 0.000 | 0.060 | 0.795 | 2456 | tags=42%, list=19%, signal=52% |
| 33 | GOBP\_MAINTENANCE\_OF\_BLOOD\_BRAIN\_BARRIER | Details ... | 29 | -0.54 | -1.87 | 0.002 | 0.064 | 0.824 | 3577 | tags=59%, list=28%, signal=81% |
| 34 | GOBP\_NUCLEAR\_EXPORT | Details ... | 128 | -0.41 | -1.87 | 0.000 | 0.064 | 0.832 | 2712 | tags=37%, list=21%, signal=46% |
| 35 | GOBP\_INOSITOL\_PHOSPHATE\_CATABOLIC\_PROCESS | Details ... | 18 | -0.63 | -1.87 | 0.002 | 0.063 | 0.834 | 1325 | tags=33%, list=10%, signal=37% |
| 36 | GOBP\_RIBOSOMAL\_SMALL\_SUBUNIT\_BIOGENESIS | Details ... | 58 | -0.47 | -1.86 | 0.000 | 0.064 | 0.847 | 2453 | tags=43%, list=19%, signal=53% |
| 37 | GOBP\_ENDOTHELIUM\_DEVELOPMENT | Details ... | 111 | -0.42 | -1.86 | 0.000 | 0.063 | 0.849 | 4301 | tags=53%, list=33%, signal=79% |
| 38 | GOBP\_EMBRYONIC\_PLACENTA\_DEVELOPMENT | Details ... | 78 | -0.44 | -1.86 | 0.000 | 0.066 | 0.868 | 2979 | tags=38%, list=23%, signal=50% |
| 39 | GOBP\_POSITIVE\_REGULATION\_OF\_MRNA\_SPLICING\_VIA\_SPLICEOSOME | Details ... | 17 | -0.63 | -1.86 | 0.000 | 0.064 | 0.868 | 4043 | tags=76%, list=31%, signal=111% |
| 40 | GOBP\_REGULATION\_OF\_EPITHELIAL\_CELL\_APOPTOTIC\_PROCESS | Details ... | 77 | -0.44 | -1.86 | 0.000 | 0.065 | 0.883 | 3162 | tags=42%, list=25%, signal=55% |
| 41 | GOBP\_REGULATION\_OF\_DNA\_TEMPLATED\_TRANSCRIPTION\_IN\_RESPONSE\_TO\_STRESS | Details ... | 46 | -0.49 | -1.85 | 0.000 | 0.070 | 0.907 | 1713 | tags=33%, list=13%, signal=37% |
| 42 | GOBP\_TOLERANCE\_INDUCTION | Details ... | 28 | -0.55 | -1.84 | 0.002 | 0.075 | 0.930 | 2582 | tags=43%, list=20%, signal=53% |
| 43 | GOBP\_REGULATION\_OF\_CELLULAR\_RESPONSE\_TO\_HEAT | Details ... | 17 | -0.63 | -1.84 | 0.002 | 0.075 | 0.934 | 3955 | tags=76%, list=31%, signal=110% |
| 44 | GOBP\_RENAL\_SYSTEM\_VASCULATURE\_DEVELOPMENT | Details ... | 28 | -0.54 | -1.83 | 0.004 | 0.081 | 0.945 | 2598 | tags=43%, list=20%, signal=54% |
| 45 | GOBP\_DEVELOPMENT\_OF\_PRIMARY\_FEMALE\_SEXUAL\_CHARACTERISTICS | Details ... | 95 | -0.42 | -1.82 | 0.002 | 0.083 | 0.950 | 2959 | tags=32%, list=23%, signal=41% |
| 46 | GOBP\_RIBONUCLEOPROTEIN\_COMPLEX\_BIOGENESIS | Details ... | 351 | -0.36 | -1.82 | 0.000 | 0.086 | 0.959 | 2952 | tags=38%, list=23%, signal=49% |
| 47 | GOBP\_RRNA\_METABOLIC\_PROCESS | Details ... | 212 | -0.38 | -1.81 | 0.000 | 0.089 | 0.966 | 3131 | tags=41%, list=24%, signal=53% |
| 48 | GOBP\_HISTONE\_DEACETYLATION | Details ... | 75 | -0.44 | -1.81 | 0.000 | 0.088 | 0.967 | 917 | tags=21%, list=7%, signal=23% |
| 49 | GOBP\_REGULATION\_OF\_NUCLEAR\_TRANSCRIBED\_MRNA\_CATABOLIC\_PROCESS\_DEADENYLATION\_DEPENDENT\_DECAY | Details ... | 18 | -0.61 | -1.80 | 0.003 | 0.097 | 0.972 | 3279 | tags=67%, list=25%, signal=89% |
| 50 | GOBP\_SKELETAL\_MUSCLE\_CELL\_DIFFERENTIATION | Details ... | 44 | -0.49 | -1.79 | 0.000 | 0.109 | 0.983 | 1880 | tags=34%, list=15%, signal=40% |
| 51 | GOBP\_NEGATIVE\_REGULATION\_OF\_MIRNA\_TRANSCRIPTION | Details ... | 19 | -0.59 | -1.79 | 0.005 | 0.108 | 0.987 | 2004 | tags=47%, list=16%, signal=56% |
| 52 | GOBP\_ENDODERM\_DEVELOPMENT | Details ... | 68 | -0.44 | -1.79 | 0.003 | 0.108 | 0.987 | 2059 | tags=32%, list=16%, signal=38% |
| 53 | GOBP\_RESPONSE\_TO\_GONADOTROPIN | Details ... | 27 | -0.53 | -1.78 | 0.000 | 0.115 | 0.993 | 2753 | tags=37%, list=21%, signal=47% |
| 54 | GOBP\_EPITHELIAL\_CELL\_APOPTOTIC\_PROCESS | Details ... | 99 | -0.41 | -1.78 | 0.000 | 0.114 | 0.993 | 3162 | tags=39%, list=25%, signal=52% |
| 55 | GOBP\_FEMALE\_SEX\_DIFFERENTIATION | Details ... | 109 | -0.40 | -1.77 | 0.002 | 0.122 | 0.997 | 2959 | tags=29%, list=23%, signal=38% |
| 56 | GOBP\_REGULATION\_OF\_GLIAL\_CELL\_DIFFERENTIATION | Details ... | 57 | -0.45 | -1.77 | 0.000 | 0.121 | 0.997 | 1983 | tags=35%, list=15%, signal=41% |
| 57 | GOBP\_RNA\_SPLICING | Details ... | 329 | -0.35 | -1.77 | 0.000 | 0.120 | 0.997 | 2692 | tags=34%, list=21%, signal=42% |
| 58 | GOBP\_MYELOID\_CELL\_HOMEOSTASIS | Details ... | 146 | -0.39 | -1.77 | 0.000 | 0.120 | 0.998 | 2078 | tags=29%, list=16%, signal=35% |
| 59 | GOBP\_REGULATION\_OF\_FATTY\_ACID\_OXIDATION | Details ... | 26 | -0.53 | -1.77 | 0.012 | 0.119 | 0.998 | 2398 | tags=46%, list=19%, signal=57% |
| 60 | GOBP\_POSITIVE\_REGULATION\_OF\_MRNA\_METABOLIC\_PROCESS | Details ... | 90 | -0.41 | -1.77 | 0.000 | 0.117 | 0.998 | 2525 | tags=37%, list=20%, signal=45% |
| 61 | GOBP\_PROTEIN\_DEMETHYLATION | Details ... | 30 | -0.53 | -1.77 | 0.002 | 0.116 | 0.998 | 1966 | tags=40%, list=15%, signal=47% |
| 62 | GOBP\_P38MAPK\_CASCADE | Details ... | 49 | -0.46 | -1.76 | 0.002 | 0.115 | 0.998 | 1983 | tags=33%, list=15%, signal=38% |
| 63 | GOBP\_POSITIVE\_REGULATION\_OF\_FATTY\_ACID\_METABOLIC\_PROCESS | Details ... | 33 | -0.51 | -1.76 | 0.003 | 0.114 | 0.998 | 2398 | tags=39%, list=19%, signal=48% |
| 64 | GOBP\_OVULATION | Details ... | 21 | -0.56 | -1.76 | 0.002 | 0.114 | 0.998 | 4378 | tags=76%, list=34%, signal=115% |
| 65 | GOBP\_MATURATION\_OF\_5\_8S\_RRNA\_FROM\_TRICISTRONIC\_RRNA\_TRANSCRIPT\_SSU\_RRNA\_5\_8S\_RRNA\_LSU\_RRNA | Details ... | 21 | -0.57 | -1.76 | 0.002 | 0.113 | 0.998 | 4584 | tags=86%, list=36%, signal=133% |
| 66 | GOBP\_MRNA\_PROCESSING | Details ... | 370 | -0.34 | -1.76 | 0.000 | 0.112 | 0.998 | 2715 | tags=34%, list=21%, signal=42% |
| 67 | GOBP\_CELL\_PROLIFERATION\_INVOLVED\_IN\_KIDNEY\_DEVELOPMENT | Details ... | 20 | -0.57 | -1.76 | 0.007 | 0.111 | 0.998 | 1983 | tags=40%, list=15%, signal=47% |
| 68 | GOBP\_EMBRYONIC\_HEMOPOIESIS | Details ... | 22 | -0.56 | -1.76 | 0.004 | 0.111 | 0.998 | 2246 | tags=50%, list=17%, signal=60% |
| 69 | GOBP\_NUCLEOSOME\_MOBILIZATION | Details ... | 17 | -0.61 | -1.75 | 0.007 | 0.116 | 0.998 | 2248 | tags=59%, list=17%, signal=71% |
| 70 | GOBP\_POSITIVE\_REGULATION\_OF\_MRNA\_PROCESSING | Details ... | 24 | -0.54 | -1.75 | 0.011 | 0.118 | 0.999 | 2027 | tags=38%, list=16%, signal=44% |
| 71 | GOBP\_ANGIOGENESIS\_INVOLVED\_IN\_WOUND\_HEALING | Details ... | 23 | -0.55 | -1.75 | 0.010 | 0.118 | 0.999 | 2618 | tags=48%, list=20%, signal=60% |
| 72 | GOBP\_ERYTHROCYTE\_HOMEOSTASIS | Details ... | 121 | -0.39 | -1.75 | 0.000 | 0.117 | 0.999 | 2022 | tags=29%, list=16%, signal=34% |
| 73 | GOBP\_RIBONUCLEOPROTEIN\_COMPLEX\_SUBUNIT\_ORGANIZATION | Details ... | 164 | -0.37 | -1.75 | 0.000 | 0.116 | 0.999 | 2905 | tags=40%, list=23%, signal=51% |
| 74 | GOBP\_REGULATION\_OF\_ENDOTHELIAL\_CELL\_DIFFERENTIATION | Details ... | 34 | -0.49 | -1.74 | 0.004 | 0.119 | 0.999 | 3137 | tags=47%, list=24%, signal=62% |
| 75 | GOBP\_REGULATION\_OF\_FATTY\_ACID\_BETA\_OXIDATION | Details ... | 15 | -0.62 | -1.74 | 0.009 | 0.117 | 0.999 | 1947 | tags=53%, list=15%, signal=63% |
| 76 | GOBP\_ESTABLISHMENT\_OF\_RNA\_LOCALIZATION | Details ... | 138 | -0.38 | -1.74 | 0.000 | 0.121 | 1.000 | 2712 | tags=35%, list=21%, signal=44% |
| 77 | GOBP\_PLACENTA\_BLOOD\_VESSEL\_DEVELOPMENT | Details ... | 28 | -0.51 | -1.74 | 0.007 | 0.120 | 1.000 | 2773 | tags=43%, list=21%, signal=54% |
| 78 | GOBP\_REPRODUCTIVE\_SYSTEM\_DEVELOPMENT | Details ... | 376 | -0.34 | -1.74 | 0.000 | 0.120 | 1.000 | 2979 | tags=30%, list=23%, signal=38% |
| 79 | GOBP\_CELLULAR\_RESPONSE\_TO\_INTERLEUKIN\_1 | Details ... | 94 | -0.40 | -1.74 | 0.000 | 0.119 | 1.000 | 3548 | tags=45%, list=28%, signal=61% |
| 80 | GOBP\_HEAD\_MORPHOGENESIS | Details ... | 29 | -0.52 | -1.73 | 0.016 | 0.121 | 1.000 | 2016 | tags=38%, list=16%, signal=45% |
| 81 | GOBP\_RESPONSE\_TO\_ATP | Details ... | 27 | -0.52 | -1.73 | 0.003 | 0.123 | 1.000 | 3768 | tags=59%, list=29%, signal=84% |
| 82 | GOBP\_GENE\_SILENCING\_BY\_RNA | Details ... | 91 | -0.40 | -1.73 | 0.000 | 0.123 | 1.000 | 2004 | tags=31%, list=16%, signal=36% |
| 83 | GOBP\_APOPTOTIC\_PROCESS\_INVOLVED\_IN\_MORPHOGENESIS | Details ... | 27 | -0.52 | -1.73 | 0.004 | 0.121 | 1.000 | 3890 | tags=56%, list=30%, signal=79% |
| 84 | GOBP\_ACTIN\_FILAMENT\_BASED\_TRANSPORT | Details ... | 17 | -0.58 | -1.73 | 0.009 | 0.122 | 1.000 | 3235 | tags=59%, list=25%, signal=78% |
| 85 | GOBP\_ESTABLISHMENT\_OF\_EPITHELIAL\_CELL\_POLARITY | Details ... | 25 | -0.54 | -1.73 | 0.002 | 0.121 | 1.000 | 2969 | tags=52%, list=23%, signal=67% |
| 86 | GOBP\_CELLULAR\_RESPONSE\_TO\_GONADOTROPIN\_STIMULUS | Details ... | 19 | -0.56 | -1.73 | 0.009 | 0.120 | 1.000 | 2724 | tags=37%, list=21%, signal=47% |
| 87 | GOBP\_PROTEIN\_DNA\_COMPLEX\_DISASSEMBLY | Details ... | 19 | -0.56 | -1.73 | 0.008 | 0.119 | 1.000 | 2936 | tags=47%, list=23%, signal=61% |
| 88 | GOBP\_CELLULAR\_RESPONSE\_TO\_ARSENIC\_CONTAINING\_SUBSTANCE | Details ... | 17 | -0.59 | -1.72 | 0.005 | 0.121 | 1.000 | 1713 | tags=53%, list=13%, signal=61% |
| 89 | GOBP\_HEART\_VALVE\_MORPHOGENESIS | Details ... | 48 | -0.45 | -1.72 | 0.003 | 0.123 | 1.000 | 3354 | tags=44%, list=26%, signal=59% |
| 90 | GOBP\_LYMPH\_VESSEL\_MORPHOGENESIS | Details ... | 19 | -0.56 | -1.72 | 0.005 | 0.122 | 1.000 | 4471 | tags=68%, list=35%, signal=105% |
| 91 | GOBP\_FIBROBLAST\_APOPTOTIC\_PROCESS | Details ... | 19 | -0.56 | -1.72 | 0.011 | 0.121 | 1.000 | 1635 | tags=32%, list=13%, signal=36% |
| 92 | GOBP\_POSITIVE\_REGULATION\_OF\_POSTTRANSCRIPTIONAL\_GENE\_SILENCING | Details ... | 26 | -0.52 | -1.72 | 0.007 | 0.120 | 1.000 | 2970 | tags=50%, list=23%, signal=65% |
| 93 | GOBP\_LYMPH\_VESSEL\_DEVELOPMENT | Details ... | 26 | -0.53 | -1.72 | 0.005 | 0.120 | 1.000 | 3478 | tags=50%, list=27%, signal=68% |
| 94 | GOBP\_HISTONE\_LYSINE\_DEMETHYLATION | Details ... | 25 | -0.53 | -1.72 | 0.005 | 0.123 | 1.000 | 1966 | tags=40%, list=15%, signal=47% |
| 95 | GOBP\_HEART\_VALVE\_DEVELOPMENT | Details ... | 57 | -0.43 | -1.71 | 0.003 | 0.128 | 1.000 | 4706 | tags=60%, list=36%, signal=93% |
| 96 | GOBP\_NUCLEAR\_TRANSPORT | Details ... | 262 | -0.35 | -1.71 | 0.000 | 0.130 | 1.000 | 2622 | tags=33%, list=20%, signal=41% |
| 97 | GOBP\_PLACENTA\_DEVELOPMENT | Details ... | 129 | -0.38 | -1.71 | 0.000 | 0.129 | 1.000 | 2842 | tags=36%, list=22%, signal=45% |
| 98 | GOBP\_SIGNAL\_TRANSDUCTION\_BY\_P53\_CLASS\_MEDIATOR | Details ... | 146 | -0.37 | -1.71 | 0.000 | 0.129 | 1.000 | 1857 | tags=27%, list=14%, signal=31% |
| 99 | GOBP\_SMOOTH\_MUSCLE\_CELL\_DIFFERENTIATION | Details ... | 50 | -0.44 | -1.71 | 0.002 | 0.129 | 1.000 | 2124 | tags=36%, list=16%, signal=43% |
| 100 | GOBP\_POSITIVE\_REGULATION\_OF\_TRANSCRIPTION\_BY\_RNA\_POLYMERASE\_I | Details ... | 18 | -0.58 | -1.71 | 0.012 | 0.128 | 1.000 | 3563 | tags=67%, list=28%, signal=92% |
| 101 | GOBP\_POST\_EMBRYONIC\_DEVELOPMENT |  | 76 | -0.41 | -1.71 | 0.003 | 0.127 | 1.000 | 2122 | tags=30%, list=16%, signal=36% |
| 102 | GOBP\_FACE\_DEVELOPMENT |  | 44 | -0.46 | -1.70 | 0.004 | 0.126 | 1.000 | 1161 | tags=27%, list=9%, signal=30% |
| 103 | GOBP\_REGULATION\_OF\_ANOIKIS |  | 23 | -0.54 | -1.70 | 0.016 | 0.130 | 1.000 | 1714 | tags=30%, list=13%, signal=35% |
| 104 | GOBP\_NEGATIVE\_REGULATION\_OF\_NOTCH\_SIGNALING\_PATHWAY |  | 30 | -0.50 | -1.70 | 0.011 | 0.129 | 1.000 | 3811 | tags=60%, list=30%, signal=85% |
| 105 | GOBP\_TIGHT\_JUNCTION\_ORGANIZATION |  | 62 | -0.43 | -1.70 | 0.000 | 0.128 | 1.000 | 3205 | tags=40%, list=25%, signal=53% |
| 106 | GOBP\_APICAL\_JUNCTION\_ASSEMBLY |  | 58 | -0.42 | -1.70 | 0.002 | 0.128 | 1.000 | 3205 | tags=41%, list=25%, signal=55% |
| 107 | GOBP\_CLEAVAGE\_INVOLVED\_IN\_RRNA\_PROCESSING |  | 21 | -0.54 | -1.70 | 0.015 | 0.126 | 1.000 | 2541 | tags=52%, list=20%, signal=65% |
| 108 | GOBP\_CELL\_MIGRATION\_INVOLVED\_IN\_SPROUTING\_ANGIOGENESIS |  | 50 | -0.44 | -1.69 | 0.007 | 0.132 | 1.000 | 2915 | tags=44%, list=23%, signal=57% |
| 109 | GOBP\_REGULATION\_OF\_MUSCLE\_HYPERTROPHY |  | 48 | -0.44 | -1.69 | 0.003 | 0.135 | 1.000 | 2461 | tags=40%, list=19%, signal=49% |
| 110 | GOBP\_POSITIVE\_REGULATION\_OF\_PROTEIN\_EXPORT\_FROM\_NUCLEUS |  | 16 | -0.59 | -1.69 | 0.012 | 0.134 | 1.000 | 2575 | tags=56%, list=20%, signal=70% |
| 111 | GOBP\_MRNA\_EXPORT\_FROM\_NUCLEUS |  | 51 | -0.44 | -1.69 | 0.007 | 0.134 | 1.000 | 2919 | tags=39%, list=23%, signal=50% |
| 112 | GOBP\_NEGATIVE\_REGULATION\_OF\_ANOIKIS |  | 17 | -0.58 | -1.69 | 0.009 | 0.134 | 1.000 | 428 | tags=24%, list=3%, signal=24% |
| 113 | GOBP\_MANGANESE\_ION\_TRANSPORT |  | 16 | -0.57 | -1.69 | 0.012 | 0.134 | 1.000 | 4103 | tags=69%, list=32%, signal=101% |
| 114 | GOBP\_RETINA\_VASCULATURE\_DEVELOPMENT\_IN\_CAMERA\_TYPE\_EYE |  | 17 | -0.57 | -1.69 | 0.005 | 0.134 | 1.000 | 2458 | tags=47%, list=19%, signal=58% |
| 115 | GOBP\_ENERGY\_HOMEOSTASIS |  | 31 | -0.49 | -1.68 | 0.016 | 0.137 | 1.000 | 5169 | tags=74%, list=40%, signal=123% |
| 116 | GOBP\_REGULATION\_OF\_MEMBRANE\_PROTEIN\_ECTODOMAIN\_PROTEOLYSIS |  | 20 | -0.55 | -1.68 | 0.015 | 0.136 | 1.000 | 2112 | tags=40%, list=16%, signal=48% |
| 117 | GOBP\_HISTONE\_MODIFICATION |  | 409 | -0.32 | -1.68 | 0.000 | 0.137 | 1.000 | 2104 | tags=25%, list=16%, signal=29% |
| 118 | GOBP\_ADENYLATE\_CYCLASE\_INHIBITING\_G\_PROTEIN\_COUPLED\_RECEPTOR\_SIGNALING\_PATHWAY |  | 70 | -0.41 | -1.68 | 0.003 | 0.136 | 1.000 | 5097 | tags=61%, list=40%, signal=101% |
| 119 | GOBP\_POLYOL\_CATABOLIC\_PROCESS |  | 24 | -0.52 | -1.68 | 0.009 | 0.137 | 1.000 | 2611 | tags=33%, list=20%, signal=42% |
| 120 | GOBP\_REGULATION\_OF\_TOLERANCE\_INDUCTION |  | 18 | -0.57 | -1.68 | 0.009 | 0.136 | 1.000 | 2318 | tags=44%, list=18%, signal=54% |
| 121 | GOBP\_REGULATION\_OF\_EPITHELIAL\_CELL\_DIFFERENTIATION |  | 124 | -0.37 | -1.68 | 0.000 | 0.138 | 1.000 | 1756 | tags=23%, list=14%, signal=26% |
| 122 | GOBP\_REGULATION\_OF\_FIBROBLAST\_GROWTH\_FACTOR\_RECEPTOR\_SIGNALING\_PATHWAY |  | 20 | -0.54 | -1.68 | 0.009 | 0.137 | 1.000 | 1806 | tags=35%, list=14%, signal=41% |
| 123 | GOBP\_REGULATION\_OF\_ERYTHROCYTE\_DIFFERENTIATION |  | 41 | -0.45 | -1.67 | 0.007 | 0.139 | 1.000 | 1944 | tags=32%, list=15%, signal=37% |
| 124 | GOBP\_VASCULOGENESIS |  | 71 | -0.41 | -1.67 | 0.000 | 0.141 | 1.000 | 2122 | tags=27%, list=16%, signal=32% |
| 125 | GOBP\_ENDODERMAL\_CELL\_DIFFERENTIATION |  | 40 | -0.46 | -1.67 | 0.005 | 0.142 | 1.000 | 2059 | tags=35%, list=16%, signal=42% |
| 126 | GOBP\_EPOXYGENASE\_P450\_PATHWAY |  | 16 | -0.57 | -1.67 | 0.009 | 0.143 | 1.000 | 5053 | tags=75%, list=39%, signal=123% |
| 127 | GOBP\_POSITIVE\_REGULATION\_OF\_GLIOGENESIS |  | 51 | -0.44 | -1.67 | 0.005 | 0.146 | 1.000 | 2145 | tags=35%, list=17%, signal=42% |
| 128 | GOBP\_REGULATION\_OF\_NEURON\_MIGRATION |  | 34 | -0.47 | -1.66 | 0.007 | 0.146 | 1.000 | 2189 | tags=38%, list=17%, signal=46% |
| 129 | GOBP\_BLASTOCYST\_FORMATION |  | 36 | -0.47 | -1.66 | 0.007 | 0.145 | 1.000 | 4349 | tags=53%, list=34%, signal=79% |
| 130 | GOBP\_NEGATIVE\_REGULATION\_OF\_LYMPHOCYTE\_DIFFERENTIATION |  | 47 | -0.44 | -1.66 | 0.008 | 0.144 | 1.000 | 2202 | tags=36%, list=17%, signal=43% |
| 131 | GOBP\_ESTABLISHMENT\_OF\_CELL\_POLARITY |  | 119 | -0.37 | -1.66 | 0.000 | 0.147 | 1.000 | 4560 | tags=55%, list=35%, signal=85% |
| 132 | GOBP\_ENDOCRINE\_HORMONE\_SECRETION |  | 45 | -0.44 | -1.66 | 0.005 | 0.147 | 1.000 | 4743 | tags=67%, list=37%, signal=105% |
| 133 | GOBP\_KERATINOCYTE\_PROLIFERATION |  | 38 | -0.46 | -1.66 | 0.007 | 0.146 | 1.000 | 2604 | tags=32%, list=20%, signal=39% |
| 134 | GOBP\_PLATELET\_DERIVED\_GROWTH\_FACTOR\_RECEPTOR\_SIGNALING\_PATHWAY |  | 49 | -0.44 | -1.66 | 0.008 | 0.148 | 1.000 | 2280 | tags=37%, list=18%, signal=44% |
| 135 | GOBP\_REGULATION\_OF\_EXTRINSIC\_APOPTOTIC\_SIGNALING\_PATHWAY\_VIA\_DEATH\_DOMAIN\_RECEPTORS |  | 41 | -0.45 | -1.66 | 0.002 | 0.148 | 1.000 | 3394 | tags=49%, list=26%, signal=66% |
| 136 | GOBP\_POSTTRANSCRIPTIONAL\_REGULATION\_OF\_GENE\_EXPRESSION |  | 406 | -0.32 | -1.66 | 0.000 | 0.148 | 1.000 | 3279 | tags=36%, list=25%, signal=47% |
| 137 | GOBP\_NEGATIVE\_REGULATION\_OF\_STEM\_CELL\_DIFFERENTIATION |  | 18 | -0.56 | -1.65 | 0.013 | 0.149 | 1.000 | 3977 | tags=72%, list=31%, signal=104% |
| 138 | GOBP\_NEGATIVE\_REGULATION\_OF\_EPITHELIAL\_CELL\_APOPTOTIC\_PROCESS |  | 47 | -0.44 | -1.65 | 0.008 | 0.149 | 1.000 | 3162 | tags=43%, list=25%, signal=56% |
| 139 | GOBP\_RNA\_LOCALIZATION |  | 169 | -0.35 | -1.65 | 0.003 | 0.155 | 1.000 | 2712 | tags=33%, list=21%, signal=41% |
| 140 | GOBP\_REGULATION\_OF\_ENDOCRINE\_PROCESS |  | 34 | -0.47 | -1.65 | 0.005 | 0.154 | 1.000 | 4742 | tags=65%, list=37%, signal=102% |
| 141 | GOBP\_RESPONSE\_TO\_IMMOBILIZATION\_STRESS |  | 17 | -0.55 | -1.65 | 0.027 | 0.155 | 1.000 | 2262 | tags=47%, list=18%, signal=57% |
| 142 | GOBP\_POSITIVE\_REGULATION\_OF\_CELLULAR\_RESPONSE\_TO\_TRANSFORMING\_GROWTH\_FACTOR\_BETA\_STIMULUS |  | 28 | -0.50 | -1.65 | 0.010 | 0.155 | 1.000 | 2842 | tags=46%, list=22%, signal=59% |
| 143 | GOBP\_ENDOTHELIAL\_CELL\_DEVELOPMENT |  | 61 | -0.41 | -1.65 | 0.003 | 0.154 | 1.000 | 4379 | tags=54%, list=34%, signal=82% |
| 144 | GOBP\_SEMI\_LUNAR\_VALVE\_DEVELOPMENT |  | 37 | -0.46 | -1.64 | 0.008 | 0.154 | 1.000 | 3354 | tags=49%, list=26%, signal=66% |
| 145 | GOBP\_MATURATION\_OF\_SSU\_RRNA |  | 39 | -0.46 | -1.64 | 0.012 | 0.162 | 1.000 | 2377 | tags=38%, list=18%, signal=47% |
| 146 | GOBP\_CELLULAR\_RESPONSE\_TO\_GLUCOSE\_STARVATION |  | 40 | -0.44 | -1.64 | 0.003 | 0.162 | 1.000 | 2877 | tags=45%, list=22%, signal=58% |
| 147 | GOBP\_PROTEIN\_AUTOPHOSPHORYLATION |  | 197 | -0.34 | -1.64 | 0.000 | 0.162 | 1.000 | 3846 | tags=41%, list=30%, signal=57% |
| 148 | GOBP\_LABYRINTHINE\_LAYER\_BLOOD\_VESSEL\_DEVELOPMENT |  | 18 | -0.54 | -1.64 | 0.016 | 0.161 | 1.000 | 1963 | tags=33%, list=15%, signal=39% |
| 149 | GOBP\_REGULATION\_OF\_ORGAN\_GROWTH |  | 79 | -0.39 | -1.64 | 0.000 | 0.161 | 1.000 | 1947 | tags=29%, list=15%, signal=34% |
| 150 | GOBP\_REGULATION\_OF\_ESTABLISHMENT\_OR\_MAINTENANCE\_OF\_CELL\_POLARITY |  | 22 | -0.53 | -1.64 | 0.012 | 0.161 | 1.000 | 3222 | tags=55%, list=25%, signal=73% |
| 151 | GOBP\_NEGATIVE\_REGULATION\_OF\_CELLULAR\_SENESCENCE |  | 17 | -0.55 | -1.63 | 0.032 | 0.161 | 1.000 | 5126 | tags=76%, list=40%, signal=127% |
| 152 | GOBP\_MUSCLE\_CELL\_APOPTOTIC\_PROCESS |  | 62 | -0.41 | -1.63 | 0.012 | 0.161 | 1.000 | 1979 | tags=27%, list=15%, signal=32% |
| 153 | GOBP\_OVARIAN\_FOLLICLE\_DEVELOPMENT |  | 50 | -0.43 | -1.63 | 0.005 | 0.160 | 1.000 | 2250 | tags=24%, list=17%, signal=29% |
| 154 | GOBP\_REGULATION\_OF\_OSTEOBLAST\_DIFFERENTIATION |  | 105 | -0.37 | -1.63 | 0.003 | 0.160 | 1.000 | 1983 | tags=26%, list=15%, signal=30% |
| 155 | GOBP\_NEGATIVE\_REGULATION\_OF\_MUSCLE\_CELL\_APOPTOTIC\_PROCESS |  | 32 | -0.47 | -1.63 | 0.015 | 0.159 | 1.000 | 1713 | tags=34%, list=13%, signal=40% |
| 156 | GOBP\_MRNA\_CATABOLIC\_PROCESS |  | 183 | -0.34 | -1.63 | 0.000 | 0.159 | 1.000 | 3151 | tags=36%, list=24%, signal=47% |
| 157 | GOBP\_NEGATIVE\_REGULATION\_OF\_BLOOD\_PRESSURE |  | 43 | -0.44 | -1.63 | 0.008 | 0.158 | 1.000 | 1951 | tags=33%, list=15%, signal=38% |
| 158 | GOBP\_LYMPHANGIOGENESIS |  | 15 | -0.58 | -1.63 | 0.025 | 0.160 | 1.000 | 3354 | tags=53%, list=26%, signal=72% |
| 159 | GOBP\_METANEPHROS\_DEVELOPMENT |  | 76 | -0.39 | -1.63 | 0.010 | 0.159 | 1.000 | 4205 | tags=47%, list=33%, signal=70% |
| 160 | GOBP\_CARDIAC\_EPITHELIAL\_TO\_MESENCHYMAL\_TRANSITION |  | 35 | -0.45 | -1.63 | 0.010 | 0.159 | 1.000 | 1983 | tags=31%, list=15%, signal=37% |
| 161 | GOBP\_REGULATION\_OF\_GLIAL\_CELL\_PROLIFERATION |  | 25 | -0.50 | -1.63 | 0.024 | 0.160 | 1.000 | 797 | tags=28%, list=6%, signal=30% |
| 162 | GOBP\_REGULATION\_OF\_ALTERNATIVE\_MRNA\_SPLICING\_VIA\_SPLICEOSOME |  | 49 | -0.42 | -1.63 | 0.008 | 0.161 | 1.000 | 2403 | tags=39%, list=19%, signal=47% |
| 163 | GOBP\_INSULIN\_RECEPTOR\_SIGNALING\_PATHWAY |  | 102 | -0.37 | -1.63 | 0.005 | 0.161 | 1.000 | 2713 | tags=35%, list=21%, signal=44% |
| 164 | GOBP\_REGULATION\_OF\_EMBRYONIC\_DEVELOPMENT |  | 46 | -0.43 | -1.63 | 0.005 | 0.160 | 1.000 | 2196 | tags=35%, list=17%, signal=42% |
| 165 | GOBP\_NEGATIVE\_REGULATION\_OF\_RNA\_SPLICING |  | 23 | -0.51 | -1.63 | 0.020 | 0.160 | 1.000 | 2643 | tags=43%, list=20%, signal=55% |
| 166 | GOBP\_MONOCYTE\_DIFFERENTIATION |  | 31 | -0.47 | -1.62 | 0.015 | 0.160 | 1.000 | 843 | tags=19%, list=7%, signal=21% |
| 167 | GOBP\_RESPONSE\_TO\_EPIDERMAL\_GROWTH\_FACTOR |  | 42 | -0.44 | -1.62 | 0.014 | 0.161 | 1.000 | 2393 | tags=33%, list=19%, signal=41% |
| 168 | GOBP\_CIRCADIAN\_REGULATION\_OF\_GENE\_EXPRESSION |  | 65 | -0.40 | -1.62 | 0.003 | 0.160 | 1.000 | 1805 | tags=34%, list=14%, signal=39% |
| 169 | GOBP\_NEGATIVE\_REGULATION\_OF\_ORGAN\_GROWTH |  | 28 | -0.48 | -1.62 | 0.010 | 0.160 | 1.000 | 2564 | tags=43%, list=20%, signal=53% |
| 170 | GOBP\_FEMALE\_GAMETE\_GENERATION |  | 117 | -0.36 | -1.62 | 0.002 | 0.159 | 1.000 | 3030 | tags=32%, list=23%, signal=41% |
| 171 | GOBP\_PROTEIN\_ACETYLATION |  | 179 | -0.34 | -1.62 | 0.000 | 0.159 | 1.000 | 2095 | tags=28%, list=16%, signal=34% |
| 172 | GOBP\_GONADOTROPIN\_SECRETION |  | 15 | -0.57 | -1.62 | 0.031 | 0.159 | 1.000 | 4742 | tags=73%, list=37%, signal=116% |
| 173 | GOBP\_LIVER\_MORPHOGENESIS |  | 21 | -0.52 | -1.62 | 0.022 | 0.159 | 1.000 | 2428 | tags=43%, list=19%, signal=53% |
| 174 | GOBP\_EPITHELIAL\_TO\_MESENCHYMAL\_TRANSITION |  | 126 | -0.36 | -1.62 | 0.000 | 0.159 | 1.000 | 2270 | tags=29%, list=18%, signal=35% |
| 175 | GOBP\_CELL\_MIGRATION\_IN\_HINDBRAIN |  | 16 | -0.56 | -1.62 | 0.020 | 0.158 | 1.000 | 2307 | tags=50%, list=18%, signal=61% |
| 176 | GOBP\_ANIMAL\_ORGAN\_REGENERATION |  | 63 | -0.41 | -1.62 | 0.006 | 0.158 | 1.000 | 2686 | tags=38%, list=21%, signal=48% |
| 177 | GOBP\_NUCLEUS\_LOCALIZATION |  | 21 | -0.53 | -1.62 | 0.023 | 0.157 | 1.000 | 2779 | tags=48%, list=22%, signal=61% |
| 178 | GOBP\_TRANSCRIPTION\_PREINITIATION\_COMPLEX\_ASSEMBLY |  | 35 | -0.46 | -1.62 | 0.011 | 0.157 | 1.000 | 1764 | tags=29%, list=14%, signal=33% |
| 179 | GOBP\_POSITIVE\_REGULATION\_OF\_VASCULAR\_ENDOTHELIAL\_GROWTH\_FACTOR\_PRODUCTION |  | 24 | -0.50 | -1.62 | 0.025 | 0.156 | 1.000 | 1069 | tags=33%, list=8%, signal=36% |
| 180 | GOBP\_TRANSEPITHELIAL\_TRANSPORT |  | 31 | -0.47 | -1.62 | 0.013 | 0.156 | 1.000 | 3246 | tags=42%, list=25%, signal=56% |
| 181 | GOBP\_MOLTING\_CYCLE |  | 87 | -0.38 | -1.62 | 0.000 | 0.156 | 1.000 | 1955 | tags=25%, list=15%, signal=30% |
| 182 | GOBP\_NEGATIVE\_REGULATION\_OF\_EPITHELIAL\_CELL\_DIFFERENTIATION |  | 35 | -0.45 | -1.62 | 0.023 | 0.156 | 1.000 | 729 | tags=20%, list=6%, signal=21% |
| 183 | GOBP\_POSITIVE\_REGULATION\_OF\_RESPONSE\_TO\_WOUNDING |  | 60 | -0.40 | -1.62 | 0.008 | 0.156 | 1.000 | 2996 | tags=40%, list=23%, signal=52% |
| 184 | GOBP\_RNA\_DESTABILIZATION |  | 66 | -0.40 | -1.61 | 0.007 | 0.157 | 1.000 | 2525 | tags=38%, list=20%, signal=47% |
| 185 | GOBP\_REGULATION\_OF\_GLIOGENESIS |  | 80 | -0.39 | -1.61 | 0.005 | 0.163 | 1.000 | 2145 | tags=29%, list=17%, signal=34% |
| 186 | GOBP\_RESPONSE\_TO\_INTERLEUKIN\_4 |  | 30 | -0.47 | -1.61 | 0.019 | 0.162 | 1.000 | 2082 | tags=37%, list=16%, signal=44% |
| 187 | GOBP\_POSITIVE\_REGULATION\_OF\_P38MAPK\_CASCADE |  | 26 | -0.49 | -1.61 | 0.018 | 0.161 | 1.000 | 4691 | tags=73%, list=36%, signal=115% |
| 188 | GOBP\_HISTONE\_H3\_DEACETYLATION |  | 16 | -0.55 | -1.61 | 0.021 | 0.162 | 1.000 | 5239 | tags=81%, list=41%, signal=137% |
| 189 | GOBP\_POSITIVE\_REGULATION\_OF\_T\_CELL\_CYTOKINE\_PRODUCTION |  | 26 | -0.49 | -1.61 | 0.022 | 0.161 | 1.000 | 797 | tags=27%, list=6%, signal=29% |
| 190 | GOBP\_POSITIVE\_REGULATION\_OF\_SPROUTING\_ANGIOGENESIS |  | 16 | -0.57 | -1.61 | 0.024 | 0.161 | 1.000 | 4598 | tags=75%, list=36%, signal=116% |
| 191 | GOBP\_ANOIKIS |  | 32 | -0.47 | -1.61 | 0.027 | 0.160 | 1.000 | 1916 | tags=31%, list=15%, signal=37% |
| 192 | GOBP\_MUSCLE\_CELL\_PROLIFERATION |  | 175 | -0.34 | -1.60 | 0.002 | 0.163 | 1.000 | 2126 | tags=24%, list=16%, signal=28% |
| 193 | GOBP\_POSITIVE\_REGULATION\_OF\_ERBB\_SIGNALING\_PATHWAY |  | 34 | -0.46 | -1.60 | 0.015 | 0.162 | 1.000 | 2514 | tags=38%, list=19%, signal=47% |
| 194 | GOBP\_REGULATION\_OF\_VASCULAR\_PERMEABILITY |  | 43 | -0.43 | -1.60 | 0.010 | 0.162 | 1.000 | 4977 | tags=70%, list=39%, signal=113% |
| 195 | GOBP\_IN\_UTERO\_EMBRYONIC\_DEVELOPMENT |  | 323 | -0.32 | -1.60 | 0.000 | 0.161 | 1.000 | 2263 | tags=25%, list=18%, signal=30% |
| 196 | GOBP\_RESPONSE\_TO\_INTERLEUKIN\_1 |  | 119 | -0.36 | -1.60 | 0.000 | 0.163 | 1.000 | 3548 | tags=40%, list=28%, signal=55% |
| 197 | GOBP\_CELL\_CELL\_JUNCTION\_ORGANIZATION |  | 168 | -0.34 | -1.60 | 0.000 | 0.162 | 1.000 | 4475 | tags=45%, list=35%, signal=68% |
| 198 | GOBP\_DIGESTIVE\_TRACT\_MORPHOGENESIS |  | 42 | -0.44 | -1.60 | 0.009 | 0.165 | 1.000 | 2059 | tags=31%, list=16%, signal=37% |
| 199 | GOBP\_CELL\_CELL\_ADHESION\_MEDIATED\_BY\_CADHERIN |  | 22 | -0.51 | -1.60 | 0.025 | 0.167 | 1.000 | 3006 | tags=50%, list=23%, signal=65% |
| 200 | GOBP\_POSITIVE\_REGULATION\_OF\_CYTOKINESIS |  | 34 | -0.45 | -1.60 | 0.027 | 0.168 | 1.000 | 1474 | tags=24%, list=11%, signal=26% |
| 201 | GOBP\_POST\_EMBRYONIC\_ANIMAL\_ORGAN\_DEVELOPMENT |  | 15 | -0.55 | -1.60 | 0.025 | 0.167 | 1.000 | 3636 | tags=53%, list=28%, signal=74% |
| 202 | GOBP\_VASCULAR\_TRANSPORT |  | 77 | -0.39 | -1.60 | 0.008 | 0.166 | 1.000 | 3556 | tags=42%, list=28%, signal=57% |
| 203 | GOBP\_REGULATION\_OF\_TRANSLATIONAL\_FIDELITY |  | 15 | -0.57 | -1.60 | 0.030 | 0.167 | 1.000 | 2821 | tags=53%, list=22%, signal=68% |
| 204 | GOBP\_RESPONSE\_TO\_PURINE\_CONTAINING\_COMPOUND |  | 122 | -0.36 | -1.59 | 0.003 | 0.168 | 1.000 | 3859 | tags=42%, list=30%, signal=59% |
| 205 | GOBP\_PROTEIN\_K48\_LINKED\_DEUBIQUITINATION |  | 29 | -0.48 | -1.59 | 0.017 | 0.168 | 1.000 | 1847 | tags=31%, list=14%, signal=36% |
| 206 | GOBP\_ESTABLISHMENT\_OF\_ENDOTHELIAL\_BARRIER |  | 49 | -0.42 | -1.59 | 0.019 | 0.168 | 1.000 | 4379 | tags=53%, list=34%, signal=80% |
| 207 | GOBP\_OOCYTE\_DIFFERENTIATION |  | 46 | -0.42 | -1.59 | 0.010 | 0.168 | 1.000 | 2696 | tags=35%, list=21%, signal=44% |
| 208 | GOBP\_NUCLEOLAR\_LARGE\_RRNA\_TRANSCRIPTION\_BY\_RNA\_POLYMERASE\_I |  | 18 | -0.53 | -1.59 | 0.023 | 0.167 | 1.000 | 4178 | tags=61%, list=32%, signal=90% |
| 209 | GOBP\_POSITIVE\_REGULATION\_OF\_GLIAL\_CELL\_DIFFERENTIATION |  | 33 | -0.46 | -1.59 | 0.015 | 0.167 | 1.000 | 1983 | tags=36%, list=15%, signal=43% |
| 210 | GOBP\_NEGATIVE\_REGULATION\_OF\_MRNA\_PROCESSING |  | 26 | -0.48 | -1.59 | 0.026 | 0.167 | 1.000 | 2243 | tags=38%, list=17%, signal=46% |
| 211 | GOBP\_POSITIVE\_REGULATION\_OF\_REGULATORY\_T\_CELL\_DIFFERENTIATION |  | 18 | -0.53 | -1.59 | 0.027 | 0.167 | 1.000 | 2033 | tags=28%, list=16%, signal=33% |
| 212 | GOBP\_INTERLEUKIN\_6\_MEDIATED\_SIGNALING\_PATHWAY |  | 17 | -0.55 | -1.59 | 0.022 | 0.167 | 1.000 | 1632 | tags=35%, list=13%, signal=40% |
| 213 | GOBP\_NEGATIVE\_REGULATION\_OF\_GLIAL\_CELL\_DIFFERENTIATION |  | 20 | -0.52 | -1.59 | 0.024 | 0.166 | 1.000 | 1966 | tags=40%, list=15%, signal=47% |
| 214 | GOBP\_PEPTIDYL\_TYROSINE\_AUTOPHOSPHORYLATION |  | 20 | -0.52 | -1.59 | 0.028 | 0.166 | 1.000 | 1448 | tags=35%, list=11%, signal=39% |
| 215 | GOBP\_FORMATION\_OF\_PRIMARY\_GERM\_LAYER |  | 99 | -0.37 | -1.59 | 0.000 | 0.165 | 1.000 | 2997 | tags=39%, list=23%, signal=51% |
| 216 | GOBP\_LABYRINTHINE\_LAYER\_DEVELOPMENT |  | 43 | -0.43 | -1.59 | 0.012 | 0.164 | 1.000 | 2824 | tags=35%, list=22%, signal=45% |
| 217 | GOBP\_BLOOD\_VESSEL\_ENDOTHELIAL\_CELL\_MIGRATION |  | 110 | -0.36 | -1.59 | 0.006 | 0.164 | 1.000 | 2956 | tags=37%, list=23%, signal=48% |
| 218 | GOBP\_REGULATION\_OF\_CELL\_CELL\_ADHESION\_MEDIATED\_BY\_CADHERIN |  | 16 | -0.54 | -1.59 | 0.035 | 0.163 | 1.000 | 2064 | tags=44%, list=16%, signal=52% |
| 219 | GOBP\_REGULATION\_OF\_HISTONE\_DEACETYLATION |  | 33 | -0.46 | -1.59 | 0.022 | 0.163 | 1.000 | 723 | tags=21%, list=6%, signal=22% |
| 220 | GOBP\_RESPONSE\_TO\_INTERLEUKIN\_6 |  | 36 | -0.45 | -1.59 | 0.021 | 0.163 | 1.000 | 1632 | tags=28%, list=13%, signal=32% |
| 221 | GOBP\_RENAL\_TUBULAR\_SECRETION |  | 25 | -0.48 | -1.59 | 0.023 | 0.164 | 1.000 | 1626 | tags=28%, list=13%, signal=32% |
| 222 | GOBP\_NEGATIVE\_REGULATION\_OF\_INTERLEUKIN\_2\_PRODUCTION |  | 25 | -0.48 | -1.59 | 0.020 | 0.163 | 1.000 | 1733 | tags=24%, list=13%, signal=28% |
| 223 | GOBP\_SMALL\_GTPASE\_MEDIATED\_SIGNAL\_TRANSDUCTION |  | 401 | -0.30 | -1.59 | 0.000 | 0.163 | 1.000 | 2442 | tags=26%, list=19%, signal=31% |
| 224 | GOBP\_T\_CELL\_TOLERANCE\_INDUCTION |  | 15 | -0.56 | -1.59 | 0.026 | 0.163 | 1.000 | 2582 | tags=47%, list=20%, signal=58% |
| 225 | GOBP\_REGULATION\_OF\_CELL\_MIGRATION\_INVOLVED\_IN\_SPROUTING\_ANGIOGENESIS |  | 35 | -0.44 | -1.59 | 0.010 | 0.162 | 1.000 | 2078 | tags=34%, list=16%, signal=41% |
| 226 | GOBP\_REGULATION\_OF\_CELLULAR\_AMIDE\_METABOLIC\_PROCESS |  | 358 | -0.31 | -1.59 | 0.000 | 0.162 | 1.000 | 3279 | tags=35%, list=25%, signal=46% |
| 227 | GOBP\_REGULATION\_OF\_NEURON\_PROJECTION\_REGENERATION |  | 25 | -0.49 | -1.59 | 0.021 | 0.162 | 1.000 | 1885 | tags=40%, list=15%, signal=47% |
| 228 | GOBP\_HEART\_GROWTH |  | 71 | -0.39 | -1.59 | 0.005 | 0.162 | 1.000 | 3027 | tags=37%, list=23%, signal=48% |
| 229 | GOBP\_REGULATION\_OF\_BICELLULAR\_TIGHT\_JUNCTION\_ASSEMBLY |  | 19 | -0.53 | -1.59 | 0.028 | 0.161 | 1.000 | 3205 | tags=53%, list=25%, signal=70% |
| 230 | GOBP\_REGULATION\_OF\_HEART\_GROWTH |  | 56 | -0.40 | -1.58 | 0.007 | 0.161 | 1.000 | 1947 | tags=30%, list=15%, signal=36% |
| 231 | GOBP\_3\_UTR\_MEDIATED\_MRNA\_STABILIZATION |  | 17 | -0.54 | -1.58 | 0.028 | 0.162 | 1.000 | 2899 | tags=41%, list=22%, signal=53% |
| 232 | GOBP\_POSITIVE\_REGULATION\_OF\_EPITHELIAL\_CELL\_PROLIFERATION |  | 165 | -0.34 | -1.58 | 0.000 | 0.163 | 1.000 | 2260 | tags=28%, list=18%, signal=34% |
| 233 | GOBP\_CELLULAR\_RESPONSE\_TO\_CORTICOSTEROID\_STIMULUS |  | 52 | -0.41 | -1.58 | 0.013 | 0.163 | 1.000 | 3424 | tags=48%, list=27%, signal=65% |
| 234 | GOBP\_REGULATION\_OF\_CYTOPLASMIC\_TRANSLATION |  | 20 | -0.51 | -1.58 | 0.023 | 0.164 | 1.000 | 3279 | tags=55%, list=25%, signal=74% |
| 235 | GOBP\_REGULATION\_OF\_POSITIVE\_CHEMOTAXIS |  | 24 | -0.49 | -1.58 | 0.019 | 0.163 | 1.000 | 1807 | tags=38%, list=14%, signal=44% |
| 236 | GOBP\_SEX\_DIFFERENTIATION |  | 237 | -0.32 | -1.58 | 0.000 | 0.165 | 1.000 | 3636 | tags=33%, list=28%, signal=46% |
| 237 | GOBP\_MORPHOGENESIS\_OF\_AN\_EPITHELIAL\_SHEET |  | 49 | -0.41 | -1.58 | 0.007 | 0.165 | 1.000 | 2182 | tags=33%, list=17%, signal=39% |
| 238 | GOBP\_RENAL\_TUBULE\_DEVELOPMENT |  | 76 | -0.38 | -1.58 | 0.008 | 0.164 | 1.000 | 2427 | tags=28%, list=19%, signal=34% |
| 239 | GOBP\_BODY\_MORPHOGENESIS |  | 39 | -0.43 | -1.58 | 0.012 | 0.164 | 1.000 | 2159 | tags=33%, list=17%, signal=40% |
| 240 | GOBP\_ARF\_PROTEIN\_SIGNAL\_TRANSDUCTION |  | 17 | -0.53 | -1.58 | 0.025 | 0.167 | 1.000 | 2768 | tags=53%, list=21%, signal=67% |
| 241 | GOBP\_PROTEIN\_ACYLATION |  | 203 | -0.33 | -1.58 | 0.001 | 0.166 | 1.000 | 2095 | tags=28%, list=16%, signal=33% |
| 242 | GOBP\_TRANSCRIPTION\_BY\_RNA\_POLYMERASE\_I |  | 45 | -0.43 | -1.57 | 0.028 | 0.167 | 1.000 | 3563 | tags=53%, list=28%, signal=73% |
| 243 | GOBP\_TELENCEPHALON\_GLIAL\_CELL\_MIGRATION |  | 21 | -0.49 | -1.57 | 0.012 | 0.166 | 1.000 | 2554 | tags=38%, list=20%, signal=47% |
| 244 | GOBP\_HISTONE\_H3\_K27\_METHYLATION |  | 15 | -0.55 | -1.57 | 0.029 | 0.166 | 1.000 | 1216 | tags=27%, list=9%, signal=29% |
| 245 | GOBP\_INTRINSIC\_APOPTOTIC\_SIGNALING\_PATHWAY\_IN\_RESPONSE\_TO\_DNA\_DAMAGE |  | 90 | -0.37 | -1.57 | 0.002 | 0.167 | 1.000 | 3270 | tags=37%, list=25%, signal=49% |
| 246 | GOBP\_REGULATION\_OF\_KERATINOCYTE\_PROLIFERATION |  | 29 | -0.47 | -1.57 | 0.023 | 0.167 | 1.000 | 2604 | tags=31%, list=20%, signal=39% |
| 247 | GOBP\_MESODERMAL\_CELL\_DIFFERENTIATION |  | 25 | -0.47 | -1.57 | 0.012 | 0.167 | 1.000 | 2997 | tags=48%, list=23%, signal=62% |
| 248 | GOBP\_SMOOTH\_MUSCLE\_CELL\_PROLIFERATION |  | 131 | -0.35 | -1.57 | 0.003 | 0.167 | 1.000 | 2678 | tags=28%, list=21%, signal=35% |
| 249 | GOBP\_NUCLEOBASE\_CONTAINING\_COMPOUND\_TRANSPORT |  | 176 | -0.33 | -1.57 | 0.001 | 0.169 | 1.000 | 2630 | tags=31%, list=20%, signal=39% |
| 250 | GOBP\_POSITIVE\_REGULATION\_OF\_HISTONE\_DEACETYLATION |  | 18 | -0.53 | -1.57 | 0.040 | 0.169 | 1.000 | 517 | tags=22%, list=4%, signal=23% |
| 251 | GOBP\_REGULATION\_OF\_ENDOPLASMIC\_RETICULUM\_UNFOLDED\_PROTEIN\_RESPONSE |  | 24 | -0.49 | -1.57 | 0.024 | 0.169 | 1.000 | 4222 | tags=63%, list=33%, signal=93% |
| 252 | GOBP\_NEGATIVE\_REGULATION\_OF\_CELLULAR\_MACROMOLECULE\_BIOSYNTHETIC\_PROCESS |  | 201 | -0.33 | -1.57 | 0.001 | 0.169 | 1.000 | 3064 | tags=35%, list=24%, signal=46% |
| 253 | GOBP\_NEUTRAL\_AMINO\_ACID\_TRANSPORT |  | 39 | -0.44 | -1.57 | 0.018 | 0.169 | 1.000 | 3363 | tags=51%, list=26%, signal=69% |
| 254 | GOBP\_PEPTIDYL\_SERINE\_MODIFICATION |  | 282 | -0.31 | -1.57 | 0.000 | 0.170 | 1.000 | 3802 | tags=38%, list=29%, signal=53% |
| 255 | GOBP\_ENDOTHELIAL\_CELL\_APOPTOTIC\_PROCESS |  | 52 | -0.41 | -1.57 | 0.008 | 0.171 | 1.000 | 3162 | tags=44%, list=25%, signal=58% |
| 256 | GOBP\_NUCLEAR\_TRANSCRIBED\_MRNA\_CATABOLIC\_PROCESS |  | 97 | -0.36 | -1.56 | 0.000 | 0.171 | 1.000 | 2712 | tags=38%, list=21%, signal=48% |
| 257 | GOBP\_PEPTIDYL\_TYROSINE\_DEPHOSPHORYLATION |  | 84 | -0.37 | -1.56 | 0.010 | 0.171 | 1.000 | 2151 | tags=29%, list=17%, signal=34% |
| 258 | GOBP\_MAMMARY\_GLAND\_EPITHELIAL\_CELL\_DIFFERENTIATION |  | 17 | -0.52 | -1.56 | 0.027 | 0.170 | 1.000 | 1142 | tags=29%, list=9%, signal=32% |
| 259 | GOBP\_VASCULAR\_ASSOCIATED\_SMOOTH\_MUSCLE\_CELL\_MIGRATION |  | 19 | -0.51 | -1.56 | 0.030 | 0.170 | 1.000 | 2126 | tags=32%, list=16%, signal=38% |
| 260 | GOBP\_CEREBRAL\_CORTEX\_RADIALLY\_ORIENTED\_CELL\_MIGRATION |  | 26 | -0.48 | -1.56 | 0.026 | 0.171 | 1.000 | 2554 | tags=35%, list=20%, signal=43% |
| 261 | GOBP\_NEGATIVE\_REGULATION\_OF\_HEMOPOIESIS |  | 88 | -0.37 | -1.56 | 0.005 | 0.172 | 1.000 | 2202 | tags=33%, list=17%, signal=39% |
| 262 | GOBP\_REGULATION\_OF\_CAMP\_MEDIATED\_SIGNALING |  | 23 | -0.49 | -1.56 | 0.035 | 0.173 | 1.000 | 2176 | tags=30%, list=17%, signal=37% |
| 263 | GOBP\_ENDOCRINE\_PROCESS |  | 79 | -0.37 | -1.56 | 0.003 | 0.172 | 1.000 | 4743 | tags=62%, list=37%, signal=97% |
| 264 | GOBP\_APOPTOTIC\_PROCESS\_INVOLVED\_IN\_DEVELOPMENT |  | 34 | -0.45 | -1.56 | 0.021 | 0.172 | 1.000 | 4706 | tags=59%, list=36%, signal=92% |
| 265 | GOBP\_POSITIVE\_REGULATION\_OF\_CELL\_CYCLE |  | 252 | -0.32 | -1.56 | 0.001 | 0.172 | 1.000 | 2392 | tags=25%, list=19%, signal=30% |
| 266 | GOBP\_POSITIVE\_REGULATION\_OF\_CELL\_MIGRATION\_INVOLVED\_IN\_SPROUTING\_ANGIOGENESIS |  | 20 | -0.50 | -1.56 | 0.033 | 0.172 | 1.000 | 3913 | tags=65%, list=30%, signal=93% |
| 267 | GOBP\_MATERNAL\_PROCESS\_INVOLVED\_IN\_FEMALE\_PREGNANCY |  | 49 | -0.41 | -1.56 | 0.008 | 0.173 | 1.000 | 4014 | tags=57%, list=31%, signal=83% |
| 268 | GOBP\_REGULATION\_OF\_MRNA\_CATABOLIC\_PROCESS |  | 134 | -0.34 | -1.56 | 0.008 | 0.174 | 1.000 | 2525 | tags=30%, list=20%, signal=37% |
| 269 | GOBP\_POSITIVE\_REGULATION\_OF\_SMOOTH\_MUSCLE\_CELL\_PROLIFERATION |  | 76 | -0.38 | -1.56 | 0.003 | 0.174 | 1.000 | 2124 | tags=26%, list=16%, signal=31% |
| 270 | GOBP\_MESENCHYMAL\_CELL\_DIFFERENTIATION |  | 192 | -0.32 | -1.56 | 0.001 | 0.174 | 1.000 | 1983 | tags=25%, list=15%, signal=29% |
| 271 | GOBP\_REGULATION\_OF\_MACROPHAGE\_DIFFERENTIATION |  | 19 | -0.51 | -1.56 | 0.016 | 0.174 | 1.000 | 2460 | tags=47%, list=19%, signal=58% |
| 272 | GOBP\_NEGATIVE\_REGULATION\_OF\_CELLULAR\_AMIDE\_METABOLIC\_PROCESS |  | 154 | -0.34 | -1.55 | 0.005 | 0.176 | 1.000 | 2899 | tags=34%, list=22%, signal=44% |
| 273 | GOBP\_ALTERNATIVE\_MRNA\_SPLICING\_VIA\_SPLICEOSOME |  | 62 | -0.39 | -1.55 | 0.016 | 0.177 | 1.000 | 2403 | tags=37%, list=19%, signal=45% |
| 274 | GOBP\_POSITIVE\_REGULATION\_OF\_FAT\_CELL\_DIFFERENTIATION |  | 50 | -0.40 | -1.55 | 0.010 | 0.180 | 1.000 | 2246 | tags=30%, list=17%, signal=36% |
| 275 | GOBP\_REGULATION\_OF\_HIPPO\_SIGNALING |  | 17 | -0.53 | -1.55 | 0.027 | 0.180 | 1.000 | 3583 | tags=53%, list=28%, signal=73% |
| 276 | GOBP\_NEGATIVE\_REGULATION\_OF\_NECROTIC\_CELL\_DEATH |  | 15 | -0.54 | -1.55 | 0.048 | 0.180 | 1.000 | 2460 | tags=40%, list=19%, signal=49% |
| 277 | GOBP\_EPITHELIAL\_CELL\_DEVELOPMENT |  | 182 | -0.33 | -1.55 | 0.001 | 0.180 | 1.000 | 3162 | tags=33%, list=25%, signal=43% |
| 278 | GOBP\_EPIDERMIS\_MORPHOGENESIS |  | 24 | -0.48 | -1.55 | 0.028 | 0.180 | 1.000 | 537 | tags=17%, list=4%, signal=17% |
| 279 | GOBP\_VASCULAR\_PROCESS\_IN\_CIRCULATORY\_SYSTEM |  | 238 | -0.31 | -1.55 | 0.000 | 0.179 | 1.000 | 4455 | tags=49%, list=35%, signal=73% |
| 280 | GOBP\_REGULATION\_OF\_STEROID\_HORMONE\_SECRETION |  | 17 | -0.53 | -1.55 | 0.027 | 0.181 | 1.000 | 2881 | tags=47%, list=22%, signal=61% |
| 281 | GOBP\_RHYTHMIC\_PROCESS |  | 266 | -0.31 | -1.54 | 0.000 | 0.183 | 1.000 | 2605 | tags=29%, list=20%, signal=35% |
| 282 | GOBP\_REGULATION\_OF\_HEMATOPOIETIC\_PROGENITOR\_CELL\_DIFFERENTIATION |  | 27 | -0.47 | -1.54 | 0.028 | 0.183 | 1.000 | 1631 | tags=37%, list=13%, signal=42% |
| 283 | GOBP\_NUCLEUS\_ORGANIZATION |  | 119 | -0.34 | -1.54 | 0.000 | 0.183 | 1.000 | 2779 | tags=35%, list=22%, signal=45% |
| 284 | GOBP\_POSITIVE\_REGULATION\_OF\_TRANSCRIPTION\_FROM\_RNA\_POLYMERASE\_II\_PROMOTER\_INVOLVED\_IN\_CELLULAR\_RESPONSE\_TO\_CHEMICAL\_STIMULUS |  | 20 | -0.51 | -1.54 | 0.036 | 0.183 | 1.000 | 1269 | tags=35%, list=10%, signal=39% |
| 285 | GOBP\_CELL\_DIFFERENTIATION\_INVOLVED\_IN\_KIDNEY\_DEVELOPMENT |  | 54 | -0.40 | -1.54 | 0.024 | 0.182 | 1.000 | 2523 | tags=30%, list=20%, signal=37% |
| 286 | GOBP\_EMBRYONIC\_HINDLIMB\_MORPHOGENESIS |  | 26 | -0.47 | -1.54 | 0.026 | 0.182 | 1.000 | 2944 | tags=46%, list=23%, signal=60% |
| 287 | GOBP\_SMOOTH\_MUSCLE\_CELL\_APOPTOTIC\_PROCESS |  | 21 | -0.50 | -1.54 | 0.030 | 0.185 | 1.000 | 1626 | tags=29%, list=13%, signal=33% |
| 288 | GOBP\_AORTA\_MORPHOGENESIS |  | 31 | -0.44 | -1.54 | 0.031 | 0.188 | 1.000 | 2604 | tags=39%, list=20%, signal=48% |
| 289 | GOBP\_CELLULAR\_RESPONSE\_TO\_VASCULAR\_ENDOTHELIAL\_GROWTH\_FACTOR\_STIMULUS |  | 56 | -0.39 | -1.54 | 0.011 | 0.188 | 1.000 | 2078 | tags=29%, list=16%, signal=34% |
| 290 | GOBP\_REGULATION\_OF\_RESPONSE\_TO\_WOUNDING |  | 138 | -0.34 | -1.54 | 0.005 | 0.188 | 1.000 | 3040 | tags=34%, list=24%, signal=44% |
| 291 | GOBP\_RESPONSE\_TO\_HYDROGEN\_PEROXIDE |  | 112 | -0.34 | -1.54 | 0.006 | 0.188 | 1.000 | 2078 | tags=25%, list=16%, signal=30% |
| 292 | GOBP\_RNA\_CATABOLIC\_PROCESS |  | 217 | -0.31 | -1.54 | 0.001 | 0.188 | 1.000 | 3279 | tags=35%, list=25%, signal=46% |
| 293 | GOBP\_RESPONSE\_TO\_TUMOR\_NECROSIS\_FACTOR |  | 206 | -0.32 | -1.53 | 0.001 | 0.189 | 1.000 | 2511 | tags=29%, list=19%, signal=36% |
| 294 | GOBP\_RAS\_PROTEIN\_SIGNAL\_TRANSDUCTION |  | 285 | -0.30 | -1.53 | 0.000 | 0.192 | 1.000 | 2442 | tags=26%, list=19%, signal=32% |
| 295 | GOBP\_INTERLEUKIN\_1\_MEDIATED\_SIGNALING\_PATHWAY |  | 26 | -0.46 | -1.53 | 0.020 | 0.191 | 1.000 | 2738 | tags=42%, list=21%, signal=54% |
| 296 | GOBP\_REGULATION\_OF\_NOTCH\_SIGNALING\_PATHWAY |  | 72 | -0.37 | -1.53 | 0.013 | 0.191 | 1.000 | 3108 | tags=38%, list=24%, signal=49% |
| 297 | GOBP\_POSITIVE\_REGULATION\_OF\_NITRIC\_OXIDE\_METABOLIC\_PROCESS |  | 37 | -0.42 | -1.53 | 0.020 | 0.191 | 1.000 | 1961 | tags=30%, list=15%, signal=35% |
| 298 | GOBP\_POSITIVE\_REGULATION\_OF\_BLOOD\_VESSEL\_ENDOTHELIAL\_CELL\_MIGRATION |  | 52 | -0.40 | -1.53 | 0.026 | 0.191 | 1.000 | 2180 | tags=35%, list=17%, signal=41% |
| 299 | GOBP\_POSITIVE\_REGULATION\_OF\_WOUND\_HEALING |  | 49 | -0.40 | -1.53 | 0.020 | 0.194 | 1.000 | 2996 | tags=39%, list=23%, signal=50% |
| 300 | GOBP\_POSITIVE\_CHEMOTAXIS |  | 55 | -0.39 | -1.53 | 0.021 | 0.196 | 1.000 | 3548 | tags=45%, list=28%, signal=62% |
| 301 | GOBP\_POSITIVE\_REGULATION\_OF\_MYELINATION |  | 15 | -0.54 | -1.53 | 0.036 | 0.196 | 1.000 | 2586 | tags=40%, list=20%, signal=50% |
| 302 | GOBP\_VASCULAR\_ASSOCIATED\_SMOOTH\_MUSCLE\_CELL\_PROLIFERATION |  | 51 | -0.40 | -1.53 | 0.025 | 0.196 | 1.000 | 2126 | tags=27%, list=16%, signal=33% |
| 303 | GOBP\_POSITIVE\_REGULATION\_OF\_CELL\_CYCLE\_PROCESS |  | 184 | -0.32 | -1.53 | 0.000 | 0.196 | 1.000 | 1756 | tags=21%, list=14%, signal=24% |
| 304 | GOBP\_FAT\_CELL\_DIFFERENTIATION |  | 191 | -0.32 | -1.52 | 0.002 | 0.196 | 1.000 | 2714 | tags=31%, list=21%, signal=39% |
| 305 | GOBP\_REGULATION\_OF\_TRANSCRIPTION\_OF\_NUCLEOLAR\_LARGE\_RRNA\_BY\_RNA\_POLYMERASE\_I |  | 15 | -0.55 | -1.52 | 0.054 | 0.196 | 1.000 | 4178 | tags=60%, list=32%, signal=89% |
| 306 | GOBP\_POSITIVE\_REGULATION\_OF\_EPITHELIAL\_TO\_MESENCHYMAL\_TRANSITION |  | 43 | -0.41 | -1.52 | 0.029 | 0.203 | 1.000 | 1983 | tags=26%, list=15%, signal=30% |
| 307 | GOBP\_RESPONSE\_TO\_ARSENIC\_CONTAINING\_SUBSTANCE |  | 29 | -0.45 | -1.52 | 0.035 | 0.202 | 1.000 | 1713 | tags=38%, list=13%, signal=44% |
| 308 | GOBP\_MACROMOLECULE\_DEACYLATION |  | 100 | -0.35 | -1.52 | 0.011 | 0.202 | 1.000 | 4243 | tags=46%, list=33%, signal=68% |
| 309 | GOBP\_POSITIVE\_REGULATION\_OF\_LAMELLIPODIUM\_ASSEMBLY |  | 23 | -0.47 | -1.52 | 0.028 | 0.202 | 1.000 | 2196 | tags=39%, list=17%, signal=47% |
| 310 | GOBP\_POSITIVE\_REGULATION\_OF\_LIPID\_CATABOLIC\_PROCESS |  | 23 | -0.47 | -1.52 | 0.045 | 0.201 | 1.000 | 3032 | tags=43%, list=24%, signal=57% |
| 311 | GOBP\_NEGATIVE\_REGULATION\_OF\_ALPHA\_BETA\_T\_CELL\_DIFFERENTIATION |  | 23 | -0.48 | -1.52 | 0.043 | 0.201 | 1.000 | 4200 | tags=57%, list=33%, signal=84% |
| 312 | GOBP\_SPLICEOSOMAL\_COMPLEX\_ASSEMBLY |  | 50 | -0.39 | -1.52 | 0.024 | 0.201 | 1.000 | 2994 | tags=52%, list=23%, signal=67% |
| 313 | GOBP\_REGULATION\_OF\_INTRINSIC\_APOPTOTIC\_SIGNALING\_PATHWAY\_IN\_RESPONSE\_TO\_DNA\_DAMAGE |  | 32 | -0.43 | -1.52 | 0.028 | 0.200 | 1.000 | 1657 | tags=28%, list=13%, signal=32% |
| 314 | GOBP\_PHOSPHATIDYLINOSITOL\_DEPHOSPHORYLATION |  | 29 | -0.46 | -1.52 | 0.026 | 0.200 | 1.000 | 3443 | tags=45%, list=27%, signal=61% |
| 315 | GOBP\_CELLULAR\_RESPONSE\_TO\_ATP |  | 16 | -0.52 | -1.52 | 0.049 | 0.200 | 1.000 | 3768 | tags=56%, list=29%, signal=79% |
| 316 | GOBP\_NEGATIVE\_REGULATION\_OF\_MUSCLE\_HYPERTROPHY |  | 24 | -0.46 | -1.52 | 0.035 | 0.199 | 1.000 | 1956 | tags=38%, list=15%, signal=44% |
| 317 | GOBP\_CELLULAR\_RESPONSE\_TO\_HORMONE\_STIMULUS |  | 500 | -0.29 | -1.52 | 0.000 | 0.199 | 1.000 | 3193 | tags=32%, list=25%, signal=41% |
| 318 | GOBP\_T\_HELPER\_1\_TYPE\_IMMUNE\_RESPONSE |  | 39 | -0.42 | -1.52 | 0.026 | 0.200 | 1.000 | 2080 | tags=31%, list=16%, signal=37% |
| 319 | GOBP\_REGULATION\_OF\_MYOTUBE\_DIFFERENTIATION |  | 31 | -0.45 | -1.52 | 0.035 | 0.200 | 1.000 | 1880 | tags=32%, list=15%, signal=38% |
| 320 | GOBP\_RNA\_SPLICING\_VIA\_TRANSESTERIFICATION\_REACTIONS |  | 243 | -0.31 | -1.51 | 0.000 | 0.202 | 1.000 | 2692 | tags=32%, list=21%, signal=40% |
| 321 | GOBP\_REGULATION\_OF\_PROGRAMMED\_NECROTIC\_CELL\_DEATH |  | 21 | -0.48 | -1.51 | 0.044 | 0.202 | 1.000 | 2738 | tags=38%, list=21%, signal=48% |
| 322 | GOBP\_MESENCHYME\_DEVELOPMENT |  | 234 | -0.31 | -1.51 | 0.001 | 0.202 | 1.000 | 1983 | tags=23%, list=15%, signal=26% |
| 323 | GOBP\_ENDOTHELIAL\_CELL\_MIGRATION |  | 200 | -0.32 | -1.51 | 0.000 | 0.204 | 1.000 | 3107 | tags=36%, list=24%, signal=46% |
| 324 | GOBP\_CELL\_CELL\_JUNCTION\_ASSEMBLY |  | 122 | -0.34 | -1.51 | 0.013 | 0.204 | 1.000 | 3205 | tags=30%, list=25%, signal=40% |
| 325 | GOBP\_PEPTIDYL\_LYSINE\_DIMETHYLATION |  | 21 | -0.50 | -1.51 | 0.041 | 0.204 | 1.000 | 2104 | tags=38%, list=16%, signal=45% |
| 326 | GOBP\_REGULATION\_OF\_CARBOHYDRATE\_BIOSYNTHETIC\_PROCESS |  | 82 | -0.36 | -1.51 | 0.014 | 0.203 | 1.000 | 1922 | tags=28%, list=15%, signal=33% |
| 327 | GOBP\_CELLULAR\_SENESCENCE |  | 70 | -0.37 | -1.51 | 0.018 | 0.203 | 1.000 | 4925 | tags=57%, list=38%, signal=92% |
| 328 | GOBP\_REGULATION\_OF\_MRNA\_3\_END\_PROCESSING |  | 24 | -0.47 | -1.51 | 0.041 | 0.204 | 1.000 | 2215 | tags=33%, list=17%, signal=40% |
| 329 | GOBP\_EPITHELIAL\_CELL\_DIFFERENTIATION\_INVOLVED\_IN\_KIDNEY\_DEVELOPMENT |  | 46 | -0.40 | -1.51 | 0.027 | 0.205 | 1.000 | 2791 | tags=33%, list=22%, signal=41% |
| 330 | GOBP\_MRNA\_MODIFICATION |  | 19 | -0.49 | -1.51 | 0.038 | 0.206 | 1.000 | 2311 | tags=37%, list=18%, signal=45% |
| 331 | GOBP\_KILLING\_OF\_CELLS\_OF\_ANOTHER\_ORGANISM |  | 30 | -0.45 | -1.51 | 0.030 | 0.208 | 1.000 | 3815 | tags=47%, list=30%, signal=66% |
| 332 | GOBP\_NEUROPEPTIDE\_SIGNALING\_PATHWAY |  | 78 | -0.37 | -1.50 | 0.018 | 0.210 | 1.000 | 2593 | tags=29%, list=20%, signal=37% |
| 333 | GOBP\_NEGATIVE\_REGULATION\_OF\_SYSTEMIC\_ARTERIAL\_BLOOD\_PRESSURE |  | 18 | -0.50 | -1.50 | 0.050 | 0.211 | 1.000 | 1728 | tags=39%, list=13%, signal=45% |
| 334 | GOBP\_REGENERATION |  | 161 | -0.32 | -1.50 | 0.005 | 0.212 | 1.000 | 2705 | tags=32%, list=21%, signal=40% |
| 335 | GOBP\_NEGATIVE\_REGULATION\_OF\_VASCULAR\_ASSOCIATED\_SMOOTH\_MUSCLE\_CELL\_PROLIFERATION |  | 20 | -0.48 | -1.50 | 0.045 | 0.211 | 1.000 | 1066 | tags=25%, list=8%, signal=27% |
| 336 | GOBP\_HINDBRAIN\_MORPHOGENESIS |  | 37 | -0.42 | -1.50 | 0.032 | 0.211 | 1.000 | 2791 | tags=41%, list=22%, signal=52% |
| 337 | GOBP\_RESPONSE\_TO\_CAMP |  | 83 | -0.36 | -1.50 | 0.020 | 0.213 | 1.000 | 2262 | tags=28%, list=18%, signal=33% |
| 338 | GOBP\_POSITIVE\_REGULATION\_OF\_NERVOUS\_SYSTEM\_PROCESS |  | 30 | -0.44 | -1.50 | 0.033 | 0.213 | 1.000 | 2593 | tags=33%, list=20%, signal=42% |
| 339 | GOBP\_POSITIVE\_REGULATION\_OF\_CELLULAR\_AMIDE\_METABOLIC\_PROCESS |  | 134 | -0.33 | -1.50 | 0.003 | 0.213 | 1.000 | 2447 | tags=30%, list=19%, signal=36% |
| 340 | GOBP\_NUCLEAR\_MIGRATION |  | 17 | -0.51 | -1.50 | 0.049 | 0.213 | 1.000 | 2554 | tags=41%, list=20%, signal=51% |
| 341 | GOBP\_NEGATIVE\_REGULATION\_OF\_CELLULAR\_CATABOLIC\_PROCESS |  | 211 | -0.31 | -1.50 | 0.000 | 0.214 | 1.000 | 2932 | tags=33%, list=23%, signal=42% |
| 342 | GOBP\_NEGATIVE\_REGULATION\_OF\_MYELOID\_CELL\_DIFFERENTIATION |  | 77 | -0.36 | -1.50 | 0.015 | 0.213 | 1.000 | 1844 | tags=26%, list=14%, signal=30% |
| 343 | GOBP\_REGULATION\_OF\_MONOCYTE\_DIFFERENTIATION |  | 16 | -0.52 | -1.50 | 0.048 | 0.213 | 1.000 | 843 | tags=19%, list=7%, signal=20% |
| 344 | GOBP\_REGULATION\_OF\_LAMELLIPODIUM\_ASSEMBLY |  | 33 | -0.43 | -1.50 | 0.035 | 0.213 | 1.000 | 2916 | tags=39%, list=23%, signal=51% |
| 345 | GOBP\_REGULATION\_OF\_MUSCLE\_CELL\_DIFFERENTIATION |  | 104 | -0.34 | -1.50 | 0.012 | 0.212 | 1.000 | 2124 | tags=29%, list=16%, signal=34% |
| 346 | GOBP\_POSITIVE\_REGULATION\_OF\_MUSCLE\_HYPERTROPHY |  | 24 | -0.47 | -1.50 | 0.053 | 0.213 | 1.000 | 2461 | tags=42%, list=19%, signal=51% |
| 347 | GOBP\_PROTEIN\_KINASE\_C\_ACTIVATING\_G\_PROTEIN\_COUPLED\_RECEPTOR\_SIGNALING\_PATHWAY |  | 25 | -0.46 | -1.50 | 0.035 | 0.213 | 1.000 | 2593 | tags=44%, list=20%, signal=55% |
| 348 | GOBP\_PROTEIN\_K63\_LINKED\_DEUBIQUITINATION |  | 27 | -0.45 | -1.50 | 0.043 | 0.213 | 1.000 | 1847 | tags=30%, list=14%, signal=35% |
| 349 | GOBP\_EXCRETION |  | 59 | -0.38 | -1.50 | 0.016 | 0.213 | 1.000 | 3957 | tags=44%, list=31%, signal=63% |
| 350 | GOBP\_LENS\_DEVELOPMENT\_IN\_CAMERA\_TYPE\_EYE |  | 74 | -0.36 | -1.50 | 0.020 | 0.213 | 1.000 | 903 | tags=16%, list=7%, signal=17% |
| 351 | GOBP\_NEGATIVE\_REGULATION\_OF\_CATABOLIC\_PROCESS |  | 260 | -0.30 | -1.50 | 0.004 | 0.213 | 1.000 | 2932 | tags=32%, list=23%, signal=41% |
| 352 | GOBP\_CELL\_DIFFERENTIATION\_INVOLVED\_IN\_EMBRYONIC\_PLACENTA\_DEVELOPMENT |  | 23 | -0.47 | -1.49 | 0.036 | 0.213 | 1.000 | 2979 | tags=52%, list=23%, signal=68% |
| 353 | GOBP\_APOPTOTIC\_DNA\_FRAGMENTATION |  | 16 | -0.52 | -1.49 | 0.049 | 0.212 | 1.000 | 2636 | tags=44%, list=20%, signal=55% |
| 354 | GOBP\_RESPONSE\_TO\_ELECTRICAL\_STIMULUS |  | 38 | -0.41 | -1.49 | 0.030 | 0.213 | 1.000 | 1353 | tags=26%, list=10%, signal=29% |
| 355 | GOBP\_ENTRAINMENT\_OF\_CIRCADIAN\_CLOCK |  | 28 | -0.45 | -1.49 | 0.027 | 0.217 | 1.000 | 1165 | tags=32%, list=9%, signal=35% |
| 356 | GOBP\_MRNA\_TRANSCRIPTION |  | 38 | -0.41 | -1.49 | 0.035 | 0.217 | 1.000 | 2124 | tags=29%, list=16%, signal=35% |
| 357 | GOBP\_VASCULAR\_WOUND\_HEALING |  | 15 | -0.53 | -1.49 | 0.051 | 0.217 | 1.000 | 2618 | tags=47%, list=20%, signal=58% |
| 358 | GOBP\_PLASMA\_MEMBRANE\_TUBULATION |  | 16 | -0.52 | -1.49 | 0.051 | 0.217 | 1.000 | 2820 | tags=56%, list=22%, signal=72% |
| 359 | GOBP\_POSITIVE\_REGULATION\_OF\_FIBROBLAST\_PROLIFERATION |  | 46 | -0.40 | -1.49 | 0.026 | 0.217 | 1.000 | 798 | tags=24%, list=6%, signal=25% |
| 360 | GOBP\_DEMETHYLATION |  | 60 | -0.37 | -1.49 | 0.028 | 0.216 | 1.000 | 2940 | tags=38%, list=23%, signal=49% |
| 361 | GOBP\_GAS\_TRANSPORT |  | 17 | -0.50 | -1.49 | 0.049 | 0.217 | 1.000 | 2429 | tags=35%, list=19%, signal=43% |
| 362 | GOBP\_CELL\_FATE\_COMMITMENT |  | 209 | -0.31 | -1.49 | 0.007 | 0.218 | 1.000 | 3046 | tags=31%, list=24%, signal=39% |
| 363 | GOBP\_REGULATION\_OF\_CLATHRIN\_DEPENDENT\_ENDOCYTOSIS |  | 17 | -0.50 | -1.49 | 0.051 | 0.219 | 1.000 | 1185 | tags=35%, list=9%, signal=39% |
| 364 | GOBP\_RESPONSE\_TO\_INSULIN |  | 217 | -0.31 | -1.49 | 0.001 | 0.219 | 1.000 | 2804 | tags=31%, list=22%, signal=39% |
| 365 | GOBP\_NEGATIVE\_REGULATION\_OF\_CELL\_DEVELOPMENT |  | 150 | -0.32 | -1.49 | 0.004 | 0.220 | 1.000 | 1966 | tags=25%, list=15%, signal=30% |
| 366 | GOBP\_ESTABLISHMENT\_OR\_MAINTENANCE\_OF\_MONOPOLAR\_CELL\_POLARITY |  | 21 | -0.48 | -1.49 | 0.041 | 0.220 | 1.000 | 4441 | tags=67%, list=34%, signal=101% |
| 367 | GOBP\_HIPPO\_SIGNALING |  | 32 | -0.43 | -1.49 | 0.043 | 0.220 | 1.000 | 2828 | tags=38%, list=22%, signal=48% |
| 368 | GOBP\_NEGATIVE\_REGULATION\_OF\_T\_CELL\_DIFFERENTIATION |  | 39 | -0.42 | -1.48 | 0.030 | 0.219 | 1.000 | 2202 | tags=33%, list=17%, signal=40% |
| 369 | GOBP\_POSITIVE\_REGULATION\_OF\_PHOSPHOLIPASE\_ACTIVITY |  | 52 | -0.38 | -1.48 | 0.033 | 0.219 | 1.000 | 2076 | tags=27%, list=16%, signal=32% |
| 370 | GOBP\_RENAL\_SODIUM\_EXCRETION |  | 21 | -0.46 | -1.48 | 0.055 | 0.222 | 1.000 | 1626 | tags=29%, list=13%, signal=33% |
| 371 | GOBP\_HEPATOCYTE\_APOPTOTIC\_PROCESS |  | 16 | -0.51 | -1.48 | 0.057 | 0.223 | 1.000 | 2246 | tags=44%, list=17%, signal=53% |
| 372 | GOBP\_VASCULAR\_ENDOTHELIAL\_GROWTH\_FACTOR\_PRODUCTION |  | 32 | -0.42 | -1.48 | 0.033 | 0.224 | 1.000 | 1287 | tags=31%, list=10%, signal=35% |
| 373 | GOBP\_CEREBRAL\_CORTEX\_CELL\_MIGRATION |  | 36 | -0.42 | -1.48 | 0.032 | 0.223 | 1.000 | 2554 | tags=33%, list=20%, signal=41% |
| 374 | GOBP\_REGULATION\_OF\_PHOSPHOLIPASE\_ACTIVITY |  | 63 | -0.37 | -1.48 | 0.018 | 0.223 | 1.000 | 2078 | tags=27%, list=16%, signal=32% |
| 375 | GOBP\_T\_HELPER\_1\_CELL\_DIFFERENTIATION |  | 18 | -0.50 | -1.48 | 0.063 | 0.225 | 1.000 | 3352 | tags=50%, list=26%, signal=67% |
| 376 | GOBP\_PEPTIDYL\_LYSINE\_ACETYLATION |  | 159 | -0.32 | -1.48 | 0.006 | 0.225 | 1.000 | 2095 | tags=27%, list=16%, signal=32% |
| 377 | GOBP\_POSITIVE\_REGULATION\_OF\_TRANSLATION |  | 112 | -0.33 | -1.48 | 0.017 | 0.225 | 1.000 | 3652 | tags=42%, list=28%, signal=58% |
| 378 | GOBP\_WOUND\_HEALING\_SPREADING\_OF\_EPIDERMAL\_CELLS |  | 16 | -0.51 | -1.48 | 0.079 | 0.225 | 1.000 | 1066 | tags=38%, list=8%, signal=41% |
| 379 | GOBP\_DIGESTIVE\_SYSTEM\_DEVELOPMENT |  | 121 | -0.33 | -1.48 | 0.016 | 0.227 | 1.000 | 2060 | tags=24%, list=16%, signal=28% |
| 380 | GOBP\_NEGATIVE\_REGULATION\_OF\_CARDIAC\_MUSCLE\_TISSUE\_GROWTH |  | 20 | -0.48 | -1.48 | 0.055 | 0.227 | 1.000 | 1792 | tags=40%, list=14%, signal=46% |
| 381 | GOBP\_AXON\_EXTENSION |  | 105 | -0.34 | -1.47 | 0.006 | 0.228 | 1.000 | 1934 | tags=26%, list=15%, signal=30% |
| 382 | GOBP\_BRANCHING\_MORPHOGENESIS\_OF\_AN\_EPITHELIAL\_TUBE |  | 132 | -0.33 | -1.47 | 0.006 | 0.228 | 1.000 | 3961 | tags=42%, list=31%, signal=60% |
| 383 | GOBP\_REGULATION\_OF\_EXTENT\_OF\_CELL\_GROWTH |  | 94 | -0.35 | -1.47 | 0.021 | 0.228 | 1.000 | 1917 | tags=26%, list=15%, signal=30% |
| 384 | GOBP\_POSITIVE\_REGULATION\_OF\_LOCOMOTION |  | 490 | -0.28 | -1.47 | 0.000 | 0.231 | 1.000 | 3001 | tags=30%, list=23%, signal=37% |
| 385 | GOBP\_POSITIVE\_REGULATION\_OF\_DNA\_REPLICATION |  | 36 | -0.41 | -1.47 | 0.045 | 0.230 | 1.000 | 2275 | tags=39%, list=18%, signal=47% |
| 386 | GOBP\_REGULATION\_OF\_GLUCOSE\_TRANSMEMBRANE\_TRANSPORT |  | 65 | -0.37 | -1.47 | 0.030 | 0.230 | 1.000 | 3267 | tags=40%, list=25%, signal=53% |
| 387 | GOBP\_CD4\_POSITIVE\_ALPHA\_BETA\_T\_CELL\_CYTOKINE\_PRODUCTION |  | 19 | -0.47 | -1.47 | 0.061 | 0.231 | 1.000 | 2101 | tags=37%, list=16%, signal=44% |
| 388 | GOBP\_REGULATION\_OF\_PROTEIN\_EXPORT\_FROM\_NUCLEUS |  | 28 | -0.43 | -1.47 | 0.059 | 0.230 | 1.000 | 2614 | tags=43%, list=20%, signal=54% |
| 389 | GOBP\_REGULATION\_OF\_SISTER\_CHROMATID\_COHESION |  | 16 | -0.51 | -1.47 | 0.060 | 0.230 | 1.000 | 295 | tags=19%, list=2%, signal=19% |
| 390 | GOBP\_POSITIVE\_REGULATION\_OF\_OSTEOBLAST\_DIFFERENTIATION |  | 58 | -0.37 | -1.47 | 0.024 | 0.231 | 1.000 | 1983 | tags=26%, list=15%, signal=30% |
| 391 | GOBP\_ACUTE\_PHASE\_RESPONSE |  | 44 | -0.40 | -1.47 | 0.041 | 0.231 | 1.000 | 3266 | tags=43%, list=25%, signal=58% |
| 392 | GOBP\_DEVELOPMENT\_OF\_PRIMARY\_SEXUAL\_CHARACTERISTICS |  | 199 | -0.31 | -1.47 | 0.001 | 0.231 | 1.000 | 2959 | tags=27%, list=23%, signal=35% |
| 393 | GOBP\_CALCIUM\_INDEPENDENT\_CELL\_CELL\_ADHESION\_VIA\_PLASMA\_MEMBRANE\_CELL\_ADHESION\_MOLECULES |  | 17 | -0.50 | -1.47 | 0.060 | 0.231 | 1.000 | 4085 | tags=59%, list=32%, signal=86% |
| 394 | GOBP\_AUTONOMIC\_NERVOUS\_SYSTEM\_DEVELOPMENT |  | 41 | -0.40 | -1.47 | 0.045 | 0.232 | 1.000 | 3723 | tags=44%, list=29%, signal=62% |
| 395 | GOBP\_NEGATIVE\_REGULATION\_OF\_MYELOID\_LEUKOCYTE\_DIFFERENTIATION |  | 42 | -0.40 | -1.47 | 0.044 | 0.234 | 1.000 | 1844 | tags=31%, list=14%, signal=36% |
| 396 | GOBP\_STRESS\_GRANULE\_ASSEMBLY |  | 26 | -0.45 | -1.47 | 0.051 | 0.234 | 1.000 | 2343 | tags=38%, list=18%, signal=47% |
| 397 | GOBP\_REGULATION\_OF\_HORMONE\_BIOSYNTHETIC\_PROCESS |  | 22 | -0.47 | -1.46 | 0.055 | 0.235 | 1.000 | 4620 | tags=73%, list=36%, signal=113% |
| 398 | GOBP\_AORTIC\_VALVE\_MORPHOGENESIS |  | 28 | -0.43 | -1.46 | 0.065 | 0.234 | 1.000 | 3354 | tags=46%, list=26%, signal=63% |
| 399 | GOBP\_CELLULAR\_RESPONSE\_TO\_STEROID\_HORMONE\_STIMULUS |  | 179 | -0.31 | -1.46 | 0.009 | 0.234 | 1.000 | 2119 | tags=25%, list=16%, signal=30% |
| 400 | GOBP\_RESPONSE\_TO\_PEPTIDE\_HORMONE |  | 348 | -0.29 | -1.46 | 0.000 | 0.234 | 1.000 | 2804 | tags=29%, list=22%, signal=36% |
| 401 | GOBP\_PEPTIDYL\_THREONINE\_MODIFICATION |  | 101 | -0.33 | -1.46 | 0.008 | 0.234 | 1.000 | 2766 | tags=32%, list=21%, signal=40% |
| 402 | GOBP\_ARTERY\_MORPHOGENESIS |  | 65 | -0.36 | -1.46 | 0.028 | 0.236 | 1.000 | 2604 | tags=32%, list=20%, signal=40% |
| 403 | GOBP\_NEPHRON\_DEVELOPMENT |  | 125 | -0.32 | -1.46 | 0.016 | 0.238 | 1.000 | 2598 | tags=27%, list=20%, signal=34% |
| 404 | GOBP\_REGULATION\_OF\_VASOCONSTRICTION |  | 59 | -0.37 | -1.46 | 0.037 | 0.238 | 1.000 | 4933 | tags=53%, list=38%, signal=85% |
| 405 | GOBP\_REGULATION\_OF\_DEVELOPMENTAL\_GROWTH |  | 273 | -0.29 | -1.46 | 0.003 | 0.240 | 1.000 | 1947 | tags=22%, list=15%, signal=25% |
| 406 | GOBP\_REGULATION\_OF\_STRIATED\_MUSCLE\_CELL\_DIFFERENTIATION |  | 67 | -0.36 | -1.46 | 0.027 | 0.241 | 1.000 | 1983 | tags=30%, list=15%, signal=35% |
| 407 | GOBP\_MUSCLE\_TISSUE\_DEVELOPMENT |  | 327 | -0.29 | -1.46 | 0.000 | 0.242 | 1.000 | 3178 | tags=31%, list=25%, signal=40% |
| 408 | GOBP\_REGULATION\_OF\_STEM\_CELL\_DIFFERENTIATION |  | 46 | -0.38 | -1.46 | 0.035 | 0.242 | 1.000 | 3977 | tags=50%, list=31%, signal=72% |
| 409 | GOBP\_MULTI\_MULTICELLULAR\_ORGANISM\_PROCESS |  | 190 | -0.31 | -1.46 | 0.000 | 0.241 | 1.000 | 2773 | tags=31%, list=21%, signal=39% |
| 410 | GOBP\_REGULATION\_OF\_RESPONSE\_TO\_DNA\_DAMAGE\_STIMULUS |  | 200 | -0.30 | -1.46 | 0.011 | 0.241 | 1.000 | 1713 | tags=22%, list=13%, signal=25% |
| 411 | GOBP\_HEART\_TRABECULA\_MORPHOGENESIS |  | 29 | -0.43 | -1.46 | 0.048 | 0.241 | 1.000 | 4414 | tags=52%, list=34%, signal=78% |
| 412 | GOBP\_REGULATION\_OF\_FAT\_CELL\_DIFFERENTIATION |  | 107 | -0.33 | -1.45 | 0.017 | 0.245 | 1.000 | 2656 | tags=29%, list=21%, signal=36% |
| 413 | GOBP\_DNA\_GEOMETRIC\_CHANGE |  | 78 | -0.35 | -1.45 | 0.032 | 0.246 | 1.000 | 3026 | tags=41%, list=23%, signal=53% |
| 414 | GOBP\_REGULATION\_OF\_CYCLASE\_ACTIVITY |  | 47 | -0.39 | -1.45 | 0.032 | 0.245 | 1.000 | 3973 | tags=53%, list=31%, signal=77% |
| 415 | GOBP\_CELL\_PROLIFERATION\_IN\_HINDBRAIN |  | 15 | -0.51 | -1.45 | 0.060 | 0.247 | 1.000 | 1024 | tags=27%, list=8%, signal=29% |
| 416 | GOBP\_ESTABLISHMENT\_OR\_MAINTENANCE\_OF\_CELL\_POLARITY |  | 189 | -0.30 | -1.45 | 0.007 | 0.248 | 1.000 | 4560 | tags=47%, list=35%, signal=72% |
| 417 | GOBP\_FOREBRAIN\_DEVELOPMENT |  | 305 | -0.29 | -1.45 | 0.001 | 0.250 | 1.000 | 2196 | tags=22%, list=17%, signal=26% |
| 418 | GOBP\_MATURATION\_OF\_SSU\_RRNA\_FROM\_TRICISTRONIC\_RRNA\_TRANSCRIPT\_SSU\_RRNA\_5\_8S\_RRNA\_LSU\_RRNA |  | 29 | -0.43 | -1.45 | 0.043 | 0.250 | 1.000 | 2377 | tags=38%, list=18%, signal=46% |
| 419 | GOBP\_REGULATION\_OF\_LAMELLIPODIUM\_ORGANIZATION |  | 45 | -0.38 | -1.45 | 0.030 | 0.253 | 1.000 | 2916 | tags=36%, list=23%, signal=46% |
| 420 | GOBP\_DECIDUALIZATION |  | 20 | -0.48 | -1.45 | 0.058 | 0.252 | 1.000 | 1601 | tags=35%, list=12%, signal=40% |
| 421 | GOBP\_CHROMATIN\_ORGANIZATION |  | 463 | -0.28 | -1.45 | 0.004 | 0.253 | 1.000 | 1426 | tags=18%, list=11%, signal=20% |
| 422 | GOBP\_POSITIVE\_REGULATION\_OF\_CELL\_GROWTH |  | 135 | -0.32 | -1.45 | 0.014 | 0.252 | 1.000 | 1728 | tags=24%, list=13%, signal=27% |
| 423 | GOBP\_REGULATION\_OF\_ENDOTHELIAL\_CELL\_MIGRATION |  | 156 | -0.31 | -1.45 | 0.012 | 0.252 | 1.000 | 2996 | tags=33%, list=23%, signal=43% |
| 424 | GOBP\_DEVELOPMENTAL\_CELL\_GROWTH |  | 189 | -0.30 | -1.45 | 0.006 | 0.252 | 1.000 | 1934 | tags=23%, list=15%, signal=26% |
| 425 | GOBP\_MESENCHYMAL\_TO\_EPITHELIAL\_TRANSITION |  | 19 | -0.47 | -1.45 | 0.068 | 0.253 | 1.000 | 817 | tags=26%, list=6%, signal=28% |
| 426 | GOBP\_REGULATION\_OF\_EPIDERMIS\_DEVELOPMENT |  | 53 | -0.38 | -1.45 | 0.029 | 0.252 | 1.000 | 2254 | tags=28%, list=17%, signal=34% |
| 427 | GOBP\_REGULATION\_OF\_ENDOTHELIAL\_CELL\_DEVELOPMENT |  | 16 | -0.50 | -1.44 | 0.069 | 0.253 | 1.000 | 3137 | tags=50%, list=24%, signal=66% |
| 428 | GOBP\_GASTRULATION\_WITH\_MOUTH\_FORMING\_SECOND |  | 23 | -0.45 | -1.44 | 0.060 | 0.253 | 1.000 | 2868 | tags=39%, list=22%, signal=50% |
| 429 | GOBP\_REGULATION\_OF\_NECROTIC\_CELL\_DEATH |  | 31 | -0.43 | -1.44 | 0.058 | 0.253 | 1.000 | 2903 | tags=35%, list=23%, signal=46% |
| 430 | GOBP\_REGULATION\_OF\_URINE\_VOLUME |  | 20 | -0.47 | -1.44 | 0.079 | 0.254 | 1.000 | 2482 | tags=45%, list=19%, signal=56% |
| 431 | GOBP\_METANEPHRIC\_NEPHRON\_DEVELOPMENT |  | 33 | -0.40 | -1.44 | 0.041 | 0.254 | 1.000 | 850 | tags=18%, list=7%, signal=19% |
| 432 | GOBP\_PEPTIDYL\_LYSINE\_MODIFICATION |  | 323 | -0.28 | -1.44 | 0.003 | 0.254 | 1.000 | 2104 | tags=24%, list=16%, signal=28% |
| 433 | GOBP\_T\_CELL\_DIFFERENTIATION\_IN\_THYMUS |  | 73 | -0.35 | -1.44 | 0.026 | 0.254 | 1.000 | 2393 | tags=29%, list=19%, signal=35% |
| 434 | GOBP\_ADHERENS\_JUNCTION\_ORGANIZATION |  | 43 | -0.38 | -1.44 | 0.033 | 0.255 | 1.000 | 4027 | tags=44%, list=31%, signal=64% |
| 435 | GOBP\_NEURON\_PROJECTION\_EXTENSION |  | 144 | -0.31 | -1.44 | 0.018 | 0.255 | 1.000 | 1934 | tags=24%, list=15%, signal=27% |
| 436 | GOBP\_REGULATION\_OF\_SMALL\_GTPASE\_MEDIATED\_SIGNAL\_TRANSDUCTION |  | 235 | -0.30 | -1.44 | 0.000 | 0.255 | 1.000 | 2902 | tags=29%, list=22%, signal=37% |
| 437 | GOBP\_ATRIOVENTRICULAR\_VALVE\_DEVELOPMENT |  | 25 | -0.44 | -1.44 | 0.069 | 0.255 | 1.000 | 1983 | tags=28%, list=15%, signal=33% |
| 438 | GOBP\_CIRCADIAN\_RHYTHM |  | 179 | -0.31 | -1.44 | 0.012 | 0.254 | 1.000 | 1908 | tags=24%, list=15%, signal=28% |
| 439 | GOBP\_MESONEPHRIC\_TUBULE\_MORPHOGENESIS |  | 54 | -0.37 | -1.44 | 0.037 | 0.257 | 1.000 | 779 | tags=17%, list=6%, signal=18% |
| 440 | GOBP\_NEGATIVE\_REGULATION\_OF\_INTRACELLULAR\_SIGNAL\_TRANSDUCTION |  | 417 | -0.28 | -1.44 | 0.000 | 0.258 | 1.000 | 2340 | tags=24%, list=18%, signal=28% |
| 441 | GOBP\_RESPONSE\_TO\_TUMOR\_CELL |  | 32 | -0.42 | -1.44 | 0.048 | 0.258 | 1.000 | 2916 | tags=38%, list=23%, signal=48% |
| 442 | GOBP\_NEPHRON\_EPITHELIUM\_DEVELOPMENT |  | 94 | -0.33 | -1.44 | 0.022 | 0.260 | 1.000 | 2598 | tags=27%, list=20%, signal=33% |
| 443 | GOBP\_VESICLE\_CYTOSKELETAL\_TRAFFICKING |  | 53 | -0.37 | -1.44 | 0.043 | 0.259 | 1.000 | 3491 | tags=43%, list=27%, signal=59% |
| 444 | GOBP\_OOCYTE\_MATURATION |  | 25 | -0.45 | -1.44 | 0.064 | 0.259 | 1.000 | 2696 | tags=40%, list=21%, signal=50% |
| 445 | GOBP\_NEGATIVE\_REGULATION\_OF\_MRNA\_SPLICING\_VIA\_SPLICEOSOME |  | 18 | -0.48 | -1.44 | 0.078 | 0.259 | 1.000 | 2243 | tags=39%, list=17%, signal=47% |
| 446 | GOBP\_RHO\_PROTEIN\_SIGNAL\_TRANSDUCTION |  | 112 | -0.33 | -1.43 | 0.028 | 0.260 | 1.000 | 4301 | tags=46%, list=33%, signal=68% |
| 447 | GOBP\_RESPONSE\_TO\_FOLLICLE\_STIMULATING\_HORMONE |  | 17 | -0.49 | -1.43 | 0.083 | 0.259 | 1.000 | 4430 | tags=59%, list=34%, signal=89% |
| 448 | GOBP\_AMEBOIDAL\_TYPE\_CELL\_MIGRATION |  | 371 | -0.28 | -1.43 | 0.000 | 0.259 | 1.000 | 2956 | tags=30%, list=23%, signal=38% |
| 449 | GOBP\_MRNA\_3\_END\_PROCESSING |  | 48 | -0.38 | -1.43 | 0.040 | 0.260 | 1.000 | 2715 | tags=31%, list=21%, signal=39% |
| 450 | GOBP\_REGULATION\_OF\_DNA\_TEMPLATED\_TRANSCRIPTION\_INITIATION |  | 51 | -0.38 | -1.43 | 0.033 | 0.260 | 1.000 | 1764 | tags=22%, list=14%, signal=25% |
| 451 | GOBP\_HOMEOSTASIS\_OF\_NUMBER\_OF\_CELLS |  | 243 | -0.29 | -1.43 | 0.006 | 0.260 | 1.000 | 3821 | tags=37%, list=30%, signal=52% |
| 452 | GOBP\_REGULATION\_OF\_BLOOD\_PRESSURE |  | 166 | -0.30 | -1.43 | 0.006 | 0.262 | 1.000 | 2013 | tags=24%, list=16%, signal=28% |
| 453 | GOBP\_NEGATIVE\_REGULATION\_OF\_DEVELOPMENTAL\_GROWTH |  | 92 | -0.33 | -1.43 | 0.014 | 0.263 | 1.000 | 2394 | tags=30%, list=19%, signal=37% |
| 454 | GOBP\_REGULATION\_OF\_CIRCADIAN\_RHYTHM |  | 103 | -0.33 | -1.43 | 0.011 | 0.262 | 1.000 | 2529 | tags=30%, list=20%, signal=37% |
| 455 | GOBP\_PROTEIN\_STABILIZATION |  | 165 | -0.30 | -1.43 | 0.007 | 0.263 | 1.000 | 3229 | tags=34%, list=25%, signal=45% |
| 456 | GOBP\_POSITIVE\_REGULATION\_OF\_REPRODUCTIVE\_PROCESS |  | 64 | -0.36 | -1.43 | 0.030 | 0.264 | 1.000 | 3030 | tags=34%, list=23%, signal=45% |
| 457 | GOBP\_REGULATION\_OF\_GENE\_SILENCING\_BY\_RNA |  | 41 | -0.40 | -1.43 | 0.050 | 0.265 | 1.000 | 2970 | tags=39%, list=23%, signal=51% |
| 458 | GOBP\_POSITIVE\_REGULATION\_OF\_COLLAGEN\_METABOLIC\_PROCESS |  | 23 | -0.45 | -1.43 | 0.062 | 0.265 | 1.000 | 571 | tags=17%, list=4%, signal=18% |
| 459 | GOBP\_CD40\_SIGNALING\_PATHWAY |  | 15 | -0.51 | -1.43 | 0.068 | 0.265 | 1.000 | 570 | tags=27%, list=4%, signal=28% |
| 460 | GOBP\_POSITIVE\_REGULATION\_OF\_RESPONSE\_TO\_DNA\_DAMAGE\_STIMULUS |  | 99 | -0.33 | -1.43 | 0.025 | 0.265 | 1.000 | 2469 | tags=29%, list=19%, signal=36% |
| 461 | GOBP\_MYOBLAST\_DIFFERENTIATION |  | 70 | -0.35 | -1.43 | 0.032 | 0.266 | 1.000 | 2345 | tags=30%, list=18%, signal=36% |
| 462 | GOBP\_RESPONSE\_TO\_CORTICOSTERONE |  | 15 | -0.50 | -1.42 | 0.082 | 0.269 | 1.000 | 2262 | tags=47%, list=18%, signal=57% |
| 463 | GOBP\_NCRNA\_PROCESSING |  | 292 | -0.28 | -1.42 | 0.004 | 0.269 | 1.000 | 2955 | tags=33%, list=23%, signal=42% |
| 464 | GOBP\_CARDIAC\_ATRIUM\_DEVELOPMENT |  | 30 | -0.42 | -1.42 | 0.065 | 0.268 | 1.000 | 3027 | tags=37%, list=23%, signal=48% |
| 465 | GOBP\_RESPONSE\_TO\_FIBROBLAST\_GROWTH\_FACTOR |  | 92 | -0.33 | -1.42 | 0.032 | 0.268 | 1.000 | 2641 | tags=30%, list=20%, signal=38% |
| 466 | GOBP\_DNA\_TEMPLATED\_TRANSCRIPTION\_INITIATION |  | 115 | -0.32 | -1.42 | 0.015 | 0.269 | 1.000 | 2398 | tags=26%, list=19%, signal=32% |
| 467 | GOBP\_MESODERM\_DEVELOPMENT |  | 102 | -0.32 | -1.42 | 0.020 | 0.268 | 1.000 | 4011 | tags=44%, list=31%, signal=64% |
| 468 | GOBP\_NEPHRON\_MORPHOGENESIS |  | 62 | -0.35 | -1.42 | 0.041 | 0.268 | 1.000 | 2427 | tags=26%, list=19%, signal=32% |
| 469 | GOBP\_ERYTHROCYTE\_DEVELOPMENT |  | 32 | -0.41 | -1.42 | 0.056 | 0.267 | 1.000 | 2655 | tags=38%, list=21%, signal=47% |
| 470 | GOBP\_MATURATION\_OF\_5\_8S\_RRNA |  | 32 | -0.40 | -1.42 | 0.068 | 0.267 | 1.000 | 4584 | tags=66%, list=36%, signal=102% |
| 471 | GOBP\_DEVELOPMENTAL\_GROWTH\_INVOLVED\_IN\_MORPHOGENESIS |  | 195 | -0.30 | -1.42 | 0.011 | 0.267 | 1.000 | 1934 | tags=22%, list=15%, signal=25% |
| 472 | GOBP\_PHOTOPERIODISM |  | 27 | -0.43 | -1.42 | 0.057 | 0.268 | 1.000 | 1908 | tags=37%, list=15%, signal=43% |
| 473 | GOBP\_EPIBOLY |  | 31 | -0.42 | -1.42 | 0.069 | 0.270 | 1.000 | 1463 | tags=29%, list=11%, signal=33% |
| 474 | GOBP\_VIRAL\_TRANSCRIPTION |  | 49 | -0.37 | -1.42 | 0.035 | 0.270 | 1.000 | 1831 | tags=27%, list=14%, signal=31% |
| 475 | GOBP\_CELLULAR\_RESPONSE\_TO\_HEAT |  | 58 | -0.36 | -1.42 | 0.035 | 0.270 | 1.000 | 3976 | tags=52%, list=31%, signal=74% |
| 476 | GOBP\_SEROTONIN\_RECEPTOR\_SIGNALING\_PATHWAY |  | 27 | -0.42 | -1.42 | 0.070 | 0.271 | 1.000 | 3003 | tags=44%, list=23%, signal=58% |
| 477 | GOBP\_GLANDULAR\_EPITHELIAL\_CELL\_DIFFERENTIATION |  | 44 | -0.38 | -1.42 | 0.069 | 0.270 | 1.000 | 2881 | tags=36%, list=22%, signal=47% |
| 478 | GOBP\_NEGATIVE\_REGULATION\_OF\_GLIOGENESIS |  | 33 | -0.41 | -1.42 | 0.073 | 0.270 | 1.000 | 1966 | tags=27%, list=15%, signal=32% |
| 479 | GOBP\_CEREBELLAR\_CORTEX\_MORPHOGENESIS |  | 29 | -0.42 | -1.42 | 0.057 | 0.270 | 1.000 | 2791 | tags=38%, list=22%, signal=48% |
| 480 | GOBP\_MAMMARY\_GLAND\_EPITHELIAL\_CELL\_PROLIFERATION |  | 22 | -0.46 | -1.42 | 0.084 | 0.270 | 1.000 | 2066 | tags=36%, list=16%, signal=43% |
| 481 | GOBP\_FOREBRAIN\_CELL\_MIGRATION |  | 52 | -0.37 | -1.42 | 0.041 | 0.270 | 1.000 | 2165 | tags=27%, list=17%, signal=32% |
| 482 | GOBP\_NEGATIVE\_REGULATION\_OF\_CD4\_POSITIVE\_ALPHA\_BETA\_T\_CELL\_DIFFERENTIATION |  | 19 | -0.46 | -1.42 | 0.067 | 0.270 | 1.000 | 4200 | tags=53%, list=33%, signal=78% |
| 483 | GOBP\_ODONTOGENESIS\_OF\_DENTIN\_CONTAINING\_TOOTH |  | 71 | -0.35 | -1.42 | 0.031 | 0.270 | 1.000 | 4391 | tags=48%, list=34%, signal=72% |
| 484 | GOBP\_RESPONSE\_TO\_PEPTIDE |  | 429 | -0.27 | -1.42 | 0.001 | 0.270 | 1.000 | 2810 | tags=28%, list=22%, signal=35% |
| 485 | GOBP\_MAINTENANCE\_OF\_CELL\_NUMBER |  | 118 | -0.32 | -1.42 | 0.015 | 0.270 | 1.000 | 3358 | tags=32%, list=26%, signal=43% |
| 486 | GOBP\_POSITIVE\_REGULATION\_OF\_MUSCLE\_CONTRACTION |  | 39 | -0.39 | -1.42 | 0.049 | 0.270 | 1.000 | 1934 | tags=26%, list=15%, signal=30% |
| 487 | GOBP\_PROTEIN\_HETEROOLIGOMERIZATION |  | 20 | -0.47 | -1.42 | 0.079 | 0.269 | 1.000 | 1415 | tags=30%, list=11%, signal=34% |
| 488 | GOBP\_POSITIVE\_REGULATION\_OF\_PLASMA\_MEMBRANE\_BOUNDED\_CELL\_PROJECTION\_ASSEMBLY |  | 88 | -0.33 | -1.42 | 0.022 | 0.269 | 1.000 | 2820 | tags=32%, list=22%, signal=40% |
| 489 | GOBP\_INTRINSIC\_APOPTOTIC\_SIGNALING\_PATHWAY\_BY\_P53\_CLASS\_MEDIATOR |  | 68 | -0.35 | -1.42 | 0.033 | 0.269 | 1.000 | 2511 | tags=28%, list=19%, signal=35% |
| 490 | GOBP\_POSITIVE\_REGULATION\_OF\_LIPID\_METABOLIC\_PROCESS |  | 131 | -0.31 | -1.41 | 0.023 | 0.271 | 1.000 | 3045 | tags=31%, list=24%, signal=40% |
| 491 | GOBP\_POSITIVE\_REGULATION\_OF\_VASCULATURE\_DEVELOPMENT |  | 142 | -0.31 | -1.41 | 0.015 | 0.271 | 1.000 | 2997 | tags=34%, list=23%, signal=44% |
| 492 | GOBP\_PROGESTERONE\_METABOLIC\_PROCESS |  | 15 | -0.49 | -1.41 | 0.089 | 0.271 | 1.000 | 1359 | tags=27%, list=11%, signal=30% |
| 493 | GOBP\_ACTIVATION\_OF\_PHOSPHOLIPASE\_C\_ACTIVITY |  | 27 | -0.42 | -1.41 | 0.071 | 0.271 | 1.000 | 1924 | tags=30%, list=15%, signal=35% |
| 494 | GOBP\_GAMMA\_AMINOBUTYRIC\_ACID\_TRANSPORT |  | 16 | -0.49 | -1.41 | 0.074 | 0.270 | 1.000 | 2573 | tags=38%, list=20%, signal=47% |
| 495 | GOBP\_PRESYNAPTIC\_ENDOCYTOSIS |  | 52 | -0.37 | -1.41 | 0.043 | 0.271 | 1.000 | 2907 | tags=31%, list=23%, signal=40% |
| 496 | GOBP\_POSITIVE\_REGULATION\_OF\_DNA\_TEMPLATED\_TRANSCRIPTION\_INITIATION |  | 41 | -0.39 | -1.41 | 0.058 | 0.271 | 1.000 | 1764 | tags=22%, list=14%, signal=25% |
| 497 | GOBP\_HINDLIMB\_MORPHOGENESIS |  | 32 | -0.41 | -1.41 | 0.061 | 0.270 | 1.000 | 2944 | tags=41%, list=23%, signal=53% |
| 498 | GOBP\_NEGATIVE\_REGULATION\_OF\_ERK1\_AND\_ERK2\_CASCADE |  | 55 | -0.36 | -1.41 | 0.063 | 0.273 | 1.000 | 1776 | tags=25%, list=14%, signal=29% |
| 499 | GOBP\_CELL\_CYCLE\_G1\_S\_PHASE\_TRANSITION |  | 189 | -0.29 | -1.41 | 0.004 | 0.273 | 1.000 | 1378 | tags=17%, list=11%, signal=19% |
| 500 | GOBP\_REGULATION\_OF\_NEUROGENESIS |  | 297 | -0.28 | -1.41 | 0.005 | 0.273 | 1.000 | 1983 | tags=21%, list=15%, signal=24% |
| 501 | GOBP\_POSITIVE\_REGULATION\_OF\_SMALL\_MOLECULE\_METABOLIC\_PROCESS |  | 125 | -0.31 | -1.41 | 0.021 | 0.273 | 1.000 | 3103 | tags=33%, list=24%, signal=43% |
| 502 | GOBP\_CELL\_SUBSTRATE\_JUNCTION\_ORGANIZATION |  | 91 | -0.33 | -1.41 | 0.034 | 0.275 | 1.000 | 3821 | tags=45%, list=30%, signal=64% |
| 503 | GOBP\_NUCLEAR\_TRANSCRIBED\_MRNA\_CATABOLIC\_PROCESS\_DEADENYLATION\_DEPENDENT\_DECAY |  | 46 | -0.37 | -1.41 | 0.040 | 0.275 | 1.000 | 2525 | tags=37%, list=20%, signal=46% |
| 504 | GOBP\_REGULATION\_OF\_MYELOID\_LEUKOCYTE\_DIFFERENTIATION |  | 100 | -0.32 | -1.41 | 0.030 | 0.276 | 1.000 | 2804 | tags=30%, list=22%, signal=38% |
| 505 | GOBP\_REGULATION\_OF\_EXCRETION |  | 23 | -0.44 | -1.41 | 0.060 | 0.275 | 1.000 | 3268 | tags=39%, list=25%, signal=52% |
| 506 | GOBP\_BLOOD\_VESSEL\_REMODELING |  | 38 | -0.39 | -1.41 | 0.062 | 0.276 | 1.000 | 4102 | tags=50%, list=32%, signal=73% |
| 507 | GOBP\_TRANSLATIONAL\_INITIATION |  | 100 | -0.33 | -1.41 | 0.022 | 0.275 | 1.000 | 3227 | tags=36%, list=25%, signal=48% |
| 508 | GOBP\_MATERNAL\_PLACENTA\_DEVELOPMENT |  | 28 | -0.42 | -1.41 | 0.064 | 0.275 | 1.000 | 2773 | tags=43%, list=21%, signal=54% |
| 509 | GOBP\_NEGATIVE\_REGULATION\_OF\_UBIQUITIN\_DEPENDENT\_PROTEIN\_CATABOLIC\_PROCESS |  | 40 | -0.39 | -1.41 | 0.062 | 0.277 | 1.000 | 2842 | tags=38%, list=22%, signal=48% |
| 510 | GOBP\_DNA\_CATABOLIC\_PROCESS\_ENDONUCLEOLYTIC |  | 23 | -0.44 | -1.41 | 0.070 | 0.276 | 1.000 | 2636 | tags=39%, list=20%, signal=49% |
| 511 | GOBP\_RNA\_POLYADENYLATION |  | 39 | -0.39 | -1.41 | 0.057 | 0.276 | 1.000 | 3116 | tags=38%, list=24%, signal=51% |
| 512 | GOBP\_IMPORT\_INTO\_NUCLEUS |  | 143 | -0.31 | -1.41 | 0.011 | 0.275 | 1.000 | 2926 | tags=33%, list=23%, signal=42% |
| 513 | GOBP\_RESPONSE\_TO\_THYROID\_HORMONE |  | 26 | -0.42 | -1.41 | 0.076 | 0.275 | 1.000 | 3182 | tags=46%, list=25%, signal=61% |
| 514 | GOBP\_SIGNAL\_TRANSDUCTION\_IN\_RESPONSE\_TO\_DNA\_DAMAGE |  | 145 | -0.30 | -1.41 | 0.019 | 0.275 | 1.000 | 2396 | tags=25%, list=19%, signal=30% |
| 515 | GOBP\_REGULATION\_OF\_ERBB\_SIGNALING\_PATHWAY |  | 63 | -0.35 | -1.41 | 0.051 | 0.275 | 1.000 | 3554 | tags=43%, list=28%, signal=59% |
| 516 | GOBP\_NCRNA\_METABOLIC\_PROCESS |  | 391 | -0.27 | -1.41 | 0.006 | 0.275 | 1.000 | 3131 | tags=33%, list=24%, signal=43% |
| 517 | GOBP\_CHROMATIN\_DISASSEMBLY |  | 19 | -0.45 | -1.41 | 0.086 | 0.275 | 1.000 | 4850 | tags=58%, list=38%, signal=93% |
| 518 | GOBP\_RESPONSE\_TO\_PLATELET\_DERIVED\_GROWTH\_FACTOR |  | 21 | -0.45 | -1.41 | 0.069 | 0.274 | 1.000 | 801 | tags=24%, list=6%, signal=25% |
| 519 | GOBP\_REGULATION\_OF\_ACUTE\_INFLAMMATORY\_RESPONSE |  | 40 | -0.39 | -1.41 | 0.063 | 0.274 | 1.000 | 3040 | tags=40%, list=24%, signal=52% |
| 520 | GOBP\_INTRINSIC\_APOPTOTIC\_SIGNALING\_PATHWAY\_IN\_RESPONSE\_TO\_DNA\_DAMAGE\_BY\_P53\_CLASS\_MEDIATOR |  | 39 | -0.39 | -1.41 | 0.060 | 0.274 | 1.000 | 2511 | tags=26%, list=19%, signal=32% |
| 521 | GOBP\_INTESTINAL\_LIPID\_ABSORPTION |  | 16 | -0.47 | -1.41 | 0.101 | 0.273 | 1.000 | 4917 | tags=69%, list=38%, signal=111% |
| 522 | GOBP\_LIVER\_REGENERATION |  | 28 | -0.42 | -1.40 | 0.068 | 0.275 | 1.000 | 1231 | tags=25%, list=10%, signal=28% |
| 523 | GOBP\_NEGATIVE\_REGULATION\_OF\_SMOOTH\_MUSCLE\_CELL\_PROLIFERATION |  | 49 | -0.37 | -1.40 | 0.049 | 0.274 | 1.000 | 3025 | tags=35%, list=23%, signal=45% |
| 524 | GOBP\_POSITIVE\_REGULATION\_OF\_CALCIUM\_ION\_IMPORT |  | 19 | -0.46 | -1.40 | 0.087 | 0.275 | 1.000 | 3394 | tags=58%, list=26%, signal=78% |
| 525 | GOBP\_DETECTION\_OF\_OTHER\_ORGANISM |  | 15 | -0.50 | -1.40 | 0.098 | 0.275 | 1.000 | 1961 | tags=40%, list=15%, signal=47% |
| 526 | GOBP\_POSITIVE\_REGULATION\_OF\_ENDOTHELIAL\_CELL\_MIGRATION |  | 99 | -0.32 | -1.40 | 0.023 | 0.275 | 1.000 | 2254 | tags=29%, list=17%, signal=35% |
| 527 | GOBP\_CELLULAR\_RESPONSE\_TO\_HYDROGEN\_PEROXIDE |  | 78 | -0.33 | -1.40 | 0.040 | 0.274 | 1.000 | 2078 | tags=26%, list=16%, signal=30% |
| 528 | GOBP\_AMINO\_ACID\_TRANSPORT |  | 115 | -0.32 | -1.40 | 0.029 | 0.274 | 1.000 | 2771 | tags=30%, list=21%, signal=37% |
| 529 | GOBP\_POLARIZED\_EPITHELIAL\_CELL\_DIFFERENTIATION |  | 20 | -0.45 | -1.40 | 0.086 | 0.275 | 1.000 | 2969 | tags=40%, list=23%, signal=52% |
| 530 | GOBP\_REGULATION\_OF\_ARP2\_3\_COMPLEX\_MEDIATED\_ACTIN\_NUCLEATION |  | 19 | -0.46 | -1.40 | 0.093 | 0.279 | 1.000 | 2586 | tags=53%, list=20%, signal=66% |
| 531 | GOBP\_CELLULAR\_RESPONSE\_TO\_INSULIN\_STIMULUS |  | 172 | -0.30 | -1.40 | 0.010 | 0.280 | 1.000 | 2804 | tags=31%, list=22%, signal=39% |
| 532 | GOBP\_MESENCHYMAL\_CELL\_PROLIFERATION |  | 40 | -0.38 | -1.40 | 0.070 | 0.279 | 1.000 | 2969 | tags=35%, list=23%, signal=45% |
| 533 | GOBP\_RESPONSE\_TO\_MINERALOCORTICOID |  | 30 | -0.42 | -1.40 | 0.074 | 0.279 | 1.000 | 2262 | tags=40%, list=18%, signal=48% |
| 534 | GOBP\_REGULATION\_OF\_PHOSPHOLIPASE\_C\_ACTIVITY |  | 40 | -0.38 | -1.40 | 0.058 | 0.279 | 1.000 | 2076 | tags=28%, list=16%, signal=33% |
| 535 | GOBP\_RETINA\_LAYER\_FORMATION |  | 19 | -0.45 | -1.40 | 0.088 | 0.279 | 1.000 | 2511 | tags=32%, list=19%, signal=39% |
| 536 | GOBP\_ASTROCYTE\_DIFFERENTIATION |  | 70 | -0.34 | -1.40 | 0.050 | 0.279 | 1.000 | 2791 | tags=37%, list=22%, signal=47% |
| 537 | GOBP\_EPITHELIAL\_CELL\_PROLIFERATION |  | 346 | -0.27 | -1.40 | 0.007 | 0.280 | 1.000 | 2260 | tags=23%, list=18%, signal=27% |
| 538 | GOBP\_CELL\_MATURATION |  | 146 | -0.30 | -1.40 | 0.026 | 0.280 | 1.000 | 2696 | tags=29%, list=21%, signal=36% |
| 539 | GOBP\_REGULATION\_OF\_LIPASE\_ACTIVITY |  | 82 | -0.34 | -1.40 | 0.027 | 0.279 | 1.000 | 2078 | tags=26%, list=16%, signal=30% |
| 540 | GOBP\_POSITIVE\_REGULATION\_OF\_TRANSCRIPTION\_ELONGATION\_FROM\_RNA\_POLYMERASE\_II\_PROMOTER |  | 17 | -0.48 | -1.40 | 0.079 | 0.280 | 1.000 | 2227 | tags=41%, list=17%, signal=50% |
| 541 | GOBP\_CELLULAR\_RESPONSE\_TO\_ABIOTIC\_STIMULUS |  | 276 | -0.28 | -1.40 | 0.003 | 0.280 | 1.000 | 2495 | tags=26%, list=19%, signal=32% |
| 542 | GOBP\_CELL\_PROLIFERATION\_INVOLVED\_IN\_HEART\_MORPHOGENESIS |  | 20 | -0.45 | -1.40 | 0.087 | 0.281 | 1.000 | 402 | tags=20%, list=3%, signal=21% |
| 543 | GOBP\_REGULATION\_OF\_VASCULATURE\_DEVELOPMENT |  | 256 | -0.28 | -1.40 | 0.006 | 0.280 | 1.000 | 4485 | tags=47%, list=35%, signal=71% |
| 544 | GOBP\_VIRAL\_GENE\_EXPRESSION |  | 92 | -0.32 | -1.39 | 0.029 | 0.281 | 1.000 | 2343 | tags=29%, list=18%, signal=36% |
| 545 | GOBP\_ROOF\_OF\_MOUTH\_DEVELOPMENT |  | 74 | -0.34 | -1.39 | 0.040 | 0.284 | 1.000 | 3288 | tags=36%, list=25%, signal=49% |
| 546 | GOBP\_LENS\_MORPHOGENESIS\_IN\_CAMERA\_TYPE\_EYE |  | 20 | -0.44 | -1.39 | 0.085 | 0.283 | 1.000 | 2683 | tags=35%, list=21%, signal=44% |
| 547 | GOBP\_POSITIVE\_REGULATION\_OF\_RECEPTOR\_MEDIATED\_ENDOCYTOSIS |  | 46 | -0.37 | -1.39 | 0.060 | 0.283 | 1.000 | 1711 | tags=33%, list=13%, signal=37% |
| 548 | GOBP\_SODIUM\_ION\_TRANSPORT |  | 182 | -0.29 | -1.39 | 0.021 | 0.284 | 1.000 | 3029 | tags=31%, list=23%, signal=40% |
| 549 | GOBP\_FIBROBLAST\_PROLIFERATION |  | 72 | -0.34 | -1.39 | 0.026 | 0.285 | 1.000 | 1263 | tags=22%, list=10%, signal=24% |
| 550 | GOBP\_RNA\_3\_END\_PROCESSING |  | 82 | -0.33 | -1.39 | 0.047 | 0.285 | 1.000 | 2955 | tags=32%, list=23%, signal=41% |
| 551 | GOBP\_NEGATIVE\_REGULATION\_OF\_T\_HELPER\_CELL\_DIFFERENTIATION |  | 15 | -0.49 | -1.39 | 0.096 | 0.285 | 1.000 | 4200 | tags=53%, list=33%, signal=79% |
| 552 | GOBP\_L\_AMINO\_ACID\_TRANSPORT |  | 52 | -0.36 | -1.39 | 0.052 | 0.285 | 1.000 | 2771 | tags=35%, list=21%, signal=44% |
| 553 | GOBP\_TISSUE\_MIGRATION |  | 275 | -0.28 | -1.39 | 0.003 | 0.286 | 1.000 | 2996 | tags=31%, list=23%, signal=39% |
| 554 | GOBP\_POSITIVE\_REGULATION\_OF\_G\_PROTEIN\_COUPLED\_RECEPTOR\_SIGNALING\_PATHWAY |  | 23 | -0.44 | -1.39 | 0.087 | 0.287 | 1.000 | 2535 | tags=35%, list=20%, signal=43% |
| 555 | GOBP\_REGULATION\_OF\_SYSTEMIC\_ARTERIAL\_BLOOD\_PRESSURE |  | 88 | -0.33 | -1.39 | 0.044 | 0.287 | 1.000 | 2125 | tags=26%, list=16%, signal=31% |
| 556 | GOBP\_EPIDERMAL\_CELL\_DIFFERENTIATION |  | 144 | -0.30 | -1.39 | 0.020 | 0.286 | 1.000 | 4102 | tags=40%, list=32%, signal=58% |
| 557 | GOBP\_REGULATION\_OF\_RAS\_PROTEIN\_SIGNAL\_TRANSDUCTION |  | 155 | -0.30 | -1.39 | 0.024 | 0.286 | 1.000 | 2425 | tags=26%, list=19%, signal=32% |
| 558 | GOBP\_POSITIVE\_REGULATION\_OF\_SODIUM\_ION\_TRANSMEMBRANE\_TRANSPORT |  | 16 | -0.48 | -1.39 | 0.082 | 0.286 | 1.000 | 2430 | tags=44%, list=19%, signal=54% |
| 559 | GOBP\_EPIDERMIS\_DEVELOPMENT |  | 252 | -0.28 | -1.39 | 0.010 | 0.288 | 1.000 | 4102 | tags=38%, list=32%, signal=54% |
| 560 | GOBP\_POSITIVE\_REGULATION\_OF\_NUCLEOTIDE\_METABOLIC\_PROCESS |  | 39 | -0.38 | -1.39 | 0.067 | 0.287 | 1.000 | 3647 | tags=51%, list=28%, signal=71% |
| 561 | GOBP\_REGULATION\_OF\_LIPID\_CATABOLIC\_PROCESS |  | 49 | -0.36 | -1.39 | 0.051 | 0.288 | 1.000 | 2561 | tags=33%, list=20%, signal=41% |
| 562 | GOBP\_SMOOTH\_MUSCLE\_CONTRACTION |  | 96 | -0.32 | -1.39 | 0.027 | 0.288 | 1.000 | 2503 | tags=27%, list=19%, signal=33% |
| 563 | GOBP\_GLIAL\_CELL\_PROLIFERATION |  | 37 | -0.38 | -1.39 | 0.068 | 0.288 | 1.000 | 1284 | tags=24%, list=10%, signal=27% |
| 564 | GOBP\_POSITIVE\_REGULATION\_OF\_VIRAL\_GENOME\_REPLICATION |  | 31 | -0.41 | -1.39 | 0.062 | 0.288 | 1.000 | 2607 | tags=39%, list=20%, signal=48% |
| 565 | GOBP\_NEGATIVE\_REGULATION\_OF\_LYASE\_ACTIVITY |  | 22 | -0.44 | -1.39 | 0.080 | 0.288 | 1.000 | 1926 | tags=32%, list=15%, signal=37% |
| 566 | GOBP\_ACID\_SECRETION |  | 39 | -0.39 | -1.39 | 0.086 | 0.288 | 1.000 | 3095 | tags=38%, list=24%, signal=50% |
| 567 | GOBP\_CELLULAR\_RESPONSE\_TO\_ORGANIC\_CYCLIC\_COMPOUND |  | 498 | -0.26 | -1.38 | 0.000 | 0.287 | 1.000 | 3205 | tags=30%, list=25%, signal=39% |
| 568 | GOBP\_OOGENESIS |  | 70 | -0.34 | -1.38 | 0.037 | 0.287 | 1.000 | 3030 | tags=31%, list=23%, signal=41% |
| 569 | GOBP\_REGULATION\_OF\_TRANSLATION\_IN\_RESPONSE\_TO\_STRESS |  | 17 | -0.47 | -1.38 | 0.105 | 0.287 | 1.000 | 795 | tags=29%, list=6%, signal=31% |
| 570 | GOBP\_METANEPHRIC\_NEPHRON\_MORPHOGENESIS |  | 21 | -0.45 | -1.38 | 0.098 | 0.288 | 1.000 | 817 | tags=19%, list=6%, signal=20% |
| 571 | GOBP\_CHROMATIN\_REMODELING |  | 208 | -0.29 | -1.38 | 0.020 | 0.288 | 1.000 | 2302 | tags=24%, list=18%, signal=28% |
| 572 | GOBP\_POSITIVE\_REGULATION\_OF\_GLUCOSE\_IMPORT |  | 31 | -0.40 | -1.38 | 0.081 | 0.289 | 1.000 | 1803 | tags=29%, list=14%, signal=34% |
| 573 | GOBP\_MORPHOGENESIS\_OF\_A\_BRANCHING\_STRUCTURE |  | 173 | -0.29 | -1.38 | 0.016 | 0.289 | 1.000 | 4158 | tags=40%, list=32%, signal=59% |
| 574 | GOBP\_ORGAN\_GROWTH |  | 137 | -0.30 | -1.38 | 0.018 | 0.288 | 1.000 | 3027 | tags=31%, list=23%, signal=41% |
| 575 | GOBP\_IRON\_ION\_TRANSPORT |  | 45 | -0.37 | -1.38 | 0.051 | 0.288 | 1.000 | 3953 | tags=53%, list=31%, signal=77% |
| 576 | GOBP\_PROTEIN\_SUMOYLATION |  | 44 | -0.37 | -1.38 | 0.077 | 0.288 | 1.000 | 2614 | tags=32%, list=20%, signal=40% |
| 577 | GOBP\_REGULATION\_OF\_CYCLIN\_DEPENDENT\_PROTEIN\_KINASE\_ACTIVITY |  | 83 | -0.33 | -1.38 | 0.053 | 0.288 | 1.000 | 2842 | tags=33%, list=22%, signal=41% |
| 578 | GOBP\_POSITIVE\_REGULATION\_OF\_CARBOHYDRATE\_METABOLIC\_PROCESS |  | 68 | -0.34 | -1.38 | 0.037 | 0.289 | 1.000 | 3103 | tags=34%, list=24%, signal=44% |
| 579 | GOBP\_MYELOID\_CELL\_DIFFERENTIATION |  | 337 | -0.27 | -1.38 | 0.008 | 0.289 | 1.000 | 2761 | tags=26%, list=21%, signal=32% |
| 580 | GOBP\_NEGATIVE\_REGULATION\_OF\_RESPONSE\_TO\_DNA\_DAMAGE\_STIMULUS |  | 65 | -0.34 | -1.38 | 0.053 | 0.288 | 1.000 | 1713 | tags=23%, list=13%, signal=26% |
| 581 | GOBP\_NEGATIVE\_REGULATION\_OF\_NERVOUS\_SYSTEM\_DEVELOPMENT |  | 120 | -0.31 | -1.38 | 0.036 | 0.290 | 1.000 | 1966 | tags=25%, list=15%, signal=29% |
| 582 | GOBP\_SOMATIC\_STEM\_CELL\_POPULATION\_MAINTENANCE |  | 39 | -0.38 | -1.38 | 0.069 | 0.289 | 1.000 | 4380 | tags=49%, list=34%, signal=74% |
| 583 | GOBP\_REGULATION\_OF\_COLLAGEN\_METABOLIC\_PROCESS |  | 33 | -0.40 | -1.38 | 0.089 | 0.289 | 1.000 | 571 | tags=18%, list=4%, signal=19% |
| 584 | GOBP\_HEART\_DEVELOPMENT |  | 477 | -0.26 | -1.38 | 0.005 | 0.290 | 1.000 | 2024 | tags=21%, list=16%, signal=24% |
| 585 | GOBP\_VASCULAR\_ENDOTHELIAL\_CELL\_PROLIFERATION |  | 19 | -0.46 | -1.38 | 0.102 | 0.290 | 1.000 | 4997 | tags=63%, list=39%, signal=103% |
| 586 | GOBP\_CD4\_POSITIVE\_ALPHA\_BETA\_T\_CELL\_DIFFERENTIATION |  | 67 | -0.34 | -1.38 | 0.055 | 0.290 | 1.000 | 2085 | tags=27%, list=16%, signal=32% |
| 587 | GOBP\_NEGATIVE\_REGULATION\_OF\_HORMONE\_SECRETION |  | 49 | -0.36 | -1.38 | 0.050 | 0.290 | 1.000 | 2445 | tags=31%, list=19%, signal=38% |
| 588 | GOBP\_SEMAPHORIN\_PLEXIN\_SIGNALING\_PATHWAY |  | 40 | -0.38 | -1.38 | 0.082 | 0.289 | 1.000 | 3172 | tags=40%, list=25%, signal=53% |
| 589 | GOBP\_REGULATION\_OF\_CALCIUM\_ION\_IMPORT |  | 37 | -0.39 | -1.38 | 0.078 | 0.290 | 1.000 | 3394 | tags=43%, list=26%, signal=59% |
| 590 | GOBP\_SISTER\_CHROMATID\_COHESION |  | 43 | -0.37 | -1.38 | 0.073 | 0.290 | 1.000 | 3392 | tags=40%, list=26%, signal=53% |
| 591 | GOBP\_REGULATION\_OF\_PLATELET\_DERIVED\_GROWTH\_FACTOR\_RECEPTOR\_SIGNALING\_PATHWAY |  | 21 | -0.44 | -1.38 | 0.095 | 0.290 | 1.000 | 3439 | tags=48%, list=27%, signal=65% |
| 592 | GOBP\_REGULATION\_OF\_MUSCLE\_ADAPTATION |  | 67 | -0.34 | -1.38 | 0.058 | 0.290 | 1.000 | 1956 | tags=28%, list=15%, signal=33% |
| 593 | GOBP\_CELL\_AGING |  | 103 | -0.32 | -1.38 | 0.044 | 0.290 | 1.000 | 3955 | tags=42%, list=31%, signal=60% |
| 594 | GOBP\_REPLICATION\_FORK\_PROCESSING |  | 27 | -0.41 | -1.38 | 0.092 | 0.290 | 1.000 | 1831 | tags=33%, list=14%, signal=39% |
| 595 | GOBP\_REGULATION\_OF\_RECEPTOR\_INTERNALIZATION |  | 44 | -0.37 | -1.38 | 0.056 | 0.290 | 1.000 | 2604 | tags=39%, list=20%, signal=48% |
| 596 | GOBP\_OSTEOBLAST\_DIFFERENTIATION |  | 199 | -0.29 | -1.38 | 0.023 | 0.290 | 1.000 | 2016 | tags=23%, list=16%, signal=27% |
| 597 | GOBP\_INOSITOL\_PHOSPHATE\_METABOLIC\_PROCESS |  | 50 | -0.36 | -1.37 | 0.083 | 0.291 | 1.000 | 1325 | tags=20%, list=10%, signal=22% |
| 598 | GOBP\_CELLULAR\_RESPONSE\_TO\_LIGHT\_STIMULUS |  | 97 | -0.32 | -1.37 | 0.036 | 0.291 | 1.000 | 2600 | tags=29%, list=20%, signal=36% |
| 599 | GOBP\_REGULATION\_OF\_DNA\_BINDING\_TRANSCRIPTION\_FACTOR\_ACTIVITY |  | 363 | -0.27 | -1.37 | 0.007 | 0.291 | 1.000 | 3609 | tags=36%, list=28%, signal=48% |
| 600 | GOBP\_AMINO\_ACID\_IMPORT |  | 49 | -0.37 | -1.37 | 0.063 | 0.291 | 1.000 | 2998 | tags=35%, list=23%, signal=45% |
| 601 | GOBP\_REGULATION\_OF\_P38MAPK\_CASCADE |  | 39 | -0.38 | -1.37 | 0.073 | 0.291 | 1.000 | 4691 | tags=59%, list=36%, signal=92% |
| 602 | GOBP\_NUCLEOTIDE\_BINDING\_DOMAIN\_LEUCINE\_RICH\_REPEAT\_CONTAINING\_RECEPTOR\_SIGNALING\_PATHWAY |  | 21 | -0.45 | -1.37 | 0.084 | 0.291 | 1.000 | 1102 | tags=24%, list=9%, signal=26% |
| 603 | GOBP\_REGULATION\_OF\_DNA\_REPLICATION |  | 108 | -0.32 | -1.37 | 0.035 | 0.291 | 1.000 | 2593 | tags=34%, list=20%, signal=43% |
| 604 | GOBP\_PEPTIDYL\_SERINE\_DEPHOSPHORYLATION |  | 16 | -0.48 | -1.37 | 0.113 | 0.292 | 1.000 | 795 | tags=25%, list=6%, signal=27% |
| 605 | GOBP\_POSITIVE\_REGULATION\_OF\_LAMELLIPODIUM\_ORGANIZATION |  | 31 | -0.39 | -1.37 | 0.074 | 0.292 | 1.000 | 2196 | tags=32%, list=17%, signal=39% |
| 606 | GOBP\_PROTEIN\_KINASE\_C\_SIGNALING |  | 26 | -0.42 | -1.37 | 0.085 | 0.293 | 1.000 | 1774 | tags=31%, list=14%, signal=36% |
| 607 | GOBP\_NEGATIVE\_REGULATION\_OF\_CELL\_CYCLE |  | 300 | -0.27 | -1.37 | 0.004 | 0.292 | 1.000 | 3420 | tags=31%, list=27%, signal=42% |
| 608 | GOBP\_EMBRYONIC\_ORGAN\_DEVELOPMENT |  | 363 | -0.27 | -1.37 | 0.008 | 0.295 | 1.000 | 3098 | tags=29%, list=24%, signal=38% |
| 609 | GOBP\_PEROXISOME\_PROLIFERATOR\_ACTIVATED\_RECEPTOR\_SIGNALING\_PATHWAY |  | 19 | -0.46 | -1.37 | 0.087 | 0.296 | 1.000 | 5521 | tags=74%, list=43%, signal=129% |
| 610 | GOBP\_POSITIVE\_REGULATION\_OF\_NEUROGENESIS |  | 188 | -0.29 | -1.37 | 0.016 | 0.297 | 1.000 | 3182 | tags=29%, list=25%, signal=38% |
| 611 | GOBP\_COLLAGEN\_METABOLIC\_PROCESS |  | 87 | -0.32 | -1.37 | 0.051 | 0.297 | 1.000 | 4321 | tags=41%, list=33%, signal=62% |
| 612 | GOBP\_POSITIVE\_REGULATION\_OF\_BLOOD\_CIRCULATION |  | 35 | -0.38 | -1.37 | 0.071 | 0.297 | 1.000 | 1934 | tags=29%, list=15%, signal=34% |
| 613 | GOBP\_T\_CELL\_DIFFERENTIATION\_INVOLVED\_IN\_IMMUNE\_RESPONSE |  | 61 | -0.34 | -1.37 | 0.069 | 0.300 | 1.000 | 2080 | tags=26%, list=16%, signal=31% |
| 614 | GOBP\_POSITIVE\_REGULATION\_OF\_GROWTH |  | 215 | -0.28 | -1.37 | 0.011 | 0.299 | 1.000 | 1626 | tags=19%, list=13%, signal=21% |
| 615 | GOBP\_NEURON\_MIGRATION |  | 132 | -0.30 | -1.37 | 0.023 | 0.299 | 1.000 | 2189 | tags=26%, list=17%, signal=31% |
| 616 | GOBP\_REGULATION\_OF\_WOUND\_HEALING |  | 113 | -0.31 | -1.37 | 0.030 | 0.299 | 1.000 | 3219 | tags=34%, list=25%, signal=44% |
| 617 | GOBP\_CYCLIC\_NUCLEOTIDE\_METABOLIC\_PROCESS |  | 36 | -0.39 | -1.37 | 0.070 | 0.299 | 1.000 | 3482 | tags=44%, list=27%, signal=61% |
| 618 | GOBP\_MELANOCYTE\_DIFFERENTIATION |  | 23 | -0.43 | -1.36 | 0.110 | 0.300 | 1.000 | 1565 | tags=30%, list=12%, signal=35% |
| 619 | GOBP\_RESPONSE\_TO\_OXYGEN\_LEVELS |  | 280 | -0.27 | -1.36 | 0.013 | 0.300 | 1.000 | 3001 | tags=28%, list=23%, signal=36% |
| 620 | GOBP\_REGULATION\_OF\_MRNA\_POLYADENYLATION |  | 18 | -0.46 | -1.36 | 0.108 | 0.300 | 1.000 | 2215 | tags=39%, list=17%, signal=47% |
| 621 | GOBP\_NEGATIVE\_REGULATION\_OF\_I\_KAPPAB\_KINASE\_NF\_KAPPAB\_SIGNALING |  | 36 | -0.38 | -1.36 | 0.081 | 0.300 | 1.000 | 949 | tags=22%, list=7%, signal=24% |
| 622 | GOBP\_POSITIVE\_REGULATION\_OF\_EPITHELIAL\_CELL\_DIFFERENTIATION |  | 52 | -0.35 | -1.36 | 0.076 | 0.299 | 1.000 | 4281 | tags=48%, list=33%, signal=72% |
| 623 | GOBP\_TELENCEPHALON\_DEVELOPMENT |  | 196 | -0.29 | -1.36 | 0.018 | 0.300 | 1.000 | 2165 | tags=22%, list=17%, signal=26% |
| 624 | GOBP\_NEGATIVE\_REGULATION\_OF\_TOR\_SIGNALING |  | 30 | -0.40 | -1.36 | 0.087 | 0.302 | 1.000 | 1587 | tags=30%, list=12%, signal=34% |
| 625 | GOBP\_REGULATION\_OF\_GLUCOSE\_IMPORT |  | 49 | -0.35 | -1.36 | 0.061 | 0.302 | 1.000 | 3267 | tags=39%, list=25%, signal=52% |
| 626 | GOBP\_AMINO\_ACID\_IMPORT\_ACROSS\_PLASMA\_MEMBRANE |  | 41 | -0.37 | -1.36 | 0.077 | 0.303 | 1.000 | 2771 | tags=34%, list=21%, signal=43% |
| 627 | GOBP\_PHARYNGEAL\_SYSTEM\_DEVELOPMENT |  | 26 | -0.42 | -1.36 | 0.096 | 0.304 | 1.000 | 2024 | tags=38%, list=16%, signal=46% |
| 628 | GOBP\_DENDRITE\_MORPHOGENESIS |  | 121 | -0.30 | -1.36 | 0.035 | 0.304 | 1.000 | 3058 | tags=30%, list=24%, signal=39% |
| 629 | GOBP\_DOSAGE\_COMPENSATION |  | 19 | -0.45 | -1.36 | 0.110 | 0.304 | 1.000 | 3055 | tags=42%, list=24%, signal=55% |
| 630 | GOBP\_GASTRULATION |  | 155 | -0.29 | -1.36 | 0.030 | 0.303 | 1.000 | 2997 | tags=33%, list=23%, signal=42% |
| 631 | GOBP\_DEVELOPMENTAL\_PIGMENTATION |  | 40 | -0.38 | -1.36 | 0.090 | 0.305 | 1.000 | 2052 | tags=30%, list=16%, signal=36% |
| 632 | GOBP\_DNA\_DAMAGE\_RESPONSE\_SIGNAL\_TRANSDUCTION\_BY\_P53\_CLASS\_MEDIATOR |  | 64 | -0.34 | -1.36 | 0.070 | 0.305 | 1.000 | 3205 | tags=34%, list=25%, signal=46% |
| 633 | GOBP\_POSITIVE\_REGULATION\_OF\_GLUCOSE\_METABOLIC\_PROCESS |  | 34 | -0.39 | -1.36 | 0.072 | 0.305 | 1.000 | 3103 | tags=38%, list=24%, signal=50% |
| 634 | GOBP\_POSITIVE\_REGULATION\_OF\_DNA\_TEMPLATED\_TRANSCRIPTION\_ELONGATION |  | 27 | -0.42 | -1.36 | 0.081 | 0.305 | 1.000 | 2227 | tags=37%, list=17%, signal=45% |
| 635 | GOBP\_CELL\_MATRIX\_ADHESION |  | 203 | -0.28 | -1.36 | 0.016 | 0.306 | 1.000 | 3845 | tags=39%, list=30%, signal=55% |
| 636 | GOBP\_SPERMATID\_NUCLEUS\_DIFFERENTIATION |  | 15 | -0.48 | -1.36 | 0.131 | 0.305 | 1.000 | 3274 | tags=47%, list=25%, signal=62% |
| 637 | GOBP\_POSITIVE\_REGULATION\_OF\_DNA\_BINDING\_TRANSCRIPTION\_FACTOR\_ACTIVITY |  | 231 | -0.28 | -1.36 | 0.016 | 0.306 | 1.000 | 3593 | tags=37%, list=28%, signal=51% |
| 638 | GOBP\_DNA\_DAMAGE\_RESPONSE\_SIGNAL\_TRANSDUCTION\_BY\_P53\_CLASS\_MEDIATOR\_RESULTING\_IN\_CELL\_CYCLE\_ARREST |  | 16 | -0.47 | -1.36 | 0.107 | 0.306 | 1.000 | 730 | tags=19%, list=6%, signal=20% |
| 639 | GOBP\_SOMITE\_DEVELOPMENT |  | 65 | -0.34 | -1.36 | 0.071 | 0.306 | 1.000 | 4102 | tags=43%, list=32%, signal=63% |
| 640 | GOBP\_MORPHOGENESIS\_OF\_AN\_EPITHELIUM |  | 414 | -0.26 | -1.36 | 0.001 | 0.307 | 1.000 | 2719 | tags=25%, list=21%, signal=31% |
| 641 | GOBP\_REGULATION\_OF\_GLUCONEOGENESIS |  | 39 | -0.37 | -1.36 | 0.081 | 0.307 | 1.000 | 2765 | tags=36%, list=21%, signal=46% |
| 642 | GOBP\_SENSORY\_SYSTEM\_DEVELOPMENT |  | 315 | -0.27 | -1.36 | 0.006 | 0.307 | 1.000 | 3763 | tags=32%, list=29%, signal=45% |
| 643 | GOBP\_REGULATION\_OF\_REPRODUCTIVE\_PROCESS |  | 132 | -0.30 | -1.36 | 0.040 | 0.307 | 1.000 | 3843 | tags=39%, list=30%, signal=56% |
| 644 | GOBP\_REGULATION\_OF\_ADENYLATE\_CYCLASE\_ACTIVITY |  | 28 | -0.41 | -1.35 | 0.087 | 0.310 | 1.000 | 1551 | tags=25%, list=12%, signal=28% |
| 645 | GOBP\_POSITIVE\_REGULATION\_OF\_CELL\_DEATH |  | 493 | -0.26 | -1.35 | 0.003 | 0.311 | 1.000 | 3214 | tags=30%, list=25%, signal=38% |
| 646 | GOBP\_NEGATIVE\_REGULATION\_OF\_EXTRINSIC\_APOPTOTIC\_SIGNALING\_PATHWAY\_VIA\_DEATH\_DOMAIN\_RECEPTORS |  | 24 | -0.41 | -1.35 | 0.092 | 0.311 | 1.000 | 3242 | tags=42%, list=25%, signal=56% |
| 647 | GOBP\_OLEFINIC\_COMPOUND\_BIOSYNTHETIC\_PROCESS |  | 24 | -0.42 | -1.35 | 0.117 | 0.311 | 1.000 | 2951 | tags=42%, list=23%, signal=54% |
| 648 | GOBP\_POSITIVE\_REGULATION\_OF\_FILOPODIUM\_ASSEMBLY |  | 25 | -0.42 | -1.35 | 0.114 | 0.312 | 1.000 | 2820 | tags=36%, list=22%, signal=46% |
| 649 | GOBP\_POSITIVE\_REGULATION\_OF\_OXIDATIVE\_STRESS\_INDUCED\_CELL\_DEATH |  | 18 | -0.45 | -1.35 | 0.123 | 0.312 | 1.000 | 1961 | tags=33%, list=15%, signal=39% |
| 650 | GOBP\_NEGATIVE\_REGULATION\_OF\_CELL\_ADHESION |  | 246 | -0.27 | -1.35 | 0.016 | 0.314 | 1.000 | 4200 | tags=43%, list=33%, signal=62% |
| 651 | GOBP\_CAMERA\_TYPE\_EYE\_DEVELOPMENT |  | 272 | -0.27 | -1.35 | 0.009 | 0.314 | 1.000 | 3763 | tags=33%, list=29%, signal=45% |
| 652 | GOBP\_OSSIFICATION |  | 354 | -0.26 | -1.35 | 0.008 | 0.313 | 1.000 | 2291 | tags=23%, list=18%, signal=28% |
| 653 | GOBP\_NEGATIVE\_REGULATION\_OF\_DNA\_BINDING\_TRANSCRIPTION\_FACTOR\_ACTIVITY |  | 137 | -0.30 | -1.35 | 0.032 | 0.313 | 1.000 | 1471 | tags=19%, list=11%, signal=21% |
| 654 | GOBP\_RIBOSOMAL\_SMALL\_SUBUNIT\_ASSEMBLY |  | 17 | -0.46 | -1.35 | 0.124 | 0.313 | 1.000 | 2453 | tags=47%, list=19%, signal=58% |
| 655 | GOBP\_POSITIVE\_REGULATION\_OF\_CATABOLIC\_PROCESS |  | 410 | -0.26 | -1.35 | 0.005 | 0.313 | 1.000 | 2567 | tags=26%, list=20%, signal=32% |
| 656 | GOBP\_CELL\_MORPHOGENESIS\_INVOLVED\_IN\_NEURON\_DIFFERENTIATION |  | 453 | -0.26 | -1.35 | 0.003 | 0.314 | 1.000 | 2882 | tags=26%, list=22%, signal=33% |
| 657 | GOBP\_CELLULAR\_RESPONSE\_TO\_INTERFERON\_GAMMA |  | 97 | -0.31 | -1.35 | 0.053 | 0.314 | 1.000 | 3421 | tags=35%, list=27%, signal=47% |
| 658 | GOBP\_MODULATION\_BY\_HOST\_OF\_SYMBIONT\_PROCESS |  | 60 | -0.34 | -1.35 | 0.065 | 0.314 | 1.000 | 3030 | tags=35%, list=23%, signal=46% |
| 659 | GOBP\_RNA\_POLYMERASE\_II\_PREINITIATION\_COMPLEX\_ASSEMBLY |  | 24 | -0.41 | -1.35 | 0.111 | 0.314 | 1.000 | 732 | tags=17%, list=6%, signal=18% |
| 660 | GOBP\_NEGATIVE\_REGULATION\_OF\_CELL\_CYCLE\_PROCESS |  | 232 | -0.28 | -1.35 | 0.013 | 0.314 | 1.000 | 3420 | tags=31%, list=27%, signal=41% |
| 661 | GOBP\_POSITIVE\_REGULATION\_OF\_LIPASE\_ACTIVITY |  | 61 | -0.34 | -1.35 | 0.073 | 0.315 | 1.000 | 2076 | tags=25%, list=16%, signal=29% |
| 662 | GOBP\_SKELETAL\_MUSCLE\_ORGAN\_DEVELOPMENT |  | 129 | -0.30 | -1.35 | 0.036 | 0.314 | 1.000 | 3330 | tags=33%, list=26%, signal=43% |
| 663 | GOBP\_CARDIOCYTE\_DIFFERENTIATION |  | 119 | -0.30 | -1.35 | 0.046 | 0.314 | 1.000 | 2103 | tags=25%, list=16%, signal=30% |
| 664 | GOBP\_CELLULAR\_RESPONSE\_TO\_PH |  | 20 | -0.44 | -1.35 | 0.119 | 0.314 | 1.000 | 3957 | tags=55%, list=31%, signal=79% |
| 665 | GOBP\_POSITIVE\_REGULATION\_OF\_TRANSFORMING\_GROWTH\_FACTOR\_BETA\_PRODUCTION |  | 20 | -0.44 | -1.35 | 0.115 | 0.314 | 1.000 | 1404 | tags=20%, list=11%, signal=22% |
| 666 | GOBP\_CARBOHYDRATE\_TRANSMEMBRANE\_TRANSPORT |  | 92 | -0.31 | -1.35 | 0.047 | 0.314 | 1.000 | 3281 | tags=36%, list=25%, signal=48% |
| 667 | GOBP\_STRIATED\_MUSCLE\_CELL\_PROLIFERATION |  | 54 | -0.35 | -1.35 | 0.076 | 0.314 | 1.000 | 1601 | tags=22%, list=12%, signal=25% |
| 668 | GOBP\_POSITIVE\_REGULATION\_OF\_CELL\_DIVISION |  | 78 | -0.32 | -1.35 | 0.066 | 0.315 | 1.000 | 960 | tags=15%, list=7%, signal=17% |
| 669 | GOBP\_CELLULAR\_RESPONSE\_TO\_PEPTIDE\_HORMONE\_STIMULUS |  | 250 | -0.27 | -1.34 | 0.020 | 0.315 | 1.000 | 3100 | tags=31%, list=24%, signal=40% |
| 670 | GOBP\_EMBRYONIC\_MORPHOGENESIS |  | 490 | -0.25 | -1.34 | 0.009 | 0.315 | 1.000 | 3108 | tags=30%, list=24%, signal=39% |
| 671 | GOBP\_RESPONSE\_TO\_CORTICOSTEROID |  | 144 | -0.29 | -1.34 | 0.038 | 0.315 | 1.000 | 2084 | tags=24%, list=16%, signal=28% |
| 672 | GOBP\_ADENYLATE\_CYCLASE\_MODULATING\_G\_PROTEIN\_COUPLED\_RECEPTOR\_SIGNALING\_PATHWAY |  | 212 | -0.28 | -1.34 | 0.017 | 0.315 | 1.000 | 3383 | tags=31%, list=26%, signal=42% |
| 673 | GOBP\_MITOTIC\_G2\_DNA\_DAMAGE\_CHECKPOINT\_SIGNALING |  | 30 | -0.40 | -1.34 | 0.087 | 0.317 | 1.000 | 1444 | tags=20%, list=11%, signal=22% |
| 674 | GOBP\_INTERFERON\_GAMMA\_MEDIATED\_SIGNALING\_PATHWAY |  | 20 | -0.44 | -1.34 | 0.127 | 0.316 | 1.000 | 3346 | tags=35%, list=26%, signal=47% |
| 675 | GOBP\_CELL\_DIFFERENTIATION\_INVOLVED\_IN\_METANEPHROS\_DEVELOPMENT |  | 22 | -0.43 | -1.34 | 0.096 | 0.318 | 1.000 | 817 | tags=18%, list=6%, signal=19% |
| 676 | GOBP\_LEUKOCYTE\_DIFFERENTIATION |  | 469 | -0.25 | -1.34 | 0.004 | 0.320 | 1.000 | 2316 | tags=23%, list=18%, signal=27% |
| 677 | GOBP\_PHOSPHATIDYLSERINE\_METABOLIC\_PROCESS |  | 16 | -0.46 | -1.34 | 0.113 | 0.322 | 1.000 | 3441 | tags=44%, list=27%, signal=60% |
| 678 | GOBP\_POSITIVE\_REGULATION\_OF\_NUCLEOTIDE\_BIOSYNTHETIC\_PROCESS |  | 21 | -0.43 | -1.34 | 0.093 | 0.321 | 1.000 | 3647 | tags=62%, list=28%, signal=86% |
| 679 | GOBP\_RESPONSE\_TO\_STARVATION |  | 158 | -0.29 | -1.34 | 0.030 | 0.323 | 1.000 | 2892 | tags=34%, list=22%, signal=44% |
| 680 | GOBP\_CELLULAR\_RESPONSE\_TO\_STARVATION |  | 128 | -0.30 | -1.34 | 0.044 | 0.324 | 1.000 | 2892 | tags=34%, list=22%, signal=44% |
| 681 | GOBP\_DENDRITIC\_SPINE\_MORPHOGENESIS |  | 47 | -0.36 | -1.34 | 0.089 | 0.324 | 1.000 | 3215 | tags=36%, list=25%, signal=48% |
| 682 | GOBP\_HEPATICOBILIARY\_SYSTEM\_DEVELOPMENT |  | 126 | -0.30 | -1.34 | 0.040 | 0.324 | 1.000 | 2819 | tags=28%, list=22%, signal=35% |
| 683 | GOBP\_REGULATION\_OF\_TRANSLATIONAL\_INITIATION |  | 71 | -0.33 | -1.34 | 0.053 | 0.325 | 1.000 | 3151 | tags=35%, list=24%, signal=46% |
| 684 | GOBP\_REGULATION\_OF\_EPITHELIAL\_TO\_MESENCHYMAL\_TRANSITION |  | 73 | -0.33 | -1.34 | 0.069 | 0.324 | 1.000 | 1206 | tags=19%, list=9%, signal=21% |
| 685 | GOBP\_MONONUCLEAR\_CELL\_DIFFERENTIATION |  | 368 | -0.26 | -1.34 | 0.008 | 0.324 | 1.000 | 2316 | tags=23%, list=18%, signal=27% |
| 686 | GOBP\_ACTIN\_CYTOSKELETON\_REORGANIZATION |  | 90 | -0.31 | -1.34 | 0.048 | 0.325 | 1.000 | 3137 | tags=32%, list=24%, signal=42% |
| 687 | GOBP\_POSITIVE\_REGULATION\_OF\_HISTONE\_MODIFICATION |  | 89 | -0.31 | -1.33 | 0.068 | 0.327 | 1.000 | 872 | tags=17%, list=7%, signal=18% |
| 688 | GOBP\_RESPIRATORY\_SYSTEM\_DEVELOPMENT |  | 175 | -0.28 | -1.33 | 0.037 | 0.327 | 1.000 | 1934 | tags=21%, list=15%, signal=24% |
| 689 | GOBP\_NEGATIVE\_REGULATION\_OF\_AXONOGENESIS |  | 56 | -0.34 | -1.33 | 0.068 | 0.327 | 1.000 | 1917 | tags=29%, list=15%, signal=33% |
| 690 | GOBP\_GLUCOSE\_IMPORT |  | 62 | -0.33 | -1.33 | 0.079 | 0.328 | 1.000 | 3281 | tags=39%, list=25%, signal=52% |
| 691 | GOBP\_CELL\_FATE\_COMMITMENT\_INVOLVED\_IN\_FORMATION\_OF\_PRIMARY\_GERM\_LAYER |  | 26 | -0.41 | -1.33 | 0.116 | 0.329 | 1.000 | 2059 | tags=31%, list=16%, signal=37% |
| 692 | GOBP\_BIOLOGICAL\_PROCESS\_INVOLVED\_IN\_SYMBIOTIC\_INTERACTION |  | 253 | -0.27 | -1.33 | 0.032 | 0.328 | 1.000 | 3179 | tags=32%, list=25%, signal=42% |
| 693 | GOBP\_POSITIVE\_REGULATION\_OF\_CYCLASE\_ACTIVITY |  | 19 | -0.44 | -1.33 | 0.128 | 0.329 | 1.000 | 3946 | tags=63%, list=31%, signal=91% |
| 694 | GOBP\_AMINO\_ACID\_TRANSMEMBRANE\_TRANSPORT |  | 73 | -0.32 | -1.33 | 0.072 | 0.329 | 1.000 | 2771 | tags=32%, list=21%, signal=40% |
| 695 | GOBP\_POSITIVE\_REGULATION\_OF\_EPITHELIAL\_CELL\_MIGRATION |  | 138 | -0.29 | -1.33 | 0.037 | 0.329 | 1.000 | 2254 | tags=25%, list=17%, signal=30% |
| 696 | GOBP\_REGULATION\_OF\_PROTEIN\_DEACETYLATION |  | 48 | -0.35 | -1.33 | 0.094 | 0.329 | 1.000 | 1403 | tags=21%, list=11%, signal=23% |
| 697 | GOBP\_REGULATION\_OF\_STEM\_CELL\_POPULATION\_MAINTENANCE |  | 30 | -0.39 | -1.33 | 0.114 | 0.331 | 1.000 | 639 | tags=17%, list=5%, signal=17% |
| 698 | GOBP\_RESPONSE\_TO\_COLD |  | 42 | -0.36 | -1.33 | 0.087 | 0.331 | 1.000 | 2460 | tags=33%, list=19%, signal=41% |
| 699 | GOBP\_CELLULAR\_RESPONSE\_TO\_ESTRADIOL\_STIMULUS |  | 36 | -0.37 | -1.33 | 0.087 | 0.331 | 1.000 | 1713 | tags=28%, list=13%, signal=32% |
| 700 | GOBP\_KIDNEY\_EPITHELIUM\_DEVELOPMENT |  | 121 | -0.29 | -1.33 | 0.046 | 0.330 | 1.000 | 2791 | tags=26%, list=22%, signal=32% |
| 701 | GOBP\_PHYSIOLOGICAL\_CARDIAC\_MUSCLE\_HYPERTROPHY |  | 20 | -0.44 | -1.33 | 0.111 | 0.330 | 1.000 | 1792 | tags=40%, list=14%, signal=46% |
| 702 | GOBP\_NEGATIVE\_REGULATION\_OF\_CELL\_CYCLE\_G1\_S\_PHASE\_TRANSITION |  | 68 | -0.32 | -1.33 | 0.075 | 0.331 | 1.000 | 3531 | tags=35%, list=27%, signal=48% |
| 703 | GOBP\_G\_PROTEIN\_COUPLED\_RECEPTOR\_SIGNALING\_PATHWAY\_COUPLED\_TO\_CYCLIC\_NUCLEOTIDE\_SECOND\_MESSENGER |  | 57 | -0.34 | -1.33 | 0.075 | 0.331 | 1.000 | 3630 | tags=39%, list=28%, signal=53% |
| 704 | GOBP\_VIRAL\_PROCESS |  | 372 | -0.26 | -1.33 | 0.006 | 0.332 | 1.000 | 3199 | tags=32%, list=25%, signal=41% |
| 705 | GOBP\_NUCLEAR\_TRANSCRIBED\_MRNA\_CATABOLIC\_PROCESS\_NONSENSE\_MEDIATED\_DECAY |  | 36 | -0.37 | -1.33 | 0.101 | 0.331 | 1.000 | 2712 | tags=39%, list=21%, signal=49% |
| 706 | GOBP\_INTERFERON\_BETA\_PRODUCTION |  | 50 | -0.35 | -1.33 | 0.077 | 0.331 | 1.000 | 2275 | tags=32%, list=18%, signal=39% |
| 707 | GOBP\_REGULATION\_OF\_KERATINOCYTE\_DIFFERENTIATION |  | 31 | -0.39 | -1.33 | 0.105 | 0.331 | 1.000 | 2254 | tags=32%, list=17%, signal=39% |
| 708 | GOBP\_REGULATION\_OF\_MYELOID\_CELL\_DIFFERENTIATION |  | 174 | -0.28 | -1.33 | 0.039 | 0.331 | 1.000 | 1944 | tags=22%, list=15%, signal=25% |
| 709 | GOBP\_SODIUM\_ION\_TRANSMEMBRANE\_TRANSPORT |  | 130 | -0.29 | -1.33 | 0.052 | 0.331 | 1.000 | 3029 | tags=29%, list=23%, signal=38% |
| 710 | GOBP\_GLUTAMINE\_METABOLIC\_PROCESS |  | 21 | -0.42 | -1.33 | 0.132 | 0.333 | 1.000 | 4664 | tags=52%, list=36%, signal=82% |
| 711 | GOBP\_CEREBELLAR\_CORTEX\_DEVELOPMENT |  | 44 | -0.36 | -1.33 | 0.082 | 0.333 | 1.000 | 2791 | tags=34%, list=22%, signal=43% |
| 712 | GOBP\_MICROTUBULE\_ORGANIZING\_CENTER\_LOCALIZATION |  | 27 | -0.40 | -1.33 | 0.125 | 0.334 | 1.000 | 3367 | tags=44%, list=26%, signal=60% |
| 713 | GOBP\_POSITIVE\_REGULATION\_OF\_ERYTHROCYTE\_DIFFERENTIATION |  | 29 | -0.39 | -1.33 | 0.116 | 0.334 | 1.000 | 1944 | tags=31%, list=15%, signal=36% |
| 714 | GOBP\_CELL\_SUBSTRATE\_ADHESION |  | 314 | -0.26 | -1.33 | 0.026 | 0.334 | 1.000 | 3845 | tags=37%, list=30%, signal=52% |
| 715 | GOBP\_STEROID\_HORMONE\_SECRETION |  | 20 | -0.44 | -1.33 | 0.123 | 0.334 | 1.000 | 2881 | tags=45%, list=22%, signal=58% |
| 716 | GOBP\_MALE\_SEX\_DIFFERENTIATION |  | 144 | -0.29 | -1.32 | 0.042 | 0.334 | 1.000 | 3966 | tags=36%, list=31%, signal=52% |
| 717 | GOBP\_DNA\_DEPENDENT\_DNA\_REPLICATION\_MAINTENANCE\_OF\_FIDELITY |  | 33 | -0.38 | -1.32 | 0.106 | 0.334 | 1.000 | 1876 | tags=33%, list=15%, signal=39% |
| 718 | GOBP\_BIOLOGICAL\_PROCESS\_INVOLVED\_IN\_INTERACTION\_WITH\_SYMBIONT |  | 86 | -0.31 | -1.32 | 0.075 | 0.333 | 1.000 | 4048 | tags=44%, list=31%, signal=64% |
| 719 | GOBP\_POSITIVE\_REGULATION\_OF\_INNATE\_IMMUNE\_RESPONSE |  | 110 | -0.30 | -1.32 | 0.042 | 0.333 | 1.000 | 3673 | tags=38%, list=28%, signal=53% |
| 720 | GOBP\_REGULATION\_OF\_LYASE\_ACTIVITY |  | 42 | -0.36 | -1.32 | 0.092 | 0.333 | 1.000 | 3951 | tags=48%, list=31%, signal=68% |
| 721 | GOBP\_IMMUNE\_RESPONSE\_TO\_TUMOR\_CELL |  | 21 | -0.43 | -1.32 | 0.138 | 0.333 | 1.000 | 2916 | tags=38%, list=23%, signal=49% |
| 722 | GOBP\_POSITIVE\_REGULATION\_OF\_CARTILAGE\_DEVELOPMENT |  | 27 | -0.40 | -1.32 | 0.117 | 0.333 | 1.000 | 2997 | tags=41%, list=23%, signal=53% |
| 723 | GOBP\_NEGATIVE\_REGULATION\_OF\_NATURAL\_KILLER\_CELL\_MEDIATED\_IMMUNITY |  | 18 | -0.44 | -1.32 | 0.139 | 0.334 | 1.000 | 2225 | tags=28%, list=17%, signal=34% |
| 724 | GOBP\_T\_CELL\_CYTOKINE\_PRODUCTION |  | 38 | -0.37 | -1.32 | 0.095 | 0.334 | 1.000 | 2101 | tags=29%, list=16%, signal=34% |
| 725 | GOBP\_PROTEIN\_LOCALIZATION\_TO\_NUCLEUS |  | 251 | -0.27 | -1.32 | 0.028 | 0.334 | 1.000 | 3149 | tags=33%, list=24%, signal=43% |
| 726 | GOBP\_DEVELOPMENTAL\_MATURATION |  | 239 | -0.27 | -1.32 | 0.018 | 0.334 | 1.000 | 2696 | tags=28%, list=21%, signal=34% |
| 727 | GOBP\_REGULATION\_OF\_HISTONE\_MODIFICATION |  | 152 | -0.29 | -1.32 | 0.045 | 0.334 | 1.000 | 2104 | tags=23%, list=16%, signal=27% |
| 728 | GOBP\_POSITIVE\_REGULATION\_OF\_STRESS\_ACTIVATED\_PROTEIN\_KINASE\_SIGNALING\_CASCADE |  | 112 | -0.30 | -1.32 | 0.049 | 0.335 | 1.000 | 2246 | tags=23%, list=17%, signal=28% |
| 729 | GOBP\_DNA\_REPLICATION |  | 228 | -0.27 | -1.32 | 0.018 | 0.335 | 1.000 | 2938 | tags=34%, list=23%, signal=44% |
| 730 | GOBP\_NEGATIVE\_REGULATION\_OF\_PROTEIN\_CATABOLIC\_PROCESS |  | 102 | -0.30 | -1.32 | 0.070 | 0.335 | 1.000 | 2926 | tags=32%, list=23%, signal=42% |
| 731 | GOBP\_MYELOID\_LEUKOCYTE\_DIFFERENTIATION |  | 182 | -0.28 | -1.32 | 0.027 | 0.335 | 1.000 | 2804 | tags=27%, list=22%, signal=34% |
| 732 | GOBP\_POSITIVE\_REGULATION\_OF\_NUCLEAR\_DIVISION |  | 47 | -0.35 | -1.32 | 0.085 | 0.337 | 1.000 | 2187 | tags=26%, list=17%, signal=31% |
| 733 | GOBP\_REGULATION\_OF\_CELLULAR\_RESPONSE\_TO\_GROWTH\_FACTOR\_STIMULUS |  | 225 | -0.27 | -1.32 | 0.027 | 0.338 | 1.000 | 2597 | tags=28%, list=20%, signal=34% |
| 734 | GOBP\_ANDROGEN\_METABOLIC\_PROCESS |  | 28 | -0.40 | -1.32 | 0.122 | 0.338 | 1.000 | 2753 | tags=36%, list=21%, signal=45% |
| 735 | GOBP\_FIBRINOLYSIS |  | 23 | -0.41 | -1.32 | 0.116 | 0.338 | 1.000 | 3040 | tags=43%, list=24%, signal=57% |
| 736 | GOBP\_RRNA\_TRANSCRIPTION |  | 32 | -0.38 | -1.32 | 0.129 | 0.338 | 1.000 | 4178 | tags=47%, list=32%, signal=69% |
| 737 | GOBP\_NEGATIVE\_REGULATION\_OF\_ENDOTHELIAL\_CELL\_APOPTOTIC\_PROCESS |  | 31 | -0.39 | -1.32 | 0.114 | 0.338 | 1.000 | 4279 | tags=55%, list=33%, signal=82% |
| 738 | GOBP\_POSITIVE\_REGULATION\_OF\_FOCAL\_ADHESION\_ASSEMBLY |  | 25 | -0.40 | -1.32 | 0.117 | 0.338 | 1.000 | 3821 | tags=48%, list=30%, signal=68% |
| 739 | GOBP\_T\_CELL\_DIFFERENTIATION |  | 225 | -0.27 | -1.32 | 0.031 | 0.337 | 1.000 | 2202 | tags=23%, list=17%, signal=27% |
| 740 | GOBP\_CARDIAC\_MUSCLE\_TISSUE\_DEVELOPMENT |  | 188 | -0.28 | -1.32 | 0.039 | 0.337 | 1.000 | 1983 | tags=21%, list=15%, signal=25% |
| 741 | GOBP\_CENTRAL\_NERVOUS\_SYSTEM\_NEURON\_AXONOGENESIS |  | 29 | -0.39 | -1.32 | 0.103 | 0.337 | 1.000 | 4067 | tags=55%, list=32%, signal=80% |
| 742 | GOBP\_CELLULAR\_RESPONSE\_TO\_OXYGEN\_LEVELS |  | 137 | -0.29 | -1.32 | 0.043 | 0.337 | 1.000 | 1668 | tags=21%, list=13%, signal=24% |
| 743 | GOBP\_METANEPHROS\_MORPHOGENESIS |  | 27 | -0.40 | -1.32 | 0.120 | 0.338 | 1.000 | 4147 | tags=41%, list=32%, signal=60% |
| 744 | GOBP\_NEGATIVE\_REGULATION\_OF\_AXON\_EXTENSION |  | 37 | -0.36 | -1.32 | 0.107 | 0.338 | 1.000 | 1917 | tags=30%, list=15%, signal=35% |
| 745 | GOBP\_CELLULAR\_COMPONENT\_DISASSEMBLY\_INVOLVED\_IN\_EXECUTION\_PHASE\_OF\_APOPTOSIS |  | 24 | -0.41 | -1.32 | 0.111 | 0.338 | 1.000 | 3043 | tags=38%, list=24%, signal=49% |
| 746 | GOBP\_CELLULAR\_RESPONSE\_TO\_LIPID |  | 498 | -0.25 | -1.32 | 0.005 | 0.339 | 1.000 | 1982 | tags=20%, list=15%, signal=23% |
| 747 | GOBP\_VASOCONSTRICTION |  | 77 | -0.32 | -1.32 | 0.070 | 0.339 | 1.000 | 4933 | tags=48%, list=38%, signal=77% |
| 748 | GOBP\_NEGATIVE\_REGULATION\_OF\_RHO\_PROTEIN\_SIGNAL\_TRANSDUCTION |  | 18 | -0.45 | -1.32 | 0.143 | 0.339 | 1.000 | 2204 | tags=33%, list=17%, signal=40% |
| 749 | GOBP\_VASCULAR\_ASSOCIATED\_SMOOTH\_MUSCLE\_CELL\_DIFFERENTIATION |  | 24 | -0.42 | -1.32 | 0.138 | 0.339 | 1.000 | 513 | tags=25%, list=4%, signal=26% |
| 750 | GOBP\_T\_HELPER\_2\_CELL\_DIFFERENTIATION |  | 15 | -0.46 | -1.31 | 0.143 | 0.340 | 1.000 | 2819 | tags=53%, list=22%, signal=68% |
| 751 | GOBP\_POSITIVE\_REGULATION\_OF\_CELLULAR\_CARBOHYDRATE\_METABOLIC\_PROCESS |  | 48 | -0.34 | -1.31 | 0.090 | 0.340 | 1.000 | 3103 | tags=33%, list=24%, signal=44% |
| 752 | GOBP\_CELL\_JUNCTION\_ASSEMBLY |  | 336 | -0.26 | -1.31 | 0.005 | 0.340 | 1.000 | 3205 | tags=27%, list=25%, signal=35% |
| 753 | GOBP\_KIDNEY\_MORPHOGENESIS |  | 76 | -0.31 | -1.31 | 0.079 | 0.339 | 1.000 | 4147 | tags=41%, list=32%, signal=60% |
| 754 | GOBP\_SIGNAL\_TRANSDUCTION\_IN\_ABSENCE\_OF\_LIGAND |  | 60 | -0.33 | -1.31 | 0.088 | 0.339 | 1.000 | 2834 | tags=35%, list=22%, signal=45% |
| 755 | GOBP\_NEGATIVE\_REGULATION\_OF\_TYPE\_I\_INTERFERON\_PRODUCTION |  | 25 | -0.40 | -1.31 | 0.133 | 0.339 | 1.000 | 4047 | tags=48%, list=31%, signal=70% |
| 756 | GOBP\_REGULATION\_OF\_POSTSYNAPTIC\_MEMBRANE\_NEUROTRANSMITTER\_RECEPTOR\_LEVELS |  | 44 | -0.35 | -1.31 | 0.109 | 0.341 | 1.000 | 2604 | tags=34%, list=20%, signal=43% |
| 757 | GOBP\_N\_TERMINAL\_PROTEIN\_AMINO\_ACID\_MODIFICATION |  | 23 | -0.40 | -1.31 | 0.137 | 0.345 | 1.000 | 2930 | tags=39%, list=23%, signal=51% |
| 758 | GOBP\_EMBRYO\_IMPLANTATION |  | 48 | -0.35 | -1.31 | 0.091 | 0.345 | 1.000 | 1884 | tags=25%, list=15%, signal=29% |
| 759 | GOBP\_DIGESTION |  | 105 | -0.30 | -1.31 | 0.058 | 0.345 | 1.000 | 4020 | tags=37%, list=31%, signal=54% |
| 760 | GOBP\_POSITIVE\_REGULATION\_OF\_PROTEIN\_ACETYLATION |  | 42 | -0.36 | -1.31 | 0.092 | 0.347 | 1.000 | 1683 | tags=29%, list=13%, signal=33% |
| 761 | GOBP\_MIRNA\_METABOLIC\_PROCESS |  | 17 | -0.44 | -1.31 | 0.139 | 0.347 | 1.000 | 2525 | tags=41%, list=20%, signal=51% |
| 762 | GOBP\_MONOCYTE\_CHEMOTAXIS |  | 58 | -0.33 | -1.31 | 0.102 | 0.347 | 1.000 | 3548 | tags=45%, list=28%, signal=62% |
| 763 | GOBP\_NEGATIVE\_REGULATION\_OF\_CELLULAR\_RESPONSE\_TO\_INSULIN\_STIMULUS |  | 32 | -0.38 | -1.31 | 0.111 | 0.347 | 1.000 | 1632 | tags=31%, list=13%, signal=36% |
| 764 | GOBP\_T\_CELL\_MIGRATION |  | 54 | -0.34 | -1.31 | 0.081 | 0.348 | 1.000 | 3162 | tags=43%, list=25%, signal=56% |
| 765 | GOBP\_NEGATIVE\_REGULATION\_OF\_NEURON\_DEATH |  | 177 | -0.28 | -1.31 | 0.050 | 0.348 | 1.000 | 2561 | tags=25%, list=20%, signal=31% |
| 766 | GOBP\_POSITIVE\_REGULATION\_OF\_GLUCOSE\_TRANSMEMBRANE\_TRANSPORT |  | 38 | -0.36 | -1.31 | 0.106 | 0.348 | 1.000 | 3267 | tags=39%, list=25%, signal=53% |
| 767 | GOBP\_EXTRINSIC\_APOPTOTIC\_SIGNALING\_PATHWAY\_VIA\_DEATH\_DOMAIN\_RECEPTORS |  | 72 | -0.33 | -1.31 | 0.070 | 0.348 | 1.000 | 4022 | tags=46%, list=31%, signal=66% |
| 768 | GOBP\_REGULATION\_OF\_CELL\_DEVELOPMENT |  | 419 | -0.25 | -1.31 | 0.023 | 0.348 | 1.000 | 1983 | tags=19%, list=15%, signal=22% |
| 769 | GOBP\_MESONEPHROS\_DEVELOPMENT |  | 86 | -0.31 | -1.31 | 0.069 | 0.349 | 1.000 | 864 | tags=14%, list=7%, signal=15% |
| 770 | GOBP\_RESPONSE\_TO\_X\_RAY |  | 28 | -0.40 | -1.31 | 0.117 | 0.350 | 1.000 | 993 | tags=25%, list=8%, signal=27% |
| 771 | GOBP\_POSITIVE\_REGULATION\_OF\_CELL\_CYCLE\_G1\_S\_PHASE\_TRANSITION |  | 41 | -0.36 | -1.31 | 0.117 | 0.350 | 1.000 | 1174 | tags=22%, list=9%, signal=24% |
| 772 | GOBP\_NEGATIVE\_REGULATION\_OF\_SMALL\_GTPASE\_MEDIATED\_SIGNAL\_TRANSDUCTION |  | 47 | -0.35 | -1.31 | 0.094 | 0.350 | 1.000 | 4067 | tags=45%, list=32%, signal=65% |
| 773 | GOBP\_MUSCLE\_HYPERTROPHY |  | 73 | -0.32 | -1.30 | 0.076 | 0.352 | 1.000 | 1966 | tags=27%, list=15%, signal=32% |
| 774 | GOBP\_GLIOGENESIS |  | 249 | -0.27 | -1.30 | 0.027 | 0.352 | 1.000 | 2798 | tags=28%, list=22%, signal=35% |
| 775 | GOBP\_NEGATIVE\_REGULATION\_OF\_CELL\_AGING |  | 23 | -0.41 | -1.30 | 0.147 | 0.352 | 1.000 | 5126 | tags=70%, list=40%, signal=115% |
| 776 | GOBP\_POSITIVE\_REGULATION\_OF\_EPITHELIAL\_CELL\_APOPTOTIC\_PROCESS |  | 27 | -0.39 | -1.30 | 0.144 | 0.353 | 1.000 | 3083 | tags=37%, list=24%, signal=49% |
| 777 | GOBP\_REGULATION\_OF\_INSULIN\_RECEPTOR\_SIGNALING\_PATHWAY |  | 54 | -0.33 | -1.30 | 0.096 | 0.353 | 1.000 | 1803 | tags=28%, list=14%, signal=32% |
| 778 | GOBP\_REGULATION\_OF\_GLUCOSE\_METABOLIC\_PROCESS |  | 99 | -0.30 | -1.30 | 0.083 | 0.352 | 1.000 | 2072 | tags=25%, list=16%, signal=30% |
| 779 | GOBP\_STRESS\_ACTIVATED\_PROTEIN\_KINASE\_SIGNALING\_CASCADE |  | 218 | -0.27 | -1.30 | 0.036 | 0.352 | 1.000 | 1427 | tags=17%, list=11%, signal=19% |
| 780 | GOBP\_HEMATOPOIETIC\_STEM\_CELL\_DIFFERENTIATION |  | 27 | -0.39 | -1.30 | 0.126 | 0.352 | 1.000 | 3977 | tags=48%, list=31%, signal=69% |
| 781 | GOBP\_POSITIVE\_REGULATION\_OF\_LIPID\_BIOSYNTHETIC\_PROCESS |  | 69 | -0.32 | -1.30 | 0.081 | 0.353 | 1.000 | 2078 | tags=25%, list=16%, signal=29% |
| 782 | GOBP\_POSITIVE\_REGULATION\_OF\_MUSCLE\_CELL\_APOPTOTIC\_PROCESS |  | 23 | -0.41 | -1.30 | 0.137 | 0.353 | 1.000 | 1979 | tags=22%, list=15%, signal=26% |
| 783 | GOBP\_SENSORY\_ORGAN\_DEVELOPMENT |  | 444 | -0.25 | -1.30 | 0.017 | 0.353 | 1.000 | 3763 | tags=33%, list=29%, signal=45% |
| 784 | GOBP\_CEREBELLAR\_PURKINJE\_CELL\_LAYER\_DEVELOPMENT |  | 25 | -0.39 | -1.30 | 0.151 | 0.353 | 1.000 | 2616 | tags=36%, list=20%, signal=45% |
| 785 | GOBP\_PROTEIN\_EXPORT\_FROM\_NUCLEUS |  | 48 | -0.35 | -1.30 | 0.109 | 0.354 | 1.000 | 4169 | tags=48%, list=32%, signal=71% |
| 786 | GOBP\_RESPONSE\_TO\_ENDOPLASMIC\_RETICULUM\_STRESS |  | 199 | -0.27 | -1.30 | 0.053 | 0.354 | 1.000 | 3963 | tags=37%, list=31%, signal=53% |
| 787 | GOBP\_NEGATIVE\_REGULATION\_OF\_BONE\_REMODELING |  | 16 | -0.45 | -1.30 | 0.151 | 0.353 | 1.000 | 1168 | tags=38%, list=9%, signal=41% |
| 788 | GOBP\_POSITIVE\_REGULATION\_OF\_G1\_S\_TRANSITION\_OF\_MITOTIC\_CELL\_CYCLE |  | 31 | -0.38 | -1.30 | 0.117 | 0.354 | 1.000 | 972 | tags=23%, list=8%, signal=24% |
| 789 | GOBP\_REGULATION\_OF\_BROWN\_FAT\_CELL\_DIFFERENTIATION |  | 17 | -0.45 | -1.30 | 0.155 | 0.353 | 1.000 | 564 | tags=24%, list=4%, signal=25% |
| 790 | GOBP\_HISTONE\_H3\_K4\_TRIMETHYLATION |  | 19 | -0.42 | -1.30 | 0.148 | 0.353 | 1.000 | 2940 | tags=42%, list=23%, signal=54% |
| 791 | GOBP\_POSITIVE\_REGULATION\_OF\_MYOBLAST\_DIFFERENTIATION |  | 17 | -0.43 | -1.30 | 0.148 | 0.353 | 1.000 | 2338 | tags=35%, list=18%, signal=43% |
| 792 | GOBP\_CELL\_CYCLE\_PHASE\_TRANSITION |  | 407 | -0.25 | -1.30 | 0.013 | 0.353 | 1.000 | 3204 | tags=28%, list=25%, signal=36% |
| 793 | GOBP\_MUSCLE\_CELL\_MIGRATION |  | 76 | -0.31 | -1.30 | 0.081 | 0.354 | 1.000 | 2791 | tags=30%, list=22%, signal=38% |
| 794 | GOBP\_CELL\_GROWTH |  | 410 | -0.25 | -1.30 | 0.017 | 0.354 | 1.000 | 1951 | tags=21%, list=15%, signal=24% |
| 795 | GOBP\_REGULATION\_OF\_METAL\_ION\_TRANSPORT |  | 311 | -0.25 | -1.30 | 0.033 | 0.354 | 1.000 | 3118 | tags=29%, list=24%, signal=37% |
| 796 | GOBP\_POSITIVE\_REGULATION\_OF\_ACTIN\_FILAMENT\_POLYMERIZATION |  | 41 | -0.35 | -1.30 | 0.113 | 0.354 | 1.000 | 3465 | tags=44%, list=27%, signal=60% |
| 797 | GOBP\_POTASSIUM\_ION\_IMPORT\_ACROSS\_PLASMA\_MEMBRANE |  | 41 | -0.35 | -1.30 | 0.096 | 0.353 | 1.000 | 4045 | tags=46%, list=31%, signal=67% |
| 798 | GOBP\_DOPAMINERGIC\_NEURON\_DIFFERENTIATION |  | 28 | -0.38 | -1.30 | 0.109 | 0.353 | 1.000 | 2196 | tags=32%, list=17%, signal=39% |
| 799 | GOBP\_POSITIVE\_REGULATION\_OF\_AXON\_EXTENSION |  | 36 | -0.37 | -1.30 | 0.113 | 0.353 | 1.000 | 3936 | tags=44%, list=31%, signal=64% |
| 800 | GOBP\_NEGATIVE\_REGULATION\_OF\_VASCULATURE\_DEVELOPMENT |  | 93 | -0.30 | -1.30 | 0.063 | 0.353 | 1.000 | 4429 | tags=48%, list=34%, signal=73% |
| 801 | GOBP\_POSITIVE\_REGULATION\_OF\_JNK\_CASCADE |  | 81 | -0.31 | -1.30 | 0.078 | 0.353 | 1.000 | 2567 | tags=27%, list=20%, signal=34% |
| 802 | GOBP\_POSITIVE\_REGULATION\_OF\_CELL\_CYCLE\_PHASE\_TRANSITION |  | 85 | -0.30 | -1.30 | 0.075 | 0.353 | 1.000 | 1174 | tags=18%, list=9%, signal=19% |
| 803 | GOBP\_MATURATION\_OF\_LSU\_RRNA |  | 24 | -0.40 | -1.30 | 0.149 | 0.353 | 1.000 | 3127 | tags=46%, list=24%, signal=60% |
| 804 | GOBP\_REGULATION\_OF\_SODIUM\_ION\_TRANSPORT |  | 67 | -0.32 | -1.30 | 0.092 | 0.353 | 1.000 | 2608 | tags=31%, list=20%, signal=39% |
| 805 | GOBP\_NIK\_NF\_KAPPAB\_SIGNALING |  | 111 | -0.29 | -1.30 | 0.077 | 0.354 | 1.000 | 2430 | tags=28%, list=19%, signal=34% |
| 806 | GOBP\_POSITIVE\_REGULATION\_OF\_NEURON\_APOPTOTIC\_PROCESS |  | 48 | -0.35 | -1.30 | 0.090 | 0.354 | 1.000 | 2396 | tags=27%, list=19%, signal=33% |
| 807 | GOBP\_LABYRINTHINE\_LAYER\_MORPHOGENESIS |  | 21 | -0.41 | -1.30 | 0.151 | 0.355 | 1.000 | 4047 | tags=43%, list=31%, signal=62% |
| 808 | GOBP\_CELLULAR\_RESPONSE\_TO\_ALCOHOL |  | 76 | -0.31 | -1.30 | 0.080 | 0.356 | 1.000 | 3250 | tags=37%, list=25%, signal=49% |
| 809 | GOBP\_ESTABLISHMENT\_OR\_MAINTENANCE\_OF\_BIPOLAR\_CELL\_POLARITY |  | 42 | -0.35 | -1.29 | 0.116 | 0.357 | 1.000 | 3538 | tags=40%, list=27%, signal=56% |
| 810 | GOBP\_POSITIVE\_REGULATION\_OF\_PROTEIN\_POLYMERIZATION |  | 74 | -0.31 | -1.29 | 0.089 | 0.357 | 1.000 | 4339 | tags=47%, list=34%, signal=71% |
| 811 | GOBP\_T\_CELL\_SELECTION |  | 43 | -0.35 | -1.29 | 0.104 | 0.357 | 1.000 | 2085 | tags=26%, list=16%, signal=30% |
| 812 | GOBP\_CARDIAC\_CONDUCTION\_SYSTEM\_DEVELOPMENT |  | 32 | -0.37 | -1.29 | 0.114 | 0.356 | 1.000 | 3904 | tags=44%, list=30%, signal=63% |
| 813 | GOBP\_NEGATIVE\_REGULATION\_OF\_GROWTH |  | 211 | -0.27 | -1.29 | 0.044 | 0.356 | 1.000 | 3439 | tags=34%, list=27%, signal=46% |
| 814 | GOBP\_ER\_NUCLEUS\_SIGNALING\_PATHWAY |  | 40 | -0.35 | -1.29 | 0.114 | 0.357 | 1.000 | 4222 | tags=58%, list=33%, signal=85% |
| 815 | GOBP\_CELLULAR\_RESPONSE\_TO\_PEPTIDE |  | 312 | -0.25 | -1.29 | 0.015 | 0.358 | 1.000 | 2810 | tags=28%, list=22%, signal=34% |
| 816 | GOBP\_POLY\_A\_PLUS\_MRNA\_EXPORT\_FROM\_NUCLEUS |  | 17 | -0.44 | -1.29 | 0.163 | 0.358 | 1.000 | 1122 | tags=29%, list=9%, signal=32% |
| 817 | GOBP\_CELLULAR\_RESPONSE\_TO\_THYROID\_HORMONE\_STIMULUS |  | 17 | -0.44 | -1.29 | 0.151 | 0.357 | 1.000 | 3082 | tags=53%, list=24%, signal=69% |
| 818 | GOBP\_POSITIVE\_REGULATION\_OF\_MYELOID\_CELL\_DIFFERENTIATION |  | 85 | -0.30 | -1.29 | 0.074 | 0.357 | 1.000 | 2460 | tags=27%, list=19%, signal=33% |
| 819 | GOBP\_POSITIVE\_REGULATION\_OF\_TRANSCRIPTION\_BY\_RNA\_POLYMERASE\_III |  | 16 | -0.45 | -1.29 | 0.156 | 0.357 | 1.000 | 5304 | tags=81%, list=41%, signal=138% |
| 820 | GOBP\_REGULATION\_OF\_RESPONSE\_TO\_CYTOKINE\_STIMULUS |  | 132 | -0.28 | -1.29 | 0.053 | 0.358 | 1.000 | 3731 | tags=37%, list=29%, signal=52% |
| 821 | GOBP\_COLUMNAR\_CUBOIDAL\_EPITHELIAL\_CELL\_DIFFERENTIATION |  | 76 | -0.31 | -1.29 | 0.077 | 0.358 | 1.000 | 2881 | tags=32%, list=22%, signal=40% |
| 822 | GOBP\_ARTERY\_DEVELOPMENT |  | 87 | -0.30 | -1.29 | 0.086 | 0.357 | 1.000 | 2259 | tags=28%, list=18%, signal=33% |
| 823 | GOBP\_NEURON\_PROJECTION\_REGENERATION |  | 50 | -0.34 | -1.29 | 0.124 | 0.358 | 1.000 | 3104 | tags=38%, list=24%, signal=50% |
| 824 | GOBP\_LEUKOCYTE\_MIGRATION |  | 333 | -0.26 | -1.29 | 0.018 | 0.359 | 1.000 | 3551 | tags=34%, list=28%, signal=46% |
| 825 | GOBP\_PIGMENT\_CELL\_DIFFERENTIATION |  | 30 | -0.38 | -1.29 | 0.149 | 0.360 | 1.000 | 2052 | tags=30%, list=16%, signal=36% |
| 826 | GOBP\_CHONDROCYTE\_PROLIFERATION |  | 21 | -0.42 | -1.29 | 0.131 | 0.360 | 1.000 | 2016 | tags=29%, list=16%, signal=34% |
| 827 | GOBP\_NEGATIVE\_REGULATION\_OF\_CELL\_CYCLE\_PHASE\_TRANSITION |  | 194 | -0.27 | -1.29 | 0.041 | 0.361 | 1.000 | 3204 | tags=28%, list=25%, signal=37% |
| 828 | GOBP\_RESPONSE\_TO\_MOLECULE\_OF\_BACTERIAL\_ORIGIN |  | 296 | -0.26 | -1.29 | 0.028 | 0.361 | 1.000 | 2023 | tags=20%, list=16%, signal=23% |
| 829 | GOBP\_POSITIVE\_REGULATION\_OF\_LYASE\_ACTIVITY |  | 19 | -0.43 | -1.29 | 0.148 | 0.361 | 1.000 | 3708 | tags=58%, list=29%, signal=81% |
| 830 | GOBP\_DNA\_INTEGRITY\_CHECKPOINT\_SIGNALING |  | 101 | -0.30 | -1.29 | 0.063 | 0.362 | 1.000 | 1833 | tags=21%, list=14%, signal=24% |
| 831 | GOBP\_REGULATION\_OF\_DNA\_BINDING |  | 109 | -0.29 | -1.29 | 0.086 | 0.363 | 1.000 | 2493 | tags=26%, list=19%, signal=32% |
| 832 | GOBP\_RESPONSE\_TO\_STEROID\_HORMONE |  | 299 | -0.25 | -1.29 | 0.030 | 0.363 | 1.000 | 1931 | tags=20%, list=15%, signal=23% |
| 833 | GOBP\_MUSCLE\_ADAPTATION |  | 89 | -0.30 | -1.29 | 0.086 | 0.363 | 1.000 | 1966 | tags=27%, list=15%, signal=32% |
| 834 | GOBP\_ODONTOGENESIS |  | 106 | -0.29 | -1.29 | 0.083 | 0.363 | 1.000 | 4391 | tags=42%, list=34%, signal=64% |
| 835 | GOBP\_POSITIVE\_REGULATION\_OF\_CELL\_DEVELOPMENT |  | 257 | -0.26 | -1.29 | 0.037 | 0.363 | 1.000 | 3626 | tags=32%, list=28%, signal=43% |
| 836 | GOBP\_REGULATION\_OF\_NERVOUS\_SYSTEM\_DEVELOPMENT |  | 357 | -0.25 | -1.29 | 0.028 | 0.363 | 1.000 | 1983 | tags=19%, list=15%, signal=22% |
| 837 | GOBP\_RESPONSE\_TO\_ESTRADIOL |  | 114 | -0.29 | -1.29 | 0.065 | 0.362 | 1.000 | 1445 | tags=18%, list=11%, signal=21% |
| 838 | GOBP\_POSITIVE\_REGULATION\_OF\_CYTOKINE\_PRODUCTION\_INVOLVED\_IN\_IMMUNE\_RESPONSE |  | 63 | -0.32 | -1.29 | 0.103 | 0.363 | 1.000 | 797 | tags=16%, list=6%, signal=17% |
| 839 | GOBP\_RESPONSE\_TO\_MURAMYL\_DIPEPTIDE |  | 19 | -0.42 | -1.28 | 0.155 | 0.364 | 1.000 | 367 | tags=26%, list=3%, signal=27% |
| 840 | GOBP\_POSITIVE\_REGULATION\_OF\_CELL\_SUBSTRATE\_ADHESION |  | 107 | -0.29 | -1.28 | 0.098 | 0.363 | 1.000 | 1981 | tags=22%, list=15%, signal=26% |
| 841 | GOBP\_NEGATIVE\_REGULATION\_OF\_MAPK\_CASCADE |  | 140 | -0.28 | -1.28 | 0.079 | 0.363 | 1.000 | 1416 | tags=19%, list=11%, signal=21% |
| 842 | GOBP\_POSITIVE\_REGULATION\_OF\_SMOOTH\_MUSCLE\_CELL\_MIGRATION |  | 32 | -0.37 | -1.28 | 0.128 | 0.364 | 1.000 | 1230 | tags=19%, list=10%, signal=21% |
| 843 | GOBP\_POSITIVE\_REGULATION\_OF\_ENDOTHELIAL\_CELL\_PROLIFERATION |  | 84 | -0.31 | -1.28 | 0.091 | 0.364 | 1.000 | 1983 | tags=25%, list=15%, signal=29% |
| 844 | GOBP\_SODIUM\_ION\_HOMEOSTASIS |  | 53 | -0.34 | -1.28 | 0.115 | 0.364 | 1.000 | 3303 | tags=38%, list=26%, signal=51% |
| 845 | GOBP\_CANONICAL\_WNT\_SIGNALING\_PATHWAY |  | 235 | -0.26 | -1.28 | 0.044 | 0.365 | 1.000 | 2246 | tags=24%, list=17%, signal=29% |
| 846 | GOBP\_NEURON\_PROJECTION\_ORGANIZATION |  | 75 | -0.31 | -1.28 | 0.105 | 0.364 | 1.000 | 3215 | tags=33%, list=25%, signal=44% |
| 847 | GOBP\_NEGATIVE\_REGULATION\_OF\_RESPONSE\_TO\_WOUNDING |  | 75 | -0.31 | -1.28 | 0.090 | 0.364 | 1.000 | 3219 | tags=36%, list=25%, signal=48% |
| 848 | GOBP\_MONONUCLEAR\_CELL\_MIGRATION |  | 167 | -0.27 | -1.28 | 0.056 | 0.364 | 1.000 | 3435 | tags=36%, list=27%, signal=48% |
| 849 | GOBP\_GASTRIC\_ACID\_SECRETION |  | 15 | -0.46 | -1.28 | 0.161 | 0.364 | 1.000 | 2829 | tags=47%, list=22%, signal=60% |
| 850 | GOBP\_THYMOCYTE\_APOPTOTIC\_PROCESS |  | 15 | -0.45 | -1.28 | 0.173 | 0.364 | 1.000 | 3636 | tags=53%, list=28%, signal=74% |
| 851 | GOBP\_NEGATIVE\_REGULATION\_OF\_TOLL\_LIKE\_RECEPTOR\_SIGNALING\_PATHWAY |  | 26 | -0.39 | -1.28 | 0.138 | 0.364 | 1.000 | 1961 | tags=31%, list=15%, signal=36% |
| 852 | GOBP\_REGULATION\_OF\_CELL\_SHAPE |  | 126 | -0.29 | -1.28 | 0.084 | 0.364 | 1.000 | 3240 | tags=32%, list=25%, signal=42% |
| 853 | GOBP\_PHOSPHATIDYLCHOLINE\_BIOSYNTHETIC\_PROCESS |  | 23 | -0.41 | -1.28 | 0.147 | 0.363 | 1.000 | 4788 | tags=61%, list=37%, signal=97% |
| 854 | GOBP\_REGULATION\_OF\_CARBOHYDRATE\_METABOLIC\_PROCESS |  | 156 | -0.27 | -1.28 | 0.057 | 0.365 | 1.000 | 2084 | tags=23%, list=16%, signal=27% |
| 855 | GOBP\_HEART\_MORPHOGENESIS |  | 212 | -0.26 | -1.28 | 0.037 | 0.366 | 1.000 | 2059 | tags=20%, list=16%, signal=23% |
| 856 | GOBP\_LYMPHOCYTE\_MIGRATION |  | 96 | -0.30 | -1.28 | 0.080 | 0.366 | 1.000 | 4069 | tags=46%, list=32%, signal=66% |
| 857 | GOBP\_NEGATIVE\_REGULATION\_OF\_CELLULAR\_RESPONSE\_TO\_GROWTH\_FACTOR\_STIMULUS |  | 79 | -0.31 | -1.28 | 0.087 | 0.366 | 1.000 | 2587 | tags=29%, list=20%, signal=36% |
| 858 | GOBP\_REGULATION\_OF\_SYSTEM\_PROCESS |  | 469 | -0.24 | -1.28 | 0.016 | 0.366 | 1.000 | 3118 | tags=27%, list=24%, signal=35% |
| 859 | GOBP\_POSITIVE\_REGULATION\_OF\_HOMOTYPIC\_CELL\_CELL\_ADHESION |  | 15 | -0.45 | -1.28 | 0.175 | 0.366 | 1.000 | 3691 | tags=47%, list=29%, signal=65% |
| 860 | GOBP\_REGULATION\_OF\_JNK\_CASCADE |  | 120 | -0.29 | -1.28 | 0.067 | 0.365 | 1.000 | 1284 | tags=18%, list=10%, signal=20% |
| 861 | GOBP\_REGULATION\_OF\_DENDRITIC\_SPINE\_MORPHOGENESIS |  | 33 | -0.37 | -1.28 | 0.133 | 0.365 | 1.000 | 4145 | tags=48%, list=32%, signal=71% |
| 862 | GOBP\_NEGATIVE\_REGULATION\_OF\_HISTONE\_MODIFICATION |  | 41 | -0.36 | -1.28 | 0.130 | 0.366 | 1.000 | 2862 | tags=34%, list=22%, signal=44% |
| 863 | GOBP\_RESPONSE\_TO\_MUSCLE\_STRETCH |  | 24 | -0.40 | -1.28 | 0.152 | 0.366 | 1.000 | 1626 | tags=25%, list=13%, signal=29% |
| 864 | GOBP\_NEGATIVE\_REGULATION\_OF\_RESPONSE\_TO\_EXTERNAL\_STIMULUS |  | 333 | -0.25 | -1.28 | 0.025 | 0.366 | 1.000 | 3237 | tags=29%, list=25%, signal=38% |
| 865 | GOBP\_REGULATION\_OF\_MACROPHAGE\_ACTIVATION |  | 50 | -0.33 | -1.28 | 0.108 | 0.367 | 1.000 | 2000 | tags=28%, list=16%, signal=33% |
| 866 | GOBP\_REGULATION\_OF\_EPITHELIAL\_CELL\_MIGRATION |  | 210 | -0.27 | -1.28 | 0.043 | 0.368 | 1.000 | 2996 | tags=30%, list=23%, signal=38% |
| 867 | GOBP\_NEGATIVE\_REGULATION\_OF\_TISSUE\_REMODELING |  | 20 | -0.41 | -1.28 | 0.150 | 0.368 | 1.000 | 1168 | tags=30%, list=9%, signal=33% |
| 868 | GOBP\_REGULATION\_OF\_RECEPTOR\_MEDIATED\_ENDOCYTOSIS |  | 91 | -0.30 | -1.28 | 0.086 | 0.369 | 1.000 | 2618 | tags=32%, list=20%, signal=40% |
| 869 | GOBP\_CARDIAC\_CELL\_DEVELOPMENT |  | 69 | -0.31 | -1.28 | 0.095 | 0.369 | 1.000 | 2103 | tags=26%, list=16%, signal=31% |
| 870 | GOBP\_HEMATOPOIETIC\_STEM\_CELL\_PROLIFERATION |  | 21 | -0.41 | -1.28 | 0.160 | 0.369 | 1.000 | 2196 | tags=33%, list=17%, signal=40% |
| 871 | GOBP\_LINOLEIC\_ACID\_METABOLIC\_PROCESS |  | 16 | -0.45 | -1.28 | 0.174 | 0.369 | 1.000 | 4823 | tags=75%, list=37%, signal=120% |
| 872 | GOBP\_POSITIVE\_REGULATION\_OF\_ESTABLISHMENT\_OF\_PROTEIN\_LOCALIZATION\_TO\_MITOCHONDRION |  | 33 | -0.37 | -1.28 | 0.154 | 0.369 | 1.000 | 2832 | tags=36%, list=22%, signal=46% |
| 873 | GOBP\_ACUTE\_INFLAMMATORY\_RESPONSE |  | 99 | -0.30 | -1.28 | 0.095 | 0.369 | 1.000 | 3133 | tags=31%, list=24%, signal=41% |
| 874 | GOBP\_SYMPATHETIC\_NERVOUS\_SYSTEM\_DEVELOPMENT |  | 21 | -0.42 | -1.27 | 0.147 | 0.370 | 1.000 | 3723 | tags=48%, list=29%, signal=67% |
| 875 | GOBP\_POSITIVE\_REGULATION\_OF\_TELOMERE\_MAINTENANCE |  | 45 | -0.34 | -1.27 | 0.122 | 0.371 | 1.000 | 2311 | tags=24%, list=18%, signal=30% |
| 876 | GOBP\_VASCULAR\_ENDOTHELIAL\_GROWTH\_FACTOR\_RECEPTOR\_SIGNALING\_PATHWAY |  | 47 | -0.34 | -1.27 | 0.132 | 0.372 | 1.000 | 2810 | tags=38%, list=22%, signal=49% |
| 877 | GOBP\_SMOOTH\_MUSCLE\_CELL\_MIGRATION |  | 65 | -0.32 | -1.27 | 0.106 | 0.371 | 1.000 | 2678 | tags=28%, list=21%, signal=35% |
| 878 | GOBP\_AXON\_DEVELOPMENT |  | 395 | -0.25 | -1.27 | 0.028 | 0.371 | 1.000 | 2842 | tags=26%, list=22%, signal=32% |
| 879 | GOBP\_REGULATION\_OF\_GTPASE\_ACTIVITY |  | 276 | -0.25 | -1.27 | 0.034 | 0.372 | 1.000 | 3406 | tags=32%, list=26%, signal=42% |
| 880 | GOBP\_IRE1\_MEDIATED\_UNFOLDED\_PROTEIN\_RESPONSE |  | 17 | -0.44 | -1.27 | 0.160 | 0.371 | 1.000 | 4073 | tags=47%, list=32%, signal=69% |
| 881 | GOBP\_SKIN\_EPIDERMIS\_DEVELOPMENT |  | 72 | -0.31 | -1.27 | 0.116 | 0.371 | 1.000 | 1955 | tags=21%, list=15%, signal=24% |
| 882 | GOBP\_POSITIVE\_REGULATION\_OF\_DNA\_METABOLIC\_PROCESS |  | 193 | -0.27 | -1.27 | 0.053 | 0.371 | 1.000 | 2469 | tags=24%, list=19%, signal=29% |
| 883 | GOBP\_CEREBELLAR\_PURKINJE\_CELL\_LAYER\_MORPHOGENESIS |  | 17 | -0.44 | -1.27 | 0.162 | 0.371 | 1.000 | 3242 | tags=41%, list=25%, signal=55% |
| 884 | GOBP\_EMBRYONIC\_HEART\_TUBE\_DEVELOPMENT |  | 60 | -0.32 | -1.27 | 0.105 | 0.371 | 1.000 | 3027 | tags=32%, list=23%, signal=41% |
| 885 | GOBP\_SOMITOGENESIS |  | 50 | -0.33 | -1.27 | 0.115 | 0.371 | 1.000 | 4102 | tags=40%, list=32%, signal=58% |
| 886 | GOBP\_CYCLIC\_NUCLEOTIDE\_MEDIATED\_SIGNALING |  | 71 | -0.31 | -1.27 | 0.107 | 0.371 | 1.000 | 2188 | tags=27%, list=17%, signal=32% |
| 887 | GOBP\_NEGATIVE\_REGULATION\_OF\_EXTRINSIC\_APOPTOTIC\_SIGNALING\_PATHWAY |  | 83 | -0.31 | -1.27 | 0.096 | 0.371 | 1.000 | 3242 | tags=33%, list=25%, signal=43% |
| 888 | GOBP\_ADAPTATION\_OF\_SIGNALING\_PATHWAY |  | 21 | -0.41 | -1.27 | 0.145 | 0.372 | 1.000 | 2247 | tags=38%, list=17%, signal=46% |
| 889 | GOBP\_AORTA\_DEVELOPMENT |  | 52 | -0.33 | -1.27 | 0.124 | 0.373 | 1.000 | 4162 | tags=48%, list=32%, signal=71% |
| 890 | GOBP\_CELLULAR\_RESPONSE\_TO\_FLUID\_SHEAR\_STRESS |  | 19 | -0.42 | -1.27 | 0.152 | 0.373 | 1.000 | 2832 | tags=42%, list=22%, signal=54% |
| 891 | GOBP\_POSITIVE\_REGULATION\_OF\_SMOOTH\_MUSCLE\_CONTRACTION |  | 26 | -0.38 | -1.27 | 0.165 | 0.373 | 1.000 | 1934 | tags=23%, list=15%, signal=27% |
| 892 | GOBP\_NEGATIVE\_REGULATION\_OF\_OSSIFICATION |  | 31 | -0.37 | -1.27 | 0.180 | 0.373 | 1.000 | 2069 | tags=29%, list=16%, signal=34% |
| 893 | GOBP\_POSITIVE\_REGULATION\_OF\_CELL\_SUBSTRATE\_JUNCTION\_ORGANIZATION |  | 30 | -0.37 | -1.27 | 0.145 | 0.373 | 1.000 | 3821 | tags=47%, list=30%, signal=66% |
| 894 | GOBP\_GLIAL\_CELL\_DIFFERENTIATION |  | 185 | -0.27 | -1.27 | 0.064 | 0.374 | 1.000 | 2798 | tags=26%, list=22%, signal=33% |
| 895 | GOBP\_GLOMERULUS\_DEVELOPMENT |  | 62 | -0.32 | -1.27 | 0.106 | 0.373 | 1.000 | 3893 | tags=39%, list=30%, signal=55% |
| 896 | GOBP\_T\_CELL\_APOPTOTIC\_PROCESS |  | 41 | -0.34 | -1.27 | 0.133 | 0.376 | 1.000 | 3730 | tags=46%, list=29%, signal=65% |
| 897 | GOBP\_CARBOHYDRATE\_TRANSPORT |  | 114 | -0.29 | -1.27 | 0.095 | 0.376 | 1.000 | 3281 | tags=32%, list=25%, signal=43% |
| 898 | GOBP\_COLLAGEN\_CATABOLIC\_PROCESS |  | 39 | -0.35 | -1.27 | 0.125 | 0.376 | 1.000 | 2657 | tags=28%, list=21%, signal=35% |
| 899 | GOBP\_POSITIVE\_REGULATION\_OF\_PROTEIN\_DEACETYLATION |  | 24 | -0.40 | -1.27 | 0.149 | 0.376 | 1.000 | 517 | tags=17%, list=4%, signal=17% |
| 900 | GOBP\_REGULATION\_OF\_NERVOUS\_SYSTEM\_PROCESS |  | 112 | -0.28 | -1.27 | 0.086 | 0.377 | 1.000 | 3118 | tags=29%, list=24%, signal=39% |
| 901 | GOBP\_MULTICELLULAR\_ORGANISMAL\_HOMEOSTASIS |  | 437 | -0.24 | -1.27 | 0.021 | 0.377 | 1.000 | 3846 | tags=36%, list=30%, signal=49% |
| 902 | GOBP\_PROTEIN\_IMPORT |  | 179 | -0.27 | -1.27 | 0.068 | 0.377 | 1.000 | 2926 | tags=31%, list=23%, signal=40% |
| 903 | GOBP\_NEGATIVE\_REGULATION\_OF\_MONONUCLEAR\_CELL\_MIGRATION |  | 20 | -0.41 | -1.27 | 0.159 | 0.377 | 1.000 | 3237 | tags=45%, list=25%, signal=60% |
| 904 | GOBP\_RESPONSE\_TO\_IONIZING\_RADIATION |  | 118 | -0.28 | -1.27 | 0.085 | 0.376 | 1.000 | 2082 | tags=24%, list=16%, signal=28% |
| 905 | GOBP\_NEGATIVE\_REGULATION\_OF\_CELL\_CELL\_ADHESION |  | 157 | -0.27 | -1.27 | 0.068 | 0.376 | 1.000 | 4200 | tags=43%, list=33%, signal=63% |
| 906 | GOBP\_ENDOPLASMIC\_RETICULUM\_UNFOLDED\_PROTEIN\_RESPONSE |  | 58 | -0.32 | -1.27 | 0.115 | 0.377 | 1.000 | 3636 | tags=40%, list=28%, signal=55% |
| 907 | GOBP\_CELLULAR\_RESPONSE\_TO\_EXTRACELLULAR\_STIMULUS |  | 203 | -0.26 | -1.27 | 0.055 | 0.377 | 1.000 | 2975 | tags=31%, list=23%, signal=40% |
| 908 | GOBP\_ANATOMICAL\_STRUCTURE\_MATURATION |  | 200 | -0.26 | -1.27 | 0.058 | 0.377 | 1.000 | 2696 | tags=27%, list=21%, signal=34% |
| 909 | GOBP\_REGULATION\_OF\_TRANSCRIPTION\_BY\_RNA\_POLYMERASE\_I |  | 27 | -0.38 | -1.27 | 0.144 | 0.377 | 1.000 | 4178 | tags=59%, list=32%, signal=87% |
| 910 | GOBP\_OVULATION\_CYCLE\_PROCESS |  | 46 | -0.34 | -1.26 | 0.129 | 0.378 | 1.000 | 4453 | tags=43%, list=35%, signal=66% |
| 911 | GOBP\_FIBROBLAST\_MIGRATION |  | 41 | -0.34 | -1.26 | 0.122 | 0.379 | 1.000 | 864 | tags=20%, list=7%, signal=21% |
| 912 | GOBP\_UROGENITAL\_SYSTEM\_DEVELOPMENT |  | 301 | -0.25 | -1.26 | 0.037 | 0.379 | 1.000 | 2791 | tags=25%, list=22%, signal=31% |
| 913 | GOBP\_VESICLE\_TRANSPORT\_ALONG\_MICROTUBULE |  | 34 | -0.36 | -1.26 | 0.140 | 0.379 | 1.000 | 2058 | tags=29%, list=16%, signal=35% |
| 914 | GOBP\_HISTONE\_H3\_K9\_MODIFICATION |  | 48 | -0.33 | -1.26 | 0.110 | 0.380 | 1.000 | 4638 | tags=54%, list=36%, signal=84% |
| 915 | GOBP\_NEGATIVE\_REGULATION\_OF\_CYSTEINE\_TYPE\_ENDOPEPTIDASE\_ACTIVITY |  | 72 | -0.31 | -1.26 | 0.123 | 0.380 | 1.000 | 2389 | tags=26%, list=19%, signal=32% |
| 916 | GOBP\_TRANSFORMING\_GROWTH\_FACTOR\_BETA\_PRODUCTION |  | 41 | -0.35 | -1.26 | 0.131 | 0.381 | 1.000 | 4310 | tags=41%, list=33%, signal=62% |
| 917 | GOBP\_POSITIVE\_REGULATION\_OF\_INTERLEUKIN\_17\_PRODUCTION |  | 21 | -0.40 | -1.26 | 0.155 | 0.382 | 1.000 | 699 | tags=19%, list=5%, signal=20% |
| 918 | GOBP\_POSITIVE\_REGULATION\_OF\_CARDIAC\_MUSCLE\_CELL\_PROLIFERATION |  | 21 | -0.41 | -1.26 | 0.184 | 0.383 | 1.000 | 1174 | tags=19%, list=9%, signal=21% |
| 919 | GOBP\_REGULATION\_OF\_PHOSPHATIDYLINOSITOL\_3\_KINASE\_ACTIVITY |  | 45 | -0.34 | -1.26 | 0.142 | 0.383 | 1.000 | 2437 | tags=31%, list=19%, signal=38% |
| 920 | GOBP\_SENSORY\_PERCEPTION\_OF\_MECHANICAL\_STIMULUS |  | 125 | -0.28 | -1.26 | 0.080 | 0.383 | 1.000 | 3631 | tags=35%, list=28%, signal=49% |
| 921 | GOBP\_METAL\_ION\_EXPORT |  | 40 | -0.35 | -1.26 | 0.144 | 0.383 | 1.000 | 3015 | tags=35%, list=23%, signal=46% |
| 922 | GOBP\_POSITIVE\_REGULATION\_OF\_MITOTIC\_NUCLEAR\_DIVISION |  | 37 | -0.35 | -1.26 | 0.173 | 0.383 | 1.000 | 2187 | tags=22%, list=17%, signal=26% |
| 923 | GOBP\_BROWN\_FAT\_CELL\_DIFFERENTIATION |  | 41 | -0.34 | -1.26 | 0.152 | 0.385 | 1.000 | 863 | tags=17%, list=7%, signal=18% |
| 924 | GOBP\_REGULATION\_OF\_AXONOGENESIS |  | 134 | -0.28 | -1.26 | 0.086 | 0.385 | 1.000 | 3240 | tags=30%, list=25%, signal=39% |
| 925 | GOBP\_METANEPHRIC\_TUBULE\_DEVELOPMENT |  | 16 | -0.44 | -1.26 | 0.202 | 0.385 | 1.000 | 817 | tags=19%, list=6%, signal=20% |
| 926 | GOBP\_CGMP\_METABOLIC\_PROCESS |  | 16 | -0.43 | -1.26 | 0.170 | 0.386 | 1.000 | 4425 | tags=56%, list=34%, signal=86% |
| 927 | GOBP\_NEURAL\_CREST\_CELL\_MIGRATION |  | 55 | -0.32 | -1.26 | 0.134 | 0.388 | 1.000 | 1776 | tags=25%, list=14%, signal=29% |
| 928 | GOBP\_SULFUR\_COMPOUND\_TRANSPORT |  | 39 | -0.35 | -1.26 | 0.151 | 0.389 | 1.000 | 4049 | tags=46%, list=31%, signal=67% |
| 929 | GOBP\_REGULATION\_OF\_CYTOKINESIS |  | 73 | -0.31 | -1.26 | 0.105 | 0.389 | 1.000 | 960 | tags=15%, list=7%, signal=16% |
| 930 | GOBP\_STEM\_CELL\_DIFFERENTIATION |  | 176 | -0.27 | -1.26 | 0.086 | 0.389 | 1.000 | 3867 | tags=39%, list=30%, signal=54% |
| 931 | GOBP\_MITOCHONDRIAL\_RNA\_METABOLIC\_PROCESS |  | 38 | -0.35 | -1.26 | 0.137 | 0.391 | 1.000 | 2032 | tags=26%, list=16%, signal=31% |
| 932 | GOBP\_REGULATION\_OF\_FIBROBLAST\_MIGRATION |  | 30 | -0.36 | -1.25 | 0.166 | 0.391 | 1.000 | 1404 | tags=23%, list=11%, signal=26% |
| 933 | GOBP\_PROTEIN\_DEPHOSPHORYLATION |  | 226 | -0.26 | -1.25 | 0.040 | 0.391 | 1.000 | 2956 | tags=28%, list=23%, signal=36% |
| 934 | GOBP\_REGULATION\_OF\_CELLULAR\_RESPONSE\_TO\_TRANSFORMING\_GROWTH\_FACTOR\_BETA\_STIMULUS |  | 99 | -0.29 | -1.25 | 0.092 | 0.391 | 1.000 | 3589 | tags=37%, list=28%, signal=51% |
| 935 | GOBP\_CELL\_ADHESION\_MEDIATED\_BY\_INTEGRIN |  | 75 | -0.30 | -1.25 | 0.111 | 0.391 | 1.000 | 3606 | tags=43%, list=28%, signal=59% |
| 936 | GOBP\_VASCULAR\_ENDOTHELIAL\_GROWTH\_FACTOR\_SIGNALING\_PATHWAY |  | 38 | -0.35 | -1.25 | 0.139 | 0.391 | 1.000 | 2076 | tags=24%, list=16%, signal=28% |
| 937 | GOBP\_STEM\_CELL\_PROLIFERATION |  | 57 | -0.32 | -1.25 | 0.135 | 0.392 | 1.000 | 2258 | tags=21%, list=18%, signal=25% |
| 938 | GOBP\_DEVELOPMENTAL\_INDUCTION |  | 28 | -0.38 | -1.25 | 0.183 | 0.392 | 1.000 | 2511 | tags=29%, list=19%, signal=35% |
| 939 | GOBP\_CELLULAR\_RESPONSE\_TO\_UNFOLDED\_PROTEIN |  | 77 | -0.30 | -1.25 | 0.118 | 0.392 | 1.000 | 4073 | tags=43%, list=32%, signal=62% |
| 940 | GOBP\_NEGATIVE\_REGULATION\_OF\_ALPHA\_BETA\_T\_CELL\_ACTIVATION |  | 34 | -0.36 | -1.25 | 0.161 | 0.392 | 1.000 | 2078 | tags=29%, list=16%, signal=35% |
| 941 | GOBP\_COLLAGEN\_BIOSYNTHETIC\_PROCESS |  | 37 | -0.35 | -1.25 | 0.158 | 0.393 | 1.000 | 5223 | tags=54%, list=40%, signal=91% |
| 942 | GOBP\_POSITIVE\_REGULATION\_OF\_T\_CELL\_MEDIATED\_IMMUNITY |  | 56 | -0.32 | -1.25 | 0.140 | 0.393 | 1.000 | 2833 | tags=30%, list=22%, signal=39% |
| 943 | GOBP\_EPITHELIAL\_TUBE\_MORPHOGENESIS |  | 265 | -0.25 | -1.25 | 0.039 | 0.393 | 1.000 | 4147 | tags=39%, list=32%, signal=57% |
| 944 | GOBP\_POSITIVE\_REGULATION\_OF\_CYTOKINE\_PRODUCTION |  | 395 | -0.24 | -1.25 | 0.027 | 0.393 | 1.000 | 3701 | tags=34%, list=29%, signal=46% |
| 945 | GOBP\_INTRINSIC\_APOPTOTIC\_SIGNALING\_PATHWAY\_IN\_RESPONSE\_TO\_ENDOPLASMIC\_RETICULUM\_STRESS |  | 52 | -0.32 | -1.25 | 0.131 | 0.393 | 1.000 | 3636 | tags=40%, list=28%, signal=56% |
| 946 | GOBP\_POSITIVE\_REGULATION\_OF\_GLYCOLYTIC\_PROCESS |  | 19 | -0.42 | -1.25 | 0.178 | 0.394 | 1.000 | 1403 | tags=26%, list=11%, signal=29% |
| 947 | GOBP\_HEMATOPOIETIC\_PROGENITOR\_CELL\_DIFFERENTIATION |  | 93 | -0.29 | -1.25 | 0.114 | 0.396 | 1.000 | 1866 | tags=24%, list=14%, signal=27% |
| 948 | GOBP\_POSITIVE\_REGULATION\_OF\_ENDOTHELIAL\_CELL\_APOPTOTIC\_PROCESS |  | 16 | -0.43 | -1.25 | 0.207 | 0.397 | 1.000 | 2180 | tags=38%, list=17%, signal=45% |
| 949 | GOBP\_RENAL\_VESICLE\_DEVELOPMENT |  | 15 | -0.43 | -1.25 | 0.212 | 0.399 | 1.000 | 817 | tags=20%, list=6%, signal=21% |
| 950 | GOBP\_REGULATION\_OF\_CELL\_SIZE |  | 154 | -0.27 | -1.25 | 0.092 | 0.400 | 1.000 | 2454 | tags=25%, list=19%, signal=30% |
| 951 | GOBP\_CARDIAC\_MUSCLE\_CELL\_PROLIFERATION |  | 42 | -0.34 | -1.25 | 0.141 | 0.401 | 1.000 | 1401 | tags=19%, list=11%, signal=21% |
| 952 | GOBP\_REGULATION\_OF\_CELL\_SUBSTRATE\_ADHESION |  | 188 | -0.26 | -1.25 | 0.085 | 0.402 | 1.000 | 3821 | tags=37%, list=30%, signal=51% |
| 953 | GOBP\_LEUKOCYTE\_ADHESION\_TO\_VASCULAR\_ENDOTHELIAL\_CELL |  | 43 | -0.34 | -1.25 | 0.159 | 0.402 | 1.000 | 856 | tags=16%, list=7%, signal=17% |
| 954 | GOBP\_POSITIVE\_REGULATION\_OF\_CYCLIN\_DEPENDENT\_PROTEIN\_KINASE\_ACTIVITY |  | 28 | -0.37 | -1.25 | 0.156 | 0.401 | 1.000 | 2207 | tags=32%, list=17%, signal=39% |
| 955 | GOBP\_ORGANIC\_ACID\_TRANSMEMBRANE\_TRANSPORT |  | 117 | -0.28 | -1.25 | 0.100 | 0.401 | 1.000 | 2998 | tags=31%, list=23%, signal=40% |
| 956 | GOBP\_NEGATIVE\_REGULATION\_OF\_G\_PROTEIN\_COUPLED\_RECEPTOR\_SIGNALING\_PATHWAY |  | 47 | -0.33 | -1.25 | 0.149 | 0.401 | 1.000 | 1931 | tags=28%, list=15%, signal=32% |
| 957 | GOBP\_PEPTIDYL\_LYSINE\_TRIMETHYLATION |  | 45 | -0.34 | -1.25 | 0.155 | 0.402 | 1.000 | 2940 | tags=33%, list=23%, signal=43% |
| 958 | GOBP\_ORGANIC\_ANION\_TRANSPORT |  | 274 | -0.25 | -1.25 | 0.045 | 0.402 | 1.000 | 2806 | tags=26%, list=22%, signal=32% |
| 959 | GOBP\_BLOOD\_VESSEL\_ENDOTHELIAL\_CELL\_PROLIFERATION\_INVOLVED\_IN\_SPROUTING\_ANGIOGENESIS |  | 20 | -0.41 | -1.25 | 0.188 | 0.402 | 1.000 | 1844 | tags=25%, list=14%, signal=29% |
| 960 | GOBP\_POSITIVE\_REGULATION\_OF\_CHROMOSOME\_ORGANIZATION |  | 77 | -0.30 | -1.25 | 0.127 | 0.401 | 1.000 | 2392 | tags=22%, list=19%, signal=27% |
| 961 | GOBP\_CELLULAR\_RESPONSE\_TO\_RADIATION |  | 155 | -0.27 | -1.25 | 0.087 | 0.401 | 1.000 | 2495 | tags=25%, list=19%, signal=31% |
| 962 | GOBP\_REGULATION\_OF\_PROTEIN\_ACETYLATION |  | 70 | -0.30 | -1.25 | 0.117 | 0.401 | 1.000 | 4095 | tags=51%, list=32%, signal=75% |
| 963 | GOBP\_LONG\_TERM\_MEMORY |  | 29 | -0.36 | -1.24 | 0.160 | 0.401 | 1.000 | 4215 | tags=48%, list=33%, signal=72% |
| 964 | GOBP\_LUNG\_ALVEOLUS\_DEVELOPMENT |  | 36 | -0.35 | -1.24 | 0.163 | 0.402 | 1.000 | 4292 | tags=44%, list=33%, signal=66% |
| 965 | GOBP\_PULMONARY\_VALVE\_DEVELOPMENT |  | 19 | -0.41 | -1.24 | 0.181 | 0.402 | 1.000 | 4706 | tags=58%, list=36%, signal=91% |
| 966 | GOBP\_INTERLEUKIN\_2\_PRODUCTION |  | 58 | -0.32 | -1.24 | 0.134 | 0.403 | 1.000 | 1733 | tags=19%, list=13%, signal=22% |
| 967 | GOBP\_POSITIVE\_REGULATION\_OF\_VASCULAR\_ASSOCIATED\_SMOOTH\_MUSCLE\_CELL\_PROLIFERATION |  | 31 | -0.36 | -1.24 | 0.171 | 0.403 | 1.000 | 1979 | tags=26%, list=15%, signal=30% |
| 968 | GOBP\_POSITIVE\_REGULATION\_OF\_VASOCONSTRICTION |  | 30 | -0.37 | -1.24 | 0.164 | 0.403 | 1.000 | 4933 | tags=53%, list=38%, signal=86% |
| 969 | GOBP\_REGULATION\_OF\_BMP\_SIGNALING\_PATHWAY |  | 77 | -0.30 | -1.24 | 0.117 | 0.403 | 1.000 | 4391 | tags=49%, list=34%, signal=74% |
| 970 | GOBP\_REGULATION\_OF\_STRESS\_ACTIVATED\_PROTEIN\_KINASE\_SIGNALING\_CASCADE |  | 174 | -0.26 | -1.24 | 0.090 | 0.402 | 1.000 | 1405 | tags=17%, list=11%, signal=18% |
| 971 | GOBP\_REGULATION\_OF\_LIPID\_BIOSYNTHETIC\_PROCESS |  | 140 | -0.27 | -1.24 | 0.092 | 0.402 | 1.000 | 2078 | tags=23%, list=16%, signal=27% |
| 972 | GOBP\_POSITIVE\_REGULATION\_OF\_PROTEIN\_MATURATION |  | 23 | -0.39 | -1.24 | 0.204 | 0.402 | 1.000 | 4439 | tags=65%, list=34%, signal=99% |
| 973 | GOBP\_OLFACTORY\_LOBE\_DEVELOPMENT |  | 27 | -0.38 | -1.24 | 0.152 | 0.402 | 1.000 | 2150 | tags=26%, list=17%, signal=31% |
| 974 | GOBP\_TRANSLATIONAL\_ELONGATION |  | 41 | -0.34 | -1.24 | 0.179 | 0.402 | 1.000 | 1775 | tags=27%, list=14%, signal=31% |
| 975 | GOBP\_REGULATION\_OF\_LEUKOCYTE\_DIFFERENTIATION |  | 241 | -0.25 | -1.24 | 0.065 | 0.402 | 1.000 | 3872 | tags=36%, list=30%, signal=50% |
| 976 | GOBP\_REGULATION\_OF\_CELLULAR\_RESPONSE\_TO\_INSULIN\_STIMULUS |  | 55 | -0.32 | -1.24 | 0.134 | 0.403 | 1.000 | 1704 | tags=25%, list=13%, signal=29% |
| 977 | GOBP\_NEGATIVE\_REGULATION\_OF\_CELL\_SUBSTRATE\_ADHESION |  | 55 | -0.32 | -1.24 | 0.122 | 0.404 | 1.000 | 3708 | tags=47%, list=29%, signal=66% |
| 978 | GOBP\_REGULATION\_OF\_MESENCHYMAL\_CELL\_PROLIFERATION |  | 29 | -0.36 | -1.24 | 0.179 | 0.405 | 1.000 | 2969 | tags=34%, list=23%, signal=45% |
| 979 | GOBP\_REGULATION\_OF\_HEMOPOIESIS |  | 310 | -0.25 | -1.24 | 0.038 | 0.406 | 1.000 | 2403 | tags=23%, list=19%, signal=27% |
| 980 | GOBP\_REGULATION\_OF\_SKELETAL\_MUSCLE\_TISSUE\_DEVELOPMENT |  | 21 | -0.39 | -1.24 | 0.182 | 0.406 | 1.000 | 1403 | tags=24%, list=11%, signal=27% |
| 981 | GOBP\_REGULATION\_OF\_CELLULAR\_CARBOHYDRATE\_METABOLIC\_PROCESS |  | 126 | -0.28 | -1.24 | 0.106 | 0.407 | 1.000 | 2084 | tags=23%, list=16%, signal=27% |
| 982 | GOBP\_NEGATIVE\_REGULATION\_OF\_PROTEIN\_SERINE\_THREONINE\_KINASE\_ACTIVITY |  | 98 | -0.28 | -1.24 | 0.108 | 0.408 | 1.000 | 3439 | tags=36%, list=27%, signal=48% |
| 983 | GOBP\_RECEPTOR\_MEDIATED\_ENDOCYTOSIS |  | 212 | -0.26 | -1.24 | 0.072 | 0.408 | 1.000 | 2085 | tags=23%, list=16%, signal=27% |
| 984 | GOBP\_SKIN\_DEVELOPMENT |  | 204 | -0.25 | -1.24 | 0.071 | 0.408 | 1.000 | 4219 | tags=40%, list=33%, signal=58% |
| 985 | GOBP\_NEGATIVE\_REGULATION\_OF\_MUSCLE\_CELL\_DIFFERENTIATION |  | 46 | -0.33 | -1.24 | 0.155 | 0.408 | 1.000 | 2124 | tags=28%, list=16%, signal=34% |
| 986 | GOBP\_REGULATION\_OF\_TELOMERE\_CAPPING |  | 24 | -0.39 | -1.24 | 0.197 | 0.410 | 1.000 | 934 | tags=17%, list=7%, signal=18% |
| 987 | GOBP\_IMPORT\_ACROSS\_PLASMA\_MEMBRANE |  | 146 | -0.27 | -1.24 | 0.101 | 0.410 | 1.000 | 3081 | tags=31%, list=24%, signal=40% |
| 988 | GOBP\_REGULATION\_OF\_MITOTIC\_CELL\_CYCLE |  | 368 | -0.24 | -1.24 | 0.042 | 0.411 | 1.000 | 2406 | tags=21%, list=19%, signal=25% |
| 989 | GOBP\_RESPONSE\_TO\_EXTRACELLULAR\_STIMULUS |  | 410 | -0.24 | -1.24 | 0.042 | 0.411 | 1.000 | 2981 | tags=29%, list=23%, signal=36% |
| 990 | GOBP\_DENDRITE\_DEVELOPMENT |  | 196 | -0.26 | -1.24 | 0.082 | 0.411 | 1.000 | 3215 | tags=30%, list=25%, signal=39% |
| 991 | GOBP\_NEGATIVE\_REGULATION\_OF\_CELL\_JUNCTION\_ASSEMBLY |  | 27 | -0.37 | -1.24 | 0.189 | 0.411 | 1.000 | 2280 | tags=37%, list=18%, signal=45% |
| 992 | GOBP\_HISTONE\_DEUBIQUITINATION |  | 32 | -0.36 | -1.24 | 0.176 | 0.411 | 1.000 | 1362 | tags=25%, list=11%, signal=28% |
| 993 | GOBP\_TYROSINE\_PHOSPHORYLATION\_OF\_STAT\_PROTEIN |  | 67 | -0.31 | -1.24 | 0.135 | 0.411 | 1.000 | 1994 | tags=22%, list=15%, signal=26% |
| 994 | GOBP\_REGULATION\_OF\_INTRINSIC\_APOPTOTIC\_SIGNALING\_PATHWAY\_BY\_P53\_CLASS\_MEDIATOR |  | 26 | -0.38 | -1.24 | 0.186 | 0.410 | 1.000 | 2294 | tags=27%, list=18%, signal=33% |
| 995 | GOBP\_POSITIVE\_REGULATION\_OF\_T\_CELL\_MEDIATED\_CYTOTOXICITY |  | 28 | -0.37 | -1.24 | 0.183 | 0.410 | 1.000 | 2833 | tags=36%, list=22%, signal=46% |
| 996 | GOBP\_POSITIVE\_REGULATION\_OF\_CANONICAL\_WNT\_SIGNALING\_PATHWAY |  | 84 | -0.29 | -1.24 | 0.120 | 0.410 | 1.000 | 1639 | tags=20%, list=13%, signal=23% |
| 997 | GOBP\_RESPONSE\_TO\_INTERFERON\_GAMMA |  | 114 | -0.28 | -1.23 | 0.112 | 0.410 | 1.000 | 3794 | tags=37%, list=29%, signal=52% |
| 998 | GOBP\_DNA\_STRAND\_ELONGATION |  | 34 | -0.35 | -1.23 | 0.150 | 0.410 | 1.000 | 2248 | tags=35%, list=17%, signal=43% |
| 999 | GOBP\_REGULATION\_OF\_CELL\_CYCLE\_PHASE\_TRANSITION |  | 303 | -0.25 | -1.23 | 0.054 | 0.410 | 1.000 | 1671 | tags=17%, list=13%, signal=19% |
| 1000 | GOBP\_MAMMARY\_GLAND\_EPITHELIUM\_DEVELOPMENT |  | 61 | -0.31 | -1.23 | 0.161 | 0.410 | 1.000 | 2969 | tags=31%, list=23%, signal=40% |
| 1001 | GOBP\_REGULATION\_OF\_PROTEIN\_MODIFICATION\_BY\_SMALL\_PROTEIN\_CONJUGATION\_OR\_REMOVAL |  | 200 | -0.26 | -1.23 | 0.076 | 0.410 | 1.000 | 2927 | tags=29%, list=23%, signal=36% |
| 1002 | GOBP\_OUTFLOW\_TRACT\_MORPHOGENESIS |  | 73 | -0.30 | -1.23 | 0.140 | 0.410 | 1.000 | 2059 | tags=23%, list=16%, signal=28% |
| 1003 | GOBP\_REGULATION\_OF\_CELL\_MORPHOGENESIS |  | 253 | -0.25 | -1.23 | 0.043 | 0.410 | 1.000 | 4206 | tags=37%, list=33%, signal=54% |
| 1004 | GOBP\_RESPONSE\_TO\_TRANSFORMING\_GROWTH\_FACTOR\_BETA |  | 216 | -0.25 | -1.23 | 0.069 | 0.411 | 1.000 | 3633 | tags=35%, list=28%, signal=48% |
| 1005 | GOBP\_INTRACELLULAR\_RECEPTOR\_SIGNALING\_PATHWAY |  | 234 | -0.25 | -1.23 | 0.071 | 0.412 | 1.000 | 3018 | tags=27%, list=23%, signal=35% |
| 1006 | GOBP\_REGULATION\_OF\_POLYSACCHARIDE\_METABOLIC\_PROCESS |  | 36 | -0.35 | -1.23 | 0.161 | 0.413 | 1.000 | 2072 | tags=31%, list=16%, signal=36% |
| 1007 | GOBP\_POSITIVE\_REGULATION\_OF\_NERVOUS\_SYSTEM\_DEVELOPMENT |  | 221 | -0.25 | -1.23 | 0.082 | 0.413 | 1.000 | 3182 | tags=27%, list=25%, signal=35% |
| 1008 | GOBP\_SENSORY\_PERCEPTION\_OF\_CHEMICAL\_STIMULUS |  | 104 | -0.28 | -1.23 | 0.116 | 0.413 | 1.000 | 4122 | tags=37%, list=32%, signal=53% |
| 1009 | GOBP\_NEGATIVE\_REGULATION\_OF\_PROTEIN\_MATURATION |  | 19 | -0.41 | -1.23 | 0.196 | 0.413 | 1.000 | 4261 | tags=47%, list=33%, signal=71% |
| 1010 | GOBP\_POSITIVE\_REGULATION\_OF\_MESENCHYMAL\_CELL\_PROLIFERATION |  | 23 | -0.38 | -1.23 | 0.197 | 0.413 | 1.000 | 2969 | tags=39%, list=23%, signal=51% |
| 1011 | GOBP\_REGULATION\_OF\_STEM\_CELL\_PROLIFERATION |  | 37 | -0.34 | -1.23 | 0.179 | 0.413 | 1.000 | 3577 | tags=35%, list=28%, signal=48% |
| 1012 | GOBP\_HISTONE\_H3\_K4\_METHYLATION |  | 58 | -0.31 | -1.23 | 0.148 | 0.414 | 1.000 | 2940 | tags=31%, list=23%, signal=40% |
| 1013 | GOBP\_RESPONSE\_TO\_TOPOLOGICALLY\_INCORRECT\_PROTEIN |  | 127 | -0.27 | -1.23 | 0.109 | 0.414 | 1.000 | 3762 | tags=37%, list=29%, signal=52% |
| 1014 | GOBP\_NEGATIVE\_REGULATION\_OF\_CELLULAR\_PROTEIN\_CATABOLIC\_PROCESS |  | 62 | -0.30 | -1.23 | 0.122 | 0.415 | 1.000 | 3215 | tags=37%, list=25%, signal=49% |
| 1015 | GOBP\_PERK\_MEDIATED\_UNFOLDED\_PROTEIN\_RESPONSE |  | 15 | -0.43 | -1.23 | 0.205 | 0.416 | 1.000 | 4073 | tags=67%, list=32%, signal=97% |
| 1016 | GOBP\_POSITIVE\_REGULATION\_OF\_CHEMOKINE\_PRODUCTION |  | 61 | -0.31 | -1.23 | 0.146 | 0.417 | 1.000 | 1629 | tags=23%, list=13%, signal=26% |
| 1017 | GOBP\_EMBRYONIC\_FORELIMB\_MORPHOGENESIS |  | 26 | -0.37 | -1.23 | 0.191 | 0.418 | 1.000 | 1206 | tags=23%, list=9%, signal=25% |
| 1018 | GOBP\_RESPONSE\_TO\_AMINO\_ACID\_STARVATION |  | 39 | -0.34 | -1.23 | 0.180 | 0.419 | 1.000 | 2251 | tags=33%, list=17%, signal=40% |
| 1019 | GOBP\_REGULATION\_OF\_PROTEIN\_MATURATION |  | 52 | -0.32 | -1.23 | 0.171 | 0.420 | 1.000 | 4505 | tags=56%, list=35%, signal=85% |
| 1020 | GOBP\_NEGATIVE\_REGULATION\_OF\_LIPID\_STORAGE |  | 20 | -0.40 | -1.23 | 0.193 | 0.420 | 1.000 | 3032 | tags=50%, list=24%, signal=65% |
| 1021 | GOBP\_TUMOR\_NECROSIS\_FACTOR\_MEDIATED\_SIGNALING\_PATHWAY |  | 85 | -0.29 | -1.23 | 0.128 | 0.420 | 1.000 | 2511 | tags=26%, list=19%, signal=32% |
| 1022 | GOBP\_NEGATIVE\_REGULATION\_OF\_BMP\_SIGNALING\_PATHWAY |  | 40 | -0.34 | -1.23 | 0.166 | 0.420 | 1.000 | 2547 | tags=30%, list=20%, signal=37% |
| 1023 | GOBP\_REGULATION\_OF\_CELL\_AGING |  | 40 | -0.34 | -1.23 | 0.164 | 0.421 | 1.000 | 4895 | tags=58%, list=38%, signal=92% |
| 1024 | GOBP\_ORGAN\_OR\_TISSUE\_SPECIFIC\_IMMUNE\_RESPONSE |  | 33 | -0.35 | -1.23 | 0.188 | 0.422 | 1.000 | 1686 | tags=21%, list=13%, signal=24% |
| 1025 | GOBP\_MYELOID\_LEUKOCYTE\_CYTOKINE\_PRODUCTION |  | 29 | -0.36 | -1.22 | 0.180 | 0.422 | 1.000 | 519 | tags=17%, list=4%, signal=18% |
| 1026 | GOBP\_POSITIVE\_REGULATION\_OF\_NEURON\_DEATH |  | 82 | -0.29 | -1.22 | 0.146 | 0.422 | 1.000 | 2604 | tags=28%, list=20%, signal=35% |
| 1027 | GOBP\_GENITALIA\_DEVELOPMENT |  | 39 | -0.34 | -1.22 | 0.182 | 0.422 | 1.000 | 4414 | tags=44%, list=34%, signal=66% |
| 1028 | GOBP\_REGULATION\_OF\_POTASSIUM\_ION\_TRANSMEMBRANE\_TRANSPORTER\_ACTIVITY |  | 45 | -0.33 | -1.22 | 0.173 | 0.423 | 1.000 | 1739 | tags=22%, list=13%, signal=26% |
| 1029 | GOBP\_RESPONSE\_TO\_SALT |  | 19 | -0.40 | -1.22 | 0.189 | 0.424 | 1.000 | 2082 | tags=37%, list=16%, signal=44% |
| 1030 | GOBP\_MESENCHYMAL\_CELL\_DEVELOPMENT |  | 80 | -0.29 | -1.22 | 0.144 | 0.425 | 1.000 | 1960 | tags=25%, list=15%, signal=29% |
| 1031 | GOBP\_PEPTIDYL\_LYSINE\_METHYLATION |  | 110 | -0.28 | -1.22 | 0.133 | 0.425 | 1.000 | 2940 | tags=29%, list=23%, signal=37% |
| 1032 | GOBP\_HISTONE\_H3\_K9\_METHYLATION |  | 35 | -0.35 | -1.22 | 0.192 | 0.425 | 1.000 | 4824 | tags=57%, list=37%, signal=91% |
| 1033 | GOBP\_REGULATORY\_T\_CELL\_DIFFERENTIATION |  | 31 | -0.35 | -1.22 | 0.188 | 0.424 | 1.000 | 3609 | tags=32%, list=28%, signal=45% |
| 1034 | GOBP\_G1\_TO\_G0\_TRANSITION |  | 16 | -0.42 | -1.22 | 0.202 | 0.425 | 1.000 | 3657 | tags=50%, list=28%, signal=70% |
| 1035 | GOBP\_REGULATION\_OF\_CARDIAC\_MUSCLE\_CELL\_PROLIFERATION |  | 35 | -0.35 | -1.22 | 0.218 | 0.425 | 1.000 | 1401 | tags=20%, list=11%, signal=22% |
| 1036 | GOBP\_NEGATIVE\_REGULATION\_OF\_PROTEIN\_MODIFICATION\_BY\_SMALL\_PROTEIN\_CONJUGATION\_OR\_REMOVAL |  | 79 | -0.30 | -1.22 | 0.139 | 0.425 | 1.000 | 1657 | tags=22%, list=13%, signal=25% |
| 1037 | GOBP\_NEGATIVE\_REGULATION\_OF\_NF\_KAPPAB\_TRANSCRIPTION\_FACTOR\_ACTIVITY |  | 66 | -0.30 | -1.22 | 0.167 | 0.425 | 1.000 | 1625 | tags=23%, list=13%, signal=26% |
| 1038 | GOBP\_REGULATION\_OF\_LIPID\_STORAGE |  | 45 | -0.33 | -1.22 | 0.164 | 0.426 | 1.000 | 3256 | tags=38%, list=25%, signal=50% |
| 1039 | GOBP\_REGULATION\_OF\_PROTEIN\_CATABOLIC\_PROCESS |  | 323 | -0.24 | -1.22 | 0.071 | 0.426 | 1.000 | 2926 | tags=28%, list=23%, signal=35% |
| 1040 | GOBP\_NEGATIVE\_REGULATION\_OF\_CELL\_MATRIX\_ADHESION |  | 31 | -0.36 | -1.22 | 0.185 | 0.425 | 1.000 | 3708 | tags=52%, list=29%, signal=72% |
| 1041 | GOBP\_REGULATION\_OF\_CHROMATIN\_BINDING |  | 19 | -0.40 | -1.22 | 0.205 | 0.425 | 1.000 | 2174 | tags=32%, list=17%, signal=38% |
| 1042 | GOBP\_REGULATION\_OF\_MEIOTIC\_CELL\_CYCLE |  | 36 | -0.35 | -1.22 | 0.194 | 0.425 | 1.000 | 3401 | tags=39%, list=26%, signal=53% |
| 1043 | GOBP\_REGULATION\_OF\_RENAL\_SYSTEM\_PROCESS |  | 33 | -0.35 | -1.22 | 0.182 | 0.425 | 1.000 | 1626 | tags=24%, list=13%, signal=28% |
| 1044 | GOBP\_MICROTUBULE\_NUCLEATION |  | 30 | -0.36 | -1.22 | 0.184 | 0.424 | 1.000 | 4215 | tags=50%, list=33%, signal=74% |
| 1045 | GOBP\_LENS\_FIBER\_CELL\_DIFFERENTIATION |  | 34 | -0.35 | -1.22 | 0.179 | 0.424 | 1.000 | 3704 | tags=35%, list=29%, signal=49% |
| 1046 | GOBP\_REGULATION\_OF\_UBIQUITIN\_PROTEIN\_LIGASE\_ACTIVITY |  | 20 | -0.40 | -1.22 | 0.195 | 0.424 | 1.000 | 4448 | tags=70%, list=34%, signal=107% |
| 1047 | GOBP\_NEGATIVE\_REGULATION\_OF\_LIPID\_LOCALIZATION |  | 41 | -0.33 | -1.22 | 0.184 | 0.424 | 1.000 | 3032 | tags=41%, list=24%, signal=54% |
| 1048 | GOBP\_POSITIVE\_REGULATION\_OF\_TRANSMEMBRANE\_RECEPTOR\_PROTEIN\_SERINE\_THREONINE\_KINASE\_SIGNALING\_PATHWAY |  | 105 | -0.28 | -1.22 | 0.123 | 0.425 | 1.000 | 2956 | tags=30%, list=23%, signal=39% |
| 1049 | GOBP\_FOCAL\_ADHESION\_ASSEMBLY |  | 76 | -0.30 | -1.22 | 0.135 | 0.426 | 1.000 | 4393 | tags=49%, list=34%, signal=73% |
| 1050 | GOBP\_INTERMEMBRANE\_LIPID\_TRANSFER |  | 38 | -0.34 | -1.22 | 0.179 | 0.426 | 1.000 | 3048 | tags=34%, list=24%, signal=45% |
| 1051 | GOBP\_POSITIVE\_REGULATION\_OF\_MITOTIC\_CELL\_CYCLE |  | 92 | -0.28 | -1.22 | 0.141 | 0.427 | 1.000 | 1713 | tags=20%, list=13%, signal=22% |
| 1052 | GOBP\_TRANSITION\_METAL\_ION\_TRANSPORT |  | 79 | -0.29 | -1.22 | 0.154 | 0.427 | 1.000 | 3317 | tags=41%, list=26%, signal=54% |
| 1053 | GOBP\_ALPHA\_BETA\_T\_CELL\_DIFFERENTIATION |  | 92 | -0.29 | -1.22 | 0.127 | 0.427 | 1.000 | 2819 | tags=28%, list=22%, signal=36% |
| 1054 | GOBP\_RESPONSE\_TO\_ACTIVITY |  | 56 | -0.31 | -1.22 | 0.156 | 0.427 | 1.000 | 2561 | tags=32%, list=20%, signal=40% |
| 1055 | GOBP\_POSITIVE\_REGULATION\_OF\_BLOOD\_PRESSURE |  | 34 | -0.34 | -1.22 | 0.178 | 0.428 | 1.000 | 3182 | tags=32%, list=25%, signal=43% |
| 1056 | GOBP\_SYNAPTIC\_VESICLE\_LOCALIZATION |  | 39 | -0.33 | -1.22 | 0.195 | 0.428 | 1.000 | 1325 | tags=21%, list=10%, signal=23% |
| 1057 | GOBP\_MICROTUBULE\_ORGANIZING\_CENTER\_ORGANIZATION |  | 114 | -0.27 | -1.22 | 0.111 | 0.428 | 1.000 | 3538 | tags=35%, list=27%, signal=48% |
| 1058 | GOBP\_REGULATION\_OF\_CELL\_CELL\_ADHESION |  | 379 | -0.24 | -1.22 | 0.049 | 0.429 | 1.000 | 3890 | tags=35%, list=30%, signal=49% |
| 1059 | GOBP\_SYNAPTIC\_VESICLE\_RECYCLING |  | 60 | -0.31 | -1.22 | 0.163 | 0.429 | 1.000 | 2907 | tags=27%, list=23%, signal=34% |
| 1060 | GOBP\_POSITIVE\_REGULATION\_OF\_DNA\_BINDING |  | 53 | -0.32 | -1.22 | 0.167 | 0.429 | 1.000 | 1206 | tags=17%, list=9%, signal=19% |
| 1061 | GOBP\_POSITIVE\_REGULATION\_OF\_CELL\_ADHESION |  | 386 | -0.23 | -1.22 | 0.060 | 0.429 | 1.000 | 3691 | tags=32%, list=29%, signal=44% |
| 1062 | GOBP\_POSITIVE\_REGULATION\_OF\_TELOMERE\_CAPPING |  | 15 | -0.43 | -1.22 | 0.211 | 0.430 | 1.000 | 934 | tags=20%, list=7%, signal=22% |
| 1063 | GOBP\_CYTOPLASMIC\_TRANSLATIONAL\_INITIATION |  | 24 | -0.38 | -1.21 | 0.220 | 0.430 | 1.000 | 855 | tags=25%, list=7%, signal=27% |
| 1064 | GOBP\_PITUITARY\_GLAND\_DEVELOPMENT |  | 37 | -0.34 | -1.21 | 0.189 | 0.430 | 1.000 | 1983 | tags=27%, list=15%, signal=32% |
| 1065 | GOBP\_INNERVATION |  | 21 | -0.39 | -1.21 | 0.218 | 0.431 | 1.000 | 3605 | tags=43%, list=28%, signal=59% |
| 1066 | GOBP\_REGULATION\_OF\_TUMOR\_NECROSIS\_FACTOR\_MEDIATED\_SIGNALING\_PATHWAY |  | 42 | -0.33 | -1.21 | 0.187 | 0.430 | 1.000 | 4706 | tags=57%, list=36%, signal=90% |
| 1067 | GOBP\_ATRIAL\_SEPTUM\_DEVELOPMENT |  | 19 | -0.40 | -1.21 | 0.217 | 0.431 | 1.000 | 3611 | tags=42%, list=28%, signal=58% |
| 1068 | GOBP\_REGULATION\_OF\_TYPE\_I\_INTERFERON\_MEDIATED\_SIGNALING\_PATHWAY |  | 35 | -0.35 | -1.21 | 0.181 | 0.431 | 1.000 | 3707 | tags=49%, list=29%, signal=68% |
| 1069 | GOBP\_POSITIVE\_REGULATION\_OF\_TRANSLATIONAL\_INITIATION |  | 24 | -0.38 | -1.21 | 0.212 | 0.431 | 1.000 | 855 | tags=21%, list=7%, signal=22% |
| 1070 | GOBP\_SENSORY\_PERCEPTION |  | 454 | -0.23 | -1.21 | 0.048 | 0.431 | 1.000 | 3704 | tags=32%, list=29%, signal=43% |
| 1071 | GOBP\_POSITIVE\_T\_CELL\_SELECTION |  | 31 | -0.35 | -1.21 | 0.180 | 0.432 | 1.000 | 2085 | tags=26%, list=16%, signal=31% |
| 1072 | GOBP\_NEGATIVE\_REGULATION\_OF\_CYCLIN\_DEPENDENT\_PROTEIN\_SERINE\_THREONINE\_KINASE\_ACTIVITY |  | 25 | -0.38 | -1.21 | 0.204 | 0.433 | 1.000 | 2773 | tags=40%, list=21%, signal=51% |
| 1073 | GOBP\_PALLIUM\_DEVELOPMENT |  | 138 | -0.26 | -1.21 | 0.114 | 0.435 | 1.000 | 3734 | tags=33%, list=29%, signal=46% |
| 1074 | GOBP\_THYMIC\_T\_CELL\_SELECTION |  | 21 | -0.38 | -1.21 | 0.203 | 0.436 | 1.000 | 1863 | tags=29%, list=14%, signal=33% |
| 1075 | GOBP\_SENSORY\_PERCEPTION\_OF\_LIGHT\_STIMULUS |  | 166 | -0.26 | -1.21 | 0.108 | 0.436 | 1.000 | 2843 | tags=28%, list=22%, signal=36% |
| 1076 | GOBP\_POSTSYNAPSE\_ORGANIZATION |  | 130 | -0.27 | -1.21 | 0.120 | 0.436 | 1.000 | 4145 | tags=39%, list=32%, signal=57% |
| 1077 | GOBP\_RESPONSE\_TO\_CARBOHYDRATE |  | 188 | -0.25 | -1.21 | 0.085 | 0.436 | 1.000 | 2785 | tags=27%, list=22%, signal=33% |
| 1078 | GOBP\_REGULATION\_OF\_ANION\_TRANSPORT |  | 81 | -0.29 | -1.21 | 0.148 | 0.436 | 1.000 | 1982 | tags=22%, list=15%, signal=26% |
| 1079 | GOBP\_REGULATION\_OF\_NUCLEOTIDE\_BIOSYNTHETIC\_PROCESS |  | 33 | -0.34 | -1.21 | 0.195 | 0.436 | 1.000 | 3296 | tags=48%, list=26%, signal=65% |
| 1080 | GOBP\_POSITIVE\_REGULATION\_OF\_GTPASE\_ACTIVITY |  | 206 | -0.25 | -1.21 | 0.118 | 0.436 | 1.000 | 3137 | tags=29%, list=24%, signal=38% |
| 1081 | GOBP\_POSITIVE\_REGULATION\_OF\_MUSCLE\_TISSUE\_DEVELOPMENT |  | 20 | -0.40 | -1.21 | 0.222 | 0.436 | 1.000 | 4356 | tags=50%, list=34%, signal=75% |
| 1082 | GOBP\_CELLULAR\_NITROGEN\_COMPOUND\_CATABOLIC\_PROCESS |  | 355 | -0.24 | -1.21 | 0.065 | 0.436 | 1.000 | 2945 | tags=28%, list=23%, signal=35% |
| 1083 | GOBP\_REGULATION\_OF\_DNA\_METABOLIC\_PROCESS |  | 324 | -0.24 | -1.21 | 0.058 | 0.436 | 1.000 | 2469 | tags=22%, list=19%, signal=27% |
| 1084 | GOBP\_REGULATION\_OF\_PROTEIN\_SERINE\_THREONINE\_KINASE\_ACTIVITY |  | 323 | -0.24 | -1.21 | 0.078 | 0.436 | 1.000 | 3015 | tags=28%, list=23%, signal=36% |
| 1085 | GOBP\_POSITIVE\_REGULATION\_OF\_CIRCADIAN\_RHYTHM |  | 15 | -0.43 | -1.21 | 0.240 | 0.436 | 1.000 | 920 | tags=27%, list=7%, signal=29% |
| 1086 | GOBP\_B\_CELL\_DIFFERENTIATION |  | 119 | -0.27 | -1.21 | 0.120 | 0.436 | 1.000 | 2235 | tags=23%, list=17%, signal=27% |
| 1087 | GOBP\_NEGATIVE\_REGULATION\_OF\_INTRACELLULAR\_STEROID\_HORMONE\_RECEPTOR\_SIGNALING\_PATHWAY |  | 33 | -0.34 | -1.21 | 0.193 | 0.437 | 1.000 | 1655 | tags=21%, list=13%, signal=24% |
| 1088 | GOBP\_PHOSPHOLIPASE\_C\_ACTIVATING\_G\_PROTEIN\_COUPLED\_RECEPTOR\_SIGNALING\_PATHWAY |  | 91 | -0.28 | -1.21 | 0.135 | 0.437 | 1.000 | 3856 | tags=38%, list=30%, signal=54% |
| 1089 | GOBP\_INTRINSIC\_APOPTOTIC\_SIGNALING\_PATHWAY |  | 253 | -0.24 | -1.21 | 0.079 | 0.438 | 1.000 | 3296 | tags=31%, list=26%, signal=41% |
| 1090 | GOBP\_INTRACELLULAR\_ESTROGEN\_RECEPTOR\_SIGNALING\_PATHWAY |  | 47 | -0.32 | -1.21 | 0.177 | 0.438 | 1.000 | 1756 | tags=23%, list=14%, signal=27% |
| 1091 | GOBP\_REGULATION\_OF\_LEUKOCYTE\_ADHESION\_TO\_VASCULAR\_ENDOTHELIAL\_CELL |  | 26 | -0.37 | -1.21 | 0.195 | 0.438 | 1.000 | 442 | tags=15%, list=3%, signal=16% |
| 1092 | GOBP\_CELL\_MIGRATION\_INVOLVED\_IN\_HEART\_DEVELOPMENT |  | 18 | -0.40 | -1.21 | 0.216 | 0.438 | 1.000 | 1626 | tags=28%, list=13%, signal=32% |
| 1093 | GOBP\_ANATOMICAL\_STRUCTURE\_HOMEOSTASIS |  | 259 | -0.24 | -1.21 | 0.077 | 0.438 | 1.000 | 3846 | tags=36%, list=30%, signal=51% |
| 1094 | GOBP\_REGULATION\_OF\_CARTILAGE\_DEVELOPMENT |  | 57 | -0.31 | -1.21 | 0.168 | 0.438 | 1.000 | 3679 | tags=39%, list=29%, signal=54% |
| 1095 | GOBP\_CELLULAR\_RESPONSE\_TO\_CARBOHYDRATE\_STIMULUS |  | 120 | -0.27 | -1.21 | 0.122 | 0.438 | 1.000 | 3867 | tags=38%, list=30%, signal=54% |
| 1096 | GOBP\_POSITIVE\_REGULATION\_OF\_HEART\_GROWTH |  | 30 | -0.35 | -1.20 | 0.216 | 0.441 | 1.000 | 1947 | tags=23%, list=15%, signal=27% |
| 1097 | GOBP\_POSITIVE\_REGULATION\_OF\_MEIOTIC\_CELL\_CYCLE |  | 18 | -0.40 | -1.20 | 0.242 | 0.441 | 1.000 | 3401 | tags=50%, list=26%, signal=68% |
| 1098 | GOBP\_V\_D\_J\_RECOMBINATION |  | 20 | -0.40 | -1.20 | 0.200 | 0.441 | 1.000 | 732 | tags=20%, list=6%, signal=21% |
| 1099 | GOBP\_GLUTAMATE\_RECEPTOR\_SIGNALING\_PATHWAY |  | 45 | -0.32 | -1.20 | 0.170 | 0.442 | 1.000 | 5067 | tags=58%, list=39%, signal=95% |
| 1100 | GOBP\_SODIUM\_ION\_IMPORT\_ACROSS\_PLASMA\_MEMBRANE |  | 18 | -0.40 | -1.20 | 0.204 | 0.442 | 1.000 | 2160 | tags=28%, list=17%, signal=33% |
| 1101 | GOBP\_NEGATIVE\_REGULATION\_OF\_STRIATED\_MUSCLE\_CELL\_APOPTOTIC\_PROCESS |  | 19 | -0.39 | -1.20 | 0.225 | 0.443 | 1.000 | 1713 | tags=26%, list=13%, signal=30% |
| 1102 | GOBP\_EMBRYONIC\_DIGESTIVE\_TRACT\_MORPHOGENESIS |  | 15 | -0.42 | -1.20 | 0.238 | 0.443 | 1.000 | 4356 | tags=53%, list=34%, signal=80% |
| 1103 | GOBP\_REGULATION\_OF\_CELL\_PROJECTION\_ASSEMBLY |  | 175 | -0.26 | -1.20 | 0.111 | 0.443 | 1.000 | 3312 | tags=31%, list=26%, signal=41% |
| 1104 | GOBP\_RESPONSE\_TO\_RADIATION |  | 359 | -0.23 | -1.20 | 0.061 | 0.443 | 1.000 | 2501 | tags=23%, list=19%, signal=28% |
| 1105 | GOBP\_CAMP\_MEDIATED\_SIGNALING |  | 43 | -0.32 | -1.20 | 0.188 | 0.446 | 1.000 | 4344 | tags=42%, list=34%, signal=63% |
| 1106 | GOBP\_NERVE\_DEVELOPMENT |  | 67 | -0.30 | -1.20 | 0.166 | 0.446 | 1.000 | 3925 | tags=37%, list=30%, signal=53% |
| 1107 | GOBP\_CARBOXYLIC\_ACID\_TRANSPORT |  | 217 | -0.25 | -1.20 | 0.096 | 0.447 | 1.000 | 3004 | tags=28%, list=23%, signal=35% |
| 1108 | GOBP\_RECEPTOR\_INTERNALIZATION |  | 96 | -0.28 | -1.20 | 0.166 | 0.447 | 1.000 | 2718 | tags=29%, list=21%, signal=37% |
| 1109 | GOBP\_REGULATION\_OF\_CELL\_CYCLE\_G1\_S\_PHASE\_TRANSITION |  | 133 | -0.26 | -1.20 | 0.131 | 0.447 | 1.000 | 1174 | tags=15%, list=9%, signal=16% |
| 1110 | GOBP\_POSITIVE\_REGULATION\_OF\_B\_CELL\_PROLIFERATION |  | 33 | -0.35 | -1.20 | 0.215 | 0.448 | 1.000 | 1874 | tags=24%, list=15%, signal=28% |
| 1111 | GOBP\_RECOMBINATIONAL\_REPAIR |  | 120 | -0.27 | -1.20 | 0.131 | 0.448 | 1.000 | 1444 | tags=18%, list=11%, signal=20% |
| 1112 | GOBP\_INTERLEUKIN\_5\_PRODUCTION |  | 18 | -0.40 | -1.20 | 0.218 | 0.448 | 1.000 | 4105 | tags=50%, list=32%, signal=73% |
| 1113 | GOBP\_MACROPHAGE\_ACTIVATION |  | 89 | -0.28 | -1.20 | 0.179 | 0.448 | 1.000 | 2000 | tags=25%, list=16%, signal=29% |
| 1114 | GOBP\_PHOSPHOLIPID\_DEPHOSPHORYLATION |  | 38 | -0.33 | -1.20 | 0.218 | 0.448 | 1.000 | 3443 | tags=39%, list=27%, signal=54% |
| 1115 | GOBP\_RESPONSE\_TO\_MANGANESE\_ION |  | 17 | -0.41 | -1.20 | 0.243 | 0.447 | 1.000 | 3592 | tags=47%, list=28%, signal=65% |
| 1116 | GOBP\_CD4\_POSITIVE\_OR\_CD8\_POSITIVE\_ALPHA\_BETA\_T\_CELL\_LINEAGE\_COMMITMENT |  | 18 | -0.40 | -1.20 | 0.239 | 0.447 | 1.000 | 2590 | tags=33%, list=20%, signal=42% |
| 1117 | GOBP\_POSITIVE\_REGULATION\_OF\_VIRAL\_PROCESS |  | 56 | -0.31 | -1.20 | 0.194 | 0.447 | 1.000 | 2607 | tags=30%, list=20%, signal=38% |
| 1118 | GOBP\_I\_KAPPAB\_KINASE\_NF\_KAPPAB\_SIGNALING |  | 231 | -0.24 | -1.20 | 0.103 | 0.447 | 1.000 | 2254 | tags=23%, list=17%, signal=28% |
| 1119 | GOBP\_CELLULAR\_RESPONSE\_TO\_TOPOLOGICALLY\_INCORRECT\_PROTEIN |  | 93 | -0.28 | -1.20 | 0.138 | 0.446 | 1.000 | 4073 | tags=41%, list=32%, signal=59% |
| 1120 | GOBP\_NEGATIVE\_REGULATION\_OF\_CELL\_GROWTH |  | 164 | -0.26 | -1.20 | 0.115 | 0.447 | 1.000 | 4062 | tags=40%, list=31%, signal=57% |
| 1121 | GOBP\_RESPONSE\_TO\_ACETYLCHOLINE |  | 29 | -0.35 | -1.20 | 0.200 | 0.447 | 1.000 | 2254 | tags=31%, list=17%, signal=38% |
| 1122 | GOBP\_REGULATION\_OF\_NEURON\_PROJECTION\_DEVELOPMENT |  | 358 | -0.23 | -1.20 | 0.060 | 0.447 | 1.000 | 3240 | tags=29%, list=25%, signal=38% |
| 1123 | GOBP\_NEGATIVE\_REGULATION\_OF\_LOCOMOTION |  | 276 | -0.24 | -1.20 | 0.082 | 0.447 | 1.000 | 2801 | tags=25%, list=22%, signal=32% |
| 1124 | GOBP\_RESPONSE\_TO\_LIGHT\_STIMULUS |  | 249 | -0.24 | -1.20 | 0.103 | 0.448 | 1.000 | 2600 | tags=24%, list=20%, signal=30% |
| 1125 | GOBP\_CELLULAR\_RESPONSE\_TO\_CAMP |  | 50 | -0.31 | -1.20 | 0.172 | 0.448 | 1.000 | 5202 | tags=56%, list=40%, signal=93% |
| 1126 | GOBP\_CELL\_CELL\_SIGNALING\_BY\_WNT |  | 346 | -0.24 | -1.20 | 0.066 | 0.448 | 1.000 | 2196 | tags=21%, list=17%, signal=25% |
| 1127 | GOBP\_REGULATION\_OF\_T\_CELL\_DIFFERENTIATION\_IN\_THYMUS |  | 24 | -0.37 | -1.20 | 0.217 | 0.448 | 1.000 | 5086 | tags=75%, list=39%, signal=124% |
| 1128 | GOBP\_SMOOTH\_MUSCLE\_TISSUE\_DEVELOPMENT |  | 21 | -0.38 | -1.20 | 0.214 | 0.447 | 1.000 | 4118 | tags=52%, list=32%, signal=77% |
| 1129 | GOBP\_ADENYLATE\_CYCLASE\_ACTIVATING\_G\_PROTEIN\_COUPLED\_RECEPTOR\_SIGNALING\_PATHWAY |  | 128 | -0.27 | -1.20 | 0.144 | 0.448 | 1.000 | 3369 | tags=30%, list=26%, signal=40% |
| 1130 | GOBP\_NEGATIVE\_REGULATION\_OF\_CD4\_POSITIVE\_ALPHA\_BETA\_T\_CELL\_ACTIVATION |  | 23 | -0.38 | -1.20 | 0.218 | 0.450 | 1.000 | 4200 | tags=43%, list=33%, signal=64% |
| 1131 | GOBP\_DNA\_CONFORMATION\_CHANGE |  | 269 | -0.24 | -1.20 | 0.080 | 0.451 | 1.000 | 2936 | tags=26%, list=23%, signal=33% |
| 1132 | GOBP\_RESPONSE\_TO\_VITAMIN\_D |  | 34 | -0.34 | -1.19 | 0.191 | 0.451 | 1.000 | 1910 | tags=26%, list=15%, signal=31% |
| 1133 | GOBP\_L\_ALPHA\_AMINO\_ACID\_TRANSMEMBRANE\_TRANSPORT |  | 51 | -0.31 | -1.19 | 0.182 | 0.452 | 1.000 | 4808 | tags=55%, list=37%, signal=87% |
| 1134 | GOBP\_REGULATION\_OF\_CELL\_SUBSTRATE\_JUNCTION\_ORGANIZATION |  | 63 | -0.30 | -1.19 | 0.170 | 0.452 | 1.000 | 3821 | tags=44%, list=30%, signal=63% |
| 1135 | GOBP\_LYMPHOCYTE\_CHEMOTAXIS |  | 51 | -0.31 | -1.19 | 0.186 | 0.452 | 1.000 | 4069 | tags=47%, list=32%, signal=68% |
| 1136 | GOBP\_NEGATIVE\_REGULATION\_OF\_PROTEOLYSIS\_INVOLVED\_IN\_CELLULAR\_PROTEIN\_CATABOLIC\_PROCESS |  | 52 | -0.31 | -1.19 | 0.192 | 0.452 | 1.000 | 3215 | tags=37%, list=25%, signal=48% |
| 1137 | GOBP\_MUSCLE\_CELL\_DIFFERENTIATION |  | 294 | -0.24 | -1.19 | 0.106 | 0.452 | 1.000 | 2204 | tags=22%, list=17%, signal=26% |
| 1138 | GOBP\_NEGATIVE\_REGULATION\_OF\_WOUND\_HEALING |  | 64 | -0.29 | -1.19 | 0.181 | 0.454 | 1.000 | 3219 | tags=36%, list=25%, signal=48% |
| 1139 | GOBP\_NEURON\_PROJECTION\_GUIDANCE |  | 191 | -0.25 | -1.19 | 0.131 | 0.454 | 1.000 | 2810 | tags=26%, list=22%, signal=33% |
| 1140 | GOBP\_REGULATION\_OF\_CELL\_DIVISION |  | 150 | -0.26 | -1.19 | 0.131 | 0.455 | 1.000 | 798 | tags=13%, list=6%, signal=13% |
| 1141 | GOBP\_REGULATION\_OF\_TELOMERE\_MAINTENANCE |  | 67 | -0.29 | -1.19 | 0.178 | 0.456 | 1.000 | 2311 | tags=22%, list=18%, signal=27% |
| 1142 | GOBP\_NEURON\_DEATH |  | 310 | -0.24 | -1.19 | 0.102 | 0.458 | 1.000 | 2848 | tags=26%, list=22%, signal=32% |
| 1143 | GOBP\_KERATINOCYTE\_DIFFERENTIATION |  | 96 | -0.28 | -1.19 | 0.151 | 0.459 | 1.000 | 3385 | tags=33%, list=26%, signal=45% |
| 1144 | GOBP\_NEURON\_APOPTOTIC\_PROCESS |  | 212 | -0.25 | -1.19 | 0.124 | 0.458 | 1.000 | 1699 | tags=17%, list=13%, signal=20% |
| 1145 | GOBP\_SEQUESTERING\_OF\_TRIGLYCERIDE |  | 15 | -0.42 | -1.19 | 0.235 | 0.459 | 1.000 | 3032 | tags=47%, list=24%, signal=61% |
| 1146 | GOBP\_EMBRYONIC\_PLACENTA\_MORPHOGENESIS |  | 25 | -0.37 | -1.19 | 0.230 | 0.459 | 1.000 | 449 | tags=12%, list=3%, signal=12% |
| 1147 | GOBP\_COLUMNAR\_CUBOIDAL\_EPITHELIAL\_CELL\_DEVELOPMENT |  | 39 | -0.33 | -1.19 | 0.198 | 0.459 | 1.000 | 2881 | tags=36%, list=22%, signal=46% |
| 1148 | GOBP\_DNA\_DAMAGE\_RESPONSE\_SIGNAL\_TRANSDUCTION\_RESULTING\_IN\_TRANSCRIPTION |  | 15 | -0.42 | -1.19 | 0.257 | 0.459 | 1.000 | 175 | tags=13%, list=1%, signal=14% |
| 1149 | GOBP\_REGULATION\_OF\_CENTRIOLE\_REPLICATION |  | 17 | -0.40 | -1.19 | 0.236 | 0.459 | 1.000 | 3420 | tags=35%, list=27%, signal=48% |
| 1150 | GOBP\_CONNECTIVE\_TISSUE\_DEVELOPMENT |  | 222 | -0.24 | -1.19 | 0.110 | 0.459 | 1.000 | 4421 | tags=41%, list=34%, signal=61% |
| 1151 | GOBP\_REGULATION\_OF\_SMALL\_MOLECULE\_METABOLIC\_PROCESS |  | 280 | -0.24 | -1.19 | 0.096 | 0.461 | 1.000 | 2188 | tags=22%, list=17%, signal=26% |
| 1152 | GOBP\_REGULATION\_OF\_UBIQUITIN\_DEPENDENT\_PROTEIN\_CATABOLIC\_PROCESS |  | 135 | -0.26 | -1.19 | 0.142 | 0.460 | 1.000 | 2567 | tags=29%, list=20%, signal=36% |
| 1153 | GOBP\_REGULATION\_OF\_BIOMINERALIZATION |  | 84 | -0.28 | -1.19 | 0.178 | 0.461 | 1.000 | 2074 | tags=25%, list=16%, signal=30% |
| 1154 | GOBP\_DENDRITIC\_SPINE\_DEVELOPMENT |  | 74 | -0.29 | -1.19 | 0.183 | 0.461 | 1.000 | 3215 | tags=32%, list=25%, signal=43% |
| 1155 | GOBP\_TISSUE\_REMODELING |  | 149 | -0.26 | -1.19 | 0.140 | 0.461 | 1.000 | 4162 | tags=40%, list=32%, signal=59% |
| 1156 | GOBP\_REGULATION\_OF\_INFLAMMATORY\_RESPONSE |  | 300 | -0.24 | -1.19 | 0.105 | 0.461 | 1.000 | 3700 | tags=32%, list=29%, signal=44% |
| 1157 | GOBP\_REGULATION\_OF\_POTASSIUM\_ION\_TRANSPORT |  | 80 | -0.28 | -1.19 | 0.188 | 0.464 | 1.000 | 4005 | tags=43%, list=31%, signal=61% |
| 1158 | GOBP\_SENSORY\_PERCEPTION\_OF\_SMELL |  | 59 | -0.30 | -1.19 | 0.197 | 0.463 | 1.000 | 4122 | tags=39%, list=32%, signal=57% |
| 1159 | GOBP\_NEGATIVE\_REGULATION\_OF\_INTRINSIC\_APOPTOTIC\_SIGNALING\_PATHWAY\_IN\_RESPONSE\_TO\_DNA\_DAMAGE |  | 26 | -0.36 | -1.19 | 0.232 | 0.464 | 1.000 | 606 | tags=15%, list=5%, signal=16% |
| 1160 | GOBP\_NEGATIVE\_REGULATION\_OF\_DEFENSE\_RESPONSE |  | 199 | -0.25 | -1.19 | 0.122 | 0.463 | 1.000 | 3441 | tags=29%, list=27%, signal=38% |
| 1161 | GOBP\_CELLULAR\_RESPONSE\_TO\_UV |  | 72 | -0.29 | -1.18 | 0.193 | 0.464 | 1.000 | 2482 | tags=26%, list=19%, signal=32% |
| 1162 | GOBP\_NEGATIVE\_REGULATION\_OF\_CELL\_CYCLE\_G2\_M\_PHASE\_TRANSITION |  | 49 | -0.31 | -1.18 | 0.210 | 0.467 | 1.000 | 3123 | tags=31%, list=24%, signal=40% |
| 1163 | GOBP\_MACROPHAGE\_DIFFERENTIATION |  | 39 | -0.32 | -1.18 | 0.220 | 0.467 | 1.000 | 1844 | tags=26%, list=14%, signal=30% |
| 1164 | GOBP\_STEROID\_HORMONE\_MEDIATED\_SIGNALING\_PATHWAY |  | 120 | -0.26 | -1.18 | 0.152 | 0.468 | 1.000 | 1878 | tags=22%, list=15%, signal=25% |
| 1165 | GOBP\_BIOLOGICAL\_PROCESS\_INVOLVED\_IN\_INTERACTION\_WITH\_HOST |  | 172 | -0.25 | -1.18 | 0.140 | 0.468 | 1.000 | 3179 | tags=31%, list=25%, signal=40% |
| 1166 | GOBP\_MITOTIC\_SISTER\_CHROMATID\_COHESION |  | 20 | -0.39 | -1.18 | 0.244 | 0.471 | 1.000 | 3392 | tags=40%, list=26%, signal=54% |
| 1167 | GOBP\_REGULATION\_OF\_GLUCAN\_BIOSYNTHETIC\_PROCESS |  | 23 | -0.37 | -1.18 | 0.238 | 0.473 | 1.000 | 1745 | tags=30%, list=14%, signal=35% |
| 1168 | GOBP\_CELLULAR\_RESPONSE\_TO\_EXTERNAL\_STIMULUS |  | 262 | -0.24 | -1.18 | 0.113 | 0.473 | 1.000 | 2927 | tags=29%, list=23%, signal=36% |
| 1169 | GOBP\_RESPONSE\_TO\_LEUKEMIA\_INHIBITORY\_FACTOR |  | 89 | -0.28 | -1.18 | 0.179 | 0.474 | 1.000 | 3371 | tags=34%, list=26%, signal=45% |
| 1170 | GOBP\_ECTODERM\_DEVELOPMENT |  | 19 | -0.39 | -1.18 | 0.263 | 0.474 | 1.000 | 1906 | tags=26%, list=15%, signal=31% |
| 1171 | GOBP\_CHEMICAL\_HOMEOSTASIS\_WITHIN\_A\_TISSUE |  | 15 | -0.41 | -1.18 | 0.236 | 0.473 | 1.000 | 3731 | tags=53%, list=29%, signal=75% |
| 1172 | GOBP\_POSITIVE\_REGULATION\_OF\_VASCULAR\_PERMEABILITY |  | 15 | -0.42 | -1.18 | 0.239 | 0.474 | 1.000 | 4977 | tags=80%, list=39%, signal=130% |
| 1173 | GOBP\_KIDNEY\_MESENCHYME\_DEVELOPMENT |  | 16 | -0.41 | -1.18 | 0.243 | 0.474 | 1.000 | 571 | tags=13%, list=4%, signal=13% |
| 1174 | GOBP\_RESPONSE\_TO\_DOPAMINE |  | 69 | -0.29 | -1.18 | 0.204 | 0.473 | 1.000 | 3003 | tags=30%, list=23%, signal=39% |
| 1175 | GOBP\_NEUTROPHIL\_MIGRATION |  | 112 | -0.27 | -1.18 | 0.176 | 0.473 | 1.000 | 3548 | tags=37%, list=28%, signal=50% |
| 1176 | GOBP\_MICROVILLUS\_ORGANIZATION |  | 28 | -0.35 | -1.18 | 0.223 | 0.474 | 1.000 | 3909 | tags=43%, list=30%, signal=61% |
| 1177 | GOBP\_CYTOKINE\_MEDIATED\_SIGNALING\_PATHWAY |  | 402 | -0.23 | -1.18 | 0.087 | 0.474 | 1.000 | 3731 | tags=33%, list=29%, signal=45% |
| 1178 | GOBP\_T\_HELPER\_17\_TYPE\_IMMUNE\_RESPONSE |  | 31 | -0.35 | -1.18 | 0.210 | 0.474 | 1.000 | 1800 | tags=19%, list=14%, signal=22% |
| 1179 | GOBP\_NEGATIVE\_REGULATION\_OF\_COAGULATION |  | 47 | -0.31 | -1.18 | 0.220 | 0.474 | 1.000 | 3219 | tags=36%, list=25%, signal=48% |
| 1180 | GOBP\_POSITIVE\_REGULATION\_OF\_KERATINOCYTE\_DIFFERENTIATION |  | 17 | -0.40 | -1.18 | 0.241 | 0.474 | 1.000 | 1756 | tags=29%, list=14%, signal=34% |
| 1181 | GOBP\_NEGATIVE\_REGULATION\_OF\_PROTEIN\_LOCALIZATION\_TO\_NUCLEUS |  | 25 | -0.36 | -1.18 | 0.247 | 0.474 | 1.000 | 2280 | tags=36%, list=18%, signal=44% |
| 1182 | GOBP\_CELL\_DIFFERENTIATION\_IN\_HINDBRAIN |  | 18 | -0.39 | -1.18 | 0.265 | 0.475 | 1.000 | 4380 | tags=50%, list=34%, signal=76% |
| 1183 | GOBP\_REGULATION\_OF\_SODIUM\_ION\_TRANSMEMBRANE\_TRANSPORT |  | 48 | -0.31 | -1.18 | 0.220 | 0.474 | 1.000 | 2608 | tags=31%, list=20%, signal=39% |
| 1184 | GOBP\_REGULATION\_OF\_TUBE\_SIZE |  | 131 | -0.26 | -1.18 | 0.140 | 0.474 | 1.000 | 4301 | tags=44%, list=33%, signal=66% |
| 1185 | GOBP\_POSITIVE\_REGULATION\_OF\_NOTCH\_SIGNALING\_PATHWAY |  | 33 | -0.33 | -1.18 | 0.224 | 0.475 | 1.000 | 3108 | tags=33%, list=24%, signal=44% |
| 1186 | GOBP\_POSITIVE\_REGULATION\_OF\_ANIMAL\_ORGAN\_MORPHOGENESIS |  | 26 | -0.36 | -1.18 | 0.250 | 0.476 | 1.000 | 3289 | tags=35%, list=25%, signal=46% |
| 1187 | GOBP\_PANCREAS\_DEVELOPMENT |  | 69 | -0.28 | -1.18 | 0.176 | 0.477 | 1.000 | 2969 | tags=30%, list=23%, signal=39% |
| 1188 | GOBP\_POSITIVE\_REGULATION\_OF\_CELL\_PROJECTION\_ORGANIZATION |  | 289 | -0.24 | -1.17 | 0.109 | 0.478 | 1.000 | 3373 | tags=30%, list=26%, signal=40% |
| 1189 | GOBP\_T\_CELL\_ACTIVATION\_INVOLVED\_IN\_IMMUNE\_RESPONSE |  | 93 | -0.27 | -1.17 | 0.177 | 0.479 | 1.000 | 4449 | tags=42%, list=34%, signal=64% |
| 1190 | GOBP\_T\_HELPER\_17\_CELL\_DIFFERENTIATION |  | 21 | -0.38 | -1.17 | 0.233 | 0.479 | 1.000 | 4782 | tags=52%, list=37%, signal=83% |
| 1191 | GOBP\_CELLULAR\_RESPONSE\_TO\_STEROL |  | 18 | -0.40 | -1.17 | 0.252 | 0.481 | 1.000 | 2791 | tags=44%, list=22%, signal=57% |
| 1192 | GOBP\_RETINA\_HOMEOSTASIS |  | 52 | -0.31 | -1.17 | 0.197 | 0.481 | 1.000 | 2833 | tags=31%, list=22%, signal=39% |
| 1193 | GOBP\_FIBROBLAST\_GROWTH\_FACTOR\_RECEPTOR\_SIGNALING\_PATHWAY |  | 61 | -0.29 | -1.17 | 0.198 | 0.481 | 1.000 | 1840 | tags=21%, list=14%, signal=25% |
| 1194 | GOBP\_STRESS\_FIBER\_ASSEMBLY |  | 96 | -0.27 | -1.17 | 0.177 | 0.481 | 1.000 | 1803 | tags=21%, list=14%, signal=24% |
| 1195 | GOBP\_CELLULAR\_RESPONSE\_TO\_CALCIUM\_ION |  | 64 | -0.30 | -1.17 | 0.192 | 0.481 | 1.000 | 1626 | tags=20%, list=13%, signal=23% |
| 1196 | GOBP\_TYPE\_I\_INTERFERON\_PRODUCTION |  | 81 | -0.28 | -1.17 | 0.188 | 0.483 | 1.000 | 2275 | tags=27%, list=18%, signal=33% |
| 1197 | GOBP\_NEGATIVE\_REGULATION\_OF\_PROTEIN\_SECRETION |  | 48 | -0.31 | -1.17 | 0.215 | 0.484 | 1.000 | 3304 | tags=35%, list=26%, signal=47% |
| 1198 | GOBP\_MITOTIC\_G1\_S\_TRANSITION\_CHECKPOINT\_SIGNALING |  | 26 | -0.35 | -1.17 | 0.244 | 0.483 | 1.000 | 3420 | tags=38%, list=27%, signal=52% |
| 1199 | GOBP\_POSITIVE\_REGULATION\_OF\_DNA\_BIOSYNTHETIC\_PROCESS |  | 66 | -0.29 | -1.17 | 0.193 | 0.483 | 1.000 | 693 | tags=14%, list=5%, signal=14% |
| 1200 | GOBP\_REGULATION\_OF\_CELL\_MATRIX\_ADHESION |  | 107 | -0.27 | -1.17 | 0.161 | 0.483 | 1.000 | 3821 | tags=39%, list=30%, signal=55% |
| 1201 | GOBP\_REGULATION\_OF\_FILOPODIUM\_ASSEMBLY |  | 43 | -0.31 | -1.17 | 0.222 | 0.483 | 1.000 | 2820 | tags=28%, list=22%, signal=36% |
| 1202 | GOBP\_REGULATION\_OF\_CANONICAL\_WNT\_SIGNALING\_PATHWAY |  | 189 | -0.25 | -1.17 | 0.131 | 0.484 | 1.000 | 2246 | tags=24%, list=17%, signal=28% |
| 1203 | GOBP\_PROGRAMMED\_NECROTIC\_CELL\_DEATH |  | 33 | -0.34 | -1.17 | 0.234 | 0.484 | 1.000 | 2903 | tags=33%, list=23%, signal=43% |
| 1204 | GOBP\_ERBB\_SIGNALING\_PATHWAY |  | 103 | -0.27 | -1.17 | 0.198 | 0.483 | 1.000 | 3821 | tags=39%, list=30%, signal=55% |
| 1205 | GOBP\_REGULATION\_OF\_TRANSCRIPTION\_REGULATORY\_REGION\_DNA\_BINDING |  | 47 | -0.31 | -1.17 | 0.205 | 0.483 | 1.000 | 1378 | tags=23%, list=11%, signal=26% |
| 1206 | GOBP\_CYTOKINE\_PRODUCTION\_INVOLVED\_IN\_IMMUNE\_RESPONSE |  | 94 | -0.27 | -1.17 | 0.180 | 0.483 | 1.000 | 2101 | tags=23%, list=16%, signal=28% |
| 1207 | GOBP\_RESPONSE\_TO\_WOUNDING |  | 485 | -0.22 | -1.17 | 0.078 | 0.483 | 1.000 | 3303 | tags=28%, list=26%, signal=36% |
| 1208 | GOBP\_GAMETE\_GENERATION |  | 493 | -0.22 | -1.17 | 0.091 | 0.483 | 1.000 | 3030 | tags=26%, list=23%, signal=32% |
| 1209 | GOBP\_REGULATION\_OF\_TRANSCRIPTION\_BY\_RNA\_POLYMERASE\_III |  | 24 | -0.36 | -1.17 | 0.252 | 0.483 | 1.000 | 3773 | tags=54%, list=29%, signal=76% |
| 1210 | GOBP\_CEREBELLAR\_CORTEX\_FORMATION |  | 21 | -0.37 | -1.17 | 0.240 | 0.483 | 1.000 | 3242 | tags=38%, list=25%, signal=51% |
| 1211 | GOBP\_CRANIAL\_NERVE\_DEVELOPMENT |  | 44 | -0.31 | -1.17 | 0.234 | 0.484 | 1.000 | 3925 | tags=39%, list=30%, signal=55% |
| 1212 | GOBP\_NEGATIVE\_REGULATION\_OF\_INTERLEUKIN\_8\_PRODUCTION |  | 15 | -0.41 | -1.17 | 0.274 | 0.483 | 1.000 | 2161 | tags=47%, list=17%, signal=56% |
| 1213 | GOBP\_RESPONSE\_TO\_PAIN |  | 26 | -0.35 | -1.17 | 0.237 | 0.483 | 1.000 | 1404 | tags=23%, list=11%, signal=26% |
| 1214 | GOBP\_REGULATION\_OF\_ACTIN\_NUCLEATION |  | 27 | -0.35 | -1.17 | 0.231 | 0.484 | 1.000 | 2586 | tags=41%, list=20%, signal=51% |
| 1215 | GOBP\_LEUKOCYTE\_CHEMOTAXIS |  | 208 | -0.24 | -1.17 | 0.153 | 0.484 | 1.000 | 3700 | tags=35%, list=29%, signal=48% |
| 1216 | GOBP\_REGULATION\_OF\_CALCIUM\_ION\_TRANSPORT |  | 204 | -0.24 | -1.17 | 0.146 | 0.484 | 1.000 | 3118 | tags=27%, list=24%, signal=36% |
| 1217 | GOBP\_POSITIVE\_REGULATION\_OF\_MAPK\_CASCADE |  | 415 | -0.22 | -1.17 | 0.084 | 0.484 | 1.000 | 4283 | tags=38%, list=33%, signal=54% |
| 1218 | GOBP\_EPITHELIAL\_CELL\_MORPHOGENESIS |  | 25 | -0.36 | -1.17 | 0.243 | 0.483 | 1.000 | 4230 | tags=52%, list=33%, signal=77% |
| 1219 | GOBP\_NEUROINFLAMMATORY\_RESPONSE |  | 33 | -0.34 | -1.17 | 0.232 | 0.484 | 1.000 | 1933 | tags=24%, list=15%, signal=28% |
| 1220 | GOBP\_ENTRY\_INTO\_HOST |  | 140 | -0.25 | -1.17 | 0.170 | 0.484 | 1.000 | 3859 | tags=36%, list=30%, signal=51% |
| 1221 | GOBP\_PROSTATE\_GLAND\_DEVELOPMENT |  | 43 | -0.31 | -1.17 | 0.225 | 0.484 | 1.000 | 1554 | tags=21%, list=12%, signal=24% |
| 1222 | GOBP\_PHASIC\_SMOOTH\_MUSCLE\_CONTRACTION |  | 20 | -0.38 | -1.17 | 0.264 | 0.484 | 1.000 | 3046 | tags=45%, list=24%, signal=59% |
| 1223 | GOBP\_JNK\_CASCADE |  | 150 | -0.25 | -1.17 | 0.153 | 0.484 | 1.000 | 2567 | tags=25%, list=20%, signal=31% |
| 1224 | GOBP\_RESPONSE\_TO\_MECHANICAL\_STIMULUS |  | 177 | -0.24 | -1.17 | 0.156 | 0.485 | 1.000 | 3137 | tags=29%, list=24%, signal=38% |
| 1225 | GOBP\_NEGATIVE\_REGULATION\_OF\_NEURON\_PROJECTION\_DEVELOPMENT |  | 113 | -0.26 | -1.17 | 0.181 | 0.485 | 1.000 | 1917 | tags=24%, list=15%, signal=28% |
| 1226 | GOBP\_NEGATIVE\_REGULATION\_OF\_CELL\_PROJECTION\_ORGANIZATION |  | 152 | -0.25 | -1.17 | 0.158 | 0.485 | 1.000 | 3703 | tags=39%, list=29%, signal=54% |
| 1227 | GOBP\_CD4\_POSITIVE\_ALPHA\_BETA\_T\_CELL\_ACTIVATION |  | 79 | -0.28 | -1.17 | 0.196 | 0.486 | 1.000 | 2085 | tags=23%, list=16%, signal=27% |
| 1228 | GOBP\_TROPHECTODERMAL\_CELL\_DIFFERENTIATION |  | 16 | -0.40 | -1.17 | 0.269 | 0.486 | 1.000 | 434 | tags=13%, list=3%, signal=13% |
| 1229 | GOBP\_REGULATION\_OF\_G\_PROTEIN\_COUPLED\_RECEPTOR\_SIGNALING\_PATHWAY |  | 122 | -0.26 | -1.17 | 0.178 | 0.486 | 1.000 | 2535 | tags=25%, list=20%, signal=30% |
| 1230 | GOBP\_REGULATION\_OF\_OSSIFICATION |  | 100 | -0.27 | -1.16 | 0.189 | 0.487 | 1.000 | 2074 | tags=24%, list=16%, signal=28% |
| 1231 | GOBP\_MESODERM\_MORPHOGENESIS |  | 56 | -0.30 | -1.16 | 0.220 | 0.488 | 1.000 | 2997 | tags=38%, list=23%, signal=49% |
| 1232 | GOBP\_INTERFERON\_ALPHA\_PRODUCTION |  | 23 | -0.36 | -1.16 | 0.253 | 0.488 | 1.000 | 2275 | tags=35%, list=18%, signal=42% |
| 1233 | GOBP\_NEGATIVE\_REGULATION\_OF\_OSTEOCLAST\_DIFFERENTIATION |  | 27 | -0.35 | -1.16 | 0.247 | 0.488 | 1.000 | 1803 | tags=30%, list=14%, signal=34% |
| 1234 | GOBP\_ADRENERGIC\_RECEPTOR\_SIGNALING\_PATHWAY |  | 26 | -0.35 | -1.16 | 0.262 | 0.488 | 1.000 | 2112 | tags=31%, list=16%, signal=37% |
| 1235 | GOBP\_NUCLEAR\_TRANSCRIBED\_MRNA\_POLY\_A\_TAIL\_SHORTENING |  | 22 | -0.37 | -1.16 | 0.262 | 0.488 | 1.000 | 1690 | tags=32%, list=13%, signal=37% |
| 1236 | GOBP\_POSITIVE\_REGULATION\_OF\_ATP\_METABOLIC\_PROCESS |  | 32 | -0.33 | -1.16 | 0.234 | 0.488 | 1.000 | 3488 | tags=41%, list=27%, signal=56% |
| 1237 | GOBP\_RESPONSE\_TO\_TEMPERATURE\_STIMULUS |  | 155 | -0.25 | -1.16 | 0.160 | 0.488 | 1.000 | 3001 | tags=29%, list=23%, signal=37% |
| 1238 | GOBP\_LAMELLIPODIUM\_ASSEMBLY |  | 57 | -0.29 | -1.16 | 0.211 | 0.488 | 1.000 | 3373 | tags=37%, list=26%, signal=50% |
| 1239 | GOBP\_HISTONE\_METHYLATION |  | 128 | -0.26 | -1.16 | 0.178 | 0.489 | 1.000 | 2940 | tags=27%, list=23%, signal=35% |
| 1240 | GOBP\_REGULATION\_OF\_VIRAL\_GENOME\_REPLICATION |  | 78 | -0.28 | -1.16 | 0.204 | 0.490 | 1.000 | 2607 | tags=27%, list=20%, signal=34% |
| 1241 | GOBP\_POSITIVE\_REGULATION\_OF\_KINASE\_ACTIVITY |  | 413 | -0.22 | -1.16 | 0.111 | 0.489 | 1.000 | 2848 | tags=25%, list=22%, signal=31% |
| 1242 | GOBP\_POSITIVE\_REGULATION\_OF\_HYDROLASE\_ACTIVITY |  | 470 | -0.22 | -1.16 | 0.094 | 0.490 | 1.000 | 3083 | tags=27%, list=24%, signal=34% |
| 1243 | GOBP\_REGULATION\_OF\_MYELINATION |  | 41 | -0.32 | -1.16 | 0.217 | 0.491 | 1.000 | 1376 | tags=20%, list=11%, signal=22% |
| 1244 | GOBP\_REGULATION\_OF\_NITRIC\_OXIDE\_METABOLIC\_PROCESS |  | 53 | -0.30 | -1.16 | 0.243 | 0.492 | 1.000 | 1626 | tags=21%, list=13%, signal=24% |
| 1245 | GOBP\_POSITIVE\_REGULATION\_OF\_INTRINSIC\_APOPTOTIC\_SIGNALING\_PATHWAY |  | 52 | -0.30 | -1.16 | 0.204 | 0.492 | 1.000 | 3155 | tags=38%, list=24%, signal=51% |
| 1246 | GOBP\_CELLULAR\_GLUCOSE\_HOMEOSTASIS |  | 123 | -0.26 | -1.16 | 0.183 | 0.491 | 1.000 | 3867 | tags=40%, list=30%, signal=56% |
| 1247 | GOBP\_POSITIVE\_REGULATION\_OF\_SMALL\_GTPASE\_MEDIATED\_SIGNAL\_TRANSDUCTION |  | 55 | -0.30 | -1.16 | 0.243 | 0.491 | 1.000 | 2378 | tags=25%, list=18%, signal=31% |
| 1248 | GOBP\_ORGANIC\_ACID\_TRANSPORT |  | 249 | -0.24 | -1.16 | 0.132 | 0.491 | 1.000 | 3004 | tags=27%, list=23%, signal=34% |
| 1249 | GOBP\_NEGATIVE\_REGULATION\_OF\_ORGANIC\_ACID\_TRANSPORT |  | 21 | -0.37 | -1.16 | 0.251 | 0.491 | 1.000 | 1931 | tags=33%, list=15%, signal=39% |
| 1250 | GOBP\_STRIATED\_MUSCLE\_CELL\_APOPTOTIC\_PROCESS |  | 35 | -0.33 | -1.16 | 0.234 | 0.491 | 1.000 | 1979 | tags=23%, list=15%, signal=27% |
| 1251 | GOBP\_POSITIVE\_REGULATION\_OF\_STRIATED\_MUSCLE\_CELL\_DIFFERENTIATION |  | 36 | -0.33 | -1.16 | 0.219 | 0.491 | 1.000 | 3961 | tags=44%, list=31%, signal=64% |
| 1252 | GOBP\_DSRNA\_PROCESSING |  | 46 | -0.31 | -1.16 | 0.232 | 0.492 | 1.000 | 1276 | tags=20%, list=10%, signal=22% |
| 1253 | GOBP\_POSITIVE\_REGULATION\_OF\_MYELOID\_LEUKOCYTE\_DIFFERENTIATION |  | 48 | -0.30 | -1.16 | 0.221 | 0.492 | 1.000 | 2460 | tags=27%, list=19%, signal=33% |
| 1254 | GOBP\_POSITIVE\_REGULATION\_OF\_ACUTE\_INFLAMMATORY\_RESPONSE |  | 22 | -0.37 | -1.16 | 0.253 | 0.493 | 1.000 | 2848 | tags=41%, list=22%, signal=52% |
| 1255 | GOBP\_EOSINOPHIL\_CHEMOTAXIS |  | 17 | -0.39 | -1.16 | 0.259 | 0.493 | 1.000 | 4756 | tags=53%, list=37%, signal=84% |
| 1256 | GOBP\_METANEPHRIC\_EPITHELIUM\_DEVELOPMENT |  | 20 | -0.38 | -1.16 | 0.274 | 0.492 | 1.000 | 817 | tags=15%, list=6%, signal=16% |
| 1257 | GOBP\_PROTEIN\_REFOLDING |  | 21 | -0.37 | -1.16 | 0.261 | 0.493 | 1.000 | 2341 | tags=33%, list=18%, signal=41% |
| 1258 | GOBP\_NEGATIVE\_REGULATION\_OF\_WNT\_SIGNALING\_PATHWAY |  | 125 | -0.26 | -1.16 | 0.173 | 0.494 | 1.000 | 2702 | tags=30%, list=21%, signal=37% |
| 1259 | GOBP\_NEGATIVE\_REGULATION\_OF\_CANONICAL\_WNT\_SIGNALING\_PATHWAY |  | 101 | -0.26 | -1.16 | 0.202 | 0.495 | 1.000 | 2649 | tags=30%, list=21%, signal=37% |
| 1260 | GOBP\_DEPHOSPHORYLATION |  | 327 | -0.23 | -1.16 | 0.136 | 0.495 | 1.000 | 2066 | tags=20%, list=16%, signal=24% |
| 1261 | GOBP\_RETINA\_DEVELOPMENT\_IN\_CAMERA\_TYPE\_EYE |  | 119 | -0.26 | -1.16 | 0.176 | 0.495 | 1.000 | 2639 | tags=24%, list=20%, signal=29% |
| 1262 | GOBP\_NEGATIVE\_REGULATION\_OF\_INNATE\_IMMUNE\_RESPONSE |  | 62 | -0.29 | -1.16 | 0.221 | 0.496 | 1.000 | 2288 | tags=23%, list=18%, signal=27% |
| 1263 | GOBP\_CYTOPLASMIC\_PATTERN\_RECOGNITION\_RECEPTOR\_SIGNALING\_PATHWAY |  | 51 | -0.30 | -1.16 | 0.207 | 0.497 | 1.000 | 1102 | tags=14%, list=9%, signal=15% |
| 1264 | GOBP\_LIPID\_EXPORT\_FROM\_CELL |  | 41 | -0.32 | -1.16 | 0.252 | 0.497 | 1.000 | 2391 | tags=29%, list=19%, signal=36% |
| 1265 | GOBP\_REGULATION\_OF\_PEPTIDYL\_SERINE\_PHOSPHORYLATION |  | 118 | -0.26 | -1.16 | 0.202 | 0.497 | 1.000 | 3773 | tags=34%, list=29%, signal=47% |
| 1266 | GOBP\_SEMAPHORIN\_PLEXIN\_SIGNALING\_PATHWAY\_INVOLVED\_IN\_NEURON\_PROJECTION\_GUIDANCE |  | 15 | -0.41 | -1.16 | 0.278 | 0.496 | 1.000 | 2220 | tags=33%, list=17%, signal=40% |
| 1267 | GOBP\_CENTRAL\_NERVOUS\_SYSTEM\_PROJECTION\_NEURON\_AXONOGENESIS |  | 20 | -0.38 | -1.15 | 0.269 | 0.499 | 1.000 | 2702 | tags=40%, list=21%, signal=51% |
| 1268 | GOBP\_POSITIVE\_REGULATION\_OF\_TELOMERE\_MAINTENANCE\_VIA\_TELOMERE\_LENGTHENING |  | 32 | -0.33 | -1.15 | 0.239 | 0.498 | 1.000 | 2311 | tags=25%, list=18%, signal=30% |
| 1269 | GOBP\_POSTSYNAPTIC\_SPECIALIZATION\_ORGANIZATION |  | 23 | -0.36 | -1.15 | 0.255 | 0.501 | 1.000 | 4093 | tags=43%, list=32%, signal=64% |
| 1270 | GOBP\_METANEPHRIC\_GLOMERULUS\_DEVELOPMENT |  | 15 | -0.41 | -1.15 | 0.277 | 0.501 | 1.000 | 3893 | tags=40%, list=30%, signal=57% |
| 1271 | GOBP\_AGING |  | 267 | -0.23 | -1.15 | 0.157 | 0.501 | 1.000 | 4162 | tags=37%, list=32%, signal=54% |
| 1272 | GOBP\_HINDBRAIN\_DEVELOPMENT |  | 133 | -0.26 | -1.15 | 0.187 | 0.501 | 1.000 | 2412 | tags=25%, list=19%, signal=30% |
| 1273 | GOBP\_MEMBRANE\_PROTEIN\_ECTODOMAIN\_PROTEOLYSIS |  | 39 | -0.32 | -1.15 | 0.242 | 0.501 | 1.000 | 2112 | tags=28%, list=16%, signal=34% |
| 1274 | GOBP\_ENDOCRINE\_SYSTEM\_DEVELOPMENT |  | 121 | -0.26 | -1.15 | 0.192 | 0.501 | 1.000 | 3027 | tags=26%, list=23%, signal=34% |
| 1275 | GOBP\_CHAPERONE\_MEDIATED\_AUTOPHAGY |  | 16 | -0.40 | -1.15 | 0.279 | 0.501 | 1.000 | 1340 | tags=31%, list=10%, signal=35% |
| 1276 | GOBP\_POSITIVE\_REGULATION\_OF\_DEVELOPMENTAL\_GROWTH |  | 142 | -0.25 | -1.15 | 0.165 | 0.501 | 1.000 | 3626 | tags=29%, list=28%, signal=40% |
| 1277 | GOBP\_EMBRYONIC\_DIGESTIVE\_TRACT\_DEVELOPMENT |  | 30 | -0.34 | -1.15 | 0.268 | 0.502 | 1.000 | 2969 | tags=30%, list=23%, signal=39% |
| 1278 | GOBP\_REACTIVE\_NITROGEN\_SPECIES\_METABOLIC\_PROCESS |  | 71 | -0.28 | -1.15 | 0.229 | 0.502 | 1.000 | 1626 | tags=18%, list=13%, signal=21% |
| 1279 | GOBP\_UTERUS\_DEVELOPMENT |  | 19 | -0.38 | -1.15 | 0.270 | 0.502 | 1.000 | 626 | tags=16%, list=5%, signal=17% |
| 1280 | GOBP\_DNA\_CATABOLIC\_PROCESS |  | 28 | -0.35 | -1.15 | 0.291 | 0.503 | 1.000 | 2636 | tags=32%, list=20%, signal=40% |
| 1281 | GOBP\_VIRAL\_LIFE\_CYCLE |  | 280 | -0.23 | -1.15 | 0.155 | 0.505 | 1.000 | 3179 | tags=31%, list=25%, signal=40% |
| 1282 | GOBP\_REGULATION\_OF\_EXTRINSIC\_APOPTOTIC\_SIGNALING\_PATHWAY |  | 133 | -0.25 | -1.15 | 0.189 | 0.507 | 1.000 | 3242 | tags=31%, list=25%, signal=41% |
| 1283 | GOBP\_RESPONSE\_TO\_BMP |  | 135 | -0.26 | -1.15 | 0.201 | 0.506 | 1.000 | 4414 | tags=44%, list=34%, signal=67% |
| 1284 | GOBP\_EXECUTION\_PHASE\_OF\_APOPTOSIS |  | 58 | -0.29 | -1.15 | 0.221 | 0.506 | 1.000 | 2636 | tags=28%, list=20%, signal=35% |
| 1285 | GOBP\_PROTEIN\_DNA\_COMPLEX\_SUBUNIT\_ORGANIZATION |  | 199 | -0.24 | -1.15 | 0.158 | 0.506 | 1.000 | 2248 | tags=20%, list=17%, signal=24% |
| 1286 | GOBP\_NEGATIVE\_REGULATION\_OF\_INFLAMMATORY\_RESPONSE |  | 124 | -0.26 | -1.15 | 0.214 | 0.506 | 1.000 | 3441 | tags=29%, list=27%, signal=39% |
| 1287 | GOBP\_CELL\_SURFACE\_RECEPTOR\_SIGNALING\_PATHWAY\_INVOLVED\_IN\_CELL\_CELL\_SIGNALING |  | 427 | -0.22 | -1.15 | 0.117 | 0.508 | 1.000 | 3027 | tags=26%, list=23%, signal=33% |
| 1288 | GOBP\_PROTEIN\_LOCALIZATION\_TO\_CELL\_CELL\_JUNCTION |  | 18 | -0.39 | -1.15 | 0.277 | 0.509 | 1.000 | 3137 | tags=44%, list=24%, signal=59% |
| 1289 | GOBP\_NEGATIVE\_REGULATION\_OF\_NEURON\_APOPTOTIC\_PROCESS |  | 124 | -0.26 | -1.15 | 0.210 | 0.509 | 1.000 | 1699 | tags=18%, list=13%, signal=20% |
| 1290 | GOBP\_ACTIVATION\_OF\_GTPASE\_ACTIVITY |  | 79 | -0.27 | -1.15 | 0.222 | 0.509 | 1.000 | 3072 | tags=33%, list=24%, signal=43% |
| 1291 | GOBP\_CELLULAR\_RESPONSE\_TO\_ACID\_CHEMICAL |  | 67 | -0.28 | -1.15 | 0.243 | 0.509 | 1.000 | 855 | tags=15%, list=7%, signal=16% |
| 1292 | GOBP\_ERK1\_AND\_ERK2\_CASCADE |  | 274 | -0.23 | -1.15 | 0.167 | 0.509 | 1.000 | 2043 | tags=19%, list=16%, signal=22% |
| 1293 | GOBP\_CELLULAR\_RESPONSE\_TO\_OSMOTIC\_STRESS |  | 35 | -0.32 | -1.15 | 0.241 | 0.510 | 1.000 | 2494 | tags=29%, list=19%, signal=35% |
| 1294 | GOBP\_POSITIVE\_REGULATION\_OF\_PEPTIDYL\_SERINE\_PHOSPHORYLATION |  | 87 | -0.27 | -1.15 | 0.221 | 0.510 | 1.000 | 3058 | tags=29%, list=24%, signal=37% |
| 1295 | GOBP\_POSITIVE\_REGULATION\_OF\_BIOMINERALIZATION |  | 43 | -0.31 | -1.15 | 0.259 | 0.511 | 1.000 | 1983 | tags=23%, list=15%, signal=27% |
| 1296 | GOBP\_POSITIVE\_REGULATION\_OF\_OSSIFICATION |  | 45 | -0.30 | -1.14 | 0.232 | 0.512 | 1.000 | 1983 | tags=22%, list=15%, signal=26% |
| 1297 | GOBP\_HIPPOCAMPUS\_DEVELOPMENT |  | 67 | -0.28 | -1.14 | 0.223 | 0.512 | 1.000 | 1917 | tags=22%, list=15%, signal=26% |
| 1298 | GOBP\_TRACHEA\_DEVELOPMENT |  | 17 | -0.39 | -1.14 | 0.305 | 0.512 | 1.000 | 2969 | tags=35%, list=23%, signal=46% |
| 1299 | GOBP\_ACTIN\_FILAMENT\_ORGANIZATION |  | 356 | -0.22 | -1.14 | 0.141 | 0.513 | 1.000 | 3160 | tags=26%, list=24%, signal=34% |
| 1300 | GOBP\_REGULATION\_OF\_HORMONE\_METABOLIC\_PROCESS |  | 33 | -0.33 | -1.14 | 0.240 | 0.514 | 1.000 | 1359 | tags=24%, list=11%, signal=27% |
| 1301 | GOBP\_ALDEHYDE\_BIOSYNTHETIC\_PROCESS |  | 16 | -0.40 | -1.14 | 0.289 | 0.515 | 1.000 | 2951 | tags=44%, list=23%, signal=57% |
| 1302 | GOBP\_NEGATIVE\_REGULATION\_OF\_LEUKOCYTE\_CELL\_CELL\_ADHESION |  | 111 | -0.26 | -1.14 | 0.201 | 0.515 | 1.000 | 2461 | tags=27%, list=19%, signal=33% |
| 1303 | GOBP\_POTASSIUM\_ION\_TRANSPORT |  | 176 | -0.24 | -1.14 | 0.184 | 0.516 | 1.000 | 3035 | tags=30%, list=24%, signal=38% |
| 1304 | GOBP\_RESPONSE\_TO\_CHEMOKINE |  | 90 | -0.27 | -1.14 | 0.213 | 0.516 | 1.000 | 4453 | tags=43%, list=35%, signal=66% |
| 1305 | GOBP\_REGULATION\_OF\_MUSCLE\_ORGAN\_DEVELOPMENT |  | 38 | -0.32 | -1.14 | 0.268 | 0.518 | 1.000 | 3577 | tags=34%, list=28%, signal=47% |
| 1306 | GOBP\_POSITIVE\_REGULATION\_OF\_LIPID\_STORAGE |  | 20 | -0.38 | -1.14 | 0.270 | 0.518 | 1.000 | 4917 | tags=55%, list=38%, signal=89% |
| 1307 | GOBP\_POSITIVE\_REGULATION\_OF\_MACROPHAGE\_ACTIVATION |  | 22 | -0.37 | -1.14 | 0.287 | 0.518 | 1.000 | 2000 | tags=36%, list=16%, signal=43% |
| 1308 | GOBP\_MYELIN\_MAINTENANCE |  | 17 | -0.38 | -1.14 | 0.301 | 0.518 | 1.000 | 2103 | tags=24%, list=16%, signal=28% |
| 1309 | GOBP\_REGULATION\_OF\_CHEMOTAXIS |  | 197 | -0.24 | -1.14 | 0.163 | 0.518 | 1.000 | 3133 | tags=29%, list=24%, signal=38% |
| 1310 | GOBP\_VESICLE\_CARGO\_LOADING |  | 22 | -0.37 | -1.14 | 0.276 | 0.518 | 1.000 | 3997 | tags=45%, list=31%, signal=66% |
| 1311 | GOBP\_SERINE\_FAMILY\_AMINO\_ACID\_BIOSYNTHETIC\_PROCESS |  | 16 | -0.39 | -1.14 | 0.288 | 0.518 | 1.000 | 3657 | tags=44%, list=28%, signal=61% |
| 1312 | GOBP\_NEGATIVE\_REGULATION\_OF\_PROTEIN\_MODIFICATION\_PROCESS |  | 415 | -0.22 | -1.14 | 0.127 | 0.518 | 1.000 | 4076 | tags=37%, list=32%, signal=52% |
| 1313 | GOBP\_EMBRYONIC\_CAMERA\_TYPE\_EYE\_MORPHOGENESIS |  | 24 | -0.36 | -1.14 | 0.282 | 0.518 | 1.000 | 2511 | tags=25%, list=19%, signal=31% |
| 1314 | GOBP\_REGULATION\_OF\_INNATE\_IMMUNE\_RESPONSE |  | 190 | -0.24 | -1.14 | 0.185 | 0.519 | 1.000 | 3707 | tags=33%, list=29%, signal=46% |
| 1315 | GOBP\_CORONARY\_VASCULATURE\_MORPHOGENESIS |  | 16 | -0.40 | -1.14 | 0.289 | 0.518 | 1.000 | 347 | tags=13%, list=3%, signal=13% |
| 1316 | GOBP\_REGULATION\_OF\_PRESYNAPTIC\_CYTOSOLIC\_CALCIUM\_ION\_CONCENTRATION |  | 15 | -0.40 | -1.14 | 0.287 | 0.518 | 1.000 | 4252 | tags=60%, list=33%, signal=89% |
| 1317 | GOBP\_GERM\_CELL\_DEVELOPMENT |  | 192 | -0.24 | -1.14 | 0.196 | 0.521 | 1.000 | 3030 | tags=29%, list=23%, signal=37% |
| 1318 | GOBP\_REGULATION\_OF\_FEEDING\_BEHAVIOR |  | 20 | -0.38 | -1.14 | 0.307 | 0.521 | 1.000 | 2391 | tags=30%, list=19%, signal=37% |
| 1319 | GOBP\_EMBRYONIC\_CAMERA\_TYPE\_EYE\_DEVELOPMENT |  | 33 | -0.33 | -1.14 | 0.268 | 0.523 | 1.000 | 5275 | tags=55%, list=41%, signal=92% |
| 1320 | GOBP\_REGULATION\_OF\_ANATOMICAL\_STRUCTURE\_SIZE |  | 414 | -0.22 | -1.14 | 0.128 | 0.523 | 1.000 | 4069 | tags=36%, list=32%, signal=51% |
| 1321 | GOBP\_RESPONSE\_TO\_CATECHOLAMINE |  | 85 | -0.27 | -1.14 | 0.236 | 0.523 | 1.000 | 3003 | tags=29%, list=23%, signal=38% |
| 1322 | GOBP\_NEURON\_DEATH\_IN\_RESPONSE\_TO\_OXIDATIVE\_STRESS |  | 31 | -0.33 | -1.14 | 0.262 | 0.523 | 1.000 | 1378 | tags=26%, list=11%, signal=29% |
| 1323 | GOBP\_PROTEIN\_MODIFICATION\_BY\_SMALL\_PROTEIN\_REMOVAL |  | 118 | -0.26 | -1.14 | 0.218 | 0.524 | 1.000 | 1996 | tags=23%, list=15%, signal=27% |
| 1324 | GOBP\_POSITIVE\_REGULATION\_OF\_NITRIC\_OXIDE\_SYNTHASE\_BIOSYNTHETIC\_PROCESS |  | 15 | -0.40 | -1.14 | 0.290 | 0.525 | 1.000 | 5199 | tags=60%, list=40%, signal=100% |
| 1325 | GOBP\_REGULATION\_OF\_LIPOPOLYSACCHARIDE\_MEDIATED\_SIGNALING\_PATHWAY |  | 21 | -0.36 | -1.14 | 0.287 | 0.525 | 1.000 | 978 | tags=19%, list=8%, signal=21% |
| 1326 | GOBP\_NEGATIVE\_REGULATION\_OF\_PLATELET\_ACTIVATION |  | 17 | -0.38 | -1.14 | 0.299 | 0.525 | 1.000 | 3219 | tags=35%, list=25%, signal=47% |
| 1327 | GOBP\_CAMP\_METABOLIC\_PROCESS |  | 19 | -0.38 | -1.14 | 0.303 | 0.525 | 1.000 | 3187 | tags=42%, list=25%, signal=56% |
| 1328 | GOBP\_ENDOTHELIAL\_CELL\_CHEMOTAXIS |  | 26 | -0.34 | -1.13 | 0.285 | 0.526 | 1.000 | 2944 | tags=38%, list=23%, signal=50% |
| 1329 | GOBP\_POSITIVE\_REGULATION\_OF\_ANION\_TRANSPORT |  | 46 | -0.30 | -1.13 | 0.272 | 0.526 | 1.000 | 1982 | tags=24%, list=15%, signal=28% |
| 1330 | GOBP\_GLUCOCORTICOID\_BIOSYNTHETIC\_PROCESS |  | 15 | -0.40 | -1.13 | 0.268 | 0.526 | 1.000 | 4646 | tags=73%, list=36%, signal=114% |
| 1331 | GOBP\_NEGATIVE\_REGULATION\_OF\_LIPID\_BIOSYNTHETIC\_PROCESS |  | 46 | -0.30 | -1.13 | 0.247 | 0.526 | 1.000 | 2063 | tags=24%, list=16%, signal=28% |
| 1332 | GOBP\_REGULATION\_OF\_GLYCOGEN\_METABOLIC\_PROCESS |  | 29 | -0.34 | -1.13 | 0.281 | 0.526 | 1.000 | 2072 | tags=31%, list=16%, signal=37% |
| 1333 | GOBP\_RESPONSE\_TO\_NERVE\_GROWTH\_FACTOR |  | 43 | -0.31 | -1.13 | 0.270 | 0.527 | 1.000 | 2531 | tags=30%, list=20%, signal=37% |
| 1334 | GOBP\_CELL\_CELL\_ADHESION\_VIA\_PLASMA\_MEMBRANE\_ADHESION\_MOLECULES |  | 186 | -0.24 | -1.13 | 0.204 | 0.527 | 1.000 | 3194 | tags=27%, list=25%, signal=36% |
| 1335 | GOBP\_PORE\_COMPLEX\_ASSEMBLY |  | 16 | -0.40 | -1.13 | 0.308 | 0.527 | 1.000 | 2027 | tags=25%, list=16%, signal=30% |
| 1336 | GOBP\_INTESTINAL\_ABSORPTION |  | 34 | -0.32 | -1.13 | 0.278 | 0.527 | 1.000 | 4917 | tags=56%, list=38%, signal=90% |
| 1337 | GOBP\_T\_CELL\_CHEMOTAXIS |  | 23 | -0.35 | -1.13 | 0.302 | 0.527 | 1.000 | 2338 | tags=35%, list=18%, signal=42% |
| 1338 | GOBP\_POSITIVE\_REGULATION\_OF\_TRANSFERASE\_ACTIVITY |  | 485 | -0.22 | -1.13 | 0.125 | 0.526 | 1.000 | 2848 | tags=25%, list=22%, signal=31% |
| 1339 | GOBP\_BICARBONATE\_TRANSPORT |  | 22 | -0.36 | -1.13 | 0.275 | 0.527 | 1.000 | 3641 | tags=41%, list=28%, signal=57% |
| 1340 | GOBP\_REGULATION\_OF\_DEFENSE\_RESPONSE\_TO\_VIRUS\_BY\_HOST |  | 29 | -0.34 | -1.13 | 0.278 | 0.529 | 1.000 | 797 | tags=17%, list=6%, signal=18% |
| 1341 | GOBP\_NEGATIVE\_REGULATION\_OF\_TRANSPORT |  | 340 | -0.22 | -1.13 | 0.147 | 0.529 | 1.000 | 4005 | tags=34%, list=31%, signal=48% |
| 1342 | GOBP\_NEURON\_PROJECTION\_EXTENSION\_INVOLVED\_IN\_NEURON\_PROJECTION\_GUIDANCE |  | 33 | -0.33 | -1.13 | 0.299 | 0.530 | 1.000 | 3001 | tags=36%, list=23%, signal=47% |
| 1343 | GOBP\_REGULATION\_OF\_CELL\_JUNCTION\_ASSEMBLY |  | 161 | -0.24 | -1.13 | 0.217 | 0.530 | 1.000 | 3821 | tags=34%, list=30%, signal=48% |
| 1344 | GOBP\_MITOTIC\_DNA\_INTEGRITY\_CHECKPOINT\_SIGNALING |  | 70 | -0.28 | -1.13 | 0.240 | 0.530 | 1.000 | 1444 | tags=17%, list=11%, signal=19% |
| 1345 | GOBP\_DOUBLE\_STRAND\_BREAK\_REPAIR |  | 205 | -0.24 | -1.13 | 0.181 | 0.530 | 1.000 | 1444 | tags=17%, list=11%, signal=18% |
| 1346 | GOBP\_REGULATION\_OF\_MICROGLIAL\_CELL\_ACTIVATION |  | 15 | -0.40 | -1.13 | 0.310 | 0.532 | 1.000 | 1437 | tags=27%, list=11%, signal=30% |
| 1347 | GOBP\_NEGATIVE\_REGULATION\_OF\_OXIDATIVE\_STRESS\_INDUCED\_NEURON\_DEATH |  | 19 | -0.37 | -1.13 | 0.292 | 0.532 | 1.000 | 1327 | tags=32%, list=10%, signal=35% |
| 1348 | GOBP\_POSITIVE\_REGULATION\_OF\_FATTY\_ACID\_BIOSYNTHETIC\_PROCESS |  | 19 | -0.37 | -1.13 | 0.297 | 0.533 | 1.000 | 2078 | tags=26%, list=16%, signal=31% |
| 1349 | GOBP\_NEGATIVE\_REGULATION\_OF\_SECRETION |  | 138 | -0.25 | -1.13 | 0.215 | 0.533 | 1.000 | 3708 | tags=34%, list=29%, signal=47% |
| 1350 | GOBP\_POSITIVE\_REGULATION\_OF\_RESPONSE\_TO\_ENDOPLASMIC\_RETICULUM\_STRESS |  | 29 | -0.33 | -1.13 | 0.285 | 0.535 | 1.000 | 4450 | tags=55%, list=34%, signal=84% |
| 1351 | GOBP\_NEURAL\_PRECURSOR\_CELL\_PROLIFERATION |  | 117 | -0.25 | -1.13 | 0.236 | 0.536 | 1.000 | 2196 | tags=20%, list=17%, signal=23% |
| 1352 | GOBP\_RESPONSE\_TO\_NICOTINE |  | 45 | -0.30 | -1.13 | 0.285 | 0.536 | 1.000 | 2725 | tags=29%, list=21%, signal=36% |
| 1353 | GOBP\_NEGATIVE\_REGULATION\_OF\_TRANSLATIONAL\_INITIATION |  | 16 | -0.39 | -1.13 | 0.302 | 0.538 | 1.000 | 2877 | tags=44%, list=22%, signal=56% |
| 1354 | GOBP\_POSITIVE\_REGULATION\_OF\_PROTEIN\_KINASE\_ACTIVITY |  | 340 | -0.22 | -1.13 | 0.178 | 0.537 | 1.000 | 2848 | tags=25%, list=22%, signal=32% |
| 1355 | GOBP\_REGULATION\_OF\_INTRACELLULAR\_STEROID\_HORMONE\_RECEPTOR\_SIGNALING\_PATHWAY |  | 65 | -0.28 | -1.13 | 0.263 | 0.537 | 1.000 | 1756 | tags=22%, list=14%, signal=25% |
| 1356 | GOBP\_NOTCH\_SIGNALING\_PATHWAY |  | 139 | -0.24 | -1.13 | 0.235 | 0.538 | 1.000 | 2514 | tags=24%, list=19%, signal=29% |
| 1357 | GOBP\_NEGATIVE\_REGULATION\_OF\_T\_CELL\_PROLIFERATION |  | 57 | -0.28 | -1.13 | 0.262 | 0.539 | 1.000 | 4200 | tags=44%, list=33%, signal=65% |
| 1358 | GOBP\_NEGATIVE\_REGULATION\_OF\_ADENYLATE\_CYCLASE\_ACTIVITY |  | 19 | -0.38 | -1.13 | 0.318 | 0.539 | 1.000 | 1551 | tags=26%, list=12%, signal=30% |
| 1359 | GOBP\_POSITIVE\_REGULATION\_OF\_AMINO\_ACID\_TRANSPORT |  | 20 | -0.37 | -1.13 | 0.305 | 0.539 | 1.000 | 1261 | tags=25%, list=10%, signal=28% |
| 1360 | GOBP\_ENDOTHELIAL\_CELL\_PROLIFERATION |  | 139 | -0.25 | -1.12 | 0.222 | 0.540 | 1.000 | 2180 | tags=22%, list=17%, signal=27% |
| 1361 | GOBP\_POSITIVE\_REGULATION\_OF\_TYPE\_I\_INTERFERON\_PRODUCTION |  | 50 | -0.30 | -1.12 | 0.271 | 0.541 | 1.000 | 3673 | tags=44%, list=28%, signal=61% |
| 1362 | GOBP\_MYELOID\_CELL\_DEVELOPMENT |  | 63 | -0.28 | -1.12 | 0.257 | 0.540 | 1.000 | 2182 | tags=25%, list=17%, signal=30% |
| 1363 | GOBP\_MYELOID\_LEUKOCYTE\_MIGRATION |  | 200 | -0.24 | -1.12 | 0.222 | 0.541 | 1.000 | 3551 | tags=35%, list=28%, signal=47% |
| 1364 | GOBP\_REGULATION\_OF\_SMOOTH\_MUSCLE\_CONTRACTION |  | 53 | -0.29 | -1.12 | 0.278 | 0.541 | 1.000 | 2126 | tags=23%, list=16%, signal=27% |
| 1365 | GOBP\_RESPONSE\_TO\_TESTOSTERONE |  | 39 | -0.31 | -1.12 | 0.292 | 0.541 | 1.000 | 2815 | tags=36%, list=22%, signal=46% |
| 1366 | GOBP\_NEGATIVE\_REGULATION\_OF\_DNA\_BINDING |  | 45 | -0.30 | -1.12 | 0.277 | 0.542 | 1.000 | 2979 | tags=31%, list=23%, signal=40% |
| 1367 | GOBP\_DNA\_TEMPLATED\_TRANSCRIPTION\_TERMINATION |  | 15 | -0.40 | -1.12 | 0.288 | 0.542 | 1.000 | 2741 | tags=47%, list=21%, signal=59% |
| 1368 | GOBP\_CELL\_SURFACE\_RECEPTOR\_SIGNALING\_PATHWAY\_INVOLVED\_IN\_HEART\_DEVELOPMENT |  | 24 | -0.35 | -1.12 | 0.285 | 0.542 | 1.000 | 2182 | tags=33%, list=17%, signal=40% |
| 1369 | GOBP\_DIACYLGLYCEROL\_METABOLIC\_PROCESS |  | 19 | -0.37 | -1.12 | 0.288 | 0.543 | 1.000 | 2372 | tags=32%, list=18%, signal=39% |
| 1370 | GOBP\_NEGATIVE\_REGULATION\_OF\_RESPONSE\_TO\_BIOTIC\_STIMULUS |  | 95 | -0.26 | -1.12 | 0.262 | 0.544 | 1.000 | 2340 | tags=21%, list=18%, signal=26% |
| 1371 | GOBP\_STEROID\_HORMONE\_BIOSYNTHETIC\_PROCESS |  | 40 | -0.31 | -1.12 | 0.272 | 0.545 | 1.000 | 1983 | tags=28%, list=15%, signal=32% |
| 1372 | GOBP\_CHEMOKINE\_PRODUCTION |  | 83 | -0.26 | -1.12 | 0.242 | 0.545 | 1.000 | 1629 | tags=20%, list=13%, signal=23% |
| 1373 | GOBP\_RESPONSE\_TO\_DSRNA |  | 44 | -0.30 | -1.12 | 0.270 | 0.545 | 1.000 | 2275 | tags=25%, list=18%, signal=30% |
| 1374 | GOBP\_EYE\_MORPHOGENESIS |  | 132 | -0.25 | -1.12 | 0.216 | 0.547 | 1.000 | 3763 | tags=30%, list=29%, signal=42% |
| 1375 | GOBP\_ANION\_TRANSMEMBRANE\_TRANSPORT |  | 189 | -0.24 | -1.12 | 0.216 | 0.547 | 1.000 | 4443 | tags=42%, list=34%, signal=63% |
| 1376 | GOBP\_GLIAL\_CELL\_MIGRATION |  | 45 | -0.30 | -1.12 | 0.253 | 0.547 | 1.000 | 2554 | tags=36%, list=20%, signal=44% |
| 1377 | GOBP\_LUNG\_MORPHOGENESIS |  | 41 | -0.30 | -1.12 | 0.272 | 0.550 | 1.000 | 1174 | tags=15%, list=9%, signal=16% |
| 1378 | GOBP\_POSITIVE\_REGULATION\_OF\_RESPONSE\_TO\_EXTERNAL\_STIMULUS |  | 362 | -0.22 | -1.12 | 0.172 | 0.550 | 1.000 | 3700 | tags=33%, list=29%, signal=45% |
| 1379 | GOBP\_ARACHIDONIC\_ACID\_METABOLIC\_PROCESS |  | 44 | -0.30 | -1.12 | 0.298 | 0.550 | 1.000 | 3303 | tags=34%, list=26%, signal=46% |
| 1380 | GOBP\_NEGATIVE\_REGULATION\_OF\_INTRINSIC\_APOPTOTIC\_SIGNALING\_PATHWAY |  | 85 | -0.26 | -1.12 | 0.266 | 0.550 | 1.000 | 1463 | tags=18%, list=11%, signal=20% |
| 1381 | GOBP\_LAMELLIPODIUM\_ORGANIZATION |  | 75 | -0.28 | -1.12 | 0.258 | 0.550 | 1.000 | 1859 | tags=24%, list=14%, signal=28% |
| 1382 | GOBP\_RESPONSE\_TO\_HEAT |  | 95 | -0.26 | -1.12 | 0.252 | 0.550 | 1.000 | 3976 | tags=42%, list=31%, signal=60% |
| 1383 | GOBP\_POSITIVE\_REGULATION\_OF\_INTERFERON\_BETA\_PRODUCTION |  | 35 | -0.32 | -1.12 | 0.302 | 0.550 | 1.000 | 2275 | tags=31%, list=18%, signal=38% |
| 1384 | GOBP\_NEGATIVE\_REGULATION\_OF\_RESPONSE\_TO\_CYTOKINE\_STIMULUS |  | 62 | -0.28 | -1.12 | 0.279 | 0.551 | 1.000 | 842 | tags=15%, list=7%, signal=15% |
| 1385 | GOBP\_HISTONE\_H3\_K14\_ACETYLATION |  | 18 | -0.38 | -1.12 | 0.317 | 0.552 | 1.000 | 1683 | tags=33%, list=13%, signal=38% |
| 1386 | GOBP\_NEURAL\_CREST\_CELL\_DIFFERENTIATION |  | 83 | -0.27 | -1.12 | 0.254 | 0.552 | 1.000 | 1960 | tags=24%, list=15%, signal=28% |
| 1387 | GOBP\_CELLULAR\_RESPONSE\_TO\_BIOTIC\_STIMULUS |  | 209 | -0.23 | -1.12 | 0.223 | 0.552 | 1.000 | 3890 | tags=34%, list=30%, signal=49% |
| 1388 | GOBP\_REGULATION\_OF\_CELL\_CYCLE\_CHECKPOINT |  | 29 | -0.33 | -1.12 | 0.285 | 0.553 | 1.000 | 475 | tags=17%, list=4%, signal=18% |
| 1389 | GOBP\_POSITIVE\_REGULATION\_OF\_INTERLEUKIN\_6\_PRODUCTION |  | 80 | -0.27 | -1.12 | 0.276 | 0.553 | 1.000 | 3700 | tags=38%, list=29%, signal=52% |
| 1390 | GOBP\_NEURON\_MATURATION |  | 38 | -0.31 | -1.12 | 0.282 | 0.552 | 1.000 | 1821 | tags=24%, list=14%, signal=27% |
| 1391 | GOBP\_POSITIVE\_REGULATION\_OF\_RESPONSE\_TO\_BIOTIC\_STIMULUS |  | 142 | -0.24 | -1.12 | 0.231 | 0.552 | 1.000 | 3673 | tags=35%, list=28%, signal=49% |
| 1392 | GOBP\_NEGATIVE\_REGULATION\_OF\_AXON\_EXTENSION\_INVOLVED\_IN\_AXON\_GUIDANCE |  | 24 | -0.34 | -1.12 | 0.291 | 0.552 | 1.000 | 2394 | tags=33%, list=19%, signal=41% |
| 1393 | GOBP\_VIRAL\_GENOME\_REPLICATION |  | 120 | -0.25 | -1.12 | 0.253 | 0.552 | 1.000 | 3088 | tags=31%, list=24%, signal=40% |
| 1394 | GOBP\_ACTIN\_FILAMENT\_BUNDLE\_ORGANIZATION |  | 137 | -0.24 | -1.11 | 0.241 | 0.553 | 1.000 | 1803 | tags=18%, list=14%, signal=21% |
| 1395 | GOBP\_INSULIN\_LIKE\_GROWTH\_FACTOR\_RECEPTOR\_SIGNALING\_PATHWAY |  | 31 | -0.33 | -1.11 | 0.313 | 0.554 | 1.000 | 2196 | tags=29%, list=17%, signal=35% |
| 1396 | GOBP\_ACTIVATION\_OF\_PROTEIN\_KINASE\_ACTIVITY |  | 122 | -0.25 | -1.11 | 0.256 | 0.557 | 1.000 | 2184 | tags=23%, list=17%, signal=27% |
| 1397 | GOBP\_REGULATION\_OF\_BINDING |  | 318 | -0.22 | -1.11 | 0.172 | 0.556 | 1.000 | 2323 | tags=22%, list=18%, signal=26% |
| 1398 | GOBP\_NECROTIC\_CELL\_DEATH |  | 45 | -0.30 | -1.11 | 0.292 | 0.556 | 1.000 | 2903 | tags=29%, list=23%, signal=37% |
| 1399 | GOBP\_REGULATION\_OF\_PROTEIN\_SUMOYLATION |  | 22 | -0.35 | -1.11 | 0.319 | 0.556 | 1.000 | 2542 | tags=27%, list=20%, signal=34% |
| 1400 | GOBP\_HEART\_FORMATION |  | 25 | -0.34 | -1.11 | 0.293 | 0.556 | 1.000 | 2059 | tags=28%, list=16%, signal=33% |
| 1401 | GOBP\_MONOUBIQUITINATED\_PROTEIN\_DEUBIQUITINATION |  | 29 | -0.33 | -1.11 | 0.278 | 0.556 | 1.000 | 639 | tags=17%, list=5%, signal=18% |
| 1402 | GOBP\_POSITIVE\_REGULATION\_OF\_INTERFERON\_ALPHA\_PRODUCTION |  | 19 | -0.36 | -1.11 | 0.325 | 0.556 | 1.000 | 2275 | tags=37%, list=18%, signal=45% |
| 1403 | GOBP\_GRANULOCYTE\_MIGRATION |  | 136 | -0.24 | -1.11 | 0.231 | 0.556 | 1.000 | 3700 | tags=37%, list=29%, signal=51% |
| 1404 | GOBP\_FORELIMB\_MORPHOGENESIS |  | 31 | -0.32 | -1.11 | 0.297 | 0.556 | 1.000 | 1206 | tags=19%, list=9%, signal=21% |
| 1405 | GOBP\_INTEGRIN\_MEDIATED\_SIGNALING\_PATHWAY |  | 98 | -0.26 | -1.11 | 0.245 | 0.556 | 1.000 | 4165 | tags=42%, list=32%, signal=61% |
| 1406 | GOBP\_PROTEIN\_TRANSMEMBRANE\_TRANSPORT |  | 51 | -0.29 | -1.11 | 0.281 | 0.556 | 1.000 | 3072 | tags=37%, list=24%, signal=49% |
| 1407 | GOBP\_POSITIVE\_REGULATION\_OF\_SODIUM\_ION\_TRANSPORT |  | 26 | -0.34 | -1.11 | 0.308 | 0.557 | 1.000 | 3027 | tags=35%, list=23%, signal=45% |
| 1408 | GOBP\_POSITIVE\_REGULATION\_OF\_APOPTOTIC\_SIGNALING\_PATHWAY |  | 113 | -0.25 | -1.11 | 0.263 | 0.557 | 1.000 | 2553 | tags=28%, list=20%, signal=35% |
| 1409 | GOBP\_POSITIVE\_REGULATION\_OF\_STEROID\_BIOSYNTHETIC\_PROCESS |  | 20 | -0.36 | -1.11 | 0.326 | 0.556 | 1.000 | 2881 | tags=30%, list=22%, signal=39% |
| 1410 | GOBP\_RESPONSE\_TO\_ACIDIC\_PH |  | 25 | -0.34 | -1.11 | 0.335 | 0.560 | 1.000 | 3957 | tags=36%, list=31%, signal=52% |
| 1411 | GOBP\_NEGATIVE\_REGULATION\_OF\_STRIATED\_MUSCLE\_CELL\_DIFFERENTIATION |  | 27 | -0.33 | -1.11 | 0.315 | 0.560 | 1.000 | 1983 | tags=30%, list=15%, signal=35% |
| 1412 | GOBP\_DETECTION\_OF\_EXTERNAL\_BIOTIC\_STIMULUS |  | 19 | -0.37 | -1.11 | 0.308 | 0.560 | 1.000 | 1961 | tags=32%, list=15%, signal=37% |
| 1413 | GOBP\_POSITIVE\_REGULATION\_OF\_CHEMOTAXIS |  | 125 | -0.24 | -1.11 | 0.247 | 0.560 | 1.000 | 2338 | tags=24%, list=18%, signal=29% |
| 1414 | GOBP\_NITRIC\_OXIDE\_SYNTHASE\_BIOSYNTHETIC\_PROCESS |  | 17 | -0.38 | -1.11 | 0.317 | 0.561 | 1.000 | 1994 | tags=29%, list=15%, signal=35% |
| 1415 | GOBP\_VITAMIN\_TRANSPORT |  | 38 | -0.31 | -1.11 | 0.299 | 0.560 | 1.000 | 4250 | tags=47%, list=33%, signal=70% |
| 1416 | GOBP\_TELOMERE\_MAINTENANCE |  | 112 | -0.25 | -1.11 | 0.260 | 0.560 | 1.000 | 1019 | tags=13%, list=8%, signal=14% |
| 1417 | GOBP\_REGULATION\_OF\_SENSORY\_PERCEPTION |  | 29 | -0.33 | -1.11 | 0.301 | 0.560 | 1.000 | 1626 | tags=21%, list=13%, signal=24% |
| 1418 | GOBP\_RELAXATION\_OF\_CARDIAC\_MUSCLE |  | 15 | -0.39 | -1.11 | 0.321 | 0.560 | 1.000 | 1197 | tags=27%, list=9%, signal=29% |
| 1419 | GOBP\_KERATINOCYTE\_MIGRATION |  | 17 | -0.38 | -1.11 | 0.324 | 0.560 | 1.000 | 2768 | tags=35%, list=21%, signal=45% |
| 1420 | GOBP\_CARDIAC\_ATRIUM\_MORPHOGENESIS |  | 24 | -0.35 | -1.11 | 0.313 | 0.560 | 1.000 | 3027 | tags=33%, list=23%, signal=43% |
| 1421 | GOBP\_MODIFICATION\_OF\_SYNAPTIC\_STRUCTURE |  | 17 | -0.38 | -1.11 | 0.327 | 0.560 | 1.000 | 3010 | tags=41%, list=23%, signal=54% |
| 1422 | GOBP\_POSITIVE\_REGULATION\_OF\_DNA\_REPAIR |  | 71 | -0.27 | -1.11 | 0.273 | 0.561 | 1.000 | 2469 | tags=25%, list=19%, signal=31% |
| 1423 | GOBP\_POSITIVE\_REGULATION\_OF\_NEURAL\_PRECURSOR\_CELL\_PROLIFERATION |  | 45 | -0.30 | -1.11 | 0.310 | 0.561 | 1.000 | 1217 | tags=16%, list=9%, signal=17% |
| 1424 | GOBP\_REGULATION\_OF\_LIPID\_KINASE\_ACTIVITY |  | 57 | -0.28 | -1.11 | 0.288 | 0.564 | 1.000 | 2437 | tags=26%, list=19%, signal=32% |
| 1425 | GOBP\_HORMONE\_TRANSPORT |  | 253 | -0.22 | -1.11 | 0.218 | 0.564 | 1.000 | 3867 | tags=34%, list=30%, signal=48% |
| 1426 | GOBP\_POSITIVE\_REGULATION\_OF\_TELOMERASE\_ACTIVITY |  | 31 | -0.32 | -1.11 | 0.296 | 0.564 | 1.000 | 693 | tags=16%, list=5%, signal=17% |
| 1427 | GOBP\_REGULATION\_OF\_RESPONSE\_TO\_TUMOR\_CELL |  | 18 | -0.37 | -1.11 | 0.315 | 0.564 | 1.000 | 2916 | tags=39%, list=23%, signal=50% |
| 1428 | GOBP\_REGULATION\_OF\_LIPID\_LOCALIZATION |  | 139 | -0.24 | -1.11 | 0.249 | 0.564 | 1.000 | 4065 | tags=40%, list=32%, signal=57% |
| 1429 | GOBP\_GLAND\_DEVELOPMENT |  | 387 | -0.21 | -1.11 | 0.205 | 0.564 | 1.000 | 1554 | tags=14%, list=12%, signal=16% |
| 1430 | GOBP\_HEMOGLOBIN\_METABOLIC\_PROCESS |  | 16 | -0.39 | -1.11 | 0.342 | 0.564 | 1.000 | 1746 | tags=31%, list=14%, signal=36% |
| 1431 | GOBP\_POSITIVE\_REGULATION\_OF\_PROTEIN\_CONTAINING\_COMPLEX\_ASSEMBLY |  | 158 | -0.24 | -1.11 | 0.233 | 0.564 | 1.000 | 3693 | tags=34%, list=29%, signal=46% |
| 1432 | GOBP\_OSTEOCLAST\_DIFFERENTIATION |  | 83 | -0.26 | -1.11 | 0.265 | 0.564 | 1.000 | 1437 | tags=18%, list=11%, signal=20% |
| 1433 | GOBP\_RENAL\_WATER\_HOMEOSTASIS |  | 20 | -0.35 | -1.10 | 0.306 | 0.568 | 1.000 | 3303 | tags=50%, list=26%, signal=67% |
| 1434 | GOBP\_CARTILAGE\_DEVELOPMENT |  | 168 | -0.24 | -1.10 | 0.248 | 0.567 | 1.000 | 4356 | tags=40%, list=34%, signal=59% |
| 1435 | GOBP\_NEGATIVE\_REGULATION\_OF\_MITOTIC\_CELL\_CYCLE |  | 182 | -0.23 | -1.10 | 0.245 | 0.568 | 1.000 | 3420 | tags=28%, list=27%, signal=38% |
| 1436 | GOBP\_SYNAPTIC\_VESICLE\_TRANSPORT |  | 29 | -0.33 | -1.10 | 0.313 | 0.569 | 1.000 | 1325 | tags=21%, list=10%, signal=23% |
| 1437 | GOBP\_REGULATION\_OF\_TRANSCRIPTION\_ELONGATION\_FROM\_RNA\_POLYMERASE\_II\_PROMOTER |  | 36 | -0.31 | -1.10 | 0.321 | 0.570 | 1.000 | 4380 | tags=53%, list=34%, signal=80% |
| 1438 | GOBP\_MUSCLE\_ORGAN\_DEVELOPMENT |  | 267 | -0.22 | -1.10 | 0.229 | 0.575 | 1.000 | 3723 | tags=30%, list=29%, signal=42% |
| 1439 | GOBP\_SECOND\_MESSENGER\_MEDIATED\_SIGNALING |  | 263 | -0.22 | -1.10 | 0.232 | 0.575 | 1.000 | 2512 | tags=23%, list=19%, signal=28% |
| 1440 | GOBP\_REGULATION\_OF\_MITOTIC\_NUCLEAR\_DIVISION |  | 95 | -0.26 | -1.10 | 0.292 | 0.575 | 1.000 | 797 | tags=11%, list=6%, signal=11% |
| 1441 | GOBP\_INTESTINAL\_EPITHELIAL\_CELL\_DIFFERENTIATION |  | 21 | -0.35 | -1.10 | 0.329 | 0.575 | 1.000 | 1069 | tags=19%, list=8%, signal=21% |
| 1442 | GOBP\_DIGESTIVE\_SYSTEM\_PROCESS |  | 84 | -0.26 | -1.10 | 0.291 | 0.576 | 1.000 | 4527 | tags=42%, list=35%, signal=64% |
| 1443 | GOBP\_ORGANELLE\_TRANSPORT\_ALONG\_MICROTUBULE |  | 68 | -0.27 | -1.10 | 0.291 | 0.576 | 1.000 | 2554 | tags=28%, list=20%, signal=35% |
| 1444 | GOBP\_REGULATION\_OF\_BLOOD\_CIRCULATION |  | 222 | -0.23 | -1.10 | 0.245 | 0.576 | 1.000 | 3115 | tags=25%, list=24%, signal=33% |
| 1445 | GOBP\_PULMONARY\_VALVE\_MORPHOGENESIS |  | 15 | -0.40 | -1.10 | 0.332 | 0.577 | 1.000 | 2922 | tags=33%, list=23%, signal=43% |
| 1446 | GOBP\_POSITIVE\_REGULATION\_OF\_DOUBLE\_STRAND\_BREAK\_REPAIR\_VIA\_HOMOLOGOUS\_RECOMBINATION |  | 29 | -0.33 | -1.10 | 0.326 | 0.578 | 1.000 | 2469 | tags=28%, list=19%, signal=34% |
| 1447 | GOBP\_RECEPTOR\_METABOLIC\_PROCESS |  | 138 | -0.24 | -1.10 | 0.241 | 0.579 | 1.000 | 1931 | tags=20%, list=15%, signal=24% |
| 1448 | GOBP\_ENDOCRINE\_PANCREAS\_DEVELOPMENT |  | 40 | -0.30 | -1.10 | 0.312 | 0.579 | 1.000 | 2881 | tags=33%, list=22%, signal=42% |
| 1449 | GOBP\_DENDRITIC\_SPINE\_MAINTENANCE |  | 17 | -0.37 | -1.10 | 0.320 | 0.578 | 1.000 | 3118 | tags=47%, list=24%, signal=62% |
| 1450 | GOBP\_POSITIVE\_REGULATION\_OF\_WNT\_SIGNALING\_PATHWAY |  | 112 | -0.25 | -1.10 | 0.267 | 0.580 | 1.000 | 1639 | tags=18%, list=13%, signal=20% |
| 1451 | GOBP\_REGULATION\_OF\_NEURAL\_PRECURSOR\_CELL\_PROLIFERATION |  | 72 | -0.27 | -1.10 | 0.308 | 0.580 | 1.000 | 1907 | tags=19%, list=15%, signal=23% |
| 1452 | GOBP\_CARBOHYDRATE\_BIOSYNTHETIC\_PROCESS |  | 173 | -0.23 | -1.10 | 0.269 | 0.579 | 1.000 | 2072 | tags=21%, list=16%, signal=24% |
| 1453 | GOBP\_ANTIGEN\_PROCESSING\_AND\_PRESENTATION\_OF\_EXOGENOUS\_PEPTIDE\_ANTIGEN |  | 35 | -0.31 | -1.10 | 0.341 | 0.579 | 1.000 | 555 | tags=14%, list=4%, signal=15% |
| 1454 | GOBP\_TEMPERATURE\_HOMEOSTASIS |  | 165 | -0.24 | -1.10 | 0.247 | 0.579 | 1.000 | 2593 | tags=23%, list=20%, signal=28% |
| 1455 | GOBP\_NEGATIVE\_REGULATION\_OF\_CELL\_ACTIVATION |  | 178 | -0.23 | -1.10 | 0.244 | 0.579 | 1.000 | 2461 | tags=24%, list=19%, signal=29% |
| 1456 | GOBP\_REGULATION\_OF\_MUSCLE\_SYSTEM\_PROCESS |  | 197 | -0.23 | -1.10 | 0.261 | 0.579 | 1.000 | 3111 | tags=27%, list=24%, signal=35% |
| 1457 | GOBP\_LEUKOCYTE\_CELL\_CELL\_ADHESION |  | 321 | -0.22 | -1.10 | 0.221 | 0.579 | 1.000 | 3669 | tags=32%, list=28%, signal=43% |
| 1458 | GOBP\_DNA\_METHYLATION\_OR\_DEMETHYLATION |  | 69 | -0.27 | -1.10 | 0.313 | 0.582 | 1.000 | 2352 | tags=25%, list=18%, signal=30% |
| 1459 | GOBP\_INTERLEUKIN\_4\_PRODUCTION |  | 28 | -0.33 | -1.09 | 0.333 | 0.582 | 1.000 | 1243 | tags=18%, list=10%, signal=20% |
| 1460 | GOBP\_TRANSFORMING\_GROWTH\_FACTOR\_BETA\_RECEPTOR\_SIGNALING\_PATHWAY |  | 164 | -0.23 | -1.09 | 0.257 | 0.582 | 1.000 | 3589 | tags=33%, list=28%, signal=45% |
| 1461 | GOBP\_NEGATIVE\_REGULATION\_OF\_VIRAL\_LIFE\_CYCLE |  | 23 | -0.34 | -1.09 | 0.313 | 0.582 | 1.000 | 3727 | tags=48%, list=29%, signal=67% |
| 1462 | GOBP\_CORTICAL\_ACTIN\_CYTOSKELETON\_ORGANIZATION |  | 35 | -0.30 | -1.09 | 0.325 | 0.581 | 1.000 | 2254 | tags=23%, list=17%, signal=28% |
| 1463 | GOBP\_MATURE\_B\_CELL\_DIFFERENTIATION |  | 28 | -0.33 | -1.09 | 0.323 | 0.582 | 1.000 | 2235 | tags=32%, list=17%, signal=39% |
| 1464 | GOBP\_REGULATION\_OF\_CYTOSOLIC\_CALCIUM\_ION\_CONCENTRATION |  | 302 | -0.22 | -1.09 | 0.234 | 0.582 | 1.000 | 3081 | tags=26%, list=24%, signal=34% |
| 1465 | GOBP\_POSITIVE\_REGULATION\_OF\_DOUBLE\_STRAND\_BREAK\_REPAIR |  | 46 | -0.29 | -1.09 | 0.326 | 0.582 | 1.000 | 1345 | tags=17%, list=10%, signal=19% |
| 1466 | GOBP\_CEREBRAL\_CORTEX\_DEVELOPMENT |  | 93 | -0.25 | -1.09 | 0.299 | 0.582 | 1.000 | 2165 | tags=20%, list=17%, signal=24% |
| 1467 | GOBP\_POSITIVE\_REGULATION\_OF\_DEFENSE\_RESPONSE\_TO\_VIRUS\_BY\_HOST |  | 20 | -0.35 | -1.09 | 0.332 | 0.583 | 1.000 | 501 | tags=15%, list=4%, signal=16% |
| 1468 | GOBP\_NEUTROPHIL\_HOMEOSTASIS |  | 15 | -0.39 | -1.09 | 0.346 | 0.583 | 1.000 | 845 | tags=20%, list=7%, signal=21% |
| 1469 | GOBP\_NEGATIVE\_REGULATION\_OF\_LYMPHOCYTE\_APOPTOTIC\_PROCESS |  | 25 | -0.34 | -1.09 | 0.347 | 0.583 | 1.000 | 3669 | tags=44%, list=28%, signal=61% |
| 1470 | GOBP\_LIPID\_IMPORT\_INTO\_CELL |  | 16 | -0.38 | -1.09 | 0.345 | 0.583 | 1.000 | 2678 | tags=31%, list=21%, signal=39% |
| 1471 | GOBP\_CELLULAR\_RESPONSE\_TO\_INORGANIC\_SUBSTANCE |  | 180 | -0.23 | -1.09 | 0.235 | 0.583 | 1.000 | 1833 | tags=19%, list=14%, signal=22% |
| 1472 | GOBP\_RESPONSE\_TO\_ACID\_CHEMICAL |  | 107 | -0.25 | -1.09 | 0.275 | 0.583 | 1.000 | 3010 | tags=26%, list=23%, signal=34% |
| 1473 | GOBP\_CARDIAC\_RIGHT\_VENTRICLE\_MORPHOGENESIS |  | 19 | -0.35 | -1.09 | 0.318 | 0.582 | 1.000 | 3820 | tags=42%, list=30%, signal=60% |
| 1474 | GOBP\_NEGATIVE\_REGULATION\_OF\_EPITHELIAL\_CELL\_MIGRATION |  | 62 | -0.27 | -1.09 | 0.321 | 0.583 | 1.000 | 2916 | tags=31%, list=23%, signal=39% |
| 1475 | GOBP\_EAR\_DEVELOPMENT |  | 166 | -0.24 | -1.09 | 0.274 | 0.582 | 1.000 | 2834 | tags=27%, list=22%, signal=34% |
| 1476 | GOBP\_NEGATIVE\_REGULATION\_OF\_INTERFERON\_GAMMA\_PRODUCTION |  | 31 | -0.32 | -1.09 | 0.317 | 0.582 | 1.000 | 4421 | tags=42%, list=34%, signal=64% |
| 1477 | GOBP\_HORMONE\_BIOSYNTHETIC\_PROCESS |  | 63 | -0.28 | -1.09 | 0.299 | 0.582 | 1.000 | 2017 | tags=25%, list=16%, signal=30% |
| 1478 | GOBP\_NEGATIVE\_REGULATION\_OF\_CYTOKINE\_PRODUCTION |  | 237 | -0.22 | -1.09 | 0.244 | 0.582 | 1.000 | 2161 | tags=19%, list=17%, signal=23% |
| 1479 | GOBP\_PRESYNAPSE\_ORGANIZATION |  | 38 | -0.30 | -1.09 | 0.323 | 0.582 | 1.000 | 3150 | tags=34%, list=24%, signal=45% |
| 1480 | GOBP\_PHOTOTRANSDUCTION |  | 38 | -0.31 | -1.09 | 0.324 | 0.583 | 1.000 | 2600 | tags=32%, list=20%, signal=39% |
| 1481 | GOBP\_RHYTHMIC\_BEHAVIOR |  | 36 | -0.30 | -1.09 | 0.308 | 0.583 | 1.000 | 1066 | tags=17%, list=8%, signal=18% |
| 1482 | GOBP\_NEGATIVE\_REGULATION\_OF\_RECEPTOR\_SIGNALING\_PATHWAY\_VIA\_JAK\_STAT |  | 20 | -0.36 | -1.09 | 0.346 | 0.583 | 1.000 | 3100 | tags=40%, list=24%, signal=53% |
| 1483 | GOBP\_NEGATIVE\_REGULATION\_OF\_TUMOR\_NECROSIS\_FACTOR\_MEDIATED\_SIGNALING\_PATHWAY |  | 17 | -0.37 | -1.09 | 0.317 | 0.582 | 1.000 | 4421 | tags=59%, list=34%, signal=89% |
| 1484 | GOBP\_CELLULAR\_RESPONSE\_TO\_LEPTIN\_STIMULUS |  | 16 | -0.38 | -1.09 | 0.355 | 0.583 | 1.000 | 370 | tags=19%, list=3%, signal=19% |
| 1485 | GOBP\_REGULATION\_OF\_NIK\_NF\_KAPPAB\_SIGNALING |  | 88 | -0.26 | -1.09 | 0.283 | 0.585 | 1.000 | 2430 | tags=26%, list=19%, signal=32% |
| 1486 | GOBP\_NEGATIVE\_REGULATION\_OF\_BLOOD\_VESSEL\_ENDOTHELIAL\_CELL\_MIGRATION |  | 33 | -0.31 | -1.09 | 0.331 | 0.587 | 1.000 | 2916 | tags=30%, list=23%, signal=39% |
| 1487 | GOBP\_DETECTION\_OF\_TEMPERATURE\_STIMULUS\_INVOLVED\_IN\_SENSORY\_PERCEPTION\_OF\_PAIN |  | 16 | -0.37 | -1.09 | 0.352 | 0.586 | 1.000 | 1229 | tags=19%, list=10%, signal=21% |
| 1488 | GOBP\_HOMOLOGOUS\_CHROMOSOME\_PAIRING\_AT\_MEIOSIS |  | 32 | -0.31 | -1.09 | 0.323 | 0.586 | 1.000 | 4169 | tags=44%, list=32%, signal=64% |
| 1489 | GOBP\_REGULATION\_OF\_DIGESTIVE\_SYSTEM\_PROCESS |  | 32 | -0.32 | -1.09 | 0.307 | 0.586 | 1.000 | 4430 | tags=50%, list=34%, signal=76% |
| 1490 | GOBP\_REGULATION\_OF\_TRANSMEMBRANE\_RECEPTOR\_PROTEIN\_SERINE\_THREONINE\_KINASE\_SIGNALING\_PATHWAY |  | 205 | -0.22 | -1.09 | 0.257 | 0.587 | 1.000 | 2597 | tags=25%, list=20%, signal=31% |
| 1491 | GOBP\_PROTEIN\_METHYLATION |  | 155 | -0.24 | -1.09 | 0.273 | 0.587 | 1.000 | 2940 | tags=28%, list=23%, signal=35% |
| 1492 | GOBP\_MRNA\_CIS\_SPLICING\_VIA\_SPLICEOSOME |  | 19 | -0.36 | -1.09 | 0.344 | 0.586 | 1.000 | 1048 | tags=21%, list=8%, signal=23% |
| 1493 | GOBP\_ANTIGEN\_PROCESSING\_AND\_PRESENTATION\_OF\_ENDOGENOUS\_PEPTIDE\_ANTIGEN |  | 17 | -0.37 | -1.09 | 0.341 | 0.588 | 1.000 | 2033 | tags=35%, list=16%, signal=42% |
| 1494 | GOBP\_REGULATION\_OF\_GENE\_EXPRESSION\_EPIGENETIC |  | 117 | -0.24 | -1.09 | 0.304 | 0.589 | 1.000 | 3094 | tags=26%, list=24%, signal=33% |
| 1495 | GOBP\_RESPONSE\_TO\_OSMOTIC\_STRESS |  | 72 | -0.27 | -1.09 | 0.317 | 0.589 | 1.000 | 3752 | tags=38%, list=29%, signal=53% |
| 1496 | GOBP\_THYMUS\_DEVELOPMENT |  | 41 | -0.30 | -1.09 | 0.337 | 0.590 | 1.000 | 1035 | tags=17%, list=8%, signal=19% |
| 1497 | GOBP\_REGULATION\_OF\_ORGANIC\_ACID\_TRANSPORT |  | 65 | -0.27 | -1.09 | 0.314 | 0.590 | 1.000 | 1931 | tags=23%, list=15%, signal=27% |
| 1498 | GOBP\_REGULATION\_OF\_FATTY\_ACID\_METABOLIC\_PROCESS |  | 75 | -0.27 | -1.09 | 0.314 | 0.590 | 1.000 | 2078 | tags=23%, list=16%, signal=27% |
| 1499 | GOBP\_MODIFICATION\_DEPENDENT\_MACROMOLECULE\_CATABOLIC\_PROCESS |  | 470 | -0.21 | -1.09 | 0.237 | 0.592 | 1.000 | 2415 | tags=22%, list=19%, signal=26% |
| 1500 | GOBP\_EXTRINSIC\_APOPTOTIC\_SIGNALING\_PATHWAY |  | 197 | -0.23 | -1.09 | 0.276 | 0.591 | 1.000 | 2834 | tags=27%, list=22%, signal=34% |
| 1501 | GOBP\_POSITIVE\_REGULATION\_OF\_RECEPTOR\_INTERNALIZATION |  | 22 | -0.34 | -1.08 | 0.337 | 0.593 | 1.000 | 2052 | tags=32%, list=16%, signal=38% |
| 1502 | GOBP\_NEUROTROPHIN\_SIGNALING\_PATHWAY |  | 32 | -0.32 | -1.08 | 0.341 | 0.593 | 1.000 | 2223 | tags=28%, list=17%, signal=34% |
| 1503 | GOBP\_MICROVILLUS\_ASSEMBLY |  | 21 | -0.35 | -1.08 | 0.339 | 0.593 | 1.000 | 2807 | tags=33%, list=22%, signal=43% |
| 1504 | GOBP\_HORMONE\_MEDIATED\_SIGNALING\_PATHWAY |  | 164 | -0.23 | -1.08 | 0.276 | 0.593 | 1.000 | 4430 | tags=42%, list=34%, signal=63% |
| 1505 | GOBP\_NEGATIVE\_REGULATION\_OF\_APOPTOTIC\_SIGNALING\_PATHWAY |  | 190 | -0.23 | -1.08 | 0.277 | 0.593 | 1.000 | 2922 | tags=26%, list=23%, signal=34% |
| 1506 | GOBP\_REGULATION\_OF\_T\_CELL\_MEDIATED\_IMMUNITY |  | 81 | -0.26 | -1.08 | 0.309 | 0.595 | 1.000 | 2290 | tags=22%, list=18%, signal=27% |
| 1507 | GOBP\_CORTICOSTEROID\_HORMONE\_SECRETION |  | 15 | -0.38 | -1.08 | 0.366 | 0.595 | 1.000 | 2881 | tags=40%, list=22%, signal=51% |
| 1508 | GOBP\_ANTIGEN\_PROCESSING\_AND\_PRESENTATION\_OF\_EXOGENOUS\_PEPTIDE\_ANTIGEN\_VIA\_MHC\_CLASS\_II |  | 27 | -0.32 | -1.08 | 0.345 | 0.596 | 1.000 | 555 | tags=15%, list=4%, signal=15% |
| 1509 | GOBP\_POSITIVE\_REGULATION\_OF\_UBIQUITIN\_PROTEIN\_TRANSFERASE\_ACTIVITY |  | 25 | -0.33 | -1.08 | 0.348 | 0.597 | 1.000 | 2906 | tags=44%, list=23%, signal=57% |
| 1510 | GOBP\_REGULATION\_OF\_NUCLEOCYTOPLASMIC\_TRANSPORT |  | 96 | -0.25 | -1.08 | 0.312 | 0.598 | 1.000 | 2926 | tags=31%, list=23%, signal=40% |
| 1511 | GOBP\_RESPONSE\_TO\_MONOSACCHARIDE |  | 162 | -0.23 | -1.08 | 0.300 | 0.598 | 1.000 | 3970 | tags=37%, list=31%, signal=53% |
| 1512 | GOBP\_HYDROGEN\_PEROXIDE\_CATABOLIC\_PROCESS |  | 23 | -0.34 | -1.08 | 0.356 | 0.598 | 1.000 | 2429 | tags=39%, list=19%, signal=48% |
| 1513 | GOBP\_LIPID\_MODIFICATION |  | 178 | -0.23 | -1.08 | 0.286 | 0.598 | 1.000 | 3443 | tags=33%, list=27%, signal=44% |
| 1514 | GOBP\_MULTICELLULAR\_ORGANISM\_GROWTH |  | 123 | -0.24 | -1.08 | 0.276 | 0.598 | 1.000 | 3459 | tags=28%, list=27%, signal=39% |
| 1515 | GOBP\_MYOTUBE\_DIFFERENTIATION |  | 90 | -0.25 | -1.08 | 0.312 | 0.598 | 1.000 | 2571 | tags=26%, list=20%, signal=32% |
| 1516 | GOBP\_MULTICELLULAR\_ORGANISMAL\_RESPONSE\_TO\_STRESS |  | 68 | -0.27 | -1.08 | 0.338 | 0.599 | 1.000 | 1404 | tags=18%, list=11%, signal=20% |
| 1517 | GOBP\_C21\_STEROID\_HORMONE\_METABOLIC\_PROCESS |  | 36 | -0.30 | -1.08 | 0.350 | 0.600 | 1.000 | 1359 | tags=19%, list=11%, signal=22% |
| 1518 | GOBP\_BONE\_RESORPTION |  | 58 | -0.27 | -1.08 | 0.318 | 0.601 | 1.000 | 1672 | tags=21%, list=13%, signal=24% |
| 1519 | GOBP\_REGULATION\_OF\_DNA\_TEMPLATED\_TRANSCRIPTION\_ELONGATION |  | 49 | -0.28 | -1.08 | 0.326 | 0.603 | 1.000 | 4380 | tags=51%, list=34%, signal=77% |
| 1520 | GOBP\_NEGATIVE\_REGULATION\_OF\_PROTEOLYSIS |  | 260 | -0.22 | -1.08 | 0.272 | 0.602 | 1.000 | 3065 | tags=27%, list=24%, signal=34% |
| 1521 | GOBP\_POSITIVE\_REGULATION\_OF\_CYTOKINE\_PRODUCTION\_INVOLVED\_IN\_INFLAMMATORY\_RESPONSE |  | 19 | -0.35 | -1.08 | 0.358 | 0.602 | 1.000 | 3700 | tags=37%, list=29%, signal=52% |
| 1522 | GOBP\_POSITIVE\_REGULATION\_OF\_TRANSCRIPTION\_REGULATORY\_REGION\_DNA\_BINDING |  | 22 | -0.34 | -1.08 | 0.349 | 0.602 | 1.000 | 1206 | tags=23%, list=9%, signal=25% |
| 1523 | GOBP\_PHOSPHATIDYLINOSITOL\_PHOSPHATE\_BIOSYNTHETIC\_PROCESS |  | 51 | -0.29 | -1.08 | 0.340 | 0.602 | 1.000 | 2452 | tags=25%, list=19%, signal=31% |
| 1524 | GOBP\_NEGATIVE\_REGULATION\_OF\_LYMPHOCYTE\_ACTIVATION |  | 134 | -0.24 | -1.08 | 0.304 | 0.602 | 1.000 | 2461 | tags=25%, list=19%, signal=30% |
| 1525 | GOBP\_REGULATION\_OF\_POSTSYNAPSE\_ORGANIZATION |  | 68 | -0.26 | -1.08 | 0.331 | 0.602 | 1.000 | 4145 | tags=43%, list=32%, signal=63% |
| 1526 | GOBP\_REGULATION\_OF\_HEART\_CONTRACTION |  | 179 | -0.23 | -1.08 | 0.296 | 0.604 | 1.000 | 3082 | tags=25%, list=24%, signal=33% |
| 1527 | GOBP\_UNSATURATED\_FATTY\_ACID\_BIOSYNTHETIC\_PROCESS |  | 42 | -0.29 | -1.08 | 0.345 | 0.604 | 1.000 | 2078 | tags=26%, list=16%, signal=31% |
| 1528 | GOBP\_NEUTROPHIL\_CHEMOTAXIS |  | 94 | -0.25 | -1.08 | 0.321 | 0.606 | 1.000 | 3655 | tags=35%, list=28%, signal=49% |
| 1529 | GOBP\_POSITIVE\_REGULATION\_OF\_BINDING |  | 156 | -0.23 | -1.08 | 0.289 | 0.607 | 1.000 | 2323 | tags=22%, list=18%, signal=27% |
| 1530 | GOBP\_REGULATION\_OF\_RECEPTOR\_SIGNALING\_PATHWAY\_VIA\_STAT |  | 88 | -0.25 | -1.08 | 0.326 | 0.607 | 1.000 | 2631 | tags=23%, list=20%, signal=28% |
| 1531 | GOBP\_POSITIVE\_REGULATION\_OF\_PROTEIN\_KINASE\_B\_SIGNALING |  | 95 | -0.25 | -1.08 | 0.336 | 0.607 | 1.000 | 1710 | tags=17%, list=13%, signal=19% |
| 1532 | GOBP\_REGULATION\_OF\_RESPONSE\_TO\_ENDOPLASMIC\_RETICULUM\_STRESS |  | 67 | -0.27 | -1.08 | 0.309 | 0.607 | 1.000 | 4247 | tags=42%, list=33%, signal=62% |
| 1533 | GOBP\_PROTEIN\_AUTOPROCESSING |  | 21 | -0.35 | -1.08 | 0.364 | 0.607 | 1.000 | 3961 | tags=48%, list=31%, signal=69% |
| 1534 | GOBP\_REGULATION\_OF\_WNT\_SIGNALING\_PATHWAY |  | 247 | -0.22 | -1.08 | 0.274 | 0.607 | 1.000 | 2246 | tags=21%, list=17%, signal=25% |
| 1535 | GOBP\_POSITIVE\_REGULATION\_OF\_MUSCLE\_CELL\_DIFFERENTIATION |  | 50 | -0.28 | -1.07 | 0.329 | 0.607 | 1.000 | 1880 | tags=24%, list=15%, signal=28% |
| 1536 | GOBP\_NEGATIVE\_REGULATION\_OF\_PEPTIDYL\_LYSINE\_ACETYLATION |  | 16 | -0.37 | -1.07 | 0.374 | 0.607 | 1.000 | 3955 | tags=56%, list=31%, signal=81% |
| 1537 | GOBP\_ACTIVATION\_OF\_INNATE\_IMMUNE\_RESPONSE |  | 48 | -0.29 | -1.07 | 0.336 | 0.608 | 1.000 | 3568 | tags=35%, list=28%, signal=49% |
| 1538 | GOBP\_TRANSMEMBRANE\_RECEPTOR\_PROTEIN\_SERINE\_THREONINE\_KINASE\_SIGNALING\_PATHWAY |  | 292 | -0.21 | -1.07 | 0.281 | 0.608 | 1.000 | 4152 | tags=37%, list=32%, signal=54% |
| 1539 | GOBP\_POSITIVE\_REGULATION\_OF\_SKELETAL\_MUSCLE\_TISSUE\_DEVELOPMENT |  | 15 | -0.37 | -1.07 | 0.372 | 0.608 | 1.000 | 1403 | tags=20%, list=11%, signal=22% |
| 1540 | GOBP\_CGMP\_MEDIATED\_SIGNALING |  | 30 | -0.32 | -1.07 | 0.329 | 0.609 | 1.000 | 2188 | tags=33%, list=17%, signal=40% |
| 1541 | GOBP\_NEGATIVE\_REGULATION\_OF\_SIGNAL\_TRANSDUCTION\_IN\_ABSENCE\_OF\_LIGAND |  | 29 | -0.32 | -1.07 | 0.361 | 0.609 | 1.000 | 3205 | tags=34%, list=25%, signal=46% |
| 1542 | GOBP\_NEGATIVE\_REGULATION\_OF\_PROTEIN\_LOCALIZATION |  | 146 | -0.23 | -1.07 | 0.304 | 0.609 | 1.000 | 4054 | tags=37%, list=31%, signal=53% |
| 1543 | GOBP\_CAMERA\_TYPE\_EYE\_MORPHOGENESIS |  | 102 | -0.25 | -1.07 | 0.320 | 0.609 | 1.000 | 3763 | tags=32%, list=29%, signal=45% |
| 1544 | GOBP\_SPERM\_MOTILITY |  | 61 | -0.27 | -1.07 | 0.335 | 0.612 | 1.000 | 4349 | tags=46%, list=34%, signal=69% |
| 1545 | GOBP\_POSITIVE\_REGULATION\_OF\_EPIDERMAL\_CELL\_DIFFERENTIATION |  | 24 | -0.33 | -1.07 | 0.368 | 0.612 | 1.000 | 4219 | tags=46%, list=33%, signal=68% |
| 1546 | GOBP\_PEPTIDYL\_TYROSINE\_MODIFICATION |  | 324 | -0.21 | -1.07 | 0.281 | 0.612 | 1.000 | 2146 | tags=19%, list=17%, signal=23% |
| 1547 | GOBP\_T\_CELL\_LINEAGE\_COMMITMENT |  | 24 | -0.33 | -1.07 | 0.374 | 0.612 | 1.000 | 4711 | tags=50%, list=37%, signal=79% |
| 1548 | GOBP\_GRANULOCYTE\_CHEMOTAXIS |  | 114 | -0.24 | -1.07 | 0.338 | 0.615 | 1.000 | 3700 | tags=35%, list=29%, signal=49% |
| 1549 | GOBP\_DETECTION\_OF\_STIMULUS |  | 223 | -0.22 | -1.07 | 0.289 | 0.617 | 1.000 | 3269 | tags=28%, list=25%, signal=37% |
| 1550 | GOBP\_PLATELET\_ACTIVATION |  | 118 | -0.24 | -1.07 | 0.312 | 0.617 | 1.000 | 4694 | tags=43%, list=36%, signal=67% |
| 1551 | GOBP\_RNA\_PHOSPHODIESTER\_BOND\_HYDROLYSIS\_ENDONUCLEOLYTIC |  | 60 | -0.27 | -1.07 | 0.319 | 0.616 | 1.000 | 2636 | tags=27%, list=20%, signal=33% |
| 1552 | GOBP\_C4\_DICARBOXYLATE\_TRANSPORT |  | 23 | -0.33 | -1.07 | 0.385 | 0.617 | 1.000 | 4745 | tags=57%, list=37%, signal=89% |
| 1553 | GOBP\_LIPID\_DIGESTION |  | 15 | -0.38 | -1.07 | 0.374 | 0.617 | 1.000 | 4917 | tags=60%, list=38%, signal=97% |
| 1554 | GOBP\_REGULATION\_OF\_SIGNALING\_RECEPTOR\_ACTIVITY |  | 120 | -0.24 | -1.07 | 0.326 | 0.617 | 1.000 | 4319 | tags=48%, list=33%, signal=72% |
| 1555 | GOBP\_HISTONE\_H3\_ACETYLATION |  | 66 | -0.26 | -1.07 | 0.351 | 0.618 | 1.000 | 1683 | tags=21%, list=13%, signal=24% |
| 1556 | GOBP\_REGULATION\_OF\_CENTROSOME\_CYCLE |  | 38 | -0.30 | -1.07 | 0.341 | 0.618 | 1.000 | 3490 | tags=39%, list=27%, signal=54% |
| 1557 | GOBP\_RESPONSE\_TO\_RETINOIC\_ACID |  | 98 | -0.25 | -1.07 | 0.328 | 0.619 | 1.000 | 1880 | tags=20%, list=15%, signal=24% |
| 1558 | GOBP\_GLYCEROPHOSPHOLIPID\_BIOSYNTHETIC\_PROCESS |  | 170 | -0.23 | -1.07 | 0.303 | 0.619 | 1.000 | 2473 | tags=23%, list=19%, signal=28% |
| 1559 | GOBP\_CERAMIDE\_BIOSYNTHETIC\_PROCESS |  | 49 | -0.28 | -1.07 | 0.358 | 0.621 | 1.000 | 1774 | tags=22%, list=14%, signal=26% |
| 1560 | GOBP\_REGULATION\_OF\_ENDOTHELIAL\_CELL\_CHEMOTAXIS |  | 20 | -0.35 | -1.07 | 0.348 | 0.621 | 1.000 | 2944 | tags=35%, list=23%, signal=45% |
| 1561 | GOBP\_CENTRAL\_NERVOUS\_SYSTEM\_NEURON\_DEVELOPMENT |  | 67 | -0.26 | -1.07 | 0.334 | 0.622 | 1.000 | 2842 | tags=28%, list=22%, signal=36% |
| 1562 | GOBP\_ENDOCARDIAL\_CUSHION\_DEVELOPMENT |  | 44 | -0.29 | -1.07 | 0.379 | 0.622 | 1.000 | 1983 | tags=23%, list=15%, signal=27% |
| 1563 | GOBP\_CELLULAR\_COMPONENT\_MAINTENANCE |  | 48 | -0.28 | -1.07 | 0.366 | 0.621 | 1.000 | 4475 | tags=50%, list=35%, signal=76% |
| 1564 | GOBP\_REGULATION\_OF\_HORMONE\_SECRETION |  | 204 | -0.22 | -1.07 | 0.322 | 0.621 | 1.000 | 3867 | tags=33%, list=30%, signal=47% |
| 1565 | GOBP\_INTEGRATED\_STRESS\_RESPONSE\_SIGNALING |  | 17 | -0.36 | -1.07 | 0.371 | 0.622 | 1.000 | 4073 | tags=59%, list=32%, signal=86% |
| 1566 | GOBP\_SEGMENTATION |  | 74 | -0.26 | -1.07 | 0.340 | 0.622 | 1.000 | 4102 | tags=35%, list=32%, signal=51% |
| 1567 | GOBP\_GLYCEROLIPID\_BIOSYNTHETIC\_PROCESS |  | 194 | -0.22 | -1.06 | 0.302 | 0.622 | 1.000 | 2473 | tags=23%, list=19%, signal=28% |
| 1568 | GOBP\_REGULATION\_OF\_DNA\_METHYLATION |  | 20 | -0.35 | -1.06 | 0.370 | 0.623 | 1.000 | 101 | tags=10%, list=1%, signal=10% |
| 1569 | GOBP\_REGULATION\_OF\_MAST\_CELL\_ACTIVATION |  | 41 | -0.29 | -1.06 | 0.367 | 0.623 | 1.000 | 2000 | tags=22%, list=16%, signal=26% |
| 1570 | GOBP\_POSITIVE\_REGULATION\_OF\_CREB\_TRANSCRIPTION\_FACTOR\_ACTIVITY |  | 16 | -0.37 | -1.06 | 0.377 | 0.623 | 1.000 | 3496 | tags=38%, list=27%, signal=51% |
| 1571 | GOBP\_SENSORY\_ORGAN\_MORPHOGENESIS |  | 211 | -0.22 | -1.06 | 0.317 | 0.624 | 1.000 | 3708 | tags=32%, list=29%, signal=44% |
| 1572 | GOBP\_NEGATIVE\_REGULATION\_OF\_IMMUNE\_RESPONSE |  | 151 | -0.23 | -1.06 | 0.319 | 0.624 | 1.000 | 3716 | tags=32%, list=29%, signal=45% |
| 1573 | GOBP\_PIGMENTATION |  | 85 | -0.26 | -1.06 | 0.337 | 0.624 | 1.000 | 2052 | tags=21%, list=16%, signal=25% |
| 1574 | GOBP\_MITOTIC\_CELL\_CYCLE\_PHASE\_TRANSITION |  | 336 | -0.21 | -1.06 | 0.309 | 0.625 | 1.000 | 3272 | tags=26%, list=25%, signal=33% |
| 1575 | GOBP\_LEFT\_RIGHT\_PATTERN\_FORMATION |  | 15 | -0.38 | -1.06 | 0.403 | 0.627 | 1.000 | 3633 | tags=60%, list=28%, signal=83% |
| 1576 | GOBP\_ANION\_TRANSPORT |  | 399 | -0.21 | -1.06 | 0.302 | 0.626 | 1.000 | 2806 | tags=23%, list=22%, signal=28% |
| 1577 | GOBP\_INORGANIC\_ION\_IMPORT\_ACROSS\_PLASMA\_MEMBRANE |  | 84 | -0.25 | -1.06 | 0.341 | 0.626 | 1.000 | 4045 | tags=39%, list=31%, signal=57% |
| 1578 | GOBP\_REGULATION\_OF\_PRODUCTION\_OF\_SMALL\_RNA\_INVOLVED\_IN\_GENE\_SILENCING\_BY\_RNA |  | 20 | -0.35 | -1.06 | 0.383 | 0.626 | 1.000 | 949 | tags=20%, list=7%, signal=22% |
| 1579 | GOBP\_CORTICAL\_CYTOSKELETON\_ORGANIZATION |  | 56 | -0.28 | -1.06 | 0.350 | 0.627 | 1.000 | 2254 | tags=21%, list=17%, signal=26% |
| 1580 | GOBP\_CALCIUM\_ION\_IMPORT |  | 74 | -0.26 | -1.06 | 0.354 | 0.628 | 1.000 | 3949 | tags=39%, list=31%, signal=56% |
| 1581 | GOBP\_REGULATION\_OF\_LYMPHOCYTE\_DIFFERENTIATION |  | 153 | -0.23 | -1.06 | 0.323 | 0.628 | 1.000 | 3872 | tags=34%, list=30%, signal=48% |
| 1582 | GOBP\_REGULATION\_OF\_T\_HELPER\_1\_TYPE\_IMMUNE\_RESPONSE |  | 25 | -0.33 | -1.06 | 0.385 | 0.630 | 1.000 | 3062 | tags=36%, list=24%, signal=47% |
| 1583 | GOBP\_LEUKOCYTE\_HOMEOSTASIS |  | 73 | -0.26 | -1.06 | 0.351 | 0.630 | 1.000 | 3821 | tags=36%, list=30%, signal=50% |
| 1584 | GOBP\_REGULATION\_OF\_LIPID\_METABOLIC\_PROCESS |  | 273 | -0.21 | -1.06 | 0.313 | 0.629 | 1.000 | 2724 | tags=24%, list=21%, signal=30% |
| 1585 | GOBP\_DEFENSE\_RESPONSE\_TO\_FUNGUS |  | 38 | -0.29 | -1.06 | 0.347 | 0.629 | 1.000 | 3890 | tags=45%, list=30%, signal=64% |
| 1586 | GOBP\_RESPONSE\_TO\_ANGIOTENSIN |  | 27 | -0.33 | -1.06 | 0.384 | 0.629 | 1.000 | 856 | tags=15%, list=7%, signal=16% |
| 1587 | GOBP\_REGULATION\_OF\_NUCLEAR\_DIVISION |  | 114 | -0.24 | -1.06 | 0.333 | 0.629 | 1.000 | 2462 | tags=20%, list=19%, signal=25% |
| 1588 | GOBP\_ACTIVATION\_OF\_PROTEIN\_KINASE\_B\_ACTIVITY |  | 27 | -0.33 | -1.06 | 0.370 | 0.628 | 1.000 | 2112 | tags=30%, list=16%, signal=35% |
| 1589 | GOBP\_TYPE\_B\_PANCREATIC\_CELL\_PROLIFERATION |  | 16 | -0.36 | -1.06 | 0.391 | 0.628 | 1.000 | 2190 | tags=25%, list=17%, signal=30% |
| 1590 | GOBP\_VESICLE\_MEDIATED\_TRANSPORT\_IN\_SYNAPSE |  | 159 | -0.23 | -1.06 | 0.315 | 0.628 | 1.000 | 2977 | tags=25%, list=23%, signal=32% |
| 1591 | GOBP\_CYTOSKELETON\_DEPENDENT\_INTRACELLULAR\_TRANSPORT |  | 155 | -0.23 | -1.06 | 0.317 | 0.628 | 1.000 | 3298 | tags=31%, list=26%, signal=41% |
| 1592 | GOBP\_NEGATIVE\_REGULATION\_OF\_MAP\_KINASE\_ACTIVITY |  | 52 | -0.27 | -1.06 | 0.351 | 0.628 | 1.000 | 3439 | tags=37%, list=27%, signal=50% |
| 1593 | GOBP\_POSITIVE\_REGULATION\_OF\_DEFENSE\_RESPONSE |  | 231 | -0.22 | -1.06 | 0.324 | 0.627 | 1.000 | 3700 | tags=33%, list=29%, signal=46% |
| 1594 | GOBP\_CALCIUM\_ION\_TRANSPORT |  | 342 | -0.21 | -1.06 | 0.296 | 0.627 | 1.000 | 3118 | tags=25%, list=24%, signal=32% |
| 1595 | GOBP\_REGULATION\_OF\_T\_CELL\_MEDIATED\_CYTOTOXICITY |  | 37 | -0.30 | -1.06 | 0.360 | 0.627 | 1.000 | 2833 | tags=27%, list=22%, signal=35% |
| 1596 | GOBP\_REGULATION\_OF\_PEPTIDYL\_LYSINE\_ACETYLATION |  | 63 | -0.27 | -1.06 | 0.368 | 0.628 | 1.000 | 4095 | tags=51%, list=32%, signal=74% |
| 1597 | GOBP\_POSITIVE\_REGULATION\_OF\_INTRACELLULAR\_PROTEIN\_TRANSPORT |  | 141 | -0.23 | -1.06 | 0.338 | 0.628 | 1.000 | 2929 | tags=29%, list=23%, signal=37% |
| 1598 | GOBP\_SPHINGOLIPID\_BIOSYNTHETIC\_PROCESS |  | 80 | -0.25 | -1.06 | 0.352 | 0.628 | 1.000 | 1774 | tags=20%, list=14%, signal=23% |
| 1599 | GOBP\_REGULATION\_OF\_RAC\_PROTEIN\_SIGNAL\_TRANSDUCTION |  | 18 | -0.36 | -1.06 | 0.392 | 0.631 | 1.000 | 4949 | tags=67%, list=38%, signal=108% |
| 1600 | GOBP\_NEGATIVE\_REGULATION\_OF\_IMMUNE\_SYSTEM\_PROCESS |  | 345 | -0.21 | -1.06 | 0.316 | 0.630 | 1.000 | 2461 | tags=21%, list=19%, signal=26% |
| 1601 | GOBP\_REGULATION\_OF\_APOPTOTIC\_SIGNALING\_PATHWAY |  | 307 | -0.21 | -1.06 | 0.315 | 0.631 | 1.000 | 3242 | tags=30%, list=25%, signal=39% |
| 1602 | GOBP\_REGULATION\_OF\_LYMPHOCYTE\_MIGRATION |  | 52 | -0.28 | -1.06 | 0.363 | 0.631 | 1.000 | 3083 | tags=35%, list=24%, signal=45% |
| 1603 | GOBP\_NEGATIVE\_REGULATION\_OF\_PEPTIDYL\_THREONINE\_PHOSPHORYLATION |  | 15 | -0.37 | -1.06 | 0.380 | 0.631 | 1.000 | 3691 | tags=47%, list=29%, signal=65% |
| 1604 | GOBP\_POSITIVE\_REGULATION\_OF\_CALCIUM\_ION\_TRANSPORT |  | 105 | -0.24 | -1.06 | 0.327 | 0.631 | 1.000 | 3118 | tags=31%, list=24%, signal=41% |
| 1605 | GOBP\_ENERGY\_RESERVE\_METABOLIC\_PROCESS |  | 71 | -0.26 | -1.06 | 0.364 | 0.630 | 1.000 | 2072 | tags=20%, list=16%, signal=23% |
| 1606 | GOBP\_RESPONSE\_TO\_REACTIVE\_OXYGEN\_SPECIES |  | 175 | -0.22 | -1.06 | 0.330 | 0.632 | 1.000 | 2482 | tags=23%, list=19%, signal=28% |
| 1607 | GOBP\_REGULATION\_OF\_LIPOPROTEIN\_METABOLIC\_PROCESS |  | 15 | -0.37 | -1.06 | 0.369 | 0.633 | 1.000 | 4973 | tags=67%, list=39%, signal=108% |
| 1608 | GOBP\_NEGATIVE\_REGULATION\_OF\_RECEPTOR\_SIGNALING\_PATHWAY\_VIA\_STAT |  | 22 | -0.34 | -1.06 | 0.382 | 0.633 | 1.000 | 3100 | tags=36%, list=24%, signal=48% |
| 1609 | GOBP\_REGULATION\_OF\_GLUTAMATE\_SECRETION |  | 17 | -0.36 | -1.05 | 0.382 | 0.633 | 1.000 | 1261 | tags=24%, list=10%, signal=26% |
| 1610 | GOBP\_TELOMERE\_ORGANIZATION |  | 136 | -0.23 | -1.05 | 0.357 | 0.633 | 1.000 | 1019 | tags=12%, list=8%, signal=13% |
| 1611 | GOBP\_WOUND\_HEALING |  | 370 | -0.21 | -1.05 | 0.310 | 0.633 | 1.000 | 3303 | tags=26%, list=26%, signal=34% |
| 1612 | GOBP\_REGULATION\_OF\_AMINO\_ACID\_TRANSPORT |  | 36 | -0.30 | -1.05 | 0.374 | 0.634 | 1.000 | 1261 | tags=19%, list=10%, signal=21% |
| 1613 | GOBP\_NEGATIVE\_REGULATION\_OF\_DNA\_REPLICATION |  | 24 | -0.32 | -1.05 | 0.373 | 0.635 | 1.000 | 3679 | tags=46%, list=29%, signal=64% |
| 1614 | GOBP\_METHYLATION |  | 270 | -0.21 | -1.05 | 0.307 | 0.634 | 1.000 | 2940 | tags=26%, list=23%, signal=33% |
| 1615 | GOBP\_CENTRIOLE\_ASSEMBLY |  | 33 | -0.30 | -1.05 | 0.376 | 0.634 | 1.000 | 3490 | tags=39%, list=27%, signal=54% |
| 1616 | GOBP\_ORGAN\_INDUCTION |  | 19 | -0.34 | -1.05 | 0.363 | 0.634 | 1.000 | 129 | tags=11%, list=1%, signal=11% |
| 1617 | GOBP\_REGULATION\_OF\_POSTSYNAPTIC\_MEMBRANE\_POTENTIAL |  | 110 | -0.24 | -1.05 | 0.343 | 0.636 | 1.000 | 3015 | tags=28%, list=23%, signal=36% |
| 1618 | GOBP\_NON\_MEMBRANE\_BOUNDED\_ORGANELLE\_ASSEMBLY |  | 313 | -0.21 | -1.05 | 0.321 | 0.637 | 1.000 | 2639 | tags=21%, list=20%, signal=26% |
| 1619 | GOBP\_POSITIVE\_REGULATION\_OF\_EXTRACELLULAR\_MATRIX\_ORGANIZATION |  | 24 | -0.33 | -1.05 | 0.382 | 0.636 | 1.000 | 823 | tags=17%, list=6%, signal=18% |
| 1620 | GOBP\_POSITIVE\_REGULATION\_OF\_T\_HELPER\_1\_TYPE\_IMMUNE\_RESPONSE |  | 16 | -0.36 | -1.05 | 0.398 | 0.636 | 1.000 | 797 | tags=19%, list=6%, signal=20% |
| 1621 | GOBP\_POSITIVE\_REGULATION\_OF\_COLD\_INDUCED\_THERMOGENESIS |  | 93 | -0.25 | -1.05 | 0.348 | 0.638 | 1.000 | 4711 | tags=46%, list=37%, signal=72% |
| 1622 | GOBP\_REGULATION\_OF\_CELLULAR\_PROTEIN\_CATABOLIC\_PROCESS |  | 210 | -0.22 | -1.05 | 0.320 | 0.638 | 1.000 | 2567 | tags=25%, list=20%, signal=30% |
| 1623 | GOBP\_POSITIVE\_REGULATION\_OF\_EPIDERMIS\_DEVELOPMENT |  | 27 | -0.31 | -1.05 | 0.396 | 0.639 | 1.000 | 1756 | tags=22%, list=14%, signal=26% |
| 1624 | GOBP\_ANTIMICROBIAL\_HUMORAL\_RESPONSE |  | 96 | -0.25 | -1.05 | 0.360 | 0.639 | 1.000 | 1774 | tags=18%, list=14%, signal=20% |
| 1625 | GOBP\_REGULATION\_OF\_T\_CELL\_ACTIVATION |  | 288 | -0.21 | -1.05 | 0.322 | 0.639 | 1.000 | 2848 | tags=25%, list=22%, signal=31% |
| 1626 | GOBP\_KERATINIZATION |  | 24 | -0.33 | -1.05 | 0.405 | 0.639 | 1.000 | 6015 | tags=63%, list=47%, signal=117% |
| 1627 | GOBP\_REGULATION\_OF\_PROTEIN\_AUTOPHOSPHORYLATION |  | 36 | -0.30 | -1.05 | 0.377 | 0.638 | 1.000 | 2323 | tags=28%, list=18%, signal=34% |
| 1628 | GOBP\_REGULATION\_OF\_NEURON\_DIFFERENTIATION |  | 155 | -0.23 | -1.05 | 0.339 | 0.638 | 1.000 | 3734 | tags=34%, list=29%, signal=48% |
| 1629 | GOBP\_REGULATION\_OF\_CD4\_POSITIVE\_ALPHA\_BETA\_T\_CELL\_DIFFERENTIATION |  | 42 | -0.28 | -1.05 | 0.364 | 0.639 | 1.000 | 2078 | tags=21%, list=16%, signal=25% |
| 1630 | GOBP\_POSITIVE\_REGULATION\_OF\_NUCLEOCYTOPLASMIC\_TRANSPORT |  | 57 | -0.27 | -1.05 | 0.378 | 0.639 | 1.000 | 2926 | tags=33%, list=23%, signal=43% |
| 1631 | GOBP\_TRANSCRIPTION\_BY\_RNA\_POLYMERASE\_III |  | 44 | -0.28 | -1.05 | 0.388 | 0.640 | 1.000 | 2884 | tags=39%, list=22%, signal=50% |
| 1632 | GOBP\_MUSCLE\_SYSTEM\_PROCESS |  | 371 | -0.20 | -1.05 | 0.326 | 0.640 | 1.000 | 3046 | tags=25%, list=24%, signal=31% |
| 1633 | GOBP\_UNSATURATED\_FATTY\_ACID\_METABOLIC\_PROCESS |  | 88 | -0.24 | -1.05 | 0.375 | 0.640 | 1.000 | 2274 | tags=23%, list=18%, signal=27% |
| 1634 | GOBP\_NUCLEIC\_ACID\_PHOSPHODIESTER\_BOND\_HYDROLYSIS |  | 196 | -0.22 | -1.05 | 0.355 | 0.640 | 1.000 | 2541 | tags=24%, list=20%, signal=29% |
| 1635 | GOBP\_CLATHRIN\_DEPENDENT\_ENDOCYTOSIS |  | 40 | -0.29 | -1.05 | 0.346 | 0.640 | 1.000 | 2820 | tags=33%, list=22%, signal=41% |
| 1636 | GOBP\_NEGATIVE\_REGULATION\_OF\_EPITHELIAL\_TO\_MESENCHYMAL\_TRANSITION |  | 23 | -0.33 | -1.05 | 0.385 | 0.640 | 1.000 | 3422 | tags=39%, list=27%, signal=53% |
| 1637 | GOBP\_NEGATIVE\_REGULATION\_OF\_PEPTIDE\_SECRETION |  | 30 | -0.31 | -1.05 | 0.394 | 0.641 | 1.000 | 2112 | tags=23%, list=16%, signal=28% |
| 1638 | GOBP\_IMPORT\_INTO\_CELL |  | 191 | -0.22 | -1.05 | 0.358 | 0.641 | 1.000 | 3015 | tags=28%, list=23%, signal=36% |
| 1639 | GOBP\_T\_CELL\_ACTIVATION |  | 425 | -0.20 | -1.05 | 0.337 | 0.641 | 1.000 | 2848 | tags=24%, list=22%, signal=29% |
| 1640 | GOBP\_REGULATION\_OF\_CHROMATIN\_ORGANIZATION |  | 33 | -0.30 | -1.05 | 0.378 | 0.641 | 1.000 | 1833 | tags=18%, list=14%, signal=21% |
| 1641 | GOBP\_REGULATION\_OF\_BONE\_RESORPTION |  | 38 | -0.29 | -1.05 | 0.389 | 0.643 | 1.000 | 1672 | tags=24%, list=13%, signal=27% |
| 1642 | GOBP\_PEPTIDE\_TRANSPORT |  | 210 | -0.22 | -1.05 | 0.343 | 0.643 | 1.000 | 3836 | tags=33%, list=30%, signal=46% |
| 1643 | GOBP\_GLOMERULAR\_EPITHELIUM\_DEVELOPMENT |  | 26 | -0.32 | -1.05 | 0.385 | 0.643 | 1.000 | 5307 | tags=62%, list=41%, signal=104% |
| 1644 | GOBP\_NEGATIVE\_REGULATION\_OF\_GLUCOSE\_TRANSMEMBRANE\_TRANSPORT |  | 17 | -0.36 | -1.05 | 0.413 | 0.644 | 1.000 | 1509 | tags=24%, list=12%, signal=27% |
| 1645 | GOBP\_TRANSPORT\_OF\_VIRUS |  | 18 | -0.35 | -1.05 | 0.393 | 0.643 | 1.000 | 3681 | tags=44%, list=29%, signal=62% |
| 1646 | GOBP\_DEFENSE\_RESPONSE\_TO\_GRAM\_POSITIVE\_BACTERIUM |  | 70 | -0.25 | -1.05 | 0.372 | 0.644 | 1.000 | 3988 | tags=37%, list=31%, signal=53% |
| 1647 | GOBP\_MICROTUBULE\_CYTOSKELETON\_ORGANIZATION |  | 463 | -0.20 | -1.05 | 0.322 | 0.643 | 1.000 | 3591 | tags=29%, list=28%, signal=39% |
| 1648 | GOBP\_NEGATIVE\_REGULATION\_OF\_FAT\_CELL\_DIFFERENTIATION |  | 45 | -0.28 | -1.05 | 0.400 | 0.644 | 1.000 | 2638 | tags=29%, list=20%, signal=36% |
| 1649 | GOBP\_NEGATIVE\_REGULATION\_OF\_PROTEIN\_CONTAINING\_COMPLEX\_DISASSEMBLY |  | 69 | -0.26 | -1.05 | 0.377 | 0.644 | 1.000 | 2889 | tags=28%, list=22%, signal=35% |
| 1650 | GOBP\_POSITIVE\_REGULATION\_OF\_CELL\_CELL\_ADHESION |  | 254 | -0.21 | -1.05 | 0.351 | 0.644 | 1.000 | 3277 | tags=27%, list=25%, signal=35% |
| 1651 | GOBP\_POSITIVE\_REGULATION\_OF\_HEMOPOIESIS |  | 142 | -0.23 | -1.04 | 0.365 | 0.645 | 1.000 | 2848 | tags=24%, list=22%, signal=30% |
| 1652 | GOBP\_REGULATION\_OF\_TRANSMEMBRANE\_TRANSPORT |  | 427 | -0.20 | -1.04 | 0.332 | 0.644 | 1.000 | 3043 | tags=26%, list=24%, signal=32% |
| 1653 | GOBP\_WATER\_HOMEOSTASIS |  | 55 | -0.27 | -1.04 | 0.409 | 0.644 | 1.000 | 3303 | tags=35%, list=26%, signal=46% |
| 1654 | GOBP\_CELLULAR\_RESPONSE\_TO\_MOLECULE\_OF\_BACTERIAL\_ORIGIN |  | 187 | -0.22 | -1.04 | 0.375 | 0.645 | 1.000 | 3890 | tags=34%, list=30%, signal=48% |
| 1655 | GOBP\_POSITIVE\_REGULATION\_OF\_PROTEIN\_SERINE\_THREONINE\_KINASE\_ACTIVITY |  | 184 | -0.22 | -1.04 | 0.350 | 0.646 | 1.000 | 2848 | tags=27%, list=22%, signal=34% |
| 1656 | GOBP\_CELL\_CHEMOTAXIS |  | 273 | -0.21 | -1.04 | 0.351 | 0.646 | 1.000 | 3551 | tags=31%, list=28%, signal=42% |
| 1657 | GOBP\_DIVALENT\_INORGANIC\_CATION\_HOMEOSTASIS |  | 426 | -0.20 | -1.04 | 0.336 | 0.649 | 1.000 | 3045 | tags=25%, list=24%, signal=32% |
| 1658 | GOBP\_CHEMICAL\_SYNAPTIC\_TRANSMISSION\_POSTSYNAPTIC |  | 85 | -0.25 | -1.04 | 0.386 | 0.649 | 1.000 | 3015 | tags=29%, list=23%, signal=38% |
| 1659 | GOBP\_REGULATION\_OF\_CELLULAR\_KETONE\_METABOLIC\_PROCESS |  | 107 | -0.24 | -1.04 | 0.363 | 0.649 | 1.000 | 2078 | tags=21%, list=16%, signal=24% |
| 1660 | GOBP\_IMMUNOLOGICAL\_MEMORY\_PROCESS |  | 15 | -0.37 | -1.04 | 0.410 | 0.652 | 1.000 | 1327 | tags=20%, list=10%, signal=22% |
| 1661 | GOBP\_MODULATION\_OF\_EXCITATORY\_POSTSYNAPTIC\_POTENTIAL |  | 28 | -0.31 | -1.04 | 0.393 | 0.652 | 1.000 | 3015 | tags=32%, list=23%, signal=42% |
| 1662 | GOBP\_POSITIVE\_REGULATION\_OF\_INTERLEUKIN\_4\_PRODUCTION |  | 22 | -0.34 | -1.04 | 0.404 | 0.652 | 1.000 | 1243 | tags=18%, list=10%, signal=20% |
| 1663 | GOBP\_POSITIVE\_REGULATION\_OF\_NF\_KAPPAB\_TRANSCRIPTION\_FACTOR\_ACTIVITY |  | 138 | -0.23 | -1.04 | 0.384 | 0.652 | 1.000 | 2094 | tags=22%, list=16%, signal=26% |
| 1664 | GOBP\_REGULATION\_OF\_UBIQUITIN\_PROTEIN\_TRANSFERASE\_ACTIVITY |  | 45 | -0.28 | -1.04 | 0.381 | 0.652 | 1.000 | 3821 | tags=49%, list=30%, signal=69% |
| 1665 | GOBP\_ORGANIC\_CYCLIC\_COMPOUND\_CATABOLIC\_PROCESS |  | 399 | -0.20 | -1.04 | 0.347 | 0.652 | 1.000 | 2945 | tags=26%, list=23%, signal=32% |
| 1666 | GOBP\_DNA\_METHYLATION |  | 51 | -0.27 | -1.04 | 0.373 | 0.652 | 1.000 | 2302 | tags=24%, list=18%, signal=29% |
| 1667 | GOBP\_REGULATION\_OF\_PLATELET\_AGGREGATION |  | 25 | -0.32 | -1.04 | 0.407 | 0.652 | 1.000 | 3691 | tags=36%, list=29%, signal=50% |
| 1668 | GOBP\_RESPONSE\_TO\_PH |  | 36 | -0.29 | -1.04 | 0.388 | 0.652 | 1.000 | 4107 | tags=39%, list=32%, signal=57% |
| 1669 | GOBP\_FEEDING\_BEHAVIOR |  | 86 | -0.25 | -1.04 | 0.372 | 0.652 | 1.000 | 5068 | tags=50%, list=39%, signal=82% |
| 1670 | GOBP\_ESTABLISHMENT\_OF\_SPINDLE\_ORIENTATION |  | 31 | -0.31 | -1.04 | 0.402 | 0.652 | 1.000 | 3891 | tags=42%, list=30%, signal=60% |
| 1671 | GOBP\_AMIDE\_TRANSPORT |  | 241 | -0.21 | -1.04 | 0.347 | 0.652 | 1.000 | 3836 | tags=32%, list=30%, signal=45% |
| 1672 | GOBP\_ADAPTIVE\_THERMOGENESIS |  | 149 | -0.22 | -1.04 | 0.371 | 0.651 | 1.000 | 4231 | tags=38%, list=33%, signal=55% |
| 1673 | GOBP\_LIGAND\_GATED\_ION\_CHANNEL\_SIGNALING\_PATHWAY |  | 17 | -0.36 | -1.04 | 0.409 | 0.651 | 1.000 | 5067 | tags=65%, list=39%, signal=106% |
| 1674 | GOBP\_LYMPHOID\_PROGENITOR\_CELL\_DIFFERENTIATION |  | 19 | -0.34 | -1.04 | 0.396 | 0.652 | 1.000 | 861 | tags=21%, list=7%, signal=23% |
| 1675 | GOBP\_ADENYLATE\_CYCLASE\_ACTIVATING\_ADRENERGIC\_RECEPTOR\_SIGNALING\_PATHWAY |  | 23 | -0.33 | -1.04 | 0.423 | 0.652 | 1.000 | 3111 | tags=35%, list=24%, signal=46% |
| 1676 | GOBP\_REGULATION\_OF\_INTRINSIC\_APOPTOTIC\_SIGNALING\_PATHWAY |  | 143 | -0.23 | -1.04 | 0.366 | 0.652 | 1.000 | 1815 | tags=20%, list=14%, signal=23% |
| 1677 | GOBP\_CELLULAR\_RESPONSE\_TO\_CHEMICAL\_STRESS |  | 279 | -0.21 | -1.04 | 0.341 | 0.652 | 1.000 | 2138 | tags=19%, list=17%, signal=22% |
| 1678 | GOBP\_RESPONSE\_TO\_FLUID\_SHEAR\_STRESS |  | 32 | -0.30 | -1.04 | 0.401 | 0.651 | 1.000 | 3182 | tags=34%, list=25%, signal=46% |
| 1679 | GOBP\_POSITIVE\_REGULATION\_OF\_NIK\_NF\_KAPPAB\_SIGNALING |  | 60 | -0.26 | -1.04 | 0.394 | 0.651 | 1.000 | 2430 | tags=27%, list=19%, signal=33% |
| 1680 | GOBP\_POSITIVE\_REGULATION\_OF\_LEUKOCYTE\_MIGRATION |  | 126 | -0.23 | -1.04 | 0.385 | 0.651 | 1.000 | 4403 | tags=44%, list=34%, signal=66% |
| 1681 | GOBP\_REGULATION\_OF\_LEUKOCYTE\_MIGRATION |  | 191 | -0.22 | -1.04 | 0.373 | 0.651 | 1.000 | 4469 | tags=41%, list=35%, signal=62% |
| 1682 | GOBP\_NEGATIVE\_REGULATION\_OF\_OSTEOBLAST\_DIFFERENTIATION |  | 38 | -0.28 | -1.04 | 0.404 | 0.651 | 1.000 | 1719 | tags=21%, list=13%, signal=24% |
| 1683 | GOBP\_LYMPHOCYTE\_APOPTOTIC\_PROCESS |  | 60 | -0.26 | -1.04 | 0.401 | 0.651 | 1.000 | 3730 | tags=38%, list=29%, signal=54% |
| 1684 | GOBP\_REGULATION\_OF\_PLATELET\_ACTIVATION |  | 44 | -0.28 | -1.04 | 0.396 | 0.652 | 1.000 | 3277 | tags=30%, list=25%, signal=39% |
| 1685 | GOBP\_GLUCOSE\_METABOLIC\_PROCESS |  | 167 | -0.22 | -1.04 | 0.374 | 0.652 | 1.000 | 2901 | tags=26%, list=22%, signal=33% |
| 1686 | GOBP\_NEGATIVE\_REGULATION\_OF\_HISTONE\_METHYLATION |  | 19 | -0.34 | -1.04 | 0.396 | 0.652 | 1.000 | 4638 | tags=58%, list=36%, signal=90% |
| 1687 | GOBP\_REGULATION\_OF\_DNA\_DAMAGE\_RESPONSE\_SIGNAL\_TRANSDUCTION\_BY\_P53\_CLASS\_MEDIATOR |  | 32 | -0.30 | -1.04 | 0.399 | 0.653 | 1.000 | 1671 | tags=25%, list=13%, signal=29% |
| 1688 | GOBP\_POSITIVE\_REGULATION\_OF\_LEUKOCYTE\_CHEMOTAXIS |  | 85 | -0.25 | -1.04 | 0.374 | 0.653 | 1.000 | 2338 | tags=25%, list=18%, signal=30% |
| 1689 | GOBP\_REGULATION\_OF\_LYMPHOCYTE\_APOPTOTIC\_PROCESS |  | 42 | -0.28 | -1.04 | 0.410 | 0.654 | 1.000 | 3669 | tags=38%, list=28%, signal=53% |
| 1690 | GOBP\_PEPTIDE\_CROSS\_LINKING |  | 32 | -0.30 | -1.04 | 0.393 | 0.655 | 1.000 | 4118 | tags=41%, list=32%, signal=60% |
| 1691 | GOBP\_BIOMINERALIZATION |  | 139 | -0.23 | -1.04 | 0.379 | 0.655 | 1.000 | 2271 | tags=23%, list=18%, signal=28% |
| 1692 | GOBP\_NEGATIVE\_REGULATION\_OF\_LIPID\_CATABOLIC\_PROCESS |  | 20 | -0.34 | -1.04 | 0.434 | 0.655 | 1.000 | 2112 | tags=35%, list=16%, signal=42% |
| 1693 | GOBP\_CARDIAC\_MUSCLE\_CELL\_DIFFERENTIATION |  | 91 | -0.25 | -1.04 | 0.390 | 0.655 | 1.000 | 2103 | tags=22%, list=16%, signal=26% |
| 1694 | GOBP\_RESPONSE\_TO\_ALCOHOL |  | 213 | -0.21 | -1.04 | 0.382 | 0.655 | 1.000 | 1982 | tags=19%, list=15%, signal=22% |
| 1695 | GOBP\_POSITIVE\_REGULATION\_OF\_PROTEIN\_CATABOLIC\_PROCESS |  | 193 | -0.22 | -1.04 | 0.366 | 0.654 | 1.000 | 2267 | tags=21%, list=18%, signal=25% |
| 1696 | GOBP\_POSITIVE\_REGULATION\_OF\_TYROSINE\_PHOSPHORYLATION\_OF\_STAT\_PROTEIN |  | 54 | -0.27 | -1.04 | 0.417 | 0.654 | 1.000 | 1746 | tags=19%, list=14%, signal=21% |
| 1697 | GOBP\_MONOSACCHARIDE\_BIOSYNTHETIC\_PROCESS |  | 73 | -0.25 | -1.04 | 0.385 | 0.654 | 1.000 | 2765 | tags=26%, list=21%, signal=33% |
| 1698 | GOBP\_RAC\_PROTEIN\_SIGNAL\_TRANSDUCTION |  | 35 | -0.29 | -1.04 | 0.423 | 0.654 | 1.000 | 2442 | tags=29%, list=19%, signal=35% |
| 1699 | GOBP\_NEGATIVE\_REGULATION\_OF\_TRANSFERASE\_ACTIVITY |  | 216 | -0.21 | -1.03 | 0.375 | 0.654 | 1.000 | 2990 | tags=27%, list=23%, signal=35% |
| 1700 | GOBP\_OVULATION\_CYCLE |  | 68 | -0.26 | -1.03 | 0.382 | 0.654 | 1.000 | 2959 | tags=26%, list=23%, signal=34% |
| 1701 | GOBP\_REGULATION\_OF\_KIDNEY\_DEVELOPMENT |  | 28 | -0.31 | -1.03 | 0.406 | 0.654 | 1.000 | 626 | tags=14%, list=5%, signal=15% |
| 1702 | GOBP\_VIRAL\_PROTEIN\_PROCESSING |  | 27 | -0.31 | -1.03 | 0.410 | 0.654 | 1.000 | 2272 | tags=33%, list=18%, signal=40% |
| 1703 | GOBP\_SYNAPTIC\_MEMBRANE\_ADHESION |  | 17 | -0.35 | -1.03 | 0.420 | 0.653 | 1.000 | 1885 | tags=24%, list=15%, signal=28% |
| 1704 | GOBP\_REGULATION\_OF\_OLIGODENDROCYTE\_DIFFERENTIATION |  | 30 | -0.30 | -1.03 | 0.404 | 0.653 | 1.000 | 855 | tags=13%, list=7%, signal=14% |
| 1705 | GOBP\_NEGATIVE\_REGULATION\_OF\_BINDING |  | 134 | -0.23 | -1.03 | 0.389 | 0.653 | 1.000 | 2671 | tags=25%, list=21%, signal=32% |
| 1706 | GOBP\_INORGANIC\_ANION\_TRANSMEMBRANE\_TRANSPORT |  | 74 | -0.25 | -1.03 | 0.393 | 0.653 | 1.000 | 4443 | tags=43%, list=34%, signal=66% |
| 1707 | GOBP\_TUBULIN\_DEACETYLATION |  | 20 | -0.34 | -1.03 | 0.423 | 0.655 | 1.000 | 1403 | tags=25%, list=11%, signal=28% |
| 1708 | GOBP\_REGULATION\_OF\_CARBOHYDRATE\_CATABOLIC\_PROCESS |  | 51 | -0.27 | -1.03 | 0.386 | 0.655 | 1.000 | 2084 | tags=25%, list=16%, signal=30% |
| 1709 | GOBP\_ANTERIOR\_POSTERIOR\_AXIS\_SPECIFICATION |  | 41 | -0.29 | -1.03 | 0.413 | 0.655 | 1.000 | 4434 | tags=41%, list=34%, signal=63% |
| 1710 | GOBP\_NEGATIVE\_REGULATION\_OF\_VIRAL\_ENTRY\_INTO\_HOST\_CELL |  | 20 | -0.33 | -1.03 | 0.388 | 0.656 | 1.000 | 3727 | tags=45%, list=29%, signal=63% |
| 1711 | GOBP\_ALPHA\_BETA\_T\_CELL\_ACTIVATION |  | 129 | -0.23 | -1.03 | 0.404 | 0.656 | 1.000 | 2831 | tags=26%, list=22%, signal=33% |
| 1712 | GOBP\_FILOPODIUM\_ASSEMBLY |  | 54 | -0.27 | -1.03 | 0.399 | 0.656 | 1.000 | 2820 | tags=28%, list=22%, signal=35% |
| 1713 | GOBP\_MULTICELLULAR\_ORGANISM\_AGING |  | 37 | -0.29 | -1.03 | 0.401 | 0.656 | 1.000 | 698 | tags=14%, list=5%, signal=14% |
| 1714 | GOBP\_POSITIVE\_REGULATION\_OF\_EXCITATORY\_POSTSYNAPTIC\_POTENTIAL |  | 20 | -0.33 | -1.03 | 0.416 | 0.657 | 1.000 | 2321 | tags=30%, list=18%, signal=37% |
| 1715 | GOBP\_NEGATIVE\_REGULATION\_OF\_SYNAPTIC\_TRANSMISSION |  | 59 | -0.27 | -1.03 | 0.413 | 0.658 | 1.000 | 1931 | tags=19%, list=15%, signal=22% |
| 1716 | GOBP\_REGULATION\_OF\_PHOSPHOLIPID\_BIOSYNTHETIC\_PROCESS |  | 16 | -0.35 | -1.03 | 0.428 | 0.658 | 1.000 | 1962 | tags=25%, list=15%, signal=29% |
| 1717 | GOBP\_VENTRICULAR\_SEPTUM\_DEVELOPMENT |  | 61 | -0.26 | -1.03 | 0.393 | 0.658 | 1.000 | 4102 | tags=34%, list=32%, signal=50% |
| 1718 | GOBP\_REGULATION\_OF\_SYSTEMIC\_ARTERIAL\_BLOOD\_PRESSURE\_MEDIATED\_BY\_A\_CHEMICAL\_SIGNAL |  | 46 | -0.28 | -1.03 | 0.407 | 0.658 | 1.000 | 4505 | tags=50%, list=35%, signal=77% |
| 1719 | GOBP\_TRABECULA\_MORPHOGENESIS |  | 43 | -0.27 | -1.03 | 0.408 | 0.660 | 1.000 | 5202 | tags=56%, list=40%, signal=93% |
| 1720 | GOBP\_GLAND\_MORPHOGENESIS |  | 110 | -0.23 | -1.03 | 0.378 | 0.660 | 1.000 | 2428 | tags=22%, list=19%, signal=27% |
| 1721 | GOBP\_NEUROBLAST\_PROLIFERATION |  | 47 | -0.27 | -1.03 | 0.413 | 0.660 | 1.000 | 2094 | tags=21%, list=16%, signal=25% |
| 1722 | GOBP\_RESPONSE\_TO\_PROTOZOAN |  | 20 | -0.34 | -1.03 | 0.422 | 0.661 | 1.000 | 5812 | tags=60%, list=45%, signal=109% |
| 1723 | GOBP\_CELL\_CYCLE\_CHECKPOINT\_SIGNALING |  | 137 | -0.22 | -1.03 | 0.384 | 0.664 | 1.000 | 2469 | tags=20%, list=19%, signal=25% |
| 1724 | GOBP\_REGULATION\_OF\_OSTEOCLAST\_DIFFERENTIATION |  | 55 | -0.26 | -1.03 | 0.416 | 0.665 | 1.000 | 2271 | tags=24%, list=18%, signal=29% |
| 1725 | GOBP\_REGULATION\_OF\_ACTOMYOSIN\_STRUCTURE\_ORGANIZATION |  | 88 | -0.24 | -1.03 | 0.397 | 0.664 | 1.000 | 1803 | tags=19%, list=14%, signal=22% |
| 1726 | GOBP\_GAMMA\_DELTA\_T\_CELL\_ACTIVATION |  | 19 | -0.34 | -1.03 | 0.407 | 0.664 | 1.000 | 1863 | tags=21%, list=14%, signal=25% |
| 1727 | GOBP\_POSITIVE\_REGULATION\_OF\_PROTEOLYSIS |  | 310 | -0.20 | -1.03 | 0.389 | 0.666 | 1.000 | 2575 | tags=23%, list=20%, signal=28% |
| 1728 | GOBP\_RNA\_PHOSPHODIESTER\_BOND\_HYDROLYSIS |  | 111 | -0.23 | -1.03 | 0.398 | 0.666 | 1.000 | 2937 | tags=28%, list=23%, signal=36% |
| 1729 | GOBP\_POSITIVE\_REGULATION\_OF\_AXONOGENESIS |  | 66 | -0.26 | -1.03 | 0.408 | 0.667 | 1.000 | 3172 | tags=27%, list=25%, signal=36% |
| 1730 | GOBP\_LIMBIC\_SYSTEM\_DEVELOPMENT |  | 92 | -0.24 | -1.03 | 0.439 | 0.668 | 1.000 | 1917 | tags=18%, list=15%, signal=22% |
| 1731 | GOBP\_RESPONSE\_TO\_IRON\_ION |  | 29 | -0.30 | -1.03 | 0.416 | 0.669 | 1.000 | 4845 | tags=59%, list=38%, signal=94% |
| 1732 | GOBP\_PHOSPHATIDYLINOSITOL\_3\_KINASE\_SIGNALING |  | 126 | -0.23 | -1.03 | 0.406 | 0.669 | 1.000 | 2076 | tags=20%, list=16%, signal=23% |
| 1733 | GOBP\_POSITIVE\_REGULATION\_OF\_I\_KAPPAB\_KINASE\_NF\_KAPPAB\_SIGNALING |  | 155 | -0.22 | -1.03 | 0.397 | 0.669 | 1.000 | 2933 | tags=27%, list=23%, signal=35% |
| 1734 | GOBP\_NEGATIVE\_REGULATION\_OF\_MACROAUTOPHAGY |  | 28 | -0.30 | -1.02 | 0.440 | 0.669 | 1.000 | 1740 | tags=21%, list=13%, signal=25% |
| 1735 | GOBP\_REGULATION\_OF\_NUCLEOTIDE\_METABOLIC\_PROCESS |  | 75 | -0.25 | -1.02 | 0.405 | 0.669 | 1.000 | 3666 | tags=40%, list=28%, signal=56% |
| 1736 | GOBP\_GLOMERULAR\_EPITHELIAL\_CELL\_DIFFERENTIATION |  | 23 | -0.32 | -1.02 | 0.424 | 0.674 | 1.000 | 5307 | tags=65%, list=41%, signal=111% |
| 1737 | GOBP\_POSITIVE\_REGULATION\_OF\_LYMPHOCYTE\_CHEMOTAXIS |  | 18 | -0.34 | -1.02 | 0.421 | 0.675 | 1.000 | 2338 | tags=33%, list=18%, signal=41% |
| 1738 | GOBP\_NEGATIVE\_REGULATION\_OF\_CALCIUM\_ION\_TRANSPORT |  | 52 | -0.26 | -1.02 | 0.419 | 0.676 | 1.000 | 765 | tags=12%, list=6%, signal=12% |
| 1739 | GOBP\_GOLGI\_TO\_ENDOSOME\_TRANSPORT |  | 15 | -0.37 | -1.02 | 0.446 | 0.676 | 1.000 | 1337 | tags=20%, list=10%, signal=22% |
| 1740 | GOBP\_REGIONALIZATION |  | 270 | -0.20 | -1.02 | 0.406 | 0.680 | 1.000 | 3108 | tags=26%, list=24%, signal=33% |
| 1741 | GOBP\_POSITIVE\_REGULATION\_OF\_MORPHOGENESIS\_OF\_AN\_EPITHELIUM |  | 31 | -0.30 | -1.02 | 0.442 | 0.680 | 1.000 | 1376 | tags=19%, list=11%, signal=22% |
| 1742 | GOBP\_BONE\_CELL\_DEVELOPMENT |  | 30 | -0.30 | -1.02 | 0.411 | 0.680 | 1.000 | 2182 | tags=27%, list=17%, signal=32% |
| 1743 | GOBP\_GLYCEROPHOSPHOLIPID\_METABOLIC\_PROCESS |  | 238 | -0.21 | -1.02 | 0.419 | 0.682 | 1.000 | 2473 | tags=21%, list=19%, signal=26% |
| 1744 | GOBP\_RESPONSE\_TO\_GAMMA\_RADIATION |  | 51 | -0.27 | -1.02 | 0.417 | 0.682 | 1.000 | 1713 | tags=20%, list=13%, signal=23% |
| 1745 | GOBP\_INNER\_EAR\_RECEPTOR\_CELL\_STEREOCILIUM\_ORGANIZATION |  | 18 | -0.34 | -1.02 | 0.442 | 0.682 | 1.000 | 3631 | tags=39%, list=28%, signal=54% |
| 1746 | GOBP\_PROTEIN\_DNA\_COMPLEX\_ASSEMBLY |  | 154 | -0.22 | -1.02 | 0.401 | 0.682 | 1.000 | 1217 | tags=13%, list=9%, signal=14% |
| 1747 | GOBP\_NEGATIVE\_REGULATION\_OF\_SMALL\_MOLECULE\_METABOLIC\_PROCESS |  | 78 | -0.24 | -1.02 | 0.421 | 0.682 | 1.000 | 2084 | tags=21%, list=16%, signal=24% |
| 1748 | GOBP\_INTERLEUKIN\_6\_PRODUCTION |  | 125 | -0.23 | -1.02 | 0.422 | 0.682 | 1.000 | 2124 | tags=20%, list=16%, signal=24% |
| 1749 | GOBP\_NEGATIVE\_REGULATION\_OF\_OXIDATIVE\_STRESS\_INDUCED\_CELL\_DEATH |  | 43 | -0.28 | -1.02 | 0.419 | 0.683 | 1.000 | 1327 | tags=21%, list=10%, signal=23% |
| 1750 | GOBP\_POSITIVE\_REGULATION\_OF\_JUN\_KINASE\_ACTIVITY |  | 39 | -0.28 | -1.02 | 0.446 | 0.684 | 1.000 | 1979 | tags=23%, list=15%, signal=27% |
| 1751 | GOBP\_CALCIUM\_MEDIATED\_SIGNALING |  | 175 | -0.22 | -1.02 | 0.405 | 0.684 | 1.000 | 2323 | tags=22%, list=18%, signal=26% |
| 1752 | GOBP\_REGULATION\_OF\_SMOOTH\_MUSCLE\_CELL\_DIFFERENTIATION |  | 24 | -0.32 | -1.02 | 0.448 | 0.685 | 1.000 | 662 | tags=17%, list=5%, signal=18% |
| 1753 | GOBP\_MONOSACCHARIDE\_METABOLIC\_PROCESS |  | 216 | -0.21 | -1.02 | 0.436 | 0.686 | 1.000 | 2072 | tags=20%, list=16%, signal=24% |
| 1754 | GOBP\_REGULATION\_OF\_OXIDATIVE\_STRESS\_INDUCED\_CELL\_DEATH |  | 64 | -0.26 | -1.02 | 0.416 | 0.686 | 1.000 | 1961 | tags=23%, list=15%, signal=28% |
| 1755 | GOBP\_PROTEASOME\_MEDIATED\_UBIQUITIN\_DEPENDENT\_PROTEIN\_CATABOLIC\_PROCESS |  | 309 | -0.20 | -1.02 | 0.414 | 0.687 | 1.000 | 2415 | tags=22%, list=19%, signal=26% |
| 1756 | GOBP\_RESPONSE\_TO\_ESTROGEN |  | 59 | -0.26 | -1.02 | 0.434 | 0.686 | 1.000 | 1519 | tags=20%, list=12%, signal=23% |
| 1757 | GOBP\_PERICARDIUM\_DEVELOPMENT |  | 15 | -0.35 | -1.02 | 0.416 | 0.686 | 1.000 | 861 | tags=20%, list=7%, signal=21% |
| 1758 | GOBP\_POSITIVE\_REGULATION\_OF\_POTASSIUM\_ION\_TRANSMEMBRANE\_TRANSPORTER\_ACTIVITY |  | 18 | -0.34 | -1.02 | 0.414 | 0.686 | 1.000 | 2307 | tags=33%, list=18%, signal=41% |
| 1759 | GOBP\_MAMMARY\_GLAND\_DEVELOPMENT |  | 118 | -0.23 | -1.01 | 0.420 | 0.689 | 1.000 | 2969 | tags=25%, list=23%, signal=33% |
| 1760 | GOBP\_NEGATIVE\_REGULATION\_OF\_PHOSPHORUS\_METABOLIC\_PROCESS |  | 352 | -0.20 | -1.01 | 0.413 | 0.690 | 1.000 | 2103 | tags=18%, list=16%, signal=21% |
| 1761 | GOBP\_GLUTAMATE\_SECRETION |  | 23 | -0.31 | -1.01 | 0.428 | 0.691 | 1.000 | 1261 | tags=17%, list=10%, signal=19% |
| 1762 | GOBP\_INTERMEDIATE\_FILAMENT\_ORGANIZATION |  | 37 | -0.28 | -1.01 | 0.438 | 0.691 | 1.000 | 4139 | tags=35%, list=32%, signal=52% |
| 1763 | GOBP\_REGULATION\_OF\_SYSTEMIC\_ARTERIAL\_BLOOD\_PRESSURE\_BY\_HORMONE |  | 36 | -0.28 | -1.01 | 0.438 | 0.691 | 1.000 | 4505 | tags=53%, list=35%, signal=81% |
| 1764 | GOBP\_REGULATION\_OF\_VIRAL\_PROCESS |  | 140 | -0.22 | -1.01 | 0.422 | 0.692 | 1.000 | 3165 | tags=29%, list=25%, signal=38% |
| 1765 | GOBP\_NEGATIVE\_REGULATION\_OF\_PROTEIN\_BINDING |  | 76 | -0.24 | -1.01 | 0.432 | 0.692 | 1.000 | 1792 | tags=21%, list=14%, signal=24% |
| 1766 | GOBP\_RESPONSE\_TO\_AXON\_INJURY |  | 71 | -0.25 | -1.01 | 0.409 | 0.692 | 1.000 | 1885 | tags=21%, list=15%, signal=25% |
| 1767 | GOBP\_REGULATION\_OF\_PEPTIDYL\_SERINE\_PHOSPHORYLATION\_OF\_STAT\_PROTEIN |  | 17 | -0.35 | -1.01 | 0.447 | 0.693 | 1.000 | 4369 | tags=41%, list=34%, signal=62% |
| 1768 | GOBP\_SPLEEN\_DEVELOPMENT |  | 27 | -0.30 | -1.01 | 0.442 | 0.693 | 1.000 | 2578 | tags=33%, list=20%, signal=42% |
| 1769 | GOBP\_HISTONE\_UBIQUITINATION |  | 38 | -0.28 | -1.01 | 0.459 | 0.697 | 1.000 | 2798 | tags=24%, list=22%, signal=30% |
| 1770 | GOBP\_CELL\_CELL\_ADHESION\_MEDIATED\_BY\_INTEGRIN |  | 15 | -0.36 | -1.01 | 0.448 | 0.697 | 1.000 | 2996 | tags=40%, list=23%, signal=52% |
| 1771 | GOBP\_SERINE\_PHOSPHORYLATION\_OF\_STAT\_PROTEIN |  | 21 | -0.32 | -1.01 | 0.434 | 0.697 | 1.000 | 4369 | tags=38%, list=34%, signal=58% |
| 1772 | GOBP\_EXTERNAL\_ENCAPSULATING\_STRUCTURE\_ORGANIZATION |  | 254 | -0.20 | -1.01 | 0.432 | 0.697 | 1.000 | 2260 | tags=20%, list=18%, signal=24% |
| 1773 | GOBP\_ACTIN\_FILAMENT\_DEPOLYMERIZATION |  | 47 | -0.27 | -1.01 | 0.433 | 0.697 | 1.000 | 3691 | tags=30%, list=29%, signal=42% |
| 1774 | GOBP\_RAB\_PROTEIN\_SIGNAL\_TRANSDUCTION |  | 17 | -0.34 | -1.01 | 0.439 | 0.697 | 1.000 | 1722 | tags=29%, list=13%, signal=34% |
| 1775 | GOBP\_VASODILATION |  | 46 | -0.27 | -1.01 | 0.428 | 0.696 | 1.000 | 2126 | tags=30%, list=16%, signal=36% |
| 1776 | GOBP\_REGULATION\_OF\_HISTONE\_METHYLATION |  | 64 | -0.26 | -1.01 | 0.454 | 0.697 | 1.000 | 2862 | tags=25%, list=22%, signal=32% |
| 1777 | GOBP\_CENTROSOME\_DUPLICATION |  | 55 | -0.26 | -1.01 | 0.408 | 0.699 | 1.000 | 3490 | tags=36%, list=27%, signal=50% |
| 1778 | GOBP\_POSITIVE\_REGULATION\_OF\_EMBRYONIC\_DEVELOPMENT |  | 15 | -0.36 | -1.01 | 0.451 | 0.699 | 1.000 | 2196 | tags=33%, list=17%, signal=40% |
| 1779 | GOBP\_POSITIVE\_REGULATION\_OF\_CELLULAR\_COMPONENT\_BIOGENESIS |  | 408 | -0.19 | -1.01 | 0.448 | 0.698 | 1.000 | 3579 | tags=28%, list=28%, signal=38% |
| 1780 | GOBP\_APPENDAGE\_DEVELOPMENT |  | 145 | -0.22 | -1.01 | 0.433 | 0.698 | 1.000 | 3636 | tags=34%, list=28%, signal=47% |
| 1781 | GOBP\_CELLULAR\_RESPONSE\_TO\_KETONE |  | 79 | -0.24 | -1.01 | 0.439 | 0.698 | 1.000 | 3346 | tags=33%, list=26%, signal=44% |
| 1782 | GOBP\_NEGATIVE\_REGULATION\_OF\_LIPID\_METABOLIC\_PROCESS |  | 78 | -0.24 | -1.01 | 0.471 | 0.698 | 1.000 | 2280 | tags=24%, list=18%, signal=29% |
| 1783 | GOBP\_REGULATION\_OF\_BONE\_MINERALIZATION |  | 67 | -0.25 | -1.01 | 0.427 | 0.698 | 1.000 | 2074 | tags=24%, list=16%, signal=28% |
| 1784 | GOBP\_CELLULAR\_EXTRAVASATION |  | 66 | -0.25 | -1.01 | 0.433 | 0.698 | 1.000 | 3526 | tags=36%, list=27%, signal=50% |
| 1785 | GOBP\_POSITIVE\_REGULATION\_OF\_LIPID\_KINASE\_ACTIVITY |  | 34 | -0.29 | -1.01 | 0.437 | 0.698 | 1.000 | 2437 | tags=24%, list=19%, signal=29% |
| 1786 | GOBP\_RESPONSE\_TO\_CALCIUM\_ION |  | 119 | -0.22 | -1.01 | 0.437 | 0.698 | 1.000 | 1739 | tags=17%, list=13%, signal=19% |
| 1787 | GOBP\_MAMMARY\_GLAND\_ALVEOLUS\_DEVELOPMENT |  | 19 | -0.33 | -1.01 | 0.449 | 0.698 | 1.000 | 1069 | tags=21%, list=8%, signal=23% |
| 1788 | GOBP\_FOAM\_CELL\_DIFFERENTIATION |  | 36 | -0.28 | -1.01 | 0.444 | 0.699 | 1.000 | 3289 | tags=28%, list=25%, signal=37% |
| 1789 | GOBP\_LEUKOCYTE\_APOPTOTIC\_PROCESS |  | 92 | -0.24 | -1.01 | 0.457 | 0.699 | 1.000 | 3730 | tags=35%, list=29%, signal=49% |
| 1790 | GOBP\_POSITIVE\_REGULATION\_OF\_RESPONSE\_TO\_CYTOKINE\_STIMULUS |  | 50 | -0.26 | -1.01 | 0.440 | 0.699 | 1.000 | 3617 | tags=38%, list=28%, signal=53% |
| 1791 | GOBP\_NEGATIVE\_REGULATION\_OF\_TRANSMEMBRANE\_RECEPTOR\_PROTEIN\_SERINE\_THREONINE\_KINASE\_SIGNALING\_PATHWAY |  | 92 | -0.23 | -1.01 | 0.445 | 0.702 | 1.000 | 2301 | tags=23%, list=18%, signal=28% |
| 1792 | GOBP\_CALCIUM\_ION\_IMPORT\_INTO\_CYTOSOL |  | 20 | -0.32 | -1.01 | 0.445 | 0.702 | 1.000 | 3081 | tags=35%, list=24%, signal=46% |
| 1793 | GOBP\_NEGATIVE\_REGULATION\_OF\_GTPASE\_ACTIVITY |  | 25 | -0.31 | -1.00 | 0.471 | 0.702 | 1.000 | 2425 | tags=28%, list=19%, signal=34% |
| 1794 | GOBP\_COPULATION |  | 21 | -0.32 | -1.00 | 0.445 | 0.702 | 1.000 | 3832 | tags=38%, list=30%, signal=54% |
| 1795 | GOBP\_NEGATIVE\_REGULATION\_OF\_TRANSFORMING\_GROWTH\_FACTOR\_BETA\_RECEPTOR\_SIGNALING\_PATHWAY |  | 57 | -0.25 | -1.00 | 0.462 | 0.703 | 1.000 | 2301 | tags=25%, list=18%, signal=30% |
| 1796 | GOBP\_POSITIVE\_REGULATION\_OF\_PROTEIN\_AUTOPHOSPHORYLATION |  | 24 | -0.31 | -1.00 | 0.445 | 0.703 | 1.000 | 2323 | tags=33%, list=18%, signal=41% |
| 1797 | GOBP\_REGULATION\_OF\_BEHAVIOR |  | 60 | -0.25 | -1.00 | 0.461 | 0.703 | 1.000 | 1931 | tags=20%, list=15%, signal=23% |
| 1798 | GOBP\_DNA\_DEPENDENT\_DNA\_REPLICATION |  | 130 | -0.22 | -1.00 | 0.467 | 0.704 | 1.000 | 2938 | tags=31%, list=23%, signal=39% |
| 1799 | GOBP\_RECEPTOR\_SIGNALING\_PATHWAY\_VIA\_STAT |  | 146 | -0.22 | -1.00 | 0.455 | 0.707 | 1.000 | 2146 | tags=18%, list=17%, signal=22% |
| 1800 | GOBP\_POSITIVE\_REGULATION\_OF\_ORGAN\_GROWTH |  | 40 | -0.28 | -1.00 | 0.460 | 0.706 | 1.000 | 2982 | tags=28%, list=23%, signal=36% |
| 1801 | GOBP\_REGULATION\_OF\_INTRACELLULAR\_PROTEIN\_TRANSPORT |  | 196 | -0.21 | -1.00 | 0.443 | 0.706 | 1.000 | 2929 | tags=27%, list=23%, signal=34% |
| 1802 | GOBP\_REGULATION\_OF\_T\_CELL\_PROLIFERATION |  | 146 | -0.22 | -1.00 | 0.456 | 0.706 | 1.000 | 2809 | tags=24%, list=22%, signal=30% |
| 1803 | GOBP\_PHOSPHOLIPID\_BIOSYNTHETIC\_PROCESS |  | 204 | -0.21 | -1.00 | 0.431 | 0.706 | 1.000 | 2473 | tags=22%, list=19%, signal=27% |
| 1804 | GOBP\_CARBOHYDRATE\_HOMEOSTASIS |  | 196 | -0.21 | -1.00 | 0.414 | 0.706 | 1.000 | 3955 | tags=36%, list=31%, signal=51% |
| 1805 | GOBP\_REGULATION\_OF\_CYSTEINE\_TYPE\_ENDOPEPTIDASE\_ACTIVITY |  | 199 | -0.21 | -1.00 | 0.446 | 0.707 | 1.000 | 2460 | tags=21%, list=19%, signal=26% |
| 1806 | GOBP\_EXTRACELLULAR\_MATRIX\_DISASSEMBLY |  | 53 | -0.26 | -1.00 | 0.452 | 0.707 | 1.000 | 2260 | tags=19%, list=18%, signal=23% |
| 1807 | GOBP\_NEGATIVE\_REGULATION\_OF\_CELL\_SUBSTRATE\_JUNCTION\_ORGANIZATION |  | 15 | -0.35 | -1.00 | 0.428 | 0.707 | 1.000 | 2927 | tags=47%, list=23%, signal=60% |
| 1808 | GOBP\_RESPONSE\_TO\_UV |  | 120 | -0.22 | -1.00 | 0.459 | 0.707 | 1.000 | 2482 | tags=22%, list=19%, signal=27% |
| 1809 | GOBP\_REGULATION\_OF\_GLYCOPROTEIN\_METABOLIC\_PROCESS |  | 42 | -0.27 | -1.00 | 0.453 | 0.708 | 1.000 | 4136 | tags=45%, list=32%, signal=66% |
| 1810 | GOBP\_GLUCAN\_BIOSYNTHETIC\_PROCESS |  | 36 | -0.28 | -1.00 | 0.468 | 0.708 | 1.000 | 2765 | tags=28%, list=21%, signal=35% |
| 1811 | GOBP\_C21\_STEROID\_HORMONE\_BIOSYNTHETIC\_PROCESS |  | 20 | -0.33 | -1.00 | 0.472 | 0.708 | 1.000 | 2951 | tags=40%, list=23%, signal=52% |
| 1812 | GOBP\_RESPONSE\_TO\_LEPTIN |  | 20 | -0.32 | -1.00 | 0.464 | 0.709 | 1.000 | 370 | tags=15%, list=3%, signal=15% |
| 1813 | GOBP\_RESPONSE\_TO\_NUTRIENT |  | 140 | -0.22 | -1.00 | 0.456 | 0.709 | 1.000 | 2782 | tags=26%, list=22%, signal=33% |
| 1814 | GOBP\_MATING\_BEHAVIOR |  | 16 | -0.35 | -1.00 | 0.484 | 0.710 | 1.000 | 1066 | tags=19%, list=8%, signal=20% |
| 1815 | GOBP\_REGULATION\_OF\_NUCLEASE\_ACTIVITY |  | 21 | -0.31 | -1.00 | 0.480 | 0.710 | 1.000 | 1939 | tags=24%, list=15%, signal=28% |
| 1816 | GOBP\_RESPONSE\_TO\_INORGANIC\_SUBSTANCE |  | 463 | -0.19 | -1.00 | 0.462 | 0.710 | 1.000 | 1750 | tags=16%, list=14%, signal=18% |
| 1817 | GOBP\_REGULATION\_OF\_CELLULAR\_COMPONENT\_SIZE |  | 289 | -0.20 | -1.00 | 0.476 | 0.711 | 1.000 | 4069 | tags=34%, list=32%, signal=49% |
| 1818 | GOBP\_PARASYMPATHETIC\_NERVOUS\_SYSTEM\_DEVELOPMENT |  | 17 | -0.34 | -1.00 | 0.451 | 0.711 | 1.000 | 2607 | tags=29%, list=20%, signal=37% |
| 1819 | GOBP\_POSITIVE\_REGULATION\_OF\_PROTEIN\_LOCALIZATION |  | 391 | -0.19 | -1.00 | 0.461 | 0.713 | 1.000 | 2929 | tags=25%, list=23%, signal=31% |
| 1820 | GOBP\_REGULATION\_OF\_B\_CELL\_APOPTOTIC\_PROCESS |  | 15 | -0.35 | -1.00 | 0.469 | 0.713 | 1.000 | 1512 | tags=27%, list=12%, signal=30% |
| 1821 | GOBP\_EATING\_BEHAVIOR |  | 32 | -0.29 | -1.00 | 0.470 | 0.714 | 1.000 | 5068 | tags=56%, list=39%, signal=92% |
| 1822 | GOBP\_DNA\_REPAIR |  | 410 | -0.19 | -1.00 | 0.458 | 0.713 | 1.000 | 2275 | tags=20%, list=18%, signal=23% |
| 1823 | GOBP\_REGULATION\_OF\_T\_CELL\_DIFFERENTIATION |  | 128 | -0.22 | -1.00 | 0.450 | 0.713 | 1.000 | 3872 | tags=33%, list=30%, signal=46% |
| 1824 | GOBP\_CELLULAR\_RESPONSE\_TO\_REACTIVE\_OXYGEN\_SPECIES |  | 125 | -0.22 | -1.00 | 0.473 | 0.713 | 1.000 | 2078 | tags=20%, list=16%, signal=24% |
| 1825 | GOBP\_ESTABLISHMENT\_OF\_PROTEIN\_LOCALIZATION\_TO\_ORGANELLE |  | 351 | -0.19 | -1.00 | 0.461 | 0.713 | 1.000 | 2926 | tags=25%, list=23%, signal=32% |
| 1826 | GOBP\_REGULATION\_OF\_TELOMERASE\_ACTIVITY |  | 43 | -0.27 | -1.00 | 0.461 | 0.713 | 1.000 | 693 | tags=12%, list=5%, signal=12% |
| 1827 | GOBP\_BIOLOGICAL\_PROCESS\_INVOLVED\_IN\_INTRASPECIES\_INTERACTION\_BETWEEN\_ORGANISMS |  | 47 | -0.27 | -1.00 | 0.462 | 0.714 | 1.000 | 2681 | tags=26%, list=21%, signal=32% |
| 1828 | GOBP\_INOSITOL\_PHOSPHATE\_MEDIATED\_SIGNALING |  | 48 | -0.27 | -1.00 | 0.444 | 0.713 | 1.000 | 1962 | tags=19%, list=15%, signal=22% |
| 1829 | GOBP\_REGULATION\_OF\_CILIUM\_ASSEMBLY |  | 65 | -0.25 | -1.00 | 0.453 | 0.713 | 1.000 | 3312 | tags=32%, list=26%, signal=43% |
| 1830 | GOBP\_POSITIVE\_REGULATION\_OF\_HEART\_RATE |  | 25 | -0.31 | -1.00 | 0.474 | 0.713 | 1.000 | 4797 | tags=60%, list=37%, signal=95% |
| 1831 | GOBP\_CARDIAC\_SEPTUM\_DEVELOPMENT |  | 91 | -0.23 | -1.00 | 0.457 | 0.714 | 1.000 | 4102 | tags=34%, list=32%, signal=50% |
| 1832 | GOBP\_CELLULAR\_PROCESS\_INVOLVED\_IN\_REPRODUCTION\_IN\_MULTICELLULAR\_ORGANISM |  | 265 | -0.20 | -1.00 | 0.467 | 0.714 | 1.000 | 2698 | tags=24%, list=21%, signal=30% |
| 1833 | GOBP\_INTERLEUKIN\_17\_PRODUCTION |  | 33 | -0.29 | -1.00 | 0.463 | 0.714 | 1.000 | 699 | tags=12%, list=5%, signal=13% |
| 1834 | GOBP\_REGULATION\_OF\_ACTIN\_FILAMENT\_BASED\_PROCESS |  | 328 | -0.20 | -0.99 | 0.474 | 0.715 | 1.000 | 3951 | tags=33%, list=31%, signal=46% |
| 1835 | GOBP\_AXONEMAL\_DYNEIN\_COMPLEX\_ASSEMBLY |  | 18 | -0.33 | -0.99 | 0.449 | 0.715 | 1.000 | 3008 | tags=33%, list=23%, signal=43% |
| 1836 | GOBP\_MESENCHYME\_MORPHOGENESIS |  | 49 | -0.26 | -0.99 | 0.464 | 0.715 | 1.000 | 1206 | tags=16%, list=9%, signal=18% |
| 1837 | GOBP\_REGULATION\_OF\_MAP\_KINASE\_ACTIVITY |  | 170 | -0.21 | -0.99 | 0.481 | 0.716 | 1.000 | 2587 | tags=24%, list=20%, signal=29% |
| 1838 | GOBP\_REGULATION\_OF\_DNA\_REPAIR |  | 116 | -0.22 | -0.99 | 0.478 | 0.717 | 1.000 | 2469 | tags=22%, list=19%, signal=27% |
| 1839 | GOBP\_HOMOTYPIC\_CELL\_CELL\_ADHESION |  | 80 | -0.24 | -0.99 | 0.472 | 0.717 | 1.000 | 4172 | tags=36%, list=32%, signal=53% |
| 1840 | GOBP\_PLASMINOGEN\_ACTIVATION |  | 25 | -0.30 | -0.99 | 0.459 | 0.717 | 1.000 | 4389 | tags=52%, list=34%, signal=79% |
| 1841 | GOBP\_CELLULAR\_SODIUM\_ION\_HOMEOSTASIS |  | 20 | -0.33 | -0.99 | 0.479 | 0.718 | 1.000 | 4684 | tags=55%, list=36%, signal=86% |
| 1842 | GOBP\_CARDIAC\_NEURAL\_CREST\_CELL\_DIFFERENTIATION\_INVOLVED\_IN\_HEART\_DEVELOPMENT |  | 16 | -0.35 | -0.99 | 0.471 | 0.719 | 1.000 | 1925 | tags=31%, list=15%, signal=37% |
| 1843 | GOBP\_POSITIVE\_REGULATION\_OF\_PRODUCTION\_OF\_MOLECULAR\_MEDIATOR\_OF\_IMMUNE\_RESPONSE |  | 106 | -0.23 | -0.99 | 0.475 | 0.720 | 1.000 | 797 | tags=11%, list=6%, signal=12% |
| 1844 | GOBP\_SCHWANN\_CELL\_DIFFERENTIATION |  | 34 | -0.28 | -0.99 | 0.466 | 0.720 | 1.000 | 2743 | tags=24%, list=21%, signal=30% |
| 1845 | GOBP\_CELL\_DEATH\_IN\_RESPONSE\_TO\_OXIDATIVE\_STRESS |  | 81 | -0.23 | -0.99 | 0.473 | 0.723 | 1.000 | 1979 | tags=21%, list=15%, signal=25% |
| 1846 | GOBP\_LYMPHOCYTE\_ACTIVATION\_INVOLVED\_IN\_IMMUNE\_RESPONSE |  | 159 | -0.21 | -0.99 | 0.486 | 0.723 | 1.000 | 4271 | tags=36%, list=33%, signal=54% |
| 1847 | GOBP\_REGULATION\_OF\_PEPTIDE\_TRANSPORT |  | 155 | -0.21 | -0.99 | 0.471 | 0.724 | 1.000 | 2765 | tags=23%, list=21%, signal=29% |
| 1848 | GOBP\_SKELETAL\_SYSTEM\_MORPHOGENESIS |  | 188 | -0.21 | -0.99 | 0.498 | 0.726 | 1.000 | 3046 | tags=27%, list=24%, signal=34% |
| 1849 | GOBP\_ORGANOPHOSPHATE\_ESTER\_TRANSPORT |  | 104 | -0.23 | -0.99 | 0.493 | 0.727 | 1.000 | 4049 | tags=38%, list=31%, signal=54% |
| 1850 | GOBP\_GLYCOSPHINGOLIPID\_BIOSYNTHETIC\_PROCESS |  | 28 | -0.30 | -0.99 | 0.482 | 0.726 | 1.000 | 1738 | tags=25%, list=13%, signal=29% |
| 1851 | GOBP\_MIDBRAIN\_DEVELOPMENT |  | 74 | -0.24 | -0.99 | 0.466 | 0.727 | 1.000 | 861 | tags=15%, list=7%, signal=16% |
| 1852 | GOBP\_REGULATION\_OF\_ENDOCYTOSIS |  | 173 | -0.21 | -0.99 | 0.493 | 0.727 | 1.000 | 1844 | tags=19%, list=14%, signal=22% |
| 1853 | GOBP\_NEGATIVE\_CHEMOTAXIS |  | 40 | -0.27 | -0.99 | 0.461 | 0.727 | 1.000 | 1776 | tags=23%, list=14%, signal=26% |
| 1854 | GOBP\_REGULATION\_OF\_MACROPHAGE\_DERIVED\_FOAM\_CELL\_DIFFERENTIATION |  | 31 | -0.29 | -0.99 | 0.480 | 0.727 | 1.000 | 3289 | tags=29%, list=25%, signal=39% |
| 1855 | GOBP\_MONOCARBOXYLIC\_ACID\_TRANSPORT |  | 88 | -0.23 | -0.99 | 0.490 | 0.727 | 1.000 | 3182 | tags=28%, list=25%, signal=37% |
| 1856 | GOBP\_NEGATIVE\_REGULATION\_OF\_HYDROLASE\_ACTIVITY |  | 275 | -0.20 | -0.99 | 0.476 | 0.728 | 1.000 | 3429 | tags=29%, list=27%, signal=39% |
| 1857 | GOBP\_REGULATION\_OF\_WATER\_LOSS\_VIA\_SKIN |  | 21 | -0.32 | -0.99 | 0.488 | 0.728 | 1.000 | 2103 | tags=24%, list=16%, signal=28% |
| 1858 | GOBP\_POSITIVE\_REGULATION\_OF\_CALCIUM\_ION\_TRANSPORT\_INTO\_CYTOSOL |  | 50 | -0.26 | -0.99 | 0.481 | 0.730 | 1.000 | 2982 | tags=30%, list=23%, signal=39% |
| 1859 | GOBP\_SECONDARY\_METABOLITE\_BIOSYNTHETIC\_PROCESS |  | 24 | -0.30 | -0.99 | 0.491 | 0.730 | 1.000 | 1937 | tags=25%, list=15%, signal=29% |
| 1860 | GOBP\_THYROID\_HORMONE\_METABOLIC\_PROCESS |  | 24 | -0.30 | -0.99 | 0.481 | 0.729 | 1.000 | 2589 | tags=29%, list=20%, signal=36% |
| 1861 | GOBP\_SPINDLE\_LOCALIZATION |  | 43 | -0.27 | -0.99 | 0.488 | 0.729 | 1.000 | 2312 | tags=23%, list=18%, signal=28% |
| 1862 | GOBP\_MRNA\_CLEAVAGE |  | 16 | -0.34 | -0.99 | 0.476 | 0.730 | 1.000 | 2715 | tags=38%, list=21%, signal=47% |
| 1863 | GOBP\_REGULATION\_OF\_HAIR\_CYCLE |  | 26 | -0.30 | -0.98 | 0.486 | 0.730 | 1.000 | 805 | tags=15%, list=6%, signal=16% |
| 1864 | GOBP\_POSITIVE\_REGULATION\_OF\_LEUKOCYTE\_CELL\_CELL\_ADHESION |  | 215 | -0.20 | -0.98 | 0.484 | 0.730 | 1.000 | 2848 | tags=23%, list=22%, signal=29% |
| 1865 | GOBP\_POSITIVE\_REGULATION\_OF\_NEUROBLAST\_PROLIFERATION |  | 22 | -0.31 | -0.98 | 0.494 | 0.730 | 1.000 | 1069 | tags=18%, list=8%, signal=20% |
| 1866 | GOBP\_ZYMOGEN\_ACTIVATION |  | 59 | -0.25 | -0.98 | 0.460 | 0.732 | 1.000 | 4492 | tags=42%, list=35%, signal=65% |
| 1867 | GOBP\_POSITIVE\_REGULATION\_OF\_AMYLOID\_BETA\_FORMATION |  | 16 | -0.34 | -0.98 | 0.474 | 0.733 | 1.000 | 1039 | tags=19%, list=8%, signal=20% |
| 1868 | GOBP\_NEGATIVE\_REGULATION\_OF\_TRANSMEMBRANE\_TRANSPORT |  | 91 | -0.23 | -0.98 | 0.502 | 0.733 | 1.000 | 1512 | tags=14%, list=12%, signal=16% |
| 1869 | GOBP\_TORC1\_SIGNALING |  | 33 | -0.29 | -0.98 | 0.462 | 0.733 | 1.000 | 2388 | tags=33%, list=19%, signal=41% |
| 1870 | GOBP\_POSITIVE\_REGULATION\_OF\_LEUKOCYTE\_ADHESION\_TO\_VASCULAR\_ENDOTHELIAL\_CELL |  | 22 | -0.31 | -0.98 | 0.485 | 0.733 | 1.000 | 442 | tags=14%, list=3%, signal=14% |
| 1871 | GOBP\_IRON\_ION\_HOMEOSTASIS |  | 66 | -0.25 | -0.98 | 0.474 | 0.734 | 1.000 | 4059 | tags=45%, list=31%, signal=66% |
| 1872 | GOBP\_POSTSYNAPSE\_ASSEMBLY |  | 23 | -0.32 | -0.98 | 0.493 | 0.734 | 1.000 | 1169 | tags=17%, list=9%, signal=19% |
| 1873 | GOBP\_VIRAL\_TRANSLATION |  | 17 | -0.33 | -0.98 | 0.493 | 0.735 | 1.000 | 3126 | tags=41%, list=24%, signal=54% |
| 1874 | GOBP\_LEARNING |  | 127 | -0.22 | -0.98 | 0.501 | 0.736 | 1.000 | 4226 | tags=34%, list=33%, signal=50% |
| 1875 | GOBP\_POSITIVE\_REGULATION\_OF\_AUTOPHAGY |  | 111 | -0.22 | -0.98 | 0.509 | 0.736 | 1.000 | 1949 | tags=22%, list=15%, signal=25% |
| 1876 | GOBP\_BEHAVIOR |  | 496 | -0.19 | -0.98 | 0.541 | 0.737 | 1.000 | 3193 | tags=25%, list=25%, signal=32% |
| 1877 | GOBP\_REGULATION\_OF\_APPETITE |  | 18 | -0.33 | -0.98 | 0.499 | 0.737 | 1.000 | 2176 | tags=28%, list=17%, signal=33% |
| 1878 | GOBP\_MEGAKARYOCYTE\_DEVELOPMENT |  | 15 | -0.35 | -0.98 | 0.493 | 0.737 | 1.000 | 1756 | tags=27%, list=14%, signal=31% |
| 1879 | GOBP\_NEGATIVE\_REGULATION\_OF\_AMINE\_TRANSPORT |  | 25 | -0.30 | -0.98 | 0.469 | 0.738 | 1.000 | 3111 | tags=36%, list=24%, signal=47% |
| 1880 | GOBP\_REGULATION\_OF\_ESTABLISHMENT\_OF\_PROTEIN\_LOCALIZATION\_TO\_MITOCHONDRION |  | 44 | -0.26 | -0.98 | 0.474 | 0.738 | 1.000 | 2861 | tags=32%, list=22%, signal=41% |
| 1881 | GOBP\_T\_CELL\_PROLIFERATION |  | 175 | -0.21 | -0.98 | 0.513 | 0.738 | 1.000 | 2831 | tags=25%, list=22%, signal=31% |
| 1882 | GOBP\_REGULATION\_OF\_RESPONSE\_TO\_BIOTIC\_STIMULUS |  | 284 | -0.20 | -0.98 | 0.502 | 0.739 | 1.000 | 3707 | tags=31%, list=29%, signal=42% |
| 1883 | GOBP\_REGULATION\_OF\_HORMONE\_LEVELS |  | 425 | -0.19 | -0.98 | 0.539 | 0.738 | 1.000 | 3970 | tags=33%, list=31%, signal=47% |
| 1884 | GOBP\_INFLAMMASOME\_COMPLEX\_ASSEMBLY |  | 17 | -0.33 | -0.98 | 0.481 | 0.738 | 1.000 | 114 | tags=12%, list=1%, signal=12% |
| 1885 | GOBP\_REGULATION\_OF\_PLASMINOGEN\_ACTIVATION |  | 15 | -0.34 | -0.98 | 0.492 | 0.738 | 1.000 | 4261 | tags=53%, list=33%, signal=80% |
| 1886 | GOBP\_POSITIVE\_REGULATION\_OF\_SIGNALING\_RECEPTOR\_ACTIVITY |  | 43 | -0.27 | -0.98 | 0.500 | 0.740 | 1.000 | 3111 | tags=35%, list=24%, signal=46% |
| 1887 | GOBP\_REGULATION\_OF\_INOSITOL\_PHOSPHATE\_BIOSYNTHETIC\_PROCESS |  | 16 | -0.34 | -0.98 | 0.480 | 0.740 | 1.000 | 1284 | tags=19%, list=10%, signal=21% |
| 1888 | GOBP\_CHLORIDE\_TRANSMEMBRANE\_TRANSPORT |  | 54 | -0.26 | -0.98 | 0.514 | 0.740 | 1.000 | 4185 | tags=39%, list=32%, signal=57% |
| 1889 | GOBP\_POSITIVE\_REGULATION\_OF\_PEPTIDYL\_LYSINE\_ACETYLATION |  | 35 | -0.28 | -0.98 | 0.501 | 0.740 | 1.000 | 4038 | tags=49%, list=31%, signal=71% |
| 1890 | GOBP\_ANDROGEN\_RECEPTOR\_SIGNALING\_PATHWAY |  | 42 | -0.26 | -0.98 | 0.480 | 0.739 | 1.000 | 3018 | tags=33%, list=23%, signal=43% |
| 1891 | GOBP\_REGULATION\_OF\_EXTRACELLULAR\_MATRIX\_ORGANIZATION |  | 42 | -0.27 | -0.98 | 0.489 | 0.741 | 1.000 | 4416 | tags=45%, list=34%, signal=69% |
| 1892 | GOBP\_POSITIVE\_REGULATION\_OF\_MAST\_CELL\_ACTIVATION |  | 20 | -0.32 | -0.98 | 0.496 | 0.741 | 1.000 | 2698 | tags=30%, list=21%, signal=38% |
| 1893 | GOBP\_REGULATION\_OF\_FATTY\_ACID\_TRANSPORT |  | 32 | -0.28 | -0.98 | 0.491 | 0.741 | 1.000 | 2197 | tags=28%, list=17%, signal=34% |
| 1894 | GOBP\_RESPONSE\_TO\_GLUCAGON |  | 20 | -0.32 | -0.98 | 0.503 | 0.742 | 1.000 | 3802 | tags=40%, list=29%, signal=57% |
| 1895 | GOBP\_NEGATIVE\_REGULATION\_OF\_CELL\_KILLING |  | 26 | -0.30 | -0.98 | 0.508 | 0.742 | 1.000 | 2225 | tags=23%, list=17%, signal=28% |
| 1896 | GOBP\_ENDOCARDIAL\_CUSHION\_MORPHOGENESIS |  | 36 | -0.28 | -0.98 | 0.475 | 0.742 | 1.000 | 1983 | tags=22%, list=15%, signal=26% |
| 1897 | GOBP\_CELLULAR\_RESPONSE\_TO\_XENOBIOTIC\_STIMULUS |  | 138 | -0.21 | -0.98 | 0.505 | 0.742 | 1.000 | 3303 | tags=32%, list=26%, signal=42% |
| 1898 | GOBP\_POSITIVE\_REGULATION\_OF\_BEHAVIOR |  | 21 | -0.31 | -0.98 | 0.515 | 0.744 | 1.000 | 557 | tags=14%, list=4%, signal=15% |
| 1899 | GOBP\_HYALURONAN\_METABOLIC\_PROCESS |  | 25 | -0.30 | -0.98 | 0.489 | 0.743 | 1.000 | 2482 | tags=28%, list=19%, signal=35% |
| 1900 | GOBP\_REGULATION\_OF\_DENDRITE\_MORPHOGENESIS |  | 57 | -0.25 | -0.98 | 0.479 | 0.743 | 1.000 | 3058 | tags=28%, list=24%, signal=37% |
| 1901 | GOBP\_PROTEIN\_ACTIVATION\_CASCADE |  | 17 | -0.33 | -0.97 | 0.494 | 0.745 | 1.000 | 337 | tags=12%, list=3%, signal=12% |
| 1902 | GOBP\_INSULIN\_SECRETION |  | 148 | -0.21 | -0.97 | 0.503 | 0.744 | 1.000 | 2493 | tags=22%, list=19%, signal=27% |
| 1903 | GOBP\_DETECTION\_OF\_TEMPERATURE\_STIMULUS\_INVOLVED\_IN\_SENSORY\_PERCEPTION |  | 18 | -0.33 | -0.97 | 0.476 | 0.747 | 1.000 | 1229 | tags=17%, list=10%, signal=18% |
| 1904 | GOBP\_RESPONSE\_TO\_XENOBIOTIC\_STIMULUS |  | 357 | -0.19 | -0.97 | 0.542 | 0.748 | 1.000 | 3031 | tags=25%, list=23%, signal=32% |
| 1905 | GOBP\_KETONE\_BIOSYNTHETIC\_PROCESS |  | 40 | -0.27 | -0.97 | 0.497 | 0.748 | 1.000 | 3152 | tags=35%, list=24%, signal=46% |
| 1906 | GOBP\_SEX\_DETERMINATION |  | 20 | -0.31 | -0.97 | 0.487 | 0.748 | 1.000 | 2594 | tags=25%, list=20%, signal=31% |
| 1907 | GOBP\_REGULATION\_OF\_CELL\_CYCLE\_G2\_M\_PHASE\_TRANSITION |  | 83 | -0.23 | -0.97 | 0.526 | 0.748 | 1.000 | 3123 | tags=27%, list=24%, signal=35% |
| 1908 | GOBP\_EXOCRINE\_SYSTEM\_DEVELOPMENT |  | 43 | -0.27 | -0.97 | 0.495 | 0.749 | 1.000 | 1554 | tags=19%, list=12%, signal=21% |
| 1909 | GOBP\_NEGATIVE\_REGULATION\_OF\_STRESS\_ACTIVATED\_PROTEIN\_KINASE\_SIGNALING\_CASCADE |  | 42 | -0.27 | -0.97 | 0.501 | 0.752 | 1.000 | 1249 | tags=17%, list=10%, signal=18% |
| 1910 | GOBP\_REGULATION\_OF\_HOMOTYPIC\_CELL\_CELL\_ADHESION |  | 31 | -0.28 | -0.97 | 0.497 | 0.753 | 1.000 | 3691 | tags=32%, list=29%, signal=45% |
| 1911 | GOBP\_NEGATIVE\_REGULATION\_OF\_PROTEIN\_ACETYLATION |  | 19 | -0.32 | -0.97 | 0.488 | 0.753 | 1.000 | 3955 | tags=53%, list=31%, signal=76% |
| 1912 | GOBP\_NEURON\_PROJECTION\_ARBORIZATION |  | 24 | -0.31 | -0.97 | 0.504 | 0.755 | 1.000 | 2184 | tags=25%, list=17%, signal=30% |
| 1913 | GOBP\_REGULATION\_OF\_TOLL\_LIKE\_RECEPTOR\_SIGNALING\_PATHWAY |  | 56 | -0.25 | -0.97 | 0.530 | 0.756 | 1.000 | 2202 | tags=25%, list=17%, signal=30% |
| 1914 | GOBP\_REGULATION\_OF\_T\_CELL\_APOPTOTIC\_PROCESS |  | 25 | -0.30 | -0.97 | 0.487 | 0.756 | 1.000 | 3669 | tags=40%, list=28%, signal=56% |
| 1915 | GOBP\_MYELOID\_DENDRITIC\_CELL\_DIFFERENTIATION |  | 18 | -0.33 | -0.97 | 0.497 | 0.756 | 1.000 | 4159 | tags=56%, list=32%, signal=82% |
| 1916 | GOBP\_NEGATIVE\_REGULATION\_OF\_VASCULAR\_PERMEABILITY |  | 17 | -0.33 | -0.97 | 0.488 | 0.756 | 1.000 | 4488 | tags=59%, list=35%, signal=90% |
| 1917 | GOBP\_REGULATION\_OF\_KETONE\_BIOSYNTHETIC\_PROCESS |  | 16 | -0.33 | -0.97 | 0.486 | 0.758 | 1.000 | 1983 | tags=31%, list=15%, signal=37% |
| 1918 | GOBP\_NEGATIVE\_REGULATION\_OF\_CARBOHYDRATE\_METABOLIC\_PROCESS |  | 41 | -0.26 | -0.97 | 0.524 | 0.758 | 1.000 | 2084 | tags=20%, list=16%, signal=23% |
| 1919 | GOBP\_REGULATION\_OF\_MEMBRANE\_PERMEABILITY |  | 57 | -0.25 | -0.97 | 0.523 | 0.758 | 1.000 | 3636 | tags=35%, list=28%, signal=49% |
| 1920 | GOBP\_POSITIVE\_REGULATION\_OF\_INTRACELLULAR\_TRANSPORT |  | 176 | -0.20 | -0.97 | 0.544 | 0.759 | 1.000 | 2929 | tags=26%, list=23%, signal=33% |
| 1921 | GOBP\_POSITIVE\_REGULATION\_OF\_SMOOTHENED\_SIGNALING\_PATHWAY |  | 25 | -0.30 | -0.97 | 0.489 | 0.759 | 1.000 | 2791 | tags=36%, list=22%, signal=46% |
| 1922 | GOBP\_POSITIVE\_REGULATION\_OF\_ION\_TRANSPORT |  | 232 | -0.20 | -0.97 | 0.553 | 0.758 | 1.000 | 3118 | tags=28%, list=24%, signal=36% |
| 1923 | GOBP\_CELLULAR\_CARBOHYDRATE\_METABOLIC\_PROCESS |  | 241 | -0.20 | -0.97 | 0.539 | 0.758 | 1.000 | 2084 | tags=18%, list=16%, signal=21% |
| 1924 | GOBP\_NEGATIVE\_REGULATION\_OF\_ESTABLISHMENT\_OF\_PROTEIN\_LOCALIZATION |  | 93 | -0.23 | -0.97 | 0.525 | 0.758 | 1.000 | 3879 | tags=35%, list=30%, signal=50% |
| 1925 | GOBP\_GLYCOPROTEIN\_CATABOLIC\_PROCESS |  | 19 | -0.32 | -0.97 | 0.483 | 0.758 | 1.000 | 3617 | tags=32%, list=28%, signal=44% |
| 1926 | GOBP\_DIENCEPHALON\_DEVELOPMENT |  | 64 | -0.24 | -0.97 | 0.528 | 0.759 | 1.000 | 2791 | tags=27%, list=22%, signal=34% |
| 1927 | GOBP\_POSITIVE\_REGULATION\_OF\_HORMONE\_SECRETION |  | 109 | -0.22 | -0.97 | 0.512 | 0.759 | 1.000 | 3867 | tags=36%, list=30%, signal=51% |
| 1928 | GOBP\_AMYLOID\_BETA\_CLEARANCE |  | 36 | -0.27 | -0.97 | 0.508 | 0.759 | 1.000 | 893 | tags=14%, list=7%, signal=15% |
| 1929 | GOBP\_FATTY\_ACID\_TRANSMEMBRANE\_TRANSPORT |  | 17 | -0.33 | -0.97 | 0.503 | 0.759 | 1.000 | 4250 | tags=53%, list=33%, signal=79% |
| 1930 | GOBP\_POSTSYNAPTIC\_SIGNAL\_TRANSDUCTION |  | 34 | -0.27 | -0.96 | 0.501 | 0.760 | 1.000 | 4868 | tags=50%, list=38%, signal=80% |
| 1931 | GOBP\_POSITIVE\_REGULATION\_OF\_CELL\_MATRIX\_ADHESION |  | 51 | -0.25 | -0.96 | 0.535 | 0.760 | 1.000 | 1981 | tags=22%, list=15%, signal=25% |
| 1932 | GOBP\_WALKING\_BEHAVIOR |  | 29 | -0.29 | -0.96 | 0.489 | 0.760 | 1.000 | 2670 | tags=38%, list=21%, signal=48% |
| 1933 | GOBP\_NEGATIVE\_REGULATION\_OF\_TYPE\_I\_INTERFERON\_MEDIATED\_SIGNALING\_PATHWAY |  | 17 | -0.32 | -0.96 | 0.482 | 0.761 | 1.000 | 2041 | tags=29%, list=16%, signal=35% |
| 1934 | GOBP\_REGULATION\_OF\_EXTRINSIC\_APOPTOTIC\_SIGNALING\_PATHWAY\_IN\_ABSENCE\_OF\_LIGAND |  | 39 | -0.27 | -0.96 | 0.497 | 0.761 | 1.000 | 2834 | tags=31%, list=22%, signal=39% |
| 1935 | GOBP\_NEGATIVE\_REGULATION\_OF\_KINASE\_ACTIVITY |  | 190 | -0.20 | -0.96 | 0.550 | 0.761 | 1.000 | 2990 | tags=26%, list=23%, signal=34% |
| 1936 | GOBP\_DNA\_PACKAGING |  | 190 | -0.20 | -0.96 | 0.548 | 0.761 | 1.000 | 1833 | tags=15%, list=14%, signal=18% |
| 1937 | GOBP\_POSITIVE\_REGULATION\_OF\_MULTICELLULAR\_ORGANISM\_GROWTH |  | 27 | -0.29 | -0.96 | 0.540 | 0.760 | 1.000 | 3369 | tags=30%, list=26%, signal=40% |
| 1938 | GOBP\_NEGATIVE\_REGULATION\_OF\_MITOTIC\_CELL\_CYCLE\_PHASE\_TRANSITION |  | 141 | -0.21 | -0.96 | 0.518 | 0.761 | 1.000 | 1124 | tags=11%, list=9%, signal=12% |
| 1939 | GOBP\_TRANSCRIPTION\_INITIATION\_FROM\_RNA\_POLYMERASE\_II\_PROMOTER |  | 71 | -0.24 | -0.96 | 0.537 | 0.761 | 1.000 | 2398 | tags=21%, list=19%, signal=26% |
| 1940 | GOBP\_DNA\_MODIFICATION |  | 87 | -0.23 | -0.96 | 0.524 | 0.765 | 1.000 | 2352 | tags=23%, list=18%, signal=28% |
| 1941 | GOBP\_COGNITION |  | 255 | -0.19 | -0.96 | 0.560 | 0.765 | 1.000 | 2581 | tags=20%, list=20%, signal=25% |
| 1942 | GOBP\_GLANDULAR\_EPITHELIAL\_CELL\_DEVELOPMENT |  | 25 | -0.30 | -0.96 | 0.497 | 0.766 | 1.000 | 2881 | tags=36%, list=22%, signal=46% |
| 1943 | GOBP\_NCRNA\_TRANSCRIPTION |  | 53 | -0.25 | -0.96 | 0.524 | 0.766 | 1.000 | 4408 | tags=43%, list=34%, signal=66% |
| 1944 | GOBP\_INSULIN\_SECRETION\_INVOLVED\_IN\_CELLULAR\_RESPONSE\_TO\_GLUCOSE\_STIMULUS |  | 48 | -0.25 | -0.96 | 0.490 | 0.766 | 1.000 | 2493 | tags=27%, list=19%, signal=33% |
| 1945 | GOBP\_POSITIVE\_REGULATION\_OF\_TOLL\_LIKE\_RECEPTOR\_SIGNALING\_PATHWAY |  | 22 | -0.31 | -0.96 | 0.514 | 0.767 | 1.000 | 1242 | tags=27%, list=10%, signal=30% |
| 1946 | GOBP\_REGULATION\_OF\_INSULIN\_SECRETION |  | 122 | -0.21 | -0.96 | 0.536 | 0.767 | 1.000 | 2493 | tags=21%, list=19%, signal=26% |
| 1947 | GOBP\_HOMOPHILIC\_CELL\_ADHESION\_VIA\_PLASMA\_MEMBRANE\_ADHESION\_MOLECULES |  | 105 | -0.22 | -0.96 | 0.517 | 0.768 | 1.000 | 3006 | tags=26%, list=23%, signal=33% |
| 1948 | GOBP\_NEGATIVE\_REGULATION\_OF\_CHEMOTAXIS |  | 57 | -0.24 | -0.96 | 0.532 | 0.769 | 1.000 | 1776 | tags=21%, list=14%, signal=24% |
| 1949 | GOBP\_NEGATIVE\_REGULATION\_OF\_NEURON\_DIFFERENTIATION |  | 57 | -0.25 | -0.96 | 0.535 | 0.769 | 1.000 | 1925 | tags=19%, list=15%, signal=23% |
| 1950 | GOBP\_REGULATION\_OF\_NATURAL\_KILLER\_CELL\_MEDIATED\_IMMUNITY |  | 40 | -0.26 | -0.96 | 0.521 | 0.769 | 1.000 | 2826 | tags=25%, list=22%, signal=32% |
| 1951 | GOBP\_NEGATIVE\_REGULATION\_OF\_ION\_TRANSPORT |  | 111 | -0.22 | -0.96 | 0.540 | 0.769 | 1.000 | 2922 | tags=23%, list=23%, signal=29% |
| 1952 | GOBP\_ENTEROENDOCRINE\_CELL\_DIFFERENTIATION |  | 26 | -0.29 | -0.96 | 0.514 | 0.770 | 1.000 | 2881 | tags=35%, list=22%, signal=44% |
| 1953 | GOBP\_REGULATION\_OF\_CHONDROCYTE\_DIFFERENTIATION |  | 41 | -0.26 | -0.96 | 0.504 | 0.770 | 1.000 | 3589 | tags=34%, list=28%, signal=47% |
| 1954 | GOBP\_ANIMAL\_ORGAN\_FORMATION |  | 54 | -0.25 | -0.96 | 0.548 | 0.770 | 1.000 | 402 | tags=9%, list=3%, signal=10% |
| 1955 | GOBP\_SYNAPSE\_ORGANIZATION |  | 327 | -0.19 | -0.96 | 0.606 | 0.770 | 1.000 | 3015 | tags=23%, list=23%, signal=29% |
| 1956 | GOBP\_REGULATION\_OF\_CYTOSKELETON\_ORGANIZATION |  | 440 | -0.18 | -0.96 | 0.607 | 0.770 | 1.000 | 3240 | tags=26%, list=25%, signal=34% |
| 1957 | GOBP\_DNA\_RECOMBINATION |  | 240 | -0.19 | -0.96 | 0.576 | 0.770 | 1.000 | 3290 | tags=26%, list=26%, signal=34% |
| 1958 | GOBP\_RNA\_INTERFERENCE |  | 15 | -0.34 | -0.96 | 0.504 | 0.771 | 1.000 | 2275 | tags=33%, list=18%, signal=40% |
| 1959 | GOBP\_POSITIVE\_REGULATION\_OF\_CHONDROCYTE\_DIFFERENTIATION |  | 16 | -0.33 | -0.96 | 0.524 | 0.771 | 1.000 | 2881 | tags=38%, list=22%, signal=48% |
| 1960 | GOBP\_REGULATION\_OF\_MITOTIC\_CELL\_CYCLE\_PHASE\_TRANSITION |  | 236 | -0.20 | -0.96 | 0.596 | 0.771 | 1.000 | 1124 | tags=11%, list=9%, signal=11% |
| 1961 | GOBP\_LIPID\_LOCALIZATION |  | 351 | -0.19 | -0.96 | 0.584 | 0.771 | 1.000 | 3095 | tags=25%, list=24%, signal=32% |
| 1962 | GOBP\_REGULATION\_OF\_TISSUE\_REMODELING |  | 69 | -0.23 | -0.96 | 0.541 | 0.771 | 1.000 | 1672 | tags=17%, list=13%, signal=20% |
| 1963 | GOBP\_T\_CELL\_MEDIATED\_IMMUNITY |  | 103 | -0.22 | -0.96 | 0.552 | 0.770 | 1.000 | 2101 | tags=19%, list=16%, signal=23% |
| 1964 | GOBP\_POSITIVE\_REGULATION\_OF\_AMYLOID\_PRECURSOR\_PROTEIN\_CATABOLIC\_PROCESS |  | 21 | -0.31 | -0.96 | 0.538 | 0.771 | 1.000 | 1381 | tags=19%, list=11%, signal=21% |
| 1965 | GOBP\_HISTONE\_H2A\_ACETYLATION |  | 19 | -0.31 | -0.96 | 0.525 | 0.771 | 1.000 | 2081 | tags=26%, list=16%, signal=31% |
| 1966 | GOBP\_POSITIVE\_REGULATION\_OF\_HISTONE\_METHYLATION |  | 41 | -0.26 | -0.96 | 0.504 | 0.771 | 1.000 | 1229 | tags=15%, list=10%, signal=16% |
| 1967 | GOBP\_REGULATION\_OF\_ALPHA\_BETA\_T\_CELL\_ACTIVATION |  | 87 | -0.22 | -0.96 | 0.544 | 0.770 | 1.000 | 2819 | tags=25%, list=22%, signal=32% |
| 1968 | GOBP\_NEGATIVE\_REGULATION\_OF\_LEUKOCYTE\_MIGRATION |  | 42 | -0.26 | -0.96 | 0.523 | 0.772 | 1.000 | 3237 | tags=31%, list=25%, signal=41% |
| 1969 | GOBP\_REGULATION\_OF\_DOUBLE\_STRAND\_BREAK\_REPAIR\_VIA\_HOMOLOGOUS\_RECOMBINATION |  | 50 | -0.24 | -0.96 | 0.552 | 0.771 | 1.000 | 1345 | tags=16%, list=10%, signal=18% |
| 1970 | GOBP\_MALE\_GENITALIA\_DEVELOPMENT |  | 20 | -0.32 | -0.96 | 0.504 | 0.771 | 1.000 | 3183 | tags=30%, list=25%, signal=40% |
| 1971 | GOBP\_POSITIVE\_REGULATION\_OF\_ADAPTIVE\_IMMUNE\_RESPONSE |  | 97 | -0.22 | -0.95 | 0.562 | 0.772 | 1.000 | 797 | tags=11%, list=6%, signal=12% |
| 1972 | GOBP\_PROTEIN\_LOCALIZATION\_TO\_CELL\_JUNCTION |  | 80 | -0.23 | -0.95 | 0.548 | 0.772 | 1.000 | 3553 | tags=33%, list=28%, signal=45% |
| 1973 | GOBP\_ESTROUS\_CYCLE |  | 15 | -0.33 | -0.95 | 0.518 | 0.772 | 1.000 | 2078 | tags=27%, list=16%, signal=32% |
| 1974 | GOBP\_CLATHRIN\_COAT\_ASSEMBLY |  | 16 | -0.33 | -0.95 | 0.521 | 0.776 | 1.000 | 3997 | tags=44%, list=31%, signal=63% |
| 1975 | GOBP\_NEGATIVE\_REGULATION\_OF\_CHEMOKINE\_PRODUCTION |  | 22 | -0.30 | -0.95 | 0.527 | 0.777 | 1.000 | 442 | tags=14%, list=3%, signal=14% |
| 1976 | GOBP\_POSITIVE\_REGULATION\_OF\_NEURON\_DIFFERENTIATION |  | 73 | -0.23 | -0.95 | 0.540 | 0.777 | 1.000 | 4390 | tags=44%, list=34%, signal=66% |
| 1977 | GOBP\_NEGATIVE\_REGULATION\_OF\_PEPTIDASE\_ACTIVITY |  | 191 | -0.20 | -0.95 | 0.578 | 0.777 | 1.000 | 3065 | tags=26%, list=24%, signal=33% |
| 1978 | GOBP\_REGULATION\_OF\_TRANS\_SYNAPTIC\_SIGNALING |  | 342 | -0.19 | -0.95 | 0.616 | 0.778 | 1.000 | 4352 | tags=38%, list=34%, signal=55% |
| 1979 | GOBP\_NEGATIVE\_REGULATION\_OF\_PHOSPHORYLATION |  | 307 | -0.19 | -0.95 | 0.594 | 0.779 | 1.000 | 3465 | tags=29%, list=27%, signal=38% |
| 1980 | GOBP\_PROTEIN\_LOCALIZATION\_TO\_MICROTUBULE\_ORGANIZING\_CENTER |  | 32 | -0.27 | -0.95 | 0.513 | 0.779 | 1.000 | 3891 | tags=38%, list=30%, signal=54% |
| 1981 | GOBP\_ADHESION\_OF\_SYMBIONT\_TO\_HOST |  | 16 | -0.32 | -0.95 | 0.522 | 0.779 | 1.000 | 3162 | tags=38%, list=25%, signal=50% |
| 1982 | GOBP\_REGULATION\_OF\_CHROMOSOME\_ORGANIZATION |  | 177 | -0.20 | -0.95 | 0.557 | 0.779 | 1.000 | 2462 | tags=19%, list=19%, signal=23% |
| 1983 | GOBP\_REGULATION\_OF\_ACTIN\_FILAMENT\_ORGANIZATION |  | 218 | -0.19 | -0.95 | 0.593 | 0.779 | 1.000 | 3160 | tags=27%, list=24%, signal=35% |
| 1984 | GOBP\_RESPONSE\_TO\_VIRUS |  | 320 | -0.19 | -0.95 | 0.584 | 0.779 | 1.000 | 3753 | tags=31%, list=29%, signal=42% |
| 1985 | GOBP\_APPENDAGE\_MORPHOGENESIS |  | 120 | -0.21 | -0.95 | 0.569 | 0.779 | 1.000 | 3636 | tags=33%, list=28%, signal=46% |
| 1986 | GOBP\_EMBRYONIC\_APPENDAGE\_MORPHOGENESIS |  | 102 | -0.21 | -0.95 | 0.571 | 0.780 | 1.000 | 2345 | tags=23%, list=18%, signal=27% |
| 1987 | GOBP\_NEGATIVE\_REGULATION\_OF\_MUSCLE\_CONTRACTION |  | 22 | -0.30 | -0.95 | 0.534 | 0.781 | 1.000 | 4684 | tags=64%, list=36%, signal=100% |
| 1988 | GOBP\_HAIR\_CELL\_DIFFERENTIATION |  | 32 | -0.27 | -0.95 | 0.523 | 0.781 | 1.000 | 2616 | tags=25%, list=20%, signal=31% |
| 1989 | GOBP\_MOTOR\_NEURON\_AXON\_GUIDANCE |  | 24 | -0.29 | -0.95 | 0.523 | 0.781 | 1.000 | 2371 | tags=25%, list=18%, signal=31% |
| 1990 | GOBP\_REGULATION\_OF\_ALPHA\_BETA\_T\_CELL\_DIFFERENTIATION |  | 57 | -0.24 | -0.95 | 0.550 | 0.781 | 1.000 | 2819 | tags=25%, list=22%, signal=31% |
| 1991 | GOBP\_NEGATIVE\_REGULATION\_OF\_DNA\_METABOLIC\_PROCESS |  | 92 | -0.22 | -0.95 | 0.570 | 0.781 | 1.000 | 1904 | tags=17%, list=15%, signal=20% |
| 1992 | GOBP\_LONG\_TERM\_SYNAPTIC\_DEPRESSION |  | 26 | -0.29 | -0.95 | 0.530 | 0.781 | 1.000 | 4351 | tags=42%, list=34%, signal=64% |
| 1993 | GOBP\_REGULATION\_OF\_SUBSTRATE\_ADHESION\_DEPENDENT\_CELL\_SPREADING |  | 54 | -0.24 | -0.95 | 0.555 | 0.781 | 1.000 | 579 | tags=11%, list=4%, signal=12% |
| 1994 | GOBP\_POSITIVE\_REGULATION\_OF\_PROTEIN\_LOCALIZATION\_TO\_MEMBRANE |  | 84 | -0.22 | -0.95 | 0.547 | 0.781 | 1.000 | 2929 | tags=27%, list=23%, signal=35% |
| 1995 | GOBP\_REGULATION\_OF\_B\_CELL\_DIFFERENTIATION |  | 24 | -0.29 | -0.95 | 0.550 | 0.781 | 1.000 | 1665 | tags=21%, list=13%, signal=24% |
| 1996 | GOBP\_REGULATION\_OF\_PRODUCTION\_OF\_MOLECULAR\_MEDIATOR\_OF\_IMMUNE\_RESPONSE |  | 148 | -0.20 | -0.95 | 0.578 | 0.781 | 1.000 | 2101 | tags=19%, list=16%, signal=22% |
| 1997 | GOBP\_PLATELET\_AGGREGATION |  | 62 | -0.24 | -0.95 | 0.554 | 0.782 | 1.000 | 4172 | tags=35%, list=32%, signal=52% |
| 1998 | GOBP\_POSITIVE\_REGULATION\_OF\_ORGANELLE\_ORGANIZATION |  | 410 | -0.18 | -0.95 | 0.640 | 0.786 | 1.000 | 1843 | tags=15%, list=14%, signal=17% |
| 1999 | GOBP\_EMBRYONIC\_DIGIT\_MORPHOGENESIS |  | 47 | -0.24 | -0.94 | 0.538 | 0.788 | 1.000 | 2021 | tags=23%, list=16%, signal=28% |
| 2000 | GOBP\_ADRENAL\_GLAND\_DEVELOPMENT |  | 26 | -0.28 | -0.94 | 0.527 | 0.788 | 1.000 | 715 | tags=12%, list=6%, signal=12% |
| 2001 | GOBP\_REGULATION\_OF\_ERAD\_PATHWAY |  | 16 | -0.33 | -0.94 | 0.511 | 0.789 | 1.000 | 2 | tags=6%, list=0%, signal=6% |
| 2002 | GOBP\_ENDOCYTOSIS |  | 490 | -0.18 | -0.94 | 0.660 | 0.790 | 1.000 | 2138 | tags=18%, list=17%, signal=21% |
| 2003 | GOBP\_REGULATION\_OF\_SYNAPTIC\_TRANSMISSION\_GABAERGIC |  | 29 | -0.28 | -0.94 | 0.563 | 0.791 | 1.000 | 2358 | tags=24%, list=18%, signal=29% |
| 2004 | GOBP\_TYPE\_B\_PANCREATIC\_CELL\_DEVELOPMENT |  | 20 | -0.31 | -0.94 | 0.545 | 0.791 | 1.000 | 2881 | tags=35%, list=22%, signal=45% |
| 2005 | GOBP\_REGULATION\_OF\_ESTABLISHMENT\_OF\_PROTEIN\_LOCALIZATION |  | 438 | -0.18 | -0.94 | 0.660 | 0.791 | 1.000 | 3970 | tags=33%, list=31%, signal=46% |
| 2006 | GOBP\_REGULATION\_OF\_ACTIN\_FILAMENT\_BUNDLE\_ASSEMBLY |  | 92 | -0.22 | -0.94 | 0.572 | 0.791 | 1.000 | 1803 | tags=17%, list=14%, signal=20% |
| 2007 | GOBP\_NEGATIVE\_REGULATION\_OF\_NIK\_NF\_KAPPAB\_SIGNALING |  | 22 | -0.31 | -0.94 | 0.541 | 0.792 | 1.000 | 2253 | tags=23%, list=17%, signal=27% |
| 2008 | GOBP\_POSITIVE\_REGULATION\_OF\_MAP\_KINASE\_ACTIVITY |  | 107 | -0.21 | -0.94 | 0.609 | 0.792 | 1.000 | 2112 | tags=21%, list=16%, signal=24% |
| 2009 | GOBP\_ICOSANOID\_METABOLIC\_PROCESS |  | 90 | -0.22 | -0.94 | 0.586 | 0.793 | 1.000 | 2274 | tags=22%, list=18%, signal=27% |
| 2010 | GOBP\_PHOSPHOLIPID\_TRANSPORT |  | 76 | -0.22 | -0.94 | 0.583 | 0.793 | 1.000 | 4048 | tags=37%, list=31%, signal=53% |
| 2011 | GOBP\_MATING |  | 35 | -0.27 | -0.94 | 0.539 | 0.793 | 1.000 | 3832 | tags=37%, list=30%, signal=53% |
| 2012 | GOBP\_NATURAL\_KILLER\_CELL\_MEDIATED\_IMMUNITY |  | 62 | -0.24 | -0.94 | 0.579 | 0.793 | 1.000 | 2225 | tags=19%, list=17%, signal=23% |
| 2013 | GOBP\_NEGATIVE\_REGULATION\_OF\_DNA\_REPAIR |  | 23 | -0.30 | -0.94 | 0.547 | 0.793 | 1.000 | 1713 | tags=22%, list=13%, signal=25% |
| 2014 | GOBP\_REGULATION\_OF\_PROTEIN\_POLYUBIQUITINATION |  | 25 | -0.28 | -0.94 | 0.536 | 0.793 | 1.000 | 916 | tags=12%, list=7%, signal=13% |
| 2015 | GOBP\_EXPORT\_ACROSS\_PLASMA\_MEMBRANE |  | 50 | -0.25 | -0.94 | 0.560 | 0.793 | 1.000 | 3028 | tags=32%, list=23%, signal=42% |
| 2016 | GOBP\_SYNAPSE\_MATURATION |  | 22 | -0.29 | -0.94 | 0.541 | 0.794 | 1.000 | 2514 | tags=32%, list=19%, signal=39% |
| 2017 | GOBP\_REGULATION\_OF\_ANIMAL\_ORGAN\_FORMATION |  | 24 | -0.29 | -0.94 | 0.546 | 0.794 | 1.000 | 129 | tags=8%, list=1%, signal=8% |
| 2018 | GOBP\_ANTERIOR\_POSTERIOR\_PATTERN\_SPECIFICATION |  | 170 | -0.20 | -0.94 | 0.586 | 0.796 | 1.000 | 3108 | tags=25%, list=24%, signal=32% |
| 2019 | GOBP\_REGULATION\_OF\_HISTONE\_H3\_K4\_METHYLATION |  | 33 | -0.27 | -0.94 | 0.559 | 0.798 | 1.000 | 626 | tags=12%, list=5%, signal=13% |
| 2020 | GOBP\_CELL\_CYCLE\_G2\_M\_PHASE\_TRANSITION |  | 123 | -0.21 | -0.94 | 0.563 | 0.797 | 1.000 | 1142 | tags=13%, list=9%, signal=14% |
| 2021 | GOBP\_EMBRYONIC\_EYE\_MORPHOGENESIS |  | 33 | -0.27 | -0.94 | 0.570 | 0.798 | 1.000 | 4202 | tags=30%, list=33%, signal=45% |
| 2022 | GOBP\_POSITIVE\_REGULATION\_OF\_PATTERN\_RECOGNITION\_RECEPTOR\_SIGNALING\_PATHWAY |  | 34 | -0.26 | -0.94 | 0.548 | 0.798 | 1.000 | 1242 | tags=21%, list=10%, signal=23% |
| 2023 | GOBP\_REGULATION\_OF\_RHO\_PROTEIN\_SIGNAL\_TRANSDUCTION |  | 71 | -0.23 | -0.94 | 0.575 | 0.797 | 1.000 | 2378 | tags=23%, list=18%, signal=27% |
| 2024 | GOBP\_NEUROEPITHELIAL\_CELL\_DIFFERENTIATION |  | 21 | -0.30 | -0.94 | 0.515 | 0.798 | 1.000 | 571 | tags=14%, list=4%, signal=15% |
| 2025 | GOBP\_TELOMERE\_CAPPING |  | 37 | -0.27 | -0.94 | 0.546 | 0.799 | 1.000 | 934 | tags=14%, list=7%, signal=15% |
| 2026 | GOBP\_AMELOGENESIS |  | 16 | -0.32 | -0.94 | 0.559 | 0.799 | 1.000 | 2131 | tags=31%, list=17%, signal=37% |
| 2027 | GOBP\_RESPONSE\_TO\_TYPE\_I\_INTERFERON |  | 56 | -0.24 | -0.94 | 0.565 | 0.799 | 1.000 | 3707 | tags=39%, list=29%, signal=55% |
| 2028 | GOBP\_CELLULAR\_HORMONE\_METABOLIC\_PROCESS |  | 115 | -0.21 | -0.94 | 0.596 | 0.800 | 1.000 | 2003 | tags=19%, list=16%, signal=22% |
| 2029 | GOBP\_POSITIVE\_REGULATION\_OF\_MACROAUTOPHAGY |  | 59 | -0.24 | -0.94 | 0.585 | 0.801 | 1.000 | 1949 | tags=22%, list=15%, signal=26% |
| 2030 | GOBP\_POTASSIUM\_ION\_HOMEOSTASIS |  | 29 | -0.28 | -0.94 | 0.558 | 0.801 | 1.000 | 4009 | tags=48%, list=31%, signal=70% |
| 2031 | GOBP\_BONE\_MORPHOGENESIS |  | 83 | -0.22 | -0.94 | 0.609 | 0.801 | 1.000 | 3046 | tags=30%, list=24%, signal=39% |
| 2032 | GOBP\_ACROSOME\_REACTION |  | 25 | -0.29 | -0.94 | 0.536 | 0.801 | 1.000 | 2504 | tags=24%, list=19%, signal=30% |
| 2033 | GOBP\_REGULATION\_OF\_MONONUCLEAR\_CELL\_MIGRATION |  | 100 | -0.21 | -0.94 | 0.589 | 0.801 | 1.000 | 3435 | tags=30%, list=27%, signal=41% |
| 2034 | GOBP\_POSITIVE\_REGULATION\_OF\_BMP\_SIGNALING\_PATHWAY |  | 32 | -0.27 | -0.93 | 0.543 | 0.800 | 1.000 | 4219 | tags=50%, list=33%, signal=74% |
| 2035 | GOBP\_PROTEIN\_DEPOLYMERIZATION |  | 98 | -0.22 | -0.93 | 0.584 | 0.801 | 1.000 | 2886 | tags=23%, list=22%, signal=30% |
| 2036 | GOBP\_CELLULAR\_DEFENSE\_RESPONSE |  | 52 | -0.24 | -0.93 | 0.566 | 0.801 | 1.000 | 3824 | tags=37%, list=30%, signal=52% |
| 2037 | GOBP\_INTERMEDIATE\_FILAMENT\_BASED\_PROCESS |  | 59 | -0.24 | -0.93 | 0.569 | 0.801 | 1.000 | 4450 | tags=36%, list=34%, signal=54% |
| 2038 | GOBP\_POSITIVE\_REGULATION\_OF\_REGULATED\_SECRETORY\_PATHWAY |  | 43 | -0.25 | -0.93 | 0.556 | 0.801 | 1.000 | 4271 | tags=37%, list=33%, signal=55% |
| 2039 | GOBP\_PROTEIN\_CONTAINING\_COMPLEX\_DISASSEMBLY |  | 197 | -0.19 | -0.93 | 0.631 | 0.803 | 1.000 | 2936 | tags=26%, list=23%, signal=33% |
| 2040 | GOBP\_SOMATIC\_DIVERSIFICATION\_OF\_IMMUNE\_RECEPTORS |  | 72 | -0.23 | -0.93 | 0.587 | 0.803 | 1.000 | 732 | tags=10%, list=6%, signal=10% |
| 2041 | GOBP\_OLEFINIC\_COMPOUND\_METABOLIC\_PROCESS |  | 126 | -0.21 | -0.93 | 0.620 | 0.803 | 1.000 | 3996 | tags=36%, list=31%, signal=51% |
| 2042 | GOBP\_PURINERGIC\_NUCLEOTIDE\_RECEPTOR\_SIGNALING\_PATHWAY |  | 29 | -0.27 | -0.93 | 0.560 | 0.804 | 1.000 | 4216 | tags=41%, list=33%, signal=61% |
| 2043 | GOBP\_NEGATIVE\_REGULATION\_OF\_T\_CELL\_APOPTOTIC\_PROCESS |  | 17 | -0.31 | -0.93 | 0.566 | 0.804 | 1.000 | 3669 | tags=41%, list=28%, signal=57% |
| 2044 | GOBP\_G\_PROTEIN\_COUPLED\_ACETYLCHOLINE\_RECEPTOR\_SIGNALING\_PATHWAY |  | 17 | -0.31 | -0.93 | 0.563 | 0.805 | 1.000 | 2177 | tags=24%, list=17%, signal=28% |
| 2045 | GOBP\_CELLULAR\_RESPONSE\_TO\_RETINOIC\_ACID |  | 60 | -0.24 | -0.93 | 0.577 | 0.804 | 1.000 | 1880 | tags=22%, list=15%, signal=25% |
| 2046 | GOBP\_LUNG\_CELL\_DIFFERENTIATION |  | 21 | -0.30 | -0.93 | 0.558 | 0.805 | 1.000 | 1552 | tags=19%, list=12%, signal=22% |
| 2047 | GOBP\_REGULATION\_OF\_MYOBLAST\_DIFFERENTIATION |  | 40 | -0.26 | -0.93 | 0.582 | 0.805 | 1.000 | 3576 | tags=35%, list=28%, signal=48% |
| 2048 | GOBP\_NEGATIVE\_REGULATION\_OF\_LIPID\_TRANSPORT |  | 25 | -0.29 | -0.93 | 0.552 | 0.804 | 1.000 | 1655 | tags=24%, list=13%, signal=27% |
| 2049 | GOBP\_STRIATED\_MUSCLE\_CELL\_DIFFERENTIATION |  | 216 | -0.19 | -0.93 | 0.644 | 0.805 | 1.000 | 3118 | tags=25%, list=24%, signal=32% |
| 2050 | GOBP\_EMBRYONIC\_ORGAN\_MORPHOGENESIS |  | 229 | -0.19 | -0.93 | 0.646 | 0.806 | 1.000 | 3371 | tags=28%, list=26%, signal=37% |
| 2051 | GOBP\_MITOTIC\_G2\_M\_TRANSITION\_CHECKPOINT |  | 38 | -0.26 | -0.93 | 0.599 | 0.810 | 1.000 | 1444 | tags=16%, list=11%, signal=18% |
| 2052 | GOBP\_REGULATION\_OF\_LYMPHOCYTE\_ACTIVATION |  | 401 | -0.18 | -0.93 | 0.688 | 0.809 | 1.000 | 2848 | tags=22%, list=22%, signal=28% |
| 2053 | GOBP\_REGULATION\_OF\_PROTEIN\_STABILITY |  | 255 | -0.19 | -0.93 | 0.660 | 0.809 | 1.000 | 3270 | tags=27%, list=25%, signal=36% |
| 2054 | GOBP\_INOSITOL\_LIPID\_MEDIATED\_SIGNALING |  | 155 | -0.20 | -0.93 | 0.610 | 0.810 | 1.000 | 2076 | tags=17%, list=16%, signal=21% |
| 2055 | GOBP\_ACTIN\_POLYMERIZATION\_OR\_DEPOLYMERIZATION |  | 155 | -0.20 | -0.93 | 0.630 | 0.809 | 1.000 | 3380 | tags=29%, list=26%, signal=39% |
| 2056 | GOBP\_PATTERN\_SPECIFICATION\_PROCESS |  | 346 | -0.18 | -0.93 | 0.691 | 0.809 | 1.000 | 3108 | tags=24%, list=24%, signal=31% |
| 2057 | GOBP\_POSITIVE\_REGULATION\_OF\_PROTEIN\_TYROSINE\_KINASE\_ACTIVITY |  | 46 | -0.25 | -0.93 | 0.580 | 0.809 | 1.000 | 3249 | tags=30%, list=25%, signal=41% |
| 2058 | GOBP\_CELLULAR\_COMPONENT\_DISASSEMBLY |  | 388 | -0.18 | -0.93 | 0.670 | 0.810 | 1.000 | 3258 | tags=26%, list=25%, signal=34% |
| 2059 | GOBP\_PROTEIN\_LIPID\_COMPLEX\_ASSEMBLY |  | 24 | -0.29 | -0.93 | 0.554 | 0.809 | 1.000 | 4046 | tags=50%, list=31%, signal=73% |
| 2060 | GOBP\_SKELETAL\_SYSTEM\_DEVELOPMENT |  | 438 | -0.18 | -0.93 | 0.713 | 0.810 | 1.000 | 3205 | tags=26%, list=25%, signal=33% |
| 2061 | GOBP\_REGULATION\_OF\_PROTEIN\_BINDING |  | 169 | -0.20 | -0.93 | 0.650 | 0.810 | 1.000 | 2291 | tags=21%, list=18%, signal=25% |
| 2062 | GOBP\_ANTIGEN\_PROCESSING\_AND\_PRESENTATION\_OF\_EXOGENOUS\_ANTIGEN |  | 44 | -0.25 | -0.93 | 0.582 | 0.810 | 1.000 | 555 | tags=11%, list=4%, signal=12% |
| 2063 | GOBP\_REPLACEMENT\_OSSIFICATION |  | 24 | -0.29 | -0.92 | 0.573 | 0.816 | 1.000 | 2881 | tags=29%, list=22%, signal=37% |
| 2064 | GOBP\_REGULATION\_OF\_PATTERN\_RECOGNITION\_RECEPTOR\_SIGNALING\_PATHWAY |  | 82 | -0.22 | -0.92 | 0.623 | 0.817 | 1.000 | 1242 | tags=15%, list=10%, signal=16% |
| 2065 | GOBP\_OLIGODENDROCYTE\_DIFFERENTIATION |  | 77 | -0.23 | -0.92 | 0.604 | 0.817 | 1.000 | 1066 | tags=13%, list=8%, signal=14% |
| 2066 | GOBP\_INTEGRIN\_ACTIVATION |  | 23 | -0.29 | -0.92 | 0.581 | 0.817 | 1.000 | 3771 | tags=43%, list=29%, signal=61% |
| 2067 | GOBP\_NEGATIVE\_REGULATION\_OF\_CYTOSKELETON\_ORGANIZATION |  | 131 | -0.20 | -0.92 | 0.641 | 0.817 | 1.000 | 3239 | tags=28%, list=25%, signal=37% |
| 2068 | GOBP\_L\_GLUTAMATE\_IMPORT\_ACROSS\_PLASMA\_MEMBRANE |  | 16 | -0.32 | -0.92 | 0.551 | 0.818 | 1.000 | 2276 | tags=25%, list=18%, signal=30% |
| 2069 | GOBP\_REGULATION\_OF\_DNA\_BIOSYNTHETIC\_PROCESS |  | 108 | -0.21 | -0.92 | 0.593 | 0.818 | 1.000 | 798 | tags=11%, list=6%, signal=12% |
| 2070 | GOBP\_REGULATION\_OF\_LEUKOCYTE\_CHEMOTAXIS |  | 112 | -0.21 | -0.92 | 0.616 | 0.818 | 1.000 | 2338 | tags=21%, list=18%, signal=25% |
| 2071 | GOBP\_CARDIAC\_CHAMBER\_DEVELOPMENT |  | 146 | -0.20 | -0.92 | 0.642 | 0.818 | 1.000 | 2182 | tags=17%, list=17%, signal=20% |
| 2072 | GOBP\_REGULATION\_OF\_ADAPTIVE\_IMMUNE\_RESPONSE |  | 159 | -0.20 | -0.92 | 0.641 | 0.818 | 1.000 | 2290 | tags=19%, list=18%, signal=23% |
| 2073 | GOBP\_LIPOPOLYSACCHARIDE\_MEDIATED\_SIGNALING\_PATHWAY |  | 56 | -0.24 | -0.92 | 0.607 | 0.818 | 1.000 | 978 | tags=13%, list=8%, signal=13% |
| 2074 | GOBP\_CELL\_DIVISION |  | 487 | -0.18 | -0.92 | 0.714 | 0.820 | 1.000 | 2473 | tags=19%, list=19%, signal=23% |
| 2075 | GOBP\_REGULATION\_OF\_DENDRITE\_DEVELOPMENT |  | 84 | -0.22 | -0.92 | 0.606 | 0.820 | 1.000 | 3184 | tags=29%, list=25%, signal=38% |
| 2076 | GOBP\_REGULATION\_OF\_PROTEIN\_TARGETING |  | 68 | -0.23 | -0.92 | 0.606 | 0.821 | 1.000 | 1962 | tags=22%, list=15%, signal=26% |
| 2077 | GOBP\_CELL\_CYCLE\_DNA\_REPLICATION |  | 40 | -0.26 | -0.92 | 0.576 | 0.821 | 1.000 | 1124 | tags=18%, list=9%, signal=19% |
| 2078 | GOBP\_REGULATION\_OF\_CARDIAC\_CONDUCTION |  | 22 | -0.29 | -0.92 | 0.579 | 0.824 | 1.000 | 3027 | tags=32%, list=23%, signal=42% |
| 2079 | GOBP\_SARCOMERE\_ORGANIZATION |  | 38 | -0.25 | -0.92 | 0.592 | 0.824 | 1.000 | 353 | tags=11%, list=3%, signal=11% |
| 2080 | GOBP\_MORPHOGENESIS\_OF\_EMBRYONIC\_EPITHELIUM |  | 118 | -0.21 | -0.92 | 0.633 | 0.827 | 1.000 | 4247 | tags=38%, list=33%, signal=56% |
| 2081 | GOBP\_POSITIVE\_REGULATION\_OF\_CELLULAR\_EXTRAVASATION |  | 16 | -0.31 | -0.92 | 0.589 | 0.829 | 1.000 | 3526 | tags=44%, list=27%, signal=60% |
| 2082 | GOBP\_MUSCLE\_CELL\_DEVELOPMENT |  | 144 | -0.20 | -0.92 | 0.676 | 0.829 | 1.000 | 3118 | tags=26%, list=24%, signal=34% |
| 2083 | GOBP\_ANTIMICROBIAL\_HUMORAL\_IMMUNE\_RESPONSE\_MEDIATED\_BY\_ANTIMICROBIAL\_PEPTIDE |  | 65 | -0.23 | -0.92 | 0.608 | 0.831 | 1.000 | 1686 | tags=14%, list=13%, signal=16% |
| 2084 | GOBP\_CELLULAR\_RESPONSE\_TO\_REACTIVE\_NITROGEN\_SPECIES |  | 17 | -0.31 | -0.92 | 0.592 | 0.831 | 1.000 | 1979 | tags=24%, list=15%, signal=28% |
| 2085 | GOBP\_POSITIVE\_REGULATION\_OF\_CYTOSKELETON\_ORGANIZATION |  | 167 | -0.20 | -0.92 | 0.640 | 0.831 | 1.000 | 4339 | tags=38%, list=34%, signal=56% |
| 2086 | GOBP\_LOW\_DENSITY\_LIPOPROTEIN\_PARTICLE\_CLEARANCE |  | 24 | -0.28 | -0.92 | 0.576 | 0.831 | 1.000 | 2561 | tags=33%, list=20%, signal=42% |
| 2087 | GOBP\_REGULATION\_OF\_PROTEIN\_CONTAINING\_COMPLEX\_ASSEMBLY |  | 324 | -0.18 | -0.92 | 0.715 | 0.831 | 1.000 | 3821 | tags=30%, list=30%, signal=42% |
| 2088 | GOBP\_POSITIVE\_REGULATION\_OF\_EXTRINSIC\_APOPTOTIC\_SIGNALING\_PATHWAY |  | 46 | -0.24 | -0.92 | 0.577 | 0.831 | 1.000 | 2818 | tags=30%, list=22%, signal=39% |
| 2089 | GOBP\_SENSORY\_PERCEPTION\_OF\_TASTE |  | 38 | -0.25 | -0.92 | 0.603 | 0.831 | 1.000 | 5548 | tags=53%, list=43%, signal=92% |
| 2090 | GOBP\_MEMBRANE\_PROTEIN\_PROTEOLYSIS |  | 48 | -0.24 | -0.91 | 0.605 | 0.832 | 1.000 | 2112 | tags=25%, list=16%, signal=30% |
| 2091 | GOBP\_CELL\_JUNCTION\_MAINTENANCE |  | 31 | -0.27 | -0.91 | 0.599 | 0.832 | 1.000 | 4475 | tags=48%, list=35%, signal=74% |
| 2092 | GOBP\_NEGATIVE\_REGULATION\_OF\_SMOOTH\_MUSCLE\_CELL\_MIGRATION |  | 22 | -0.29 | -0.91 | 0.590 | 0.833 | 1.000 | 2618 | tags=27%, list=20%, signal=34% |
| 2093 | GOBP\_HEART\_PROCESS |  | 222 | -0.19 | -0.91 | 0.704 | 0.833 | 1.000 | 3082 | tags=23%, list=24%, signal=30% |
| 2094 | GOBP\_MICROTUBULE\_POLYMERIZATION\_OR\_DEPOLYMERIZATION |  | 103 | -0.21 | -0.91 | 0.625 | 0.834 | 1.000 | 2650 | tags=23%, list=21%, signal=29% |
| 2095 | GOBP\_MITOTIC\_CHROMOSOME\_CONDENSATION |  | 16 | -0.31 | -0.91 | 0.603 | 0.835 | 1.000 | 2899 | tags=31%, list=22%, signal=40% |
| 2096 | GOBP\_REGULATION\_OF\_MAST\_CELL\_ACTIVATION\_INVOLVED\_IN\_IMMUNE\_RESPONSE |  | 32 | -0.26 | -0.91 | 0.588 | 0.835 | 1.000 | 2000 | tags=22%, list=16%, signal=26% |
| 2097 | GOBP\_TRANSCRIPTION\_ELONGATION\_FROM\_RNA\_POLYMERASE\_II\_PROMOTER |  | 54 | -0.23 | -0.91 | 0.624 | 0.835 | 1.000 | 4233 | tags=46%, list=33%, signal=69% |
| 2098 | GOBP\_GLIAL\_CELL\_DEVELOPMENT |  | 101 | -0.21 | -0.91 | 0.667 | 0.835 | 1.000 | 1066 | tags=12%, list=8%, signal=13% |
| 2099 | GOBP\_NCRNA\_3\_END\_PROCESSING |  | 30 | -0.27 | -0.91 | 0.585 | 0.836 | 1.000 | 2401 | tags=27%, list=19%, signal=33% |
| 2100 | GOBP\_REGULATION\_OF\_GLYCOLYTIC\_PROCESS |  | 43 | -0.25 | -0.91 | 0.584 | 0.835 | 1.000 | 2084 | tags=23%, list=16%, signal=28% |
| 2101 | GOBP\_REGULATION\_OF\_BONE\_REMODELING |  | 45 | -0.25 | -0.91 | 0.618 | 0.835 | 1.000 | 1672 | tags=20%, list=13%, signal=23% |
| 2102 | GOBP\_MEIOTIC\_CELL\_CYCLE |  | 178 | -0.19 | -0.91 | 0.677 | 0.835 | 1.000 | 2696 | tags=20%, list=21%, signal=25% |
| 2103 | GOBP\_MICROTUBULE\_ANCHORING |  | 18 | -0.31 | -0.91 | 0.603 | 0.834 | 1.000 | 2283 | tags=28%, list=18%, signal=34% |
| 2104 | GOBP\_POSITIVE\_REGULATION\_OF\_ALCOHOL\_BIOSYNTHETIC\_PROCESS |  | 25 | -0.28 | -0.91 | 0.598 | 0.834 | 1.000 | 2977 | tags=28%, list=23%, signal=36% |
| 2105 | GOBP\_SMAD\_PROTEIN\_SIGNAL\_TRANSDUCTION |  | 76 | -0.22 | -0.91 | 0.616 | 0.835 | 1.000 | 3362 | tags=26%, list=26%, signal=35% |
| 2106 | GOBP\_ENSHEATHMENT\_OF\_NEURONS |  | 121 | -0.20 | -0.91 | 0.660 | 0.835 | 1.000 | 4214 | tags=37%, list=33%, signal=55% |
| 2107 | GOBP\_NITRIC\_OXIDE\_MEDIATED\_SIGNAL\_TRANSDUCTION |  | 24 | -0.28 | -0.91 | 0.576 | 0.836 | 1.000 | 3893 | tags=42%, list=30%, signal=60% |
| 2108 | GOBP\_NEGATIVE\_REGULATION\_OF\_PEPTIDYL\_SERINE\_PHOSPHORYLATION |  | 26 | -0.28 | -0.91 | 0.623 | 0.837 | 1.000 | 4178 | tags=38%, list=32%, signal=57% |
| 2109 | GOBP\_MYELIN\_ASSEMBLY |  | 21 | -0.30 | -0.91 | 0.577 | 0.837 | 1.000 | 687 | tags=14%, list=5%, signal=15% |
| 2110 | GOBP\_SNRNA\_PROCESSING |  | 18 | -0.31 | -0.91 | 0.601 | 0.838 | 1.000 | 2955 | tags=33%, list=23%, signal=43% |
| 2111 | GOBP\_POLYSACCHARIDE\_BIOSYNTHETIC\_PROCESS |  | 59 | -0.23 | -0.91 | 0.625 | 0.838 | 1.000 | 2072 | tags=20%, list=16%, signal=24% |
| 2112 | GOBP\_PHOSPHATIDIC\_ACID\_METABOLIC\_PROCESS |  | 29 | -0.27 | -0.91 | 0.597 | 0.837 | 1.000 | 4694 | tags=52%, list=36%, signal=81% |
| 2113 | GOBP\_INORGANIC\_ANION\_TRANSPORT |  | 151 | -0.20 | -0.91 | 0.664 | 0.837 | 1.000 | 4443 | tags=38%, list=34%, signal=58% |
| 2114 | GOBP\_POSITIVE\_REGULATION\_OF\_SUPRAMOLECULAR\_FIBER\_ORGANIZATION |  | 152 | -0.20 | -0.91 | 0.693 | 0.837 | 1.000 | 4395 | tags=39%, list=34%, signal=59% |
| 2115 | GOBP\_EPITHELIAL\_TUBE\_BRANCHING\_INVOLVED\_IN\_LUNG\_MORPHOGENESIS |  | 23 | -0.28 | -0.91 | 0.592 | 0.840 | 1.000 | 129 | tags=9%, list=1%, signal=9% |
| 2116 | GOBP\_SCHWANN\_CELL\_DEVELOPMENT |  | 29 | -0.27 | -0.91 | 0.588 | 0.839 | 1.000 | 687 | tags=10%, list=5%, signal=11% |
| 2117 | GOBP\_REGULATION\_OF\_ACTIN\_CYTOSKELETON\_REORGANIZATION |  | 34 | -0.26 | -0.91 | 0.607 | 0.839 | 1.000 | 1522 | tags=18%, list=12%, signal=20% |
| 2118 | GOBP\_CRANIAL\_SKELETAL\_SYSTEM\_DEVELOPMENT |  | 58 | -0.23 | -0.91 | 0.655 | 0.840 | 1.000 | 3046 | tags=28%, list=24%, signal=36% |
| 2119 | GOBP\_CELLULAR\_RESPONSE\_TO\_IONIZING\_RADIATION |  | 64 | -0.23 | -0.91 | 0.642 | 0.840 | 1.000 | 1713 | tags=19%, list=13%, signal=22% |
| 2120 | GOBP\_PROXIMAL\_DISTAL\_PATTERN\_FORMATION |  | 23 | -0.28 | -0.91 | 0.613 | 0.840 | 1.000 | 1880 | tags=26%, list=15%, signal=30% |
| 2121 | GOBP\_PROTEIN\_KINASE\_B\_SIGNALING |  | 169 | -0.19 | -0.91 | 0.676 | 0.841 | 1.000 | 1792 | tags=15%, list=14%, signal=18% |
| 2122 | GOBP\_EPITHELIAL\_TO\_MESENCHYMAL\_TRANSITION\_INVOLVED\_IN\_ENDOCARDIAL\_CUSHION\_FORMATION |  | 19 | -0.30 | -0.91 | 0.577 | 0.841 | 1.000 | 848 | tags=16%, list=7%, signal=17% |
| 2123 | GOBP\_REGULATION\_OF\_T\_CELL\_MIGRATION |  | 36 | -0.25 | -0.91 | 0.609 | 0.841 | 1.000 | 3001 | tags=33%, list=23%, signal=43% |
| 2124 | GOBP\_REGULATION\_OF\_LEUKOCYTE\_APOPTOTIC\_PROCESS |  | 68 | -0.22 | -0.91 | 0.647 | 0.840 | 1.000 | 3669 | tags=32%, list=28%, signal=45% |
| 2125 | GOBP\_ANTIGEN\_PROCESSING\_AND\_PRESENTATION\_OF\_ENDOGENOUS\_ANTIGEN |  | 24 | -0.28 | -0.91 | 0.596 | 0.842 | 1.000 | 1286 | tags=21%, list=10%, signal=23% |
| 2126 | GOBP\_PROTEIN\_LOCALIZATION\_TO\_CHROMOSOME\_CENTROMERIC\_REGION |  | 20 | -0.30 | -0.91 | 0.563 | 0.841 | 1.000 | 1406 | tags=15%, list=11%, signal=17% |
| 2127 | GOBP\_MEMBRANE\_DEPOLARIZATION\_DURING\_CARDIAC\_MUSCLE\_CELL\_ACTION\_POTENTIAL |  | 18 | -0.30 | -0.91 | 0.593 | 0.841 | 1.000 | 4684 | tags=44%, list=36%, signal=70% |
| 2128 | GOBP\_REGULATION\_OF\_LEUKOCYTE\_PROLIFERATION |  | 214 | -0.19 | -0.91 | 0.707 | 0.842 | 1.000 | 2513 | tags=20%, list=19%, signal=24% |
| 2129 | GOBP\_INTERLEUKIN\_8\_PRODUCTION |  | 75 | -0.22 | -0.91 | 0.669 | 0.842 | 1.000 | 2161 | tags=23%, list=17%, signal=27% |
| 2130 | GOBP\_NEGATIVE\_REGULATION\_OF\_VIRAL\_PROCESS |  | 80 | -0.22 | -0.90 | 0.657 | 0.842 | 1.000 | 3165 | tags=29%, list=25%, signal=38% |
| 2131 | GOBP\_REGULATION\_OF\_VESICLE\_FUSION |  | 18 | -0.30 | -0.90 | 0.595 | 0.842 | 1.000 | 2078 | tags=22%, list=16%, signal=26% |
| 2132 | GOBP\_ORGANIC\_HYDROXY\_COMPOUND\_TRANSPORT |  | 220 | -0.19 | -0.90 | 0.711 | 0.842 | 1.000 | 4065 | tags=34%, list=32%, signal=49% |
| 2133 | GOBP\_REGULATION\_OF\_LIPID\_TRANSPORT |  | 115 | -0.20 | -0.90 | 0.660 | 0.843 | 1.000 | 4065 | tags=37%, list=32%, signal=54% |
| 2134 | GOBP\_TOR\_SIGNALING |  | 90 | -0.21 | -0.90 | 0.660 | 0.842 | 1.000 | 2902 | tags=29%, list=22%, signal=37% |
| 2135 | GOBP\_BLASTOCYST\_DEVELOPMENT |  | 82 | -0.22 | -0.90 | 0.657 | 0.842 | 1.000 | 3201 | tags=28%, list=25%, signal=37% |
| 2136 | GOBP\_ASSOCIATIVE\_LEARNING |  | 70 | -0.23 | -0.90 | 0.633 | 0.844 | 1.000 | 4816 | tags=44%, list=37%, signal=70% |
| 2137 | GOBP\_CENTRAL\_NERVOUS\_SYSTEM\_NEURON\_DIFFERENTIATION |  | 128 | -0.20 | -0.90 | 0.682 | 0.845 | 1.000 | 2842 | tags=24%, list=22%, signal=31% |
| 2138 | GOBP\_MUSCLE\_HYPERTROPHY\_IN\_RESPONSE\_TO\_STRESS |  | 20 | -0.29 | -0.90 | 0.621 | 0.845 | 1.000 | 1966 | tags=20%, list=15%, signal=24% |
| 2139 | GOBP\_CELL\_PROJECTION\_ASSEMBLY |  | 413 | -0.17 | -0.90 | 0.785 | 0.846 | 1.000 | 3312 | tags=27%, list=26%, signal=36% |
| 2140 | GOBP\_REGULATION\_OF\_PROTEIN\_DEPHOSPHORYLATION |  | 76 | -0.22 | -0.90 | 0.642 | 0.847 | 1.000 | 1745 | tags=20%, list=14%, signal=23% |
| 2141 | GOBP\_NEGATIVE\_REGULATION\_OF\_BIOMINERALIZATION |  | 26 | -0.27 | -0.90 | 0.612 | 0.847 | 1.000 | 2254 | tags=27%, list=17%, signal=33% |
| 2142 | GOBP\_EXCITATORY\_SYNAPSE\_ASSEMBLY |  | 18 | -0.30 | -0.90 | 0.592 | 0.849 | 1.000 | 1169 | tags=17%, list=9%, signal=18% |
| 2143 | GOBP\_REGULATION\_OF\_DEPHOSPHORYLATION |  | 108 | -0.21 | -0.90 | 0.677 | 0.848 | 1.000 | 2956 | tags=25%, list=23%, signal=32% |
| 2144 | GOBP\_DETECTION\_OF\_LIGHT\_STIMULUS |  | 49 | -0.24 | -0.90 | 0.634 | 0.848 | 1.000 | 2600 | tags=27%, list=20%, signal=33% |
| 2145 | GOBP\_LIPID\_STORAGE |  | 70 | -0.22 | -0.90 | 0.654 | 0.848 | 1.000 | 3032 | tags=27%, list=24%, signal=35% |
| 2146 | GOBP\_POSITIVE\_REGULATION\_OF\_HISTONE\_H3\_K4\_METHYLATION |  | 24 | -0.28 | -0.90 | 0.606 | 0.848 | 1.000 | 462 | tags=13%, list=4%, signal=13% |
| 2147 | GOBP\_POSITIVE\_REGULATION\_OF\_ESTABLISHMENT\_OF\_PROTEIN\_LOCALIZATION |  | 276 | -0.18 | -0.90 | 0.744 | 0.848 | 1.000 | 2929 | tags=25%, list=23%, signal=31% |
| 2148 | GOBP\_POSITIVE\_REGULATION\_OF\_CYTOSOLIC\_CALCIUM\_ION\_CONCENTRATION\_INVOLVED\_IN\_PHOSPHOLIPASE\_C\_ACTIVATING\_G\_PROTEIN\_COUPLED\_SIGNALING\_PATHWAY |  | 26 | -0.27 | -0.90 | 0.606 | 0.848 | 1.000 | 2378 | tags=27%, list=18%, signal=33% |
| 2149 | GOBP\_PHOSPHATIDYLCHOLINE\_METABOLIC\_PROCESS |  | 61 | -0.23 | -0.90 | 0.639 | 0.848 | 1.000 | 4788 | tags=43%, list=37%, signal=67% |
| 2150 | GOBP\_MICROTUBULE\_BUNDLE\_FORMATION |  | 67 | -0.22 | -0.90 | 0.659 | 0.848 | 1.000 | 3008 | tags=25%, list=23%, signal=33% |
| 2151 | GOBP\_REGULATION\_OF\_PH |  | 72 | -0.22 | -0.90 | 0.654 | 0.848 | 1.000 | 2060 | tags=21%, list=16%, signal=25% |
| 2152 | GOBP\_ALCOHOL\_CATABOLIC\_PROCESS |  | 48 | -0.24 | -0.90 | 0.633 | 0.849 | 1.000 | 2611 | tags=21%, list=20%, signal=26% |
| 2153 | GOBP\_NEURON\_FATE\_COMMITMENT |  | 46 | -0.24 | -0.90 | 0.614 | 0.849 | 1.000 | 4226 | tags=41%, list=33%, signal=61% |
| 2154 | GOBP\_PHOSPHATIDYLETHANOLAMINE\_METABOLIC\_PROCESS |  | 20 | -0.29 | -0.90 | 0.597 | 0.850 | 1.000 | 2750 | tags=30%, list=21%, signal=38% |
| 2155 | GOBP\_CHAPERONE\_MEDIATED\_PROTEIN\_COMPLEX\_ASSEMBLY |  | 20 | -0.29 | -0.90 | 0.614 | 0.852 | 1.000 | 3113 | tags=40%, list=24%, signal=53% |
| 2156 | GOBP\_CHROMOSOME\_CONDENSATION |  | 40 | -0.24 | -0.90 | 0.641 | 0.853 | 1.000 | 1501 | tags=15%, list=12%, signal=17% |
| 2157 | GOBP\_NEGATIVE\_REGULATION\_OF\_REPRODUCTIVE\_PROCESS |  | 48 | -0.24 | -0.90 | 0.622 | 0.853 | 1.000 | 2594 | tags=25%, list=20%, signal=31% |
| 2158 | GOBP\_NEUROTRANSMITTER\_RECEPTOR\_TRANSPORT |  | 17 | -0.31 | -0.90 | 0.606 | 0.853 | 1.000 | 2406 | tags=29%, list=19%, signal=36% |
| 2159 | GOBP\_POSITIVE\_REGULATION\_OF\_PEPTIDYL\_TYROSINE\_PHOSPHORYLATION |  | 163 | -0.19 | -0.90 | 0.694 | 0.854 | 1.000 | 2124 | tags=18%, list=16%, signal=22% |
| 2160 | GOBP\_ESTROGEN\_METABOLIC\_PROCESS |  | 31 | -0.27 | -0.90 | 0.592 | 0.854 | 1.000 | 2003 | tags=23%, list=16%, signal=27% |
| 2161 | GOBP\_ARP2\_3\_COMPLEX\_MEDIATED\_ACTIN\_NUCLEATION |  | 32 | -0.26 | -0.90 | 0.607 | 0.854 | 1.000 | 2008 | tags=28%, list=16%, signal=33% |
| 2162 | GOBP\_REGULATION\_OF\_ALCOHOL\_BIOSYNTHETIC\_PROCESS |  | 48 | -0.24 | -0.90 | 0.634 | 0.854 | 1.000 | 1983 | tags=19%, list=15%, signal=22% |
| 2163 | GOBP\_POSITIVE\_REGULATION\_OF\_RECEPTOR\_SIGNALING\_PATHWAY\_VIA\_STAT |  | 41 | -0.25 | -0.89 | 0.637 | 0.854 | 1.000 | 1287 | tags=15%, list=10%, signal=16% |
| 2164 | GOBP\_RESPONSE\_TO\_ETHANOL |  | 113 | -0.20 | -0.89 | 0.698 | 0.854 | 1.000 | 1931 | tags=19%, list=15%, signal=22% |
| 2165 | GOBP\_RECEPTOR\_CATABOLIC\_PROCESS |  | 22 | -0.29 | -0.89 | 0.638 | 0.855 | 1.000 | 1430 | tags=18%, list=11%, signal=20% |
| 2166 | GOBP\_PATTERN\_RECOGNITION\_RECEPTOR\_SIGNALING\_PATHWAY |  | 143 | -0.19 | -0.89 | 0.727 | 0.856 | 1.000 | 2028 | tags=17%, list=16%, signal=21% |
| 2167 | GOBP\_NEGATIVE\_REGULATION\_OF\_TRANSCRIPTION\_REGULATORY\_REGION\_DNA\_BINDING |  | 17 | -0.31 | -0.89 | 0.588 | 0.859 | 1.000 | 2457 | tags=35%, list=19%, signal=44% |
| 2168 | GOBP\_INTERLEUKIN\_13\_PRODUCTION |  | 18 | -0.29 | -0.89 | 0.606 | 0.859 | 1.000 | 3700 | tags=33%, list=29%, signal=47% |
| 2169 | GOBP\_INNER\_EAR\_RECEPTOR\_CELL\_DEVELOPMENT |  | 26 | -0.27 | -0.89 | 0.613 | 0.859 | 1.000 | 2702 | tags=27%, list=21%, signal=34% |
| 2170 | GOBP\_REGULATION\_OF\_DOUBLE\_STRAND\_BREAK\_REPAIR |  | 80 | -0.21 | -0.89 | 0.679 | 0.859 | 1.000 | 1345 | tags=14%, list=10%, signal=15% |
| 2171 | GOBP\_NOREPINEPHRINE\_TRANSPORT |  | 21 | -0.29 | -0.89 | 0.623 | 0.859 | 1.000 | 4430 | tags=43%, list=34%, signal=65% |
| 2172 | GOBP\_REGULATION\_OF\_TRANSPORTER\_ACTIVITY |  | 208 | -0.19 | -0.89 | 0.743 | 0.859 | 1.000 | 2323 | tags=20%, list=18%, signal=24% |
| 2173 | GOBP\_OSTEOCLAST\_DEVELOPMENT |  | 15 | -0.31 | -0.89 | 0.601 | 0.859 | 1.000 | 4021 | tags=47%, list=31%, signal=68% |
| 2174 | GOBP\_REGULATION\_OF\_SYNAPTIC\_PLASTICITY |  | 151 | -0.19 | -0.89 | 0.708 | 0.860 | 1.000 | 4351 | tags=40%, list=34%, signal=59% |
| 2175 | GOBP\_BONE\_REMODELING |  | 81 | -0.21 | -0.89 | 0.669 | 0.860 | 1.000 | 2848 | tags=25%, list=22%, signal=31% |
| 2176 | GOBP\_RESPONSE\_TO\_VITAMIN |  | 81 | -0.21 | -0.89 | 0.721 | 0.861 | 1.000 | 2493 | tags=23%, list=19%, signal=29% |
| 2177 | GOBP\_ORGANELLE\_LOCALIZATION |  | 437 | -0.17 | -0.89 | 0.809 | 0.861 | 1.000 | 2820 | tags=22%, list=22%, signal=27% |
| 2178 | GOBP\_POSITIVE\_REGULATION\_OF\_ORGANIC\_ACID\_TRANSPORT |  | 41 | -0.24 | -0.89 | 0.639 | 0.862 | 1.000 | 2459 | tags=24%, list=19%, signal=30% |
| 2179 | GOBP\_NEGATIVE\_REGULATION\_OF\_FIBROBLAST\_PROLIFERATION |  | 23 | -0.28 | -0.89 | 0.611 | 0.861 | 1.000 | 1059 | tags=17%, list=8%, signal=19% |
| 2180 | GOBP\_POSITIVE\_REGULATION\_OF\_SECRETION |  | 268 | -0.18 | -0.89 | 0.782 | 0.861 | 1.000 | 3867 | tags=31%, list=30%, signal=43% |
| 2181 | GOBP\_RESPONSE\_TO\_STEROL |  | 27 | -0.27 | -0.89 | 0.648 | 0.861 | 1.000 | 2791 | tags=33%, list=22%, signal=42% |
| 2182 | GOBP\_METENCEPHALON\_DEVELOPMENT |  | 95 | -0.20 | -0.89 | 0.694 | 0.863 | 1.000 | 3103 | tags=28%, list=24%, signal=37% |
| 2183 | GOBP\_MYELOID\_LEUKOCYTE\_ACTIVATION |  | 203 | -0.18 | -0.89 | 0.771 | 0.864 | 1.000 | 4214 | tags=37%, list=33%, signal=54% |
| 2184 | GOBP\_REGULATION\_OF\_MYOBLAST\_FUSION |  | 18 | -0.30 | -0.89 | 0.627 | 0.864 | 1.000 | 2338 | tags=33%, list=18%, signal=41% |
| 2185 | GOBP\_REGULATION\_OF\_PROTEIN\_SECRETION |  | 209 | -0.18 | -0.89 | 0.757 | 0.864 | 1.000 | 2941 | tags=22%, list=23%, signal=29% |
| 2186 | GOBP\_ACTIVIN\_RECEPTOR\_SIGNALING\_PATHWAY |  | 37 | -0.25 | -0.89 | 0.638 | 0.864 | 1.000 | 1891 | tags=22%, list=15%, signal=25% |
| 2187 | GOBP\_PURINE\_CONTAINING\_COMPOUND\_TRANSMEMBRANE\_TRANSPORT |  | 19 | -0.29 | -0.89 | 0.642 | 0.865 | 1.000 | 2971 | tags=32%, list=23%, signal=41% |
| 2188 | GOBP\_REGULATION\_OF\_PHOSPHATIDYLINOSITOL\_3\_KINASE\_SIGNALING |  | 97 | -0.21 | -0.89 | 0.703 | 0.865 | 1.000 | 1601 | tags=15%, list=12%, signal=18% |
| 2189 | GOBP\_CELLULAR\_RESPONSE\_TO\_FATTY\_ACID |  | 31 | -0.26 | -0.89 | 0.649 | 0.865 | 1.000 | 1626 | tags=19%, list=13%, signal=22% |
| 2190 | GOBP\_REGULATION\_OF\_HETEROTYPIC\_CELL\_CELL\_ADHESION |  | 24 | -0.27 | -0.89 | 0.652 | 0.866 | 1.000 | 4340 | tags=50%, list=34%, signal=75% |
| 2191 | GOBP\_PROTEIN\_CONTAINING\_COMPLEX\_LOCALIZATION |  | 122 | -0.20 | -0.89 | 0.713 | 0.866 | 1.000 | 2665 | tags=22%, list=21%, signal=28% |
| 2192 | GOBP\_ANTEROGRADE\_AXONAL\_TRANSPORT |  | 36 | -0.25 | -0.89 | 0.627 | 0.866 | 1.000 | 2150 | tags=22%, list=17%, signal=27% |
| 2193 | GOBP\_REGULATION\_OF\_PHOSPHOLIPID\_METABOLIC\_PROCESS |  | 32 | -0.26 | -0.89 | 0.644 | 0.866 | 1.000 | 2473 | tags=25%, list=19%, signal=31% |
| 2194 | GOBP\_GLYCEROLIPID\_METABOLIC\_PROCESS |  | 300 | -0.18 | -0.88 | 0.802 | 0.868 | 1.000 | 2750 | tags=22%, list=21%, signal=27% |
| 2195 | GOBP\_CILIUM\_OR\_FLAGELLUM\_DEPENDENT\_CELL\_MOTILITY |  | 75 | -0.22 | -0.88 | 0.675 | 0.868 | 1.000 | 3097 | tags=28%, list=24%, signal=37% |
| 2196 | GOBP\_HISTONE\_H4\_ACETYLATION |  | 64 | -0.22 | -0.88 | 0.677 | 0.868 | 1.000 | 1546 | tags=17%, list=12%, signal=19% |
| 2197 | GOBP\_CHLORIDE\_TRANSPORT |  | 90 | -0.21 | -0.88 | 0.704 | 0.868 | 1.000 | 4185 | tags=34%, list=32%, signal=51% |
| 2198 | GOBP\_VESICLE\_LOCALIZATION |  | 159 | -0.19 | -0.88 | 0.738 | 0.868 | 1.000 | 2960 | tags=24%, list=23%, signal=31% |
| 2199 | GOBP\_MEGAKARYOCYTE\_DIFFERENTIATION |  | 46 | -0.23 | -0.88 | 0.659 | 0.869 | 1.000 | 2578 | tags=24%, list=20%, signal=30% |
| 2200 | GOBP\_PROTEIN\_PEPTIDYL\_PROLYL\_ISOMERIZATION |  | 31 | -0.26 | -0.88 | 0.628 | 0.869 | 1.000 | 1366 | tags=19%, list=11%, signal=22% |
| 2201 | GOBP\_POSITIVE\_REGULATION\_OF\_GRANULOCYTE\_CHEMOTAXIS |  | 25 | -0.27 | -0.88 | 0.652 | 0.869 | 1.000 | 2204 | tags=24%, list=17%, signal=29% |
| 2202 | GOBP\_BONE\_MINERALIZATION |  | 102 | -0.20 | -0.88 | 0.696 | 0.870 | 1.000 | 2074 | tags=21%, list=16%, signal=24% |
| 2203 | GOBP\_MORPHOGENESIS\_OF\_AN\_EPITHELIAL\_FOLD |  | 19 | -0.29 | -0.88 | 0.637 | 0.870 | 1.000 | 1069 | tags=16%, list=8%, signal=17% |
| 2204 | GOBP\_POSITIVE\_REGULATION\_OF\_INSULIN\_SECRETION\_INVOLVED\_IN\_CELLULAR\_RESPONSE\_TO\_GLUCOSE\_STIMULUS |  | 24 | -0.27 | -0.88 | 0.654 | 0.870 | 1.000 | 3742 | tags=42%, list=29%, signal=59% |
| 2205 | GOBP\_INTRACELLULAR\_PROTEIN\_TRANSMEMBRANE\_TRANSPORT |  | 41 | -0.24 | -0.88 | 0.635 | 0.870 | 1.000 | 2165 | tags=27%, list=17%, signal=32% |
| 2206 | GOBP\_B\_CELL\_ACTIVATION\_INVOLVED\_IN\_IMMUNE\_RESPONSE |  | 71 | -0.21 | -0.88 | 0.664 | 0.871 | 1.000 | 2235 | tags=20%, list=17%, signal=24% |
| 2207 | GOBP\_CELLULAR\_RESPONSE\_TO\_CADMIUM\_ION |  | 31 | -0.26 | -0.88 | 0.636 | 0.870 | 1.000 | 2508 | tags=29%, list=19%, signal=36% |
| 2208 | GOBP\_MALE\_GAMETE\_GENERATION |  | 379 | -0.17 | -0.88 | 0.821 | 0.870 | 1.000 | 4087 | tags=32%, list=32%, signal=46% |
| 2209 | GOBP\_INOSITOL\_PHOSPHATE\_BIOSYNTHETIC\_PROCESS |  | 26 | -0.26 | -0.88 | 0.664 | 0.870 | 1.000 | 1284 | tags=15%, list=10%, signal=17% |
| 2210 | GOBP\_CELLULAR\_COMPONENT\_ASSEMBLY\_INVOLVED\_IN\_MORPHOGENESIS |  | 96 | -0.20 | -0.88 | 0.710 | 0.871 | 1.000 | 2159 | tags=18%, list=17%, signal=21% |
| 2211 | GOBP\_INFLAMMATORY\_RESPONSE\_TO\_ANTIGENIC\_STIMULUS |  | 63 | -0.22 | -0.88 | 0.679 | 0.871 | 1.000 | 2182 | tags=21%, list=17%, signal=25% |
| 2212 | GOBP\_TISSUE\_REGENERATION |  | 56 | -0.23 | -0.88 | 0.690 | 0.871 | 1.000 | 823 | tags=13%, list=6%, signal=13% |
| 2213 | GOBP\_INNER\_EAR\_AUDITORY\_RECEPTOR\_CELL\_DIFFERENTIATION |  | 25 | -0.27 | -0.88 | 0.637 | 0.871 | 1.000 | 2616 | tags=28%, list=20%, signal=35% |
| 2214 | GOBP\_PHOSPHATE\_ION\_TRANSPORT |  | 22 | -0.28 | -0.88 | 0.656 | 0.873 | 1.000 | 903 | tags=14%, list=7%, signal=15% |
| 2215 | GOBP\_INNATE\_IMMUNE\_RESPONSE\_IN\_MUCOSA |  | 22 | -0.28 | -0.88 | 0.634 | 0.873 | 1.000 | 4670 | tags=41%, list=36%, signal=64% |
| 2216 | GOBP\_HISTONE\_EXCHANGE |  | 16 | -0.30 | -0.88 | 0.637 | 0.873 | 1.000 | 2208 | tags=31%, list=17%, signal=38% |
| 2217 | GOBP\_FLUID\_TRANSPORT |  | 29 | -0.26 | -0.88 | 0.644 | 0.873 | 1.000 | 3169 | tags=31%, list=25%, signal=41% |
| 2218 | GOBP\_TUBE\_FORMATION |  | 119 | -0.20 | -0.88 | 0.725 | 0.874 | 1.000 | 1426 | tags=14%, list=11%, signal=16% |
| 2219 | GOBP\_REGULATION\_OF\_VESICLE\_MEDIATED\_TRANSPORT |  | 442 | -0.17 | -0.88 | 0.835 | 0.874 | 1.000 | 3083 | tags=24%, list=24%, signal=31% |
| 2220 | GOBP\_HETEROPHILIC\_CELL\_CELL\_ADHESION\_VIA\_PLASMA\_MEMBRANE\_CELL\_ADHESION\_MOLECULES |  | 37 | -0.24 | -0.88 | 0.653 | 0.874 | 1.000 | 428 | tags=8%, list=3%, signal=8% |
| 2221 | GOBP\_NEGATIVE\_REGULATION\_OF\_PRODUCTION\_OF\_MOLECULAR\_MEDIATOR\_OF\_IMMUNE\_RESPONSE |  | 36 | -0.24 | -0.88 | 0.659 | 0.875 | 1.000 | 2101 | tags=22%, list=16%, signal=26% |
| 2222 | GOBP\_MONOVALENT\_INORGANIC\_CATION\_HOMEOSTASIS |  | 129 | -0.19 | -0.88 | 0.718 | 0.875 | 1.000 | 3365 | tags=29%, list=26%, signal=39% |
| 2223 | GOBP\_RETINOIC\_ACID\_METABOLIC\_PROCESS |  | 25 | -0.27 | -0.88 | 0.641 | 0.875 | 1.000 | 4904 | tags=56%, list=38%, signal=90% |
| 2224 | GOBP\_NEGATIVE\_REGULATION\_OF\_ACTIN\_FILAMENT\_DEPOLYMERIZATION |  | 36 | -0.25 | -0.88 | 0.688 | 0.877 | 1.000 | 4590 | tags=36%, list=36%, signal=56% |
| 2225 | GOBP\_MODIFIED\_AMINO\_ACID\_TRANSPORT |  | 28 | -0.26 | -0.87 | 0.633 | 0.878 | 1.000 | 3836 | tags=46%, list=30%, signal=66% |
| 2226 | GOBP\_PHOSPHATIDYLINOSITOL\_METABOLIC\_PROCESS |  | 130 | -0.19 | -0.87 | 0.773 | 0.879 | 1.000 | 2452 | tags=22%, list=19%, signal=27% |
| 2227 | GOBP\_PHOSPHOLIPID\_METABOLIC\_PROCESS |  | 297 | -0.17 | -0.87 | 0.822 | 0.881 | 1.000 | 2473 | tags=20%, list=19%, signal=24% |
| 2228 | GOBP\_SPERM\_CAPACITATION |  | 19 | -0.29 | -0.87 | 0.646 | 0.881 | 1.000 | 3897 | tags=53%, list=30%, signal=75% |
| 2229 | GOBP\_REGULATION\_OF\_NEUROBLAST\_PROLIFERATION |  | 27 | -0.26 | -0.87 | 0.638 | 0.880 | 1.000 | 1069 | tags=15%, list=8%, signal=16% |
| 2230 | GOBP\_RNA\_MODIFICATION |  | 112 | -0.19 | -0.87 | 0.743 | 0.880 | 1.000 | 2607 | tags=24%, list=20%, signal=30% |
| 2231 | GOBP\_SEROTONIN\_TRANSPORT |  | 18 | -0.29 | -0.87 | 0.638 | 0.881 | 1.000 | 3777 | tags=44%, list=29%, signal=63% |
| 2232 | GOBP\_MIDDLE\_EAR\_MORPHOGENESIS |  | 16 | -0.30 | -0.87 | 0.635 | 0.882 | 1.000 | 2638 | tags=38%, list=20%, signal=47% |
| 2233 | GOBP\_VASCULAR\_ASSOCIATED\_SMOOTH\_MUSCLE\_CONTRACTION |  | 23 | -0.27 | -0.87 | 0.642 | 0.883 | 1.000 | 4522 | tags=48%, list=35%, signal=74% |
| 2234 | GOBP\_NEUROTROPHIN\_TRK\_RECEPTOR\_SIGNALING\_PATHWAY |  | 20 | -0.29 | -0.87 | 0.632 | 0.883 | 1.000 | 2223 | tags=30%, list=17%, signal=36% |
| 2235 | GOBP\_POSITIVE\_REGULATION\_OF\_LIPID\_LOCALIZATION |  | 94 | -0.20 | -0.87 | 0.708 | 0.884 | 1.000 | 4065 | tags=36%, list=32%, signal=52% |
| 2236 | GOBP\_REGULATION\_OF\_SUPRAMOLECULAR\_FIBER\_ORGANIZATION |  | 311 | -0.17 | -0.87 | 0.814 | 0.884 | 1.000 | 3160 | tags=26%, list=24%, signal=33% |
| 2237 | GOBP\_WATER\_TRANSPORT |  | 20 | -0.28 | -0.87 | 0.643 | 0.885 | 1.000 | 4969 | tags=50%, list=39%, signal=81% |
| 2238 | GOBP\_OSTEOBLAST\_PROLIFERATION |  | 26 | -0.27 | -0.87 | 0.668 | 0.885 | 1.000 | 2220 | tags=27%, list=17%, signal=32% |
| 2239 | GOBP\_NEGATIVE\_REGULATION\_OF\_LEUKOCYTE\_PROLIFERATION |  | 78 | -0.21 | -0.87 | 0.724 | 0.885 | 1.000 | 2461 | tags=22%, list=19%, signal=27% |
| 2240 | GOBP\_AXONAL\_TRANSPORT |  | 51 | -0.23 | -0.87 | 0.695 | 0.885 | 1.000 | 2150 | tags=22%, list=17%, signal=26% |
| 2241 | GOBP\_STARTLE\_RESPONSE |  | 21 | -0.28 | -0.87 | 0.684 | 0.886 | 1.000 | 1596 | tags=24%, list=12%, signal=27% |
| 2242 | GOBP\_RESPONSE\_TO\_AUDITORY\_STIMULUS |  | 21 | -0.28 | -0.87 | 0.653 | 0.885 | 1.000 | 4816 | tags=52%, list=37%, signal=83% |
| 2243 | GOBP\_RESPONSE\_TO\_KETONE |  | 170 | -0.19 | -0.87 | 0.795 | 0.885 | 1.000 | 2306 | tags=19%, list=18%, signal=23% |
| 2244 | GOBP\_ADIPOSE\_TISSUE\_DEVELOPMENT |  | 36 | -0.24 | -0.87 | 0.666 | 0.885 | 1.000 | 4754 | tags=53%, list=37%, signal=83% |
| 2245 | GOBP\_TOLL\_LIKE\_RECEPTOR\_SIGNALING\_PATHWAY |  | 98 | -0.20 | -0.87 | 0.738 | 0.887 | 1.000 | 2028 | tags=19%, list=16%, signal=23% |
| 2246 | GOBP\_RENAL\_SYSTEM\_PROCESS |  | 103 | -0.20 | -0.87 | 0.741 | 0.888 | 1.000 | 4455 | tags=43%, list=35%, signal=65% |
| 2247 | GOBP\_VACUOLAR\_LOCALIZATION |  | 71 | -0.21 | -0.87 | 0.729 | 0.888 | 1.000 | 2000 | tags=18%, list=16%, signal=22% |
| 2248 | GOBP\_MACROPHAGE\_CHEMOTAXIS |  | 35 | -0.24 | -0.87 | 0.666 | 0.888 | 1.000 | 1934 | tags=23%, list=15%, signal=27% |
| 2249 | GOBP\_CERAMIDE\_METABOLIC\_PROCESS |  | 81 | -0.21 | -0.87 | 0.710 | 0.888 | 1.000 | 1774 | tags=19%, list=14%, signal=21% |
| 2250 | GOBP\_PROTEASOMAL\_PROTEIN\_CATABOLIC\_PROCESS |  | 370 | -0.17 | -0.87 | 0.864 | 0.890 | 1.000 | 2415 | tags=19%, list=19%, signal=23% |
| 2251 | GOBP\_IMP\_METABOLIC\_PROCESS |  | 16 | -0.30 | -0.87 | 0.678 | 0.890 | 1.000 | 3427 | tags=50%, list=27%, signal=68% |
| 2252 | GOBP\_NEGATIVE\_REGULATION\_OF\_DNA\_DAMAGE\_RESPONSE\_SIGNAL\_TRANSDUCTION\_BY\_P53\_CLASS\_MEDIATOR |  | 15 | -0.30 | -0.86 | 0.654 | 0.891 | 1.000 | 1463 | tags=27%, list=11%, signal=30% |
| 2253 | GOBP\_REGULATION\_OF\_SYNAPTIC\_TRANSMISSION\_GLUTAMATERGIC |  | 57 | -0.22 | -0.86 | 0.694 | 0.891 | 1.000 | 3118 | tags=28%, list=24%, signal=37% |
| 2254 | GOBP\_REGULATION\_OF\_PEPTIDYL\_TYROSINE\_PHOSPHORYLATION |  | 220 | -0.18 | -0.86 | 0.798 | 0.891 | 1.000 | 2124 | tags=18%, list=16%, signal=21% |
| 2255 | GOBP\_POSITIVE\_REGULATION\_OF\_CELLULAR\_RESPONSE\_TO\_INSULIN\_STIMULUS |  | 21 | -0.28 | -0.86 | 0.647 | 0.891 | 1.000 | 4421 | tags=52%, list=34%, signal=80% |
| 2256 | GOBP\_SUBSTRATE\_DEPENDENT\_CELL\_MIGRATION |  | 21 | -0.28 | -0.86 | 0.643 | 0.891 | 1.000 | 3936 | tags=48%, list=31%, signal=68% |
| 2257 | GOBP\_REGULATION\_OF\_ANDROGEN\_RECEPTOR\_SIGNALING\_PATHWAY |  | 26 | -0.27 | -0.86 | 0.680 | 0.890 | 1.000 | 1614 | tags=23%, list=13%, signal=26% |
| 2258 | GOBP\_RESPONSE\_TO\_OXIDATIVE\_STRESS |  | 364 | -0.17 | -0.86 | 0.862 | 0.891 | 1.000 | 2482 | tags=20%, list=19%, signal=23% |
| 2259 | GOBP\_SECRETORY\_GRANULE\_ORGANIZATION |  | 25 | -0.26 | -0.86 | 0.660 | 0.891 | 1.000 | 3028 | tags=32%, list=23%, signal=42% |
| 2260 | GOBP\_REGULATION\_OF\_RESPONSE\_TO\_OXIDATIVE\_STRESS |  | 78 | -0.21 | -0.86 | 0.716 | 0.891 | 1.000 | 1750 | tags=18%, list=14%, signal=21% |
| 2261 | GOBP\_MUSCLE\_CONTRACTION |  | 299 | -0.17 | -0.86 | 0.834 | 0.891 | 1.000 | 3118 | tags=22%, list=24%, signal=29% |
| 2262 | GOBP\_REGULATION\_OF\_CD4\_POSITIVE\_ALPHA\_BETA\_T\_CELL\_ACTIVATION |  | 51 | -0.22 | -0.86 | 0.723 | 0.890 | 1.000 | 2078 | tags=18%, list=16%, signal=21% |
| 2263 | GOBP\_PHENOL\_CONTAINING\_COMPOUND\_BIOSYNTHETIC\_PROCESS |  | 44 | -0.23 | -0.86 | 0.704 | 0.890 | 1.000 | 2911 | tags=25%, list=23%, signal=32% |
| 2264 | GOBP\_NEGATIVE\_REGULATION\_OF\_REGULATED\_SECRETORY\_PATHWAY |  | 23 | -0.27 | -0.86 | 0.660 | 0.890 | 1.000 | 520 | tags=13%, list=4%, signal=14% |
| 2265 | GOBP\_REGULATION\_OF\_CALCIUM\_ION\_TRANSPORT\_INTO\_CYTOSOL |  | 84 | -0.21 | -0.86 | 0.733 | 0.891 | 1.000 | 2982 | tags=25%, list=23%, signal=32% |
| 2266 | GOBP\_REGULATION\_OF\_INTRACELLULAR\_TRANSPORT |  | 282 | -0.17 | -0.86 | 0.844 | 0.891 | 1.000 | 2970 | tags=24%, list=23%, signal=30% |
| 2267 | GOBP\_GLUTAMINE\_FAMILY\_AMINO\_ACID\_CATABOLIC\_PROCESS |  | 24 | -0.27 | -0.86 | 0.667 | 0.891 | 1.000 | 1355 | tags=21%, list=11%, signal=23% |
| 2268 | GOBP\_REGULATION\_OF\_CELL\_MORPHOGENESIS\_INVOLVED\_IN\_DIFFERENTIATION |  | 87 | -0.20 | -0.86 | 0.728 | 0.891 | 1.000 | 4156 | tags=32%, list=32%, signal=47% |
| 2269 | GOBP\_HYDROGEN\_PEROXIDE\_METABOLIC\_PROCESS |  | 41 | -0.23 | -0.86 | 0.682 | 0.892 | 1.000 | 2429 | tags=27%, list=19%, signal=33% |
| 2270 | GOBP\_DICARBOXYLIC\_ACID\_TRANSPORT |  | 79 | -0.21 | -0.86 | 0.738 | 0.893 | 1.000 | 4787 | tags=44%, list=37%, signal=70% |
| 2271 | GOBP\_DNA\_TEMPLATED\_TRANSCRIPTION\_ELONGATION |  | 69 | -0.21 | -0.86 | 0.720 | 0.893 | 1.000 | 3055 | tags=29%, list=24%, signal=38% |
| 2272 | GOBP\_POSITIVE\_REGULATION\_OF\_CYSTEINE\_TYPE\_ENDOPEPTIDASE\_ACTIVITY |  | 126 | -0.19 | -0.86 | 0.760 | 0.892 | 1.000 | 1713 | tags=15%, list=13%, signal=17% |
| 2273 | GOBP\_REGULATION\_OF\_RESPIRATORY\_BURST |  | 15 | -0.30 | -0.86 | 0.655 | 0.893 | 1.000 | 3237 | tags=33%, list=25%, signal=44% |
| 2274 | GOBP\_POSITIVE\_REGULATION\_OF\_NEUTROPHIL\_MIGRATION |  | 28 | -0.26 | -0.86 | 0.681 | 0.893 | 1.000 | 3133 | tags=32%, list=24%, signal=42% |
| 2275 | GOBP\_MONOAMINE\_TRANSPORT |  | 72 | -0.21 | -0.86 | 0.716 | 0.893 | 1.000 | 4646 | tags=44%, list=36%, signal=69% |
| 2276 | GOBP\_REGULATION\_OF\_ISOTYPE\_SWITCHING |  | 30 | -0.26 | -0.86 | 0.651 | 0.893 | 1.000 | 542 | tags=10%, list=4%, signal=10% |
| 2277 | GOBP\_REGULATION\_OF\_CELL\_ADHESION\_MEDIATED\_BY\_INTEGRIN |  | 42 | -0.23 | -0.86 | 0.685 | 0.893 | 1.000 | 3605 | tags=36%, list=28%, signal=49% |
| 2278 | GOBP\_HISTONE\_PHOSPHORYLATION |  | 42 | -0.23 | -0.86 | 0.696 | 0.894 | 1.000 | 1833 | tags=19%, list=14%, signal=22% |
| 2279 | GOBP\_MYELOID\_CELL\_APOPTOTIC\_PROCESS |  | 30 | -0.25 | -0.86 | 0.682 | 0.895 | 1.000 | 2085 | tags=27%, list=16%, signal=32% |
| 2280 | GOBP\_REGULATION\_OF\_PROTEIN\_CONTAINING\_COMPLEX\_DISASSEMBLY |  | 105 | -0.20 | -0.86 | 0.761 | 0.894 | 1.000 | 2889 | tags=25%, list=22%, signal=32% |
| 2281 | GOBP\_ONE\_CARBON\_METABOLIC\_PROCESS |  | 35 | -0.25 | -0.86 | 0.688 | 0.894 | 1.000 | 3694 | tags=29%, list=29%, signal=40% |
| 2282 | GOBP\_NEGATIVE\_REGULATION\_OF\_ENDOCYTOSIS |  | 42 | -0.23 | -0.86 | 0.697 | 0.894 | 1.000 | 1825 | tags=19%, list=14%, signal=22% |
| 2283 | GOBP\_NEURON\_CELLULAR\_HOMEOSTASIS |  | 32 | -0.25 | -0.86 | 0.678 | 0.894 | 1.000 | 3015 | tags=34%, list=23%, signal=45% |
| 2284 | GOBP\_ACTIN\_FILAMENT\_POLYMERIZATION |  | 132 | -0.19 | -0.86 | 0.755 | 0.894 | 1.000 | 3465 | tags=30%, list=27%, signal=40% |
| 2285 | GOBP\_REGULATION\_OF\_JUN\_KINASE\_ACTIVITY |  | 53 | -0.22 | -0.86 | 0.723 | 0.894 | 1.000 | 2946 | tags=28%, list=23%, signal=37% |
| 2286 | GOBP\_REGULATION\_OF\_INSULIN\_SECRETION\_INVOLVED\_IN\_CELLULAR\_RESPONSE\_TO\_GLUCOSE\_STIMULUS |  | 40 | -0.23 | -0.86 | 0.690 | 0.895 | 1.000 | 2493 | tags=25%, list=19%, signal=31% |
| 2287 | GOBP\_MITOTIC\_CELL\_CYCLE\_CHECKPOINT\_SIGNALING |  | 106 | -0.19 | -0.86 | 0.788 | 0.895 | 1.000 | 1444 | tags=12%, list=11%, signal=14% |
| 2288 | GOBP\_POSITIVE\_REGULATION\_OF\_TRANSMEMBRANE\_TRANSPORT |  | 176 | -0.18 | -0.86 | 0.814 | 0.895 | 1.000 | 3083 | tags=27%, list=24%, signal=35% |
| 2289 | GOBP\_DENDRITIC\_CELL\_DIFFERENTIATION |  | 45 | -0.23 | -0.86 | 0.715 | 0.895 | 1.000 | 4661 | tags=51%, list=36%, signal=80% |
| 2290 | GOBP\_CATECHOLAMINE\_SECRETION |  | 48 | -0.23 | -0.86 | 0.719 | 0.897 | 1.000 | 4646 | tags=46%, list=36%, signal=71% |
| 2291 | GOBP\_PROTEIN\_LOCALIZATION\_TO\_PLASMA\_MEMBRANE |  | 228 | -0.18 | -0.86 | 0.853 | 0.897 | 1.000 | 4081 | tags=34%, list=32%, signal=49% |
| 2292 | GOBP\_REGULATION\_OF\_PEPTIDASE\_ACTIVITY |  | 359 | -0.17 | -0.86 | 0.868 | 0.897 | 1.000 | 2976 | tags=23%, list=23%, signal=29% |
| 2293 | GOBP\_EPHRIN\_RECEPTOR\_SIGNALING\_PATHWAY |  | 47 | -0.23 | -0.85 | 0.739 | 0.898 | 1.000 | 4708 | tags=47%, list=36%, signal=73% |
| 2294 | GOBP\_NEGATIVE\_REGULATION\_OF\_INTERLEUKIN\_12\_PRODUCTION |  | 17 | -0.29 | -0.85 | 0.655 | 0.898 | 1.000 | 2539 | tags=24%, list=20%, signal=29% |
| 2295 | GOBP\_RNA\_DEPENDENT\_DNA\_BIOSYNTHETIC\_PROCESS |  | 59 | -0.22 | -0.85 | 0.730 | 0.898 | 1.000 | 3113 | tags=25%, list=24%, signal=33% |
| 2296 | GOBP\_FOREBRAIN\_REGIONALIZATION |  | 19 | -0.28 | -0.85 | 0.684 | 0.899 | 1.000 | 3961 | tags=37%, list=31%, signal=53% |
| 2297 | GOBP\_LONG\_CHAIN\_FATTY\_ACID\_BIOSYNTHETIC\_PROCESS |  | 24 | -0.26 | -0.85 | 0.675 | 0.900 | 1.000 | 3285 | tags=38%, list=25%, signal=50% |
| 2298 | GOBP\_PROTEIN\_POLYUBIQUITINATION |  | 182 | -0.18 | -0.85 | 0.803 | 0.900 | 1.000 | 1935 | tags=16%, list=15%, signal=19% |
| 2299 | GOBP\_LONG\_CHAIN\_FATTY\_ACID\_METABOLIC\_PROCESS |  | 85 | -0.20 | -0.85 | 0.780 | 0.900 | 1.000 | 4856 | tags=47%, list=38%, signal=75% |
| 2300 | GOBP\_POSITIVE\_REGULATION\_OF\_SUBSTRATE\_ADHESION\_DEPENDENT\_CELL\_SPREADING |  | 39 | -0.23 | -0.85 | 0.706 | 0.900 | 1.000 | 540 | tags=10%, list=4%, signal=11% |
| 2301 | GOBP\_CELL\_DEATH\_IN\_RESPONSE\_TO\_HYDROGEN\_PEROXIDE |  | 24 | -0.26 | -0.85 | 0.681 | 0.900 | 1.000 | 1750 | tags=21%, list=14%, signal=24% |
| 2302 | GOBP\_EMBRYONIC\_CRANIAL\_SKELETON\_MORPHOGENESIS |  | 39 | -0.24 | -0.85 | 0.687 | 0.902 | 1.000 | 2132 | tags=21%, list=17%, signal=24% |
| 2303 | GOBP\_NEGATIVE\_REGULATION\_OF\_STEROID\_METABOLIC\_PROCESS |  | 23 | -0.27 | -0.85 | 0.671 | 0.902 | 1.000 | 571 | tags=13%, list=4%, signal=14% |
| 2304 | GOBP\_NEGATIVE\_REGULATION\_OF\_NUCLEOTIDE\_METABOLIC\_PROCESS |  | 19 | -0.28 | -0.85 | 0.668 | 0.903 | 1.000 | 4578 | tags=53%, list=35%, signal=81% |
| 2305 | GOBP\_REGULATION\_OF\_LYMPHOCYTE\_CHEMOTAXIS |  | 23 | -0.26 | -0.85 | 0.690 | 0.903 | 1.000 | 4027 | tags=43%, list=31%, signal=63% |
| 2306 | GOBP\_REGULATION\_OF\_STEROID\_BIOSYNTHETIC\_PROCESS |  | 61 | -0.21 | -0.85 | 0.749 | 0.904 | 1.000 | 3441 | tags=30%, list=27%, signal=40% |
| 2307 | GOBP\_POSITIVE\_REGULATION\_OF\_AMINE\_TRANSPORT |  | 37 | -0.24 | -0.85 | 0.720 | 0.906 | 1.000 | 2459 | tags=22%, list=19%, signal=27% |
| 2308 | GOBP\_REGULATION\_OF\_MITOCHONDRIAL\_OUTER\_MEMBRANE\_PERMEABILIZATION\_INVOLVED\_IN\_APOPTOTIC\_SIGNALING\_PATHWAY |  | 17 | -0.29 | -0.85 | 0.681 | 0.908 | 1.000 | 1745 | tags=24%, list=14%, signal=27% |
| 2309 | GOBP\_BRAIN\_MORPHOGENESIS |  | 29 | -0.25 | -0.85 | 0.712 | 0.909 | 1.000 | 1597 | tags=24%, list=12%, signal=27% |
| 2310 | GOBP\_DNA\_REPLICATION\_INDEPENDENT\_CHROMATIN\_ORGANIZATION |  | 26 | -0.26 | -0.85 | 0.714 | 0.908 | 1.000 | 932 | tags=15%, list=7%, signal=17% |
| 2311 | GOBP\_NUCLEOSOME\_ORGANIZATION |  | 132 | -0.19 | -0.85 | 0.797 | 0.908 | 1.000 | 2248 | tags=18%, list=17%, signal=22% |
| 2312 | GOBP\_CORONARY\_VASCULATURE\_DEVELOPMENT |  | 37 | -0.23 | -0.85 | 0.708 | 0.908 | 1.000 | 2783 | tags=22%, list=22%, signal=27% |
| 2313 | GOBP\_REGULATION\_OF\_EPIDERMAL\_GROWTH\_FACTOR\_ACTIVATED\_RECEPTOR\_ACTIVITY |  | 24 | -0.27 | -0.85 | 0.691 | 0.908 | 1.000 | 3423 | tags=46%, list=27%, signal=62% |
| 2314 | GOBP\_NEGATIVE\_REGULATION\_OF\_CHONDROCYTE\_DIFFERENTIATION |  | 18 | -0.28 | -0.85 | 0.664 | 0.907 | 1.000 | 436 | tags=11%, list=3%, signal=11% |
| 2315 | GOBP\_PROTEIN\_ADP\_RIBOSYLATION |  | 32 | -0.24 | -0.85 | 0.715 | 0.908 | 1.000 | 1131 | tags=13%, list=9%, signal=14% |
| 2316 | GOBP\_OLIGODENDROCYTE\_DEVELOPMENT |  | 37 | -0.24 | -0.85 | 0.714 | 0.910 | 1.000 | 1066 | tags=14%, list=8%, signal=15% |
| 2317 | GOBP\_NEGATIVE\_REGULATION\_OF\_PLASMA\_MEMBRANE\_BOUNDED\_CELL\_PROJECTION\_ASSEMBLY |  | 27 | -0.25 | -0.84 | 0.715 | 0.910 | 1.000 | 3090 | tags=30%, list=24%, signal=39% |
| 2318 | GOBP\_NEGATIVE\_REGULATION\_OF\_CYTOKINE\_PRODUCTION\_INVOLVED\_IN\_IMMUNE\_RESPONSE |  | 27 | -0.25 | -0.84 | 0.690 | 0.911 | 1.000 | 2101 | tags=22%, list=16%, signal=26% |
| 2319 | GOBP\_PHOTOTRANSDUCTION\_VISIBLE\_LIGHT |  | 17 | -0.29 | -0.84 | 0.683 | 0.912 | 1.000 | 527 | tags=12%, list=4%, signal=12% |
| 2320 | GOBP\_HORMONE\_METABOLIC\_PROCESS |  | 196 | -0.18 | -0.84 | 0.825 | 0.912 | 1.000 | 2085 | tags=18%, list=16%, signal=21% |
| 2321 | GOBP\_CARDIAC\_CHAMBER\_MORPHOGENESIS |  | 114 | -0.19 | -0.84 | 0.798 | 0.913 | 1.000 | 1174 | tags=11%, list=9%, signal=11% |
| 2322 | GOBP\_VITAMIN\_TRANSMEMBRANE\_TRANSPORT |  | 15 | -0.29 | -0.84 | 0.677 | 0.913 | 1.000 | 4170 | tags=60%, list=32%, signal=89% |
| 2323 | GOBP\_MUSCLE\_CELL\_FATE\_COMMITMENT |  | 15 | -0.30 | -0.84 | 0.697 | 0.915 | 1.000 | 4102 | tags=33%, list=32%, signal=49% |
| 2324 | GOBP\_POSITIVE\_REGULATION\_OF\_MITOTIC\_CELL\_CYCLE\_PHASE\_TRANSITION |  | 67 | -0.21 | -0.84 | 0.757 | 0.918 | 1.000 | 972 | tags=12%, list=8%, signal=13% |
| 2325 | GOBP\_EMBRYONIC\_PATTERN\_SPECIFICATION |  | 54 | -0.22 | -0.84 | 0.746 | 0.918 | 1.000 | 4456 | tags=39%, list=35%, signal=59% |
| 2326 | GOBP\_RESPONSE\_TO\_MUSCLE\_ACTIVITY |  | 15 | -0.29 | -0.84 | 0.669 | 0.918 | 1.000 | 1069 | tags=20%, list=8%, signal=22% |
| 2327 | GOBP\_PROTEIN\_FOLDING |  | 167 | -0.18 | -0.84 | 0.833 | 0.918 | 1.000 | 3974 | tags=37%, list=31%, signal=53% |
| 2328 | GOBP\_POSITIVE\_REGULATION\_OF\_ACTIN\_FILAMENT\_BUNDLE\_ASSEMBLY |  | 60 | -0.21 | -0.84 | 0.756 | 0.918 | 1.000 | 1522 | tags=15%, list=12%, signal=17% |
| 2329 | GOBP\_CHONDROCYTE\_DIFFERENTIATION |  | 93 | -0.19 | -0.84 | 0.806 | 0.919 | 1.000 | 4356 | tags=38%, list=34%, signal=56% |
| 2330 | GOBP\_DETECTION\_OF\_STIMULUS\_INVOLVED\_IN\_SENSORY\_PERCEPTION |  | 116 | -0.19 | -0.84 | 0.783 | 0.919 | 1.000 | 3213 | tags=22%, list=25%, signal=30% |
| 2331 | GOBP\_ESTABLISHMENT\_OF\_MITOTIC\_SPINDLE\_LOCALIZATION |  | 30 | -0.24 | -0.84 | 0.730 | 0.919 | 1.000 | 2312 | tags=23%, list=18%, signal=28% |
| 2332 | GOBP\_B\_CELL\_HOMEOSTASIS |  | 25 | -0.26 | -0.84 | 0.715 | 0.919 | 1.000 | 1800 | tags=20%, list=14%, signal=23% |
| 2333 | GOBP\_REGULATION\_OF\_LEUKOCYTE\_MEDIATED\_IMMUNITY |  | 206 | -0.17 | -0.84 | 0.871 | 0.919 | 1.000 | 2101 | tags=17%, list=16%, signal=21% |
| 2334 | GOBP\_REGULATION\_OF\_PROTEIN\_LOCALIZATION\_TO\_NUCLEUS |  | 114 | -0.19 | -0.84 | 0.782 | 0.919 | 1.000 | 3113 | tags=30%, list=24%, signal=39% |
| 2335 | GOBP\_ACTOMYOSIN\_STRUCTURE\_ORGANIZATION |  | 170 | -0.18 | -0.84 | 0.850 | 0.920 | 1.000 | 1803 | tags=14%, list=14%, signal=16% |
| 2336 | GOBP\_SALIVARY\_GLAND\_DEVELOPMENT |  | 33 | -0.24 | -0.84 | 0.725 | 0.920 | 1.000 | 1554 | tags=18%, list=12%, signal=21% |
| 2337 | GOBP\_RENAL\_SYSTEM\_PROCESS\_INVOLVED\_IN\_REGULATION\_OF\_SYSTEMIC\_ARTERIAL\_BLOOD\_PRESSURE |  | 24 | -0.26 | -0.84 | 0.709 | 0.921 | 1.000 | 4455 | tags=58%, list=35%, signal=89% |
| 2338 | GOBP\_POSITIVE\_REGULATION\_OF\_LEUKOCYTE\_DEGRANULATION |  | 24 | -0.26 | -0.84 | 0.723 | 0.921 | 1.000 | 4271 | tags=46%, list=33%, signal=68% |
| 2339 | GOBP\_POSITIVE\_REGULATION\_OF\_RHO\_PROTEIN\_SIGNAL\_TRANSDUCTION |  | 26 | -0.26 | -0.84 | 0.725 | 0.921 | 1.000 | 4455 | tags=46%, list=35%, signal=70% |
| 2340 | GOBP\_FERTILIZATION |  | 129 | -0.18 | -0.84 | 0.820 | 0.920 | 1.000 | 4714 | tags=43%, list=37%, signal=67% |
| 2341 | GOBP\_PROTEIN\_K63\_LINKED\_UBIQUITINATION |  | 47 | -0.22 | -0.84 | 0.738 | 0.921 | 1.000 | 2817 | tags=26%, list=22%, signal=33% |
| 2342 | GOBP\_DETECTION\_OF\_VISIBLE\_LIGHT |  | 37 | -0.24 | -0.83 | 0.749 | 0.924 | 1.000 | 2600 | tags=27%, list=20%, signal=34% |
| 2343 | GOBP\_COCHLEA\_DEVELOPMENT |  | 40 | -0.23 | -0.83 | 0.726 | 0.925 | 1.000 | 2024 | tags=23%, list=16%, signal=27% |
| 2344 | GOBP\_MITOCHONDRIAL\_GENOME\_MAINTENANCE |  | 17 | -0.29 | -0.83 | 0.694 | 0.926 | 1.000 | 754 | tags=12%, list=6%, signal=12% |
| 2345 | GOBP\_POSITIVE\_REGULATION\_OF\_PHOSPHATIDYLINOSITOL\_3\_KINASE\_SIGNALING |  | 73 | -0.20 | -0.83 | 0.772 | 0.926 | 1.000 | 1601 | tags=15%, list=12%, signal=17% |
| 2346 | GOBP\_TRIGLYCERIDE\_BIOSYNTHETIC\_PROCESS |  | 21 | -0.27 | -0.83 | 0.717 | 0.927 | 1.000 | 4528 | tags=48%, list=35%, signal=73% |
| 2347 | GOBP\_REGULATION\_OF\_CELLULAR\_EXTRAVASATION |  | 34 | -0.24 | -0.83 | 0.712 | 0.927 | 1.000 | 3526 | tags=38%, list=27%, signal=52% |
| 2348 | GOBP\_NEGATIVE\_REGULATION\_OF\_LEUKOCYTE\_APOPTOTIC\_PROCESS |  | 41 | -0.23 | -0.83 | 0.752 | 0.927 | 1.000 | 3669 | tags=32%, list=28%, signal=44% |
| 2349 | GOBP\_POSITIVE\_REGULATION\_OF\_ERK1\_AND\_ERK2\_CASCADE |  | 184 | -0.18 | -0.83 | 0.856 | 0.927 | 1.000 | 3588 | tags=28%, list=28%, signal=38% |
| 2350 | GOBP\_LEUKOCYTE\_TETHERING\_OR\_ROLLING |  | 31 | -0.24 | -0.83 | 0.727 | 0.927 | 1.000 | 856 | tags=13%, list=7%, signal=14% |
| 2351 | GOBP\_CELL\_ACTIVATION\_INVOLVED\_IN\_IMMUNE\_RESPONSE |  | 241 | -0.17 | -0.83 | 0.905 | 0.928 | 1.000 | 4271 | tags=35%, list=33%, signal=51% |
| 2352 | GOBP\_TRANSPORT\_ALONG\_MICROTUBULE |  | 125 | -0.18 | -0.83 | 0.822 | 0.928 | 1.000 | 2150 | tags=19%, list=17%, signal=23% |
| 2353 | GOBP\_REGULATION\_OF\_PEPTIDYL\_THREONINE\_PHOSPHORYLATION |  | 34 | -0.23 | -0.83 | 0.758 | 0.928 | 1.000 | 3691 | tags=32%, list=29%, signal=45% |
| 2354 | GOBP\_REGULATION\_OF\_CATION\_TRANSMEMBRANE\_TRANSPORT |  | 256 | -0.17 | -0.83 | 0.887 | 0.929 | 1.000 | 3118 | tags=25%, list=24%, signal=32% |
| 2355 | GOBP\_EMBRYONIC\_SKELETAL\_SYSTEM\_MORPHOGENESIS |  | 79 | -0.20 | -0.83 | 0.799 | 0.930 | 1.000 | 3019 | tags=25%, list=23%, signal=33% |
| 2356 | GOBP\_NEGATIVE\_REGULATION\_OF\_PROTEASOMAL\_UBIQUITIN\_DEPENDENT\_PROTEIN\_CATABOLIC\_PROCESS |  | 29 | -0.24 | -0.83 | 0.742 | 0.930 | 1.000 | 3161 | tags=31%, list=25%, signal=41% |
| 2357 | GOBP\_REGULATION\_OF\_INTRACELLULAR\_ESTROGEN\_RECEPTOR\_SIGNALING\_PATHWAY |  | 30 | -0.24 | -0.83 | 0.721 | 0.930 | 1.000 | 3501 | tags=30%, list=27%, signal=41% |
| 2358 | GOBP\_REGULATION\_OF\_NEUROTRANSMITTER\_RECEPTOR\_ACTIVITY |  | 41 | -0.23 | -0.83 | 0.743 | 0.930 | 1.000 | 4402 | tags=44%, list=34%, signal=66% |
| 2359 | GOBP\_POSITIVE\_REGULATION\_OF\_PROTEIN\_MODIFICATION\_BY\_SMALL\_PROTEIN\_CONJUGATION\_OR\_REMOVAL |  | 111 | -0.19 | -0.83 | 0.832 | 0.930 | 1.000 | 2567 | tags=23%, list=20%, signal=28% |
| 2360 | GOBP\_POSITIVE\_REGULATION\_OF\_BONE\_MINERALIZATION |  | 36 | -0.23 | -0.83 | 0.735 | 0.930 | 1.000 | 1983 | tags=19%, list=15%, signal=23% |
| 2361 | GOBP\_NUCLEOBASE\_CONTAINING\_SMALL\_MOLECULE\_CATABOLIC\_PROCESS |  | 26 | -0.25 | -0.83 | 0.723 | 0.930 | 1.000 | 889 | tags=15%, list=7%, signal=16% |
| 2362 | GOBP\_ASPARTATE\_FAMILY\_AMINO\_ACID\_METABOLIC\_PROCESS |  | 40 | -0.23 | -0.83 | 0.754 | 0.931 | 1.000 | 989 | tags=13%, list=8%, signal=13% |
| 2363 | GOBP\_DE\_NOVO\_PROTEIN\_FOLDING |  | 36 | -0.23 | -0.83 | 0.754 | 0.931 | 1.000 | 3762 | tags=42%, list=29%, signal=59% |
| 2364 | GOBP\_REGULATION\_OF\_PROTEIN\_TYROSINE\_KINASE\_ACTIVITY |  | 75 | -0.20 | -0.83 | 0.798 | 0.931 | 1.000 | 3423 | tags=31%, list=27%, signal=41% |
| 2365 | GOBP\_KILLING\_BY\_HOST\_OF\_SYMBIONT\_CELLS |  | 22 | -0.26 | -0.83 | 0.724 | 0.932 | 1.000 | 4336 | tags=50%, list=34%, signal=75% |
| 2366 | GOBP\_ESTABLISHMENT\_OF\_ORGANELLE\_LOCALIZATION |  | 336 | -0.16 | -0.83 | 0.935 | 0.931 | 1.000 | 2587 | tags=20%, list=20%, signal=24% |
| 2367 | GOBP\_NEURAL\_TUBE\_DEVELOPMENT |  | 124 | -0.18 | -0.82 | 0.843 | 0.931 | 1.000 | 4247 | tags=38%, list=33%, signal=56% |
| 2368 | GOBP\_NEGATIVE\_REGULATION\_OF\_ACTIN\_FILAMENT\_POLYMERIZATION |  | 51 | -0.21 | -0.82 | 0.780 | 0.931 | 1.000 | 3691 | tags=29%, list=29%, signal=41% |
| 2369 | GOBP\_REGULATION\_OF\_CELLULAR\_RESPONSE\_TO\_VASCULAR\_ENDOTHELIAL\_GROWTH\_FACTOR\_STIMULUS |  | 19 | -0.27 | -0.82 | 0.696 | 0.932 | 1.000 | 4918 | tags=47%, list=38%, signal=76% |
| 2370 | GOBP\_REGULATION\_OF\_MUSCLE\_CONTRACTION |  | 142 | -0.18 | -0.82 | 0.838 | 0.933 | 1.000 | 1254 | tags=12%, list=10%, signal=13% |
| 2371 | GOBP\_CYCLIC\_NUCLEOTIDE\_BIOSYNTHETIC\_PROCESS |  | 20 | -0.27 | -0.82 | 0.714 | 0.933 | 1.000 | 3482 | tags=35%, list=27%, signal=48% |
| 2372 | GOBP\_MICROTUBULE\_BASED\_TRANSPORT |  | 147 | -0.18 | -0.82 | 0.881 | 0.933 | 1.000 | 2150 | tags=19%, list=17%, signal=23% |
| 2373 | GOBP\_PLATELET\_MORPHOGENESIS |  | 20 | -0.27 | -0.82 | 0.705 | 0.933 | 1.000 | 1944 | tags=25%, list=15%, signal=29% |
| 2374 | GOBP\_MEMORY |  | 99 | -0.19 | -0.82 | 0.829 | 0.935 | 1.000 | 2678 | tags=21%, list=21%, signal=27% |
| 2375 | GOBP\_LOCOMOTORY\_BEHAVIOR |  | 165 | -0.18 | -0.82 | 0.885 | 0.935 | 1.000 | 2757 | tags=22%, list=21%, signal=27% |
| 2376 | GOBP\_POSITIVE\_REGULATION\_OF\_LYMPHOCYTE\_MEDIATED\_IMMUNITY |  | 100 | -0.19 | -0.82 | 0.832 | 0.935 | 1.000 | 2080 | tags=17%, list=16%, signal=20% |
| 2377 | GOBP\_NEGATIVE\_REGULATION\_OF\_ANION\_TRANSPORT |  | 19 | -0.27 | -0.82 | 0.725 | 0.935 | 1.000 | 2230 | tags=26%, list=17%, signal=32% |
| 2378 | GOBP\_REGULATION\_OF\_STEROID\_METABOLIC\_PROCESS |  | 83 | -0.20 | -0.82 | 0.842 | 0.935 | 1.000 | 1622 | tags=16%, list=13%, signal=18% |
| 2379 | GOBP\_DETECTION\_OF\_ABIOTIC\_STIMULUS |  | 107 | -0.19 | -0.82 | 0.838 | 0.936 | 1.000 | 2810 | tags=22%, list=22%, signal=28% |
| 2380 | GOBP\_REGULATION\_OF\_VOLTAGE\_GATED\_CALCIUM\_CHANNEL\_ACTIVITY |  | 31 | -0.24 | -0.82 | 0.774 | 0.936 | 1.000 | 2935 | tags=23%, list=23%, signal=29% |
| 2381 | GOBP\_POSITIVE\_REGULATION\_OF\_MAST\_CELL\_ACTIVATION\_INVOLVED\_IN\_IMMUNE\_RESPONSE |  | 15 | -0.29 | -0.82 | 0.716 | 0.936 | 1.000 | 2000 | tags=27%, list=16%, signal=32% |
| 2382 | GOBP\_CELL\_AGGREGATION |  | 21 | -0.27 | -0.82 | 0.728 | 0.936 | 1.000 | 3330 | tags=33%, list=26%, signal=45% |
| 2383 | GOBP\_POSITIVE\_REGULATION\_OF\_HISTONE\_ACETYLATION |  | 30 | -0.24 | -0.82 | 0.757 | 0.935 | 1.000 | 3496 | tags=40%, list=27%, signal=55% |
| 2384 | GOBP\_ACIDIC\_AMINO\_ACID\_TRANSPORT |  | 56 | -0.21 | -0.82 | 0.789 | 0.937 | 1.000 | 1261 | tags=13%, list=10%, signal=14% |
| 2385 | GOBP\_POSITIVE\_REGULATION\_OF\_STEROID\_METABOLIC\_PROCESS |  | 29 | -0.24 | -0.82 | 0.718 | 0.937 | 1.000 | 1519 | tags=17%, list=12%, signal=19% |
| 2386 | GOBP\_REGULATION\_OF\_DNA\_RECOMBINATION |  | 97 | -0.19 | -0.82 | 0.836 | 0.937 | 1.000 | 1444 | tags=12%, list=11%, signal=14% |
| 2387 | GOBP\_TRANSITION\_METAL\_ION\_HOMEOSTASIS |  | 102 | -0.18 | -0.82 | 0.860 | 0.937 | 1.000 | 4198 | tags=40%, list=33%, signal=59% |
| 2388 | GOBP\_SERINE\_FAMILY\_AMINO\_ACID\_METABOLIC\_PROCESS |  | 35 | -0.23 | -0.82 | 0.762 | 0.937 | 1.000 | 4445 | tags=40%, list=34%, signal=61% |
| 2389 | GOBP\_ACTIVATION\_OF\_ADENYLATE\_CYCLASE\_ACTIVITY |  | 28 | -0.24 | -0.82 | 0.739 | 0.937 | 1.000 | 1390 | tags=18%, list=11%, signal=20% |
| 2390 | GOBP\_SENSORY\_PERCEPTION\_OF\_BITTER\_TASTE |  | 25 | -0.25 | -0.82 | 0.732 | 0.936 | 1.000 | 4091 | tags=36%, list=32%, signal=53% |
| 2391 | GOBP\_REGULATION\_OF\_TRANSMISSION\_OF\_NERVE\_IMPULSE |  | 16 | -0.28 | -0.82 | 0.712 | 0.937 | 1.000 | 5071 | tags=50%, list=39%, signal=82% |
| 2392 | GOBP\_POSITIVE\_REGULATION\_OF\_DENDRITE\_MORPHOGENESIS |  | 30 | -0.24 | -0.82 | 0.749 | 0.937 | 1.000 | 3058 | tags=23%, list=24%, signal=31% |
| 2393 | GOBP\_POSITIVE\_REGULATION\_OF\_PEPTIDASE\_ACTIVITY |  | 166 | -0.17 | -0.82 | 0.896 | 0.937 | 1.000 | 2976 | tags=23%, list=23%, signal=30% |
| 2394 | GOBP\_ENDOCHONDRAL\_BONE\_MORPHOGENESIS |  | 51 | -0.21 | -0.82 | 0.793 | 0.937 | 1.000 | 2881 | tags=27%, list=22%, signal=35% |
| 2395 | GOBP\_CRANIAL\_NERVE\_MORPHOGENESIS |  | 25 | -0.25 | -0.82 | 0.728 | 0.937 | 1.000 | 3046 | tags=28%, list=24%, signal=37% |
| 2396 | GOBP\_POSITIVE\_REGULATION\_OF\_PROTEIN\_LOCALIZATION\_TO\_CELL\_PERIPHERY |  | 53 | -0.21 | -0.82 | 0.799 | 0.937 | 1.000 | 2683 | tags=23%, list=21%, signal=28% |
| 2397 | GOBP\_REGULATION\_OF\_MORPHOGENESIS\_OF\_AN\_EPITHELIUM |  | 54 | -0.21 | -0.81 | 0.784 | 0.938 | 1.000 | 1376 | tags=15%, list=11%, signal=17% |
| 2398 | GOBP\_EAR\_MORPHOGENESIS |  | 91 | -0.19 | -0.81 | 0.819 | 0.938 | 1.000 | 3631 | tags=33%, list=28%, signal=46% |
| 2399 | GOBP\_REGULATION\_OF\_CALCIUM\_ION\_TRANSMEMBRANE\_TRANSPORT |  | 121 | -0.18 | -0.81 | 0.868 | 0.938 | 1.000 | 2982 | tags=24%, list=23%, signal=31% |
| 2400 | GOBP\_SPECIFICATION\_OF\_ANIMAL\_ORGAN\_IDENTITY |  | 31 | -0.24 | -0.81 | 0.771 | 0.939 | 1.000 | 129 | tags=6%, list=1%, signal=7% |
| 2401 | GOBP\_GAMMA\_AMINOBUTYRIC\_ACID\_SIGNALING\_PATHWAY |  | 23 | -0.26 | -0.81 | 0.738 | 0.939 | 1.000 | 2894 | tags=26%, list=22%, signal=34% |
| 2402 | GOBP\_AXIS\_SPECIFICATION |  | 70 | -0.20 | -0.81 | 0.823 | 0.939 | 1.000 | 4434 | tags=40%, list=34%, signal=61% |
| 2403 | GOBP\_STEROL\_TRANSPORT |  | 94 | -0.19 | -0.81 | 0.851 | 0.939 | 1.000 | 4162 | tags=37%, list=32%, signal=55% |
| 2404 | GOBP\_POSITIVE\_REGULATION\_OF\_TUMOR\_NECROSIS\_FACTOR\_SUPERFAMILY\_CYTOKINE\_PRODUCTION |  | 94 | -0.19 | -0.81 | 0.826 | 0.938 | 1.000 | 2319 | tags=21%, list=18%, signal=26% |
| 2405 | GOBP\_REGULATION\_OF\_PROTEIN\_IMPORT |  | 58 | -0.21 | -0.81 | 0.801 | 0.939 | 1.000 | 4329 | tags=47%, list=34%, signal=70% |
| 2406 | GOBP\_MEMBRANE\_REPOLARIZATION |  | 39 | -0.22 | -0.81 | 0.777 | 0.938 | 1.000 | 3951 | tags=36%, list=31%, signal=52% |
| 2407 | GOBP\_MODULATION\_BY\_HOST\_OF\_VIRAL\_GENOME\_REPLICATION |  | 16 | -0.28 | -0.81 | 0.750 | 0.941 | 1.000 | 4048 | tags=50%, list=31%, signal=73% |
| 2408 | GOBP\_ENDOCARDIAL\_CUSHION\_FORMATION |  | 28 | -0.24 | -0.81 | 0.750 | 0.941 | 1.000 | 1174 | tags=14%, list=9%, signal=16% |
| 2409 | GOBP\_POSITIVE\_REGULATION\_OF\_INTERLEUKIN\_1\_BETA\_PRODUCTION |  | 53 | -0.21 | -0.81 | 0.808 | 0.942 | 1.000 | 2539 | tags=23%, list=20%, signal=28% |
| 2410 | GOBP\_VIRAL\_RNA\_GENOME\_REPLICATION |  | 33 | -0.23 | -0.81 | 0.766 | 0.942 | 1.000 | 3088 | tags=30%, list=24%, signal=40% |
| 2411 | GOBP\_PYRIMIDINE\_CONTAINING\_COMPOUND\_CATABOLIC\_PROCESS |  | 34 | -0.23 | -0.81 | 0.782 | 0.942 | 1.000 | 2352 | tags=24%, list=18%, signal=29% |
| 2412 | GOBP\_FC\_EPSILON\_RECEPTOR\_SIGNALING\_PATHWAY |  | 24 | -0.25 | -0.81 | 0.743 | 0.943 | 1.000 | 1489 | tags=21%, list=12%, signal=24% |
| 2413 | GOBP\_SYNAPTIC\_TRANSMISSION\_GABAERGIC |  | 45 | -0.21 | -0.81 | 0.799 | 0.943 | 1.000 | 2358 | tags=20%, list=18%, signal=24% |
| 2414 | GOBP\_REGULATION\_OF\_MICROTUBULE\_BASED\_PROCESS |  | 196 | -0.17 | -0.81 | 0.898 | 0.943 | 1.000 | 4349 | tags=38%, list=34%, signal=56% |
| 2415 | GOBP\_IRON\_ION\_TRANSMEMBRANE\_TRANSPORT |  | 16 | -0.28 | -0.81 | 0.721 | 0.942 | 1.000 | 2572 | tags=31%, list=20%, signal=39% |
| 2416 | GOBP\_POSITIVE\_REGULATION\_OF\_LEUKOCYTE\_MEDIATED\_IMMUNITY |  | 120 | -0.18 | -0.81 | 0.856 | 0.943 | 1.000 | 797 | tags=10%, list=6%, signal=11% |
| 2417 | GOBP\_MICROTUBULE\_POLYMERIZATION |  | 73 | -0.19 | -0.81 | 0.824 | 0.943 | 1.000 | 4339 | tags=38%, list=34%, signal=57% |
| 2418 | GOBP\_PROTEIN\_COMPLEX\_OLIGOMERIZATION |  | 188 | -0.17 | -0.81 | 0.897 | 0.943 | 1.000 | 2682 | tags=21%, list=21%, signal=26% |
| 2419 | GOBP\_POSITIVE\_REGULATION\_OF\_CELL\_KILLING |  | 59 | -0.20 | -0.81 | 0.817 | 0.943 | 1.000 | 2833 | tags=22%, list=22%, signal=28% |
| 2420 | GOBP\_BARBED\_END\_ACTIN\_FILAMENT\_CAPPING |  | 21 | -0.26 | -0.81 | 0.760 | 0.943 | 1.000 | 3663 | tags=29%, list=28%, signal=40% |
| 2421 | GOBP\_POSITIVE\_REGULATION\_OF\_SYNAPTIC\_TRANSMISSION |  | 128 | -0.18 | -0.81 | 0.888 | 0.944 | 1.000 | 4352 | tags=38%, list=34%, signal=57% |
| 2422 | GOBP\_LEUKOCYTE\_PROLIFERATION |  | 282 | -0.16 | -0.81 | 0.951 | 0.943 | 1.000 | 2513 | tags=19%, list=19%, signal=23% |
| 2423 | GOBP\_REGULATION\_OF\_TELOMERE\_MAINTENANCE\_VIA\_TELOMERE\_LENGTHENING |  | 49 | -0.21 | -0.81 | 0.789 | 0.943 | 1.000 | 2311 | tags=18%, list=18%, signal=22% |
| 2424 | GOBP\_RECEPTOR\_RECYCLING |  | 26 | -0.25 | -0.81 | 0.761 | 0.944 | 1.000 | 1430 | tags=19%, list=11%, signal=22% |
| 2425 | GOBP\_SUBSTRATE\_ADHESION\_DEPENDENT\_CELL\_SPREADING |  | 97 | -0.19 | -0.81 | 0.838 | 0.944 | 1.000 | 801 | tags=9%, list=6%, signal=10% |
| 2426 | GOBP\_CELLULAR\_RESPONSE\_TO\_GROWTH\_HORMONE\_STIMULUS |  | 22 | -0.26 | -0.81 | 0.761 | 0.944 | 1.000 | 3100 | tags=27%, list=24%, signal=36% |
| 2427 | GOBP\_POSITIVE\_REGULATION\_OF\_LYMPHOCYTE\_DIFFERENTIATION |  | 97 | -0.18 | -0.81 | 0.850 | 0.944 | 1.000 | 4363 | tags=35%, list=34%, signal=53% |
| 2428 | GOBP\_HOMEOSTASIS\_OF\_NUMBER\_OF\_CELLS\_WITHIN\_A\_TISSUE |  | 25 | -0.25 | -0.81 | 0.738 | 0.944 | 1.000 | 3579 | tags=36%, list=28%, signal=50% |
| 2429 | GOBP\_RESPONSE\_TO\_FATTY\_ACID |  | 57 | -0.20 | -0.81 | 0.813 | 0.943 | 1.000 | 1626 | tags=16%, list=13%, signal=18% |
| 2430 | GOBP\_HISTONE\_H3\_K9\_ACETYLATION |  | 15 | -0.29 | -0.80 | 0.741 | 0.943 | 1.000 | 4485 | tags=53%, list=35%, signal=82% |
| 2431 | GOBP\_AXONEME\_ASSEMBLY |  | 42 | -0.22 | -0.80 | 0.788 | 0.943 | 1.000 | 3008 | tags=29%, list=23%, signal=37% |
| 2432 | GOBP\_CELL\_FATE\_SPECIFICATION |  | 74 | -0.20 | -0.80 | 0.827 | 0.943 | 1.000 | 2059 | tags=18%, list=16%, signal=21% |
| 2433 | GOBP\_PYRIMIDINE\_NUCLEOTIDE\_CATABOLIC\_PROCESS |  | 18 | -0.27 | -0.80 | 0.730 | 0.943 | 1.000 | 2174 | tags=28%, list=17%, signal=33% |
| 2434 | GOBP\_NEGATIVE\_REGULATION\_OF\_PROTEIN\_DEPHOSPHORYLATION |  | 28 | -0.24 | -0.80 | 0.763 | 0.943 | 1.000 | 3394 | tags=36%, list=26%, signal=48% |
| 2435 | GOBP\_SIGNAL\_RELEASE |  | 382 | -0.16 | -0.80 | 0.974 | 0.943 | 1.000 | 3895 | tags=30%, list=30%, signal=42% |
| 2436 | GOBP\_REGULATION\_OF\_RESPONSE\_TO\_REACTIVE\_OXYGEN\_SPECIES |  | 28 | -0.24 | -0.80 | 0.777 | 0.944 | 1.000 | 4136 | tags=43%, list=32%, signal=63% |
| 2437 | GOBP\_CHROMOSOME\_SEGREGATION |  | 254 | -0.16 | -0.80 | 0.949 | 0.944 | 1.000 | 3290 | tags=23%, list=26%, signal=30% |
| 2438 | GOBP\_NEGATIVE\_REGULATION\_OF\_GLYCOPROTEIN\_METABOLIC\_PROCESS |  | 15 | -0.28 | -0.80 | 0.722 | 0.944 | 1.000 | 4046 | tags=53%, list=31%, signal=78% |
| 2439 | GOBP\_NEURAL\_RETINA\_DEVELOPMENT |  | 53 | -0.20 | -0.80 | 0.803 | 0.944 | 1.000 | 3734 | tags=30%, list=29%, signal=42% |
| 2440 | GOBP\_CALCINEURIN\_MEDIATED\_SIGNALING |  | 41 | -0.22 | -0.80 | 0.794 | 0.945 | 1.000 | 3860 | tags=32%, list=30%, signal=45% |
| 2441 | GOBP\_SERTOLI\_CELL\_DIFFERENTIATION |  | 16 | -0.27 | -0.80 | 0.741 | 0.944 | 1.000 | 2761 | tags=25%, list=21%, signal=32% |
| 2442 | GOBP\_MONOCYTE\_CHEMOTACTIC\_PROTEIN\_1\_PRODUCTION |  | 18 | -0.27 | -0.80 | 0.748 | 0.944 | 1.000 | 1206 | tags=17%, list=9%, signal=18% |
| 2443 | GOBP\_PROSTAGLANDIN\_TRANSPORT |  | 19 | -0.26 | -0.80 | 0.721 | 0.944 | 1.000 | 2356 | tags=26%, list=18%, signal=32% |
| 2444 | GOBP\_TYPE\_2\_IMMUNE\_RESPONSE |  | 35 | -0.23 | -0.80 | 0.790 | 0.944 | 1.000 | 2819 | tags=29%, list=22%, signal=36% |
| 2445 | GOBP\_HEMOSTASIS |  | 206 | -0.17 | -0.80 | 0.921 | 0.945 | 1.000 | 4602 | tags=38%, list=36%, signal=58% |
| 2446 | GOBP\_REGULATION\_OF\_OSTEOBLAST\_PROLIFERATION |  | 22 | -0.25 | -0.80 | 0.761 | 0.944 | 1.000 | 2220 | tags=27%, list=17%, signal=33% |
| 2447 | GOBP\_POSITIVE\_REGULATION\_OF\_CELL\_CYCLE\_G2\_M\_PHASE\_TRANSITION |  | 26 | -0.24 | -0.80 | 0.776 | 0.945 | 1.000 | 523 | tags=12%, list=4%, signal=12% |
| 2448 | GOBP\_CALCIUM\_ION\_TRANSMEMBRANE\_TRANSPORT |  | 244 | -0.16 | -0.80 | 0.944 | 0.945 | 1.000 | 3081 | tags=23%, list=24%, signal=30% |
| 2449 | GOBP\_POSITIVE\_REGULATION\_OF\_DEPHOSPHORYLATION |  | 53 | -0.21 | -0.80 | 0.821 | 0.946 | 1.000 | 2956 | tags=25%, list=23%, signal=32% |
| 2450 | GOBP\_MYD88\_DEPENDENT\_TOLL\_LIKE\_RECEPTOR\_SIGNALING\_PATHWAY |  | 18 | -0.27 | -0.80 | 0.745 | 0.946 | 1.000 | 2202 | tags=28%, list=17%, signal=33% |
| 2451 | GOBP\_MECHANORECEPTOR\_DIFFERENTIATION |  | 44 | -0.22 | -0.80 | 0.778 | 0.946 | 1.000 | 2702 | tags=23%, list=21%, signal=29% |
| 2452 | GOBP\_REGULATION\_OF\_HISTONE\_H3\_K9\_METHYLATION |  | 23 | -0.25 | -0.80 | 0.774 | 0.946 | 1.000 | 4638 | tags=52%, list=36%, signal=81% |
| 2453 | GOBP\_POSITIVE\_REGULATION\_OF\_EXOSOMAL\_SECRETION |  | 15 | -0.28 | -0.80 | 0.722 | 0.946 | 1.000 | 2587 | tags=27%, list=20%, signal=33% |
| 2454 | GOBP\_RESPONSE\_TO\_FUNGUS |  | 48 | -0.21 | -0.80 | 0.793 | 0.946 | 1.000 | 3890 | tags=40%, list=30%, signal=56% |
| 2455 | GOBP\_OSTEOBLAST\_DEVELOPMENT |  | 18 | -0.27 | -0.80 | 0.755 | 0.948 | 1.000 | 3961 | tags=44%, list=31%, signal=64% |
| 2456 | GOBP\_PURINE\_NUCLEOTIDE\_TRANSPORT |  | 20 | -0.26 | -0.80 | 0.761 | 0.948 | 1.000 | 1526 | tags=20%, list=12%, signal=23% |
| 2457 | GOBP\_PROTEIN\_O\_LINKED\_GLYCOSYLATION |  | 61 | -0.20 | -0.80 | 0.835 | 0.948 | 1.000 | 4032 | tags=39%, list=31%, signal=57% |
| 2458 | GOBP\_POSITIVE\_REGULATION\_OF\_DNA\_RECOMBINATION |  | 55 | -0.20 | -0.80 | 0.818 | 0.948 | 1.000 | 2718 | tags=20%, list=21%, signal=25% |
| 2459 | GOBP\_DETECTION\_OF\_BIOTIC\_STIMULUS |  | 31 | -0.23 | -0.79 | 0.785 | 0.950 | 1.000 | 1961 | tags=23%, list=15%, signal=27% |
| 2460 | GOBP\_NEGATIVE\_REGULATION\_OF\_ORGANELLE\_ASSEMBLY |  | 34 | -0.23 | -0.79 | 0.768 | 0.949 | 1.000 | 4485 | tags=41%, list=35%, signal=63% |
| 2461 | GOBP\_REGULATION\_OF\_ION\_TRANSMEMBRANE\_TRANSPORT |  | 353 | -0.16 | -0.79 | 0.972 | 0.950 | 1.000 | 2982 | tags=23%, list=23%, signal=29% |
| 2462 | GOBP\_CELLULAR\_IRON\_ION\_HOMEOSTASIS |  | 48 | -0.21 | -0.79 | 0.817 | 0.950 | 1.000 | 4198 | tags=48%, list=33%, signal=71% |
| 2463 | GOBP\_RESPONSE\_TO\_CHOLESTEROL |  | 23 | -0.25 | -0.79 | 0.795 | 0.950 | 1.000 | 2791 | tags=30%, list=22%, signal=39% |
| 2464 | GOBP\_AXO\_DENDRITIC\_TRANSPORT |  | 62 | -0.20 | -0.79 | 0.840 | 0.950 | 1.000 | 2150 | tags=19%, list=17%, signal=23% |
| 2465 | GOBP\_EMBRYONIC\_AXIS\_SPECIFICATION |  | 30 | -0.23 | -0.79 | 0.785 | 0.950 | 1.000 | 4065 | tags=33%, list=32%, signal=49% |
| 2466 | GOBP\_PHOTORECEPTOR\_CELL\_DIFFERENTIATION |  | 48 | -0.21 | -0.79 | 0.832 | 0.951 | 1.000 | 3959 | tags=31%, list=31%, signal=45% |
| 2467 | GOBP\_NEGATIVE\_REGULATION\_OF\_LYMPHOCYTE\_MEDIATED\_IMMUNITY |  | 49 | -0.21 | -0.79 | 0.844 | 0.952 | 1.000 | 2225 | tags=18%, list=17%, signal=22% |
| 2468 | GOBP\_NEGATIVE\_REGULATION\_OF\_CALCIUM\_MEDIATED\_SIGNALING |  | 17 | -0.27 | -0.79 | 0.756 | 0.951 | 1.000 | 1962 | tags=18%, list=15%, signal=21% |
| 2469 | GOBP\_BRANCHING\_INVOLVED\_IN\_MAMMARY\_GLAND\_DUCT\_MORPHOGENESIS |  | 22 | -0.25 | -0.79 | 0.793 | 0.953 | 1.000 | 3949 | tags=41%, list=31%, signal=59% |
| 2470 | GOBP\_NEGATIVE\_REGULATION\_OF\_NERVOUS\_SYSTEM\_PROCESS |  | 18 | -0.26 | -0.79 | 0.749 | 0.953 | 1.000 | 1066 | tags=17%, list=8%, signal=18% |
| 2471 | GOBP\_NEGATIVE\_REGULATION\_OF\_GENE\_EXPRESSION\_EPIGENETIC |  | 66 | -0.20 | -0.79 | 0.844 | 0.953 | 1.000 | 1833 | tags=15%, list=14%, signal=18% |
| 2472 | GOBP\_REGULATION\_OF\_CATION\_CHANNEL\_ACTIVITY |  | 122 | -0.18 | -0.79 | 0.912 | 0.953 | 1.000 | 3118 | tags=24%, list=24%, signal=31% |
| 2473 | GOBP\_REGULATION\_OF\_ACTION\_POTENTIAL |  | 47 | -0.21 | -0.79 | 0.812 | 0.954 | 1.000 | 3951 | tags=34%, list=31%, signal=49% |
| 2474 | GOBP\_PHENOL\_CONTAINING\_COMPOUND\_METABOLIC\_PROCESS |  | 100 | -0.18 | -0.79 | 0.904 | 0.954 | 1.000 | 2017 | tags=18%, list=16%, signal=21% |
| 2475 | GOBP\_POSITIVE\_REGULATION\_OF\_PROTEIN\_DEPHOSPHORYLATION |  | 40 | -0.22 | -0.79 | 0.826 | 0.953 | 1.000 | 2956 | tags=28%, list=23%, signal=36% |
| 2476 | GOBP\_PROTEIN\_PROCESSING |  | 194 | -0.16 | -0.79 | 0.948 | 0.954 | 1.000 | 4505 | tags=40%, list=35%, signal=60% |
| 2477 | GOBP\_LONG\_CHAIN\_FATTY\_ACID\_TRANSPORT |  | 56 | -0.20 | -0.79 | 0.831 | 0.954 | 1.000 | 2750 | tags=23%, list=21%, signal=29% |
| 2478 | GOBP\_REGULATION\_OF\_ACTIN\_FILAMENT\_LENGTH |  | 124 | -0.17 | -0.79 | 0.887 | 0.954 | 1.000 | 3773 | tags=31%, list=29%, signal=44% |
| 2479 | GOBP\_BRANCH\_ELONGATION\_OF\_AN\_EPITHELIUM |  | 15 | -0.28 | -0.79 | 0.746 | 0.954 | 1.000 | 1756 | tags=20%, list=14%, signal=23% |
| 2480 | GOBP\_SPECIFICATION\_OF\_SYMMETRY |  | 92 | -0.19 | -0.79 | 0.872 | 0.954 | 1.000 | 3577 | tags=29%, list=28%, signal=40% |
| 2481 | GOBP\_XENOBIOTIC\_TRANSPORT |  | 33 | -0.22 | -0.79 | 0.812 | 0.955 | 1.000 | 3930 | tags=36%, list=30%, signal=52% |
| 2482 | GOBP\_REGULATION\_OF\_PROTEIN\_POLYMERIZATION |  | 161 | -0.17 | -0.79 | 0.918 | 0.955 | 1.000 | 3821 | tags=31%, list=30%, signal=44% |
| 2483 | GOBP\_TELOMERE\_MAINTENANCE\_VIA\_TELOMERE\_LENGTHENING |  | 66 | -0.19 | -0.79 | 0.869 | 0.955 | 1.000 | 2311 | tags=18%, list=18%, signal=22% |
| 2484 | GOBP\_DETECTION\_OF\_TEMPERATURE\_STIMULUS |  | 22 | -0.25 | -0.78 | 0.777 | 0.957 | 1.000 | 1229 | tags=14%, list=10%, signal=15% |
| 2485 | GOBP\_REGULATION\_OF\_LYMPHOCYTE\_MEDIATED\_IMMUNITY |  | 148 | -0.17 | -0.78 | 0.930 | 0.957 | 1.000 | 2290 | tags=18%, list=18%, signal=21% |
| 2486 | GOBP\_PROTEIN\_MATURATION |  | 248 | -0.16 | -0.78 | 0.963 | 0.957 | 1.000 | 4505 | tags=39%, list=35%, signal=59% |
| 2487 | GOBP\_REGULATION\_OF\_FATTY\_ACID\_BIOSYNTHETIC\_PROCESS |  | 39 | -0.22 | -0.78 | 0.821 | 0.957 | 1.000 | 2078 | tags=18%, list=16%, signal=21% |
| 2488 | GOBP\_DETECTION\_OF\_STIMULUS\_INVOLVED\_IN\_SENSORY\_PERCEPTION\_OF\_PAIN |  | 24 | -0.24 | -0.78 | 0.770 | 0.957 | 1.000 | 1229 | tags=13%, list=10%, signal=14% |
| 2489 | GOBP\_POSITIVE\_REGULATION\_OF\_POTASSIUM\_ION\_TRANSPORT |  | 36 | -0.22 | -0.78 | 0.792 | 0.957 | 1.000 | 2982 | tags=31%, list=23%, signal=40% |
| 2490 | GOBP\_NEGATIVE\_REGULATION\_OF\_B\_CELL\_ACTIVATION |  | 32 | -0.23 | -0.78 | 0.784 | 0.957 | 1.000 | 1665 | tags=19%, list=13%, signal=21% |
| 2491 | GOBP\_CELLULAR\_RESPONSE\_TO\_DSRNA |  | 19 | -0.26 | -0.78 | 0.779 | 0.957 | 1.000 | 2275 | tags=26%, list=18%, signal=32% |
| 2492 | GOBP\_NEGATIVE\_REGULATION\_OF\_COLD\_INDUCED\_THERMOGENESIS |  | 43 | -0.21 | -0.78 | 0.837 | 0.957 | 1.000 | 2080 | tags=21%, list=16%, signal=25% |
| 2493 | GOBP\_ATTACHMENT\_OF\_SPINDLE\_MICROTUBULES\_TO\_KINETOCHORE |  | 29 | -0.23 | -0.78 | 0.810 | 0.957 | 1.000 | 4258 | tags=34%, list=33%, signal=51% |
| 2494 | GOBP\_REGULATION\_OF\_GRANULOCYTE\_CHEMOTAXIS |  | 46 | -0.21 | -0.78 | 0.824 | 0.957 | 1.000 | 2204 | tags=20%, list=17%, signal=24% |
| 2495 | GOBP\_NEGATIVE\_REGULATION\_OF\_TELOMERE\_MAINTENANCE |  | 28 | -0.23 | -0.78 | 0.797 | 0.957 | 1.000 | 934 | tags=11%, list=7%, signal=12% |
| 2496 | GOBP\_POSITIVE\_REGULATION\_OF\_CILIUM\_ASSEMBLY |  | 22 | -0.25 | -0.78 | 0.775 | 0.958 | 1.000 | 3566 | tags=36%, list=28%, signal=50% |
| 2497 | GOBP\_POSITIVE\_REGULATION\_OF\_PROTEIN\_BINDING |  | 77 | -0.19 | -0.78 | 0.870 | 0.958 | 1.000 | 2246 | tags=22%, list=17%, signal=27% |
| 2498 | GOBP\_HISTONE\_H2A\_UBIQUITINATION |  | 21 | -0.26 | -0.78 | 0.799 | 0.957 | 1.000 | 1263 | tags=14%, list=10%, signal=16% |
| 2499 | GOBP\_CALCIUM\_ION\_TRANSPORT\_INTO\_CYTOSOL |  | 140 | -0.17 | -0.78 | 0.936 | 0.957 | 1.000 | 3081 | tags=23%, list=24%, signal=30% |
| 2500 | GOBP\_POSITIVE\_REGULATION\_OF\_INTERLEUKIN\_10\_PRODUCTION |  | 35 | -0.22 | -0.78 | 0.818 | 0.957 | 1.000 | 1475 | tags=17%, list=11%, signal=19% |
| 2501 | GOBP\_REGULATION\_OF\_MEIOTIC\_NUCLEAR\_DIVISION |  | 22 | -0.25 | -0.78 | 0.786 | 0.957 | 1.000 | 2104 | tags=27%, list=16%, signal=33% |
| 2502 | GOBP\_POSITIVE\_REGULATION\_OF\_LYMPHOCYTE\_MIGRATION |  | 32 | -0.23 | -0.78 | 0.806 | 0.958 | 1.000 | 4027 | tags=44%, list=31%, signal=63% |
| 2503 | GOBP\_RIBONUCLEOSIDE\_MONOPHOSPHATE\_BIOSYNTHETIC\_PROCESS |  | 30 | -0.23 | -0.78 | 0.808 | 0.958 | 1.000 | 3427 | tags=33%, list=27%, signal=45% |
| 2504 | GOBP\_POSITIVE\_REGULATION\_OF\_EXOCYTOSIS |  | 72 | -0.19 | -0.78 | 0.867 | 0.958 | 1.000 | 2134 | tags=18%, list=17%, signal=22% |
| 2505 | GOBP\_MICROTUBULE\_BASED\_MOVEMENT |  | 252 | -0.16 | -0.78 | 0.971 | 0.958 | 1.000 | 2061 | tags=17%, list=16%, signal=19% |
| 2506 | GOBP\_REGULATION\_OF\_AMYLOID\_PRECURSOR\_PROTEIN\_CATABOLIC\_PROCESS |  | 39 | -0.22 | -0.78 | 0.823 | 0.958 | 1.000 | 1381 | tags=15%, list=11%, signal=17% |
| 2507 | GOBP\_MORPHOGENESIS\_OF\_A\_POLARIZED\_EPITHELIUM |  | 74 | -0.18 | -0.78 | 0.875 | 0.957 | 1.000 | 736 | tags=9%, list=6%, signal=10% |
| 2508 | GOBP\_CELLULAR\_RESPONSE\_TO\_ZINC\_ION |  | 16 | -0.27 | -0.78 | 0.785 | 0.958 | 1.000 | 3393 | tags=38%, list=26%, signal=51% |
| 2509 | GOBP\_NEGATIVE\_REGULATION\_OF\_EPITHELIAL\_CELL\_PROLIFERATION |  | 112 | -0.18 | -0.78 | 0.906 | 0.958 | 1.000 | 2604 | tags=19%, list=20%, signal=23% |
| 2510 | GOBP\_CELL\_KILLING |  | 154 | -0.17 | -0.78 | 0.938 | 0.958 | 1.000 | 2842 | tags=21%, list=22%, signal=27% |
| 2511 | GOBP\_T\_CELL\_MEDIATED\_CYTOTOXICITY |  | 47 | -0.20 | -0.78 | 0.831 | 0.958 | 1.000 | 400 | tags=9%, list=3%, signal=9% |
| 2512 | GOBP\_PROTEIN\_LOCALIZATION\_TO\_EXTRACELLULAR\_REGION |  | 286 | -0.16 | -0.78 | 0.981 | 0.957 | 1.000 | 3832 | tags=29%, list=30%, signal=40% |
| 2513 | GOBP\_REGULATION\_OF\_CALCIUM\_ION\_DEPENDENT\_EXOCYTOSIS |  | 31 | -0.22 | -0.78 | 0.833 | 0.958 | 1.000 | 4602 | tags=39%, list=36%, signal=60% |
| 2514 | GOBP\_STEM\_CELL\_DIVISION |  | 22 | -0.24 | -0.77 | 0.793 | 0.958 | 1.000 | 2656 | tags=32%, list=21%, signal=40% |
| 2515 | GOBP\_RIBONUCLEOTIDE\_CATABOLIC\_PROCESS |  | 36 | -0.22 | -0.77 | 0.814 | 0.958 | 1.000 | 4105 | tags=47%, list=32%, signal=69% |
| 2516 | GOBP\_REGULATION\_OF\_LEUKOCYTE\_DEGRANULATION |  | 46 | -0.20 | -0.77 | 0.862 | 0.959 | 1.000 | 4271 | tags=39%, list=33%, signal=58% |
| 2517 | GOBP\_DENDRITE\_EXTENSION |  | 26 | -0.24 | -0.77 | 0.806 | 0.960 | 1.000 | 1571 | tags=19%, list=12%, signal=22% |
| 2518 | GOBP\_HEPARAN\_SULFATE\_PROTEOGLYCAN\_BIOSYNTHETIC\_PROCESS |  | 21 | -0.25 | -0.77 | 0.802 | 0.960 | 1.000 | 1661 | tags=19%, list=13%, signal=22% |
| 2519 | GOBP\_MYELOID\_DENDRITIC\_CELL\_ACTIVATION |  | 25 | -0.24 | -0.77 | 0.804 | 0.959 | 1.000 | 4159 | tags=52%, list=32%, signal=77% |
| 2520 | GOBP\_EMBRYONIC\_HEART\_TUBE\_MORPHOGENESIS |  | 50 | -0.21 | -0.77 | 0.821 | 0.959 | 1.000 | 3027 | tags=26%, list=23%, signal=34% |
| 2521 | GOBP\_NATURAL\_KILLER\_CELL\_ACTIVATION\_INVOLVED\_IN\_IMMUNE\_RESPONSE |  | 22 | -0.24 | -0.77 | 0.802 | 0.959 | 1.000 | 4369 | tags=41%, list=34%, signal=62% |
| 2522 | GOBP\_NEUTROPHIL\_MEDIATED\_IMMUNITY |  | 33 | -0.22 | -0.77 | 0.825 | 0.959 | 1.000 | 4700 | tags=52%, list=36%, signal=81% |
| 2523 | GOBP\_POLYSACCHARIDE\_METABOLIC\_PROCESS |  | 89 | -0.18 | -0.77 | 0.888 | 0.962 | 1.000 | 2072 | tags=17%, list=16%, signal=20% |
| 2524 | GOBP\_REGULATION\_OF\_COAGULATION |  | 65 | -0.19 | -0.77 | 0.874 | 0.961 | 1.000 | 3219 | tags=28%, list=25%, signal=37% |
| 2525 | GOBP\_NEGATIVE\_REGULATION\_OF\_B\_CELL\_MEDIATED\_IMMUNITY |  | 15 | -0.27 | -0.77 | 0.799 | 0.961 | 1.000 | 1526 | tags=20%, list=12%, signal=23% |
| 2526 | GOBP\_GRANULOCYTE\_DIFFERENTIATION |  | 30 | -0.22 | -0.77 | 0.830 | 0.961 | 1.000 | 1944 | tags=20%, list=15%, signal=23% |
| 2527 | GOBP\_CARDIAC\_VENTRICLE\_DEVELOPMENT |  | 108 | -0.18 | -0.77 | 0.889 | 0.961 | 1.000 | 3027 | tags=21%, list=23%, signal=28% |
| 2528 | GOBP\_NEGATIVE\_REGULATION\_OF\_TUMOR\_NECROSIS\_FACTOR\_SUPERFAMILY\_CYTOKINE\_PRODUCTION |  | 55 | -0.20 | -0.77 | 0.863 | 0.961 | 1.000 | 1892 | tags=16%, list=15%, signal=19% |
| 2529 | GOBP\_DEFENSE\_RESPONSE\_TO\_BACTERIUM |  | 218 | -0.16 | -0.77 | 0.957 | 0.962 | 1.000 | 4102 | tags=33%, list=32%, signal=48% |
| 2530 | GOBP\_POSITIVE\_REGULATION\_OF\_PHOSPHOPROTEIN\_PHOSPHATASE\_ACTIVITY |  | 19 | -0.25 | -0.77 | 0.790 | 0.962 | 1.000 | 3252 | tags=37%, list=25%, signal=49% |
| 2531 | GOBP\_REGULATION\_OF\_SYNAPSE\_STRUCTURE\_OR\_ACTIVITY |  | 165 | -0.16 | -0.77 | 0.929 | 0.962 | 1.000 | 3118 | tags=24%, list=24%, signal=31% |
| 2532 | GOBP\_FEAR\_RESPONSE |  | 36 | -0.22 | -0.77 | 0.853 | 0.962 | 1.000 | 3193 | tags=28%, list=25%, signal=37% |
| 2533 | GOBP\_REGULATION\_OF\_BODY\_FLUID\_LEVELS |  | 339 | -0.15 | -0.77 | 0.986 | 0.962 | 1.000 | 4603 | tags=37%, list=36%, signal=56% |
| 2534 | GOBP\_FOREBRAIN\_GENERATION\_OF\_NEURONS |  | 40 | -0.21 | -0.77 | 0.850 | 0.962 | 1.000 | 1907 | tags=18%, list=15%, signal=20% |
| 2535 | GOBP\_REGULATION\_OF\_MEMBRANE\_POTENTIAL |  | 342 | -0.15 | -0.77 | 0.981 | 0.962 | 1.000 | 3035 | tags=22%, list=24%, signal=28% |
| 2536 | GOBP\_ACYLGLYCEROL\_HOMEOSTASIS |  | 27 | -0.23 | -0.77 | 0.795 | 0.961 | 1.000 | 1513 | tags=15%, list=12%, signal=17% |
| 2537 | GOBP\_BONE\_DEVELOPMENT |  | 183 | -0.16 | -0.77 | 0.969 | 0.962 | 1.000 | 3599 | tags=31%, list=28%, signal=42% |
| 2538 | GOBP\_FATTY\_ACID\_TRANSPORT |  | 80 | -0.18 | -0.76 | 0.898 | 0.963 | 1.000 | 2750 | tags=23%, list=21%, signal=28% |
| 2539 | GOBP\_NEGATIVE\_REGULATION\_OF\_TELOMERE\_MAINTENANCE\_VIA\_TELOMERE\_LENGTHENING |  | 20 | -0.25 | -0.76 | 0.811 | 0.963 | 1.000 | 546 | tags=10%, list=4%, signal=10% |
| 2540 | GOBP\_FOREBRAIN\_NEURON\_DIFFERENTIATION |  | 33 | -0.22 | -0.76 | 0.841 | 0.963 | 1.000 | 1861 | tags=18%, list=14%, signal=21% |
| 2541 | GOBP\_NEGATIVE\_REGULATION\_OF\_SUPRAMOLECULAR\_FIBER\_ORGANIZATION |  | 135 | -0.17 | -0.76 | 0.939 | 0.962 | 1.000 | 2650 | tags=22%, list=21%, signal=28% |
| 2542 | GOBP\_NEGATIVE\_REGULATION\_OF\_DEPHOSPHORYLATION |  | 37 | -0.21 | -0.76 | 0.832 | 0.962 | 1.000 | 5122 | tags=54%, list=40%, signal=89% |
| 2543 | GOBP\_CELLULAR\_RESPONSE\_TO\_VITAMIN\_D |  | 21 | -0.25 | -0.76 | 0.805 | 0.962 | 1.000 | 3320 | tags=33%, list=26%, signal=45% |
| 2544 | GOBP\_NEGATIVE\_REGULATION\_OF\_IMMUNE\_EFFECTOR\_PROCESS |  | 99 | -0.17 | -0.76 | 0.913 | 0.962 | 1.000 | 4047 | tags=34%, list=31%, signal=50% |
| 2545 | GOBP\_NUCLEOTIDE\_TRANSPORT |  | 23 | -0.24 | -0.76 | 0.820 | 0.962 | 1.000 | 1526 | tags=17%, list=12%, signal=20% |
| 2546 | GOBP\_CHROMATIN\_ASSEMBLY\_OR\_DISASSEMBLY |  | 175 | -0.16 | -0.76 | 0.954 | 0.962 | 1.000 | 1833 | tags=13%, list=14%, signal=15% |
| 2547 | GOBP\_SYNAPTIC\_TRANSMISSION\_GLUTAMATERGIC |  | 75 | -0.19 | -0.76 | 0.896 | 0.962 | 1.000 | 3118 | tags=27%, list=24%, signal=35% |
| 2548 | GOBP\_MITOCHONDRIAL\_OUTER\_MEMBRANE\_PERMEABILIZATION\_INVOLVED\_IN\_PROGRAMMED\_CELL\_DEATH |  | 31 | -0.22 | -0.76 | 0.840 | 0.962 | 1.000 | 1745 | tags=19%, list=14%, signal=22% |
| 2549 | GOBP\_POSITIVE\_REGULATION\_OF\_IMMUNE\_EFFECTOR\_PROCESS |  | 217 | -0.16 | -0.76 | 0.966 | 0.962 | 1.000 | 2080 | tags=16%, list=16%, signal=18% |
| 2550 | GOBP\_INTERLEUKIN\_12\_PRODUCTION |  | 56 | -0.20 | -0.76 | 0.873 | 0.962 | 1.000 | 2202 | tags=18%, list=17%, signal=21% |
| 2551 | GOBP\_POSITIVE\_REGULATION\_OF\_PHOSPHATASE\_ACTIVITY |  | 31 | -0.22 | -0.76 | 0.838 | 0.963 | 1.000 | 3252 | tags=29%, list=25%, signal=39% |
| 2552 | GOBP\_MEMBRANE\_DEPOLARIZATION |  | 72 | -0.18 | -0.76 | 0.889 | 0.963 | 1.000 | 4368 | tags=36%, list=34%, signal=54% |
| 2553 | GOBP\_REPLICATIVE\_SENESCENCE |  | 15 | -0.27 | -0.76 | 0.809 | 0.963 | 1.000 | 4094 | tags=40%, list=32%, signal=59% |
| 2554 | GOBP\_POSITIVE\_REGULATION\_OF\_PROTEIN\_IMPORT |  | 40 | -0.21 | -0.76 | 0.846 | 0.963 | 1.000 | 4329 | tags=48%, list=34%, signal=71% |
| 2555 | GOBP\_NEGATIVE\_REGULATION\_OF\_CARTILAGE\_DEVELOPMENT |  | 23 | -0.24 | -0.76 | 0.821 | 0.962 | 1.000 | 436 | tags=9%, list=3%, signal=9% |
| 2556 | GOBP\_EOSINOPHIL\_MIGRATION |  | 21 | -0.24 | -0.76 | 0.788 | 0.963 | 1.000 | 4756 | tags=52%, list=37%, signal=83% |
| 2557 | GOBP\_RESPONSE\_TO\_LITHIUM\_ION |  | 19 | -0.25 | -0.76 | 0.795 | 0.963 | 1.000 | 1539 | tags=21%, list=12%, signal=24% |
| 2558 | GOBP\_REGULATION\_OF\_HEART\_RATE |  | 92 | -0.18 | -0.76 | 0.922 | 0.963 | 1.000 | 3035 | tags=22%, list=24%, signal=28% |
| 2559 | GOBP\_PROTEIN\_POLYMERIZATION |  | 224 | -0.15 | -0.76 | 0.972 | 0.963 | 1.000 | 4219 | tags=34%, list=33%, signal=50% |
| 2560 | GOBP\_REGULATION\_OF\_ATP\_BIOSYNTHETIC\_PROCESS |  | 17 | -0.26 | -0.76 | 0.796 | 0.962 | 1.000 | 3296 | tags=41%, list=26%, signal=55% |
| 2561 | GOBP\_TOOTH\_MINERALIZATION |  | 19 | -0.25 | -0.76 | 0.823 | 0.963 | 1.000 | 2131 | tags=26%, list=17%, signal=31% |
| 2562 | GOBP\_LYMPHOCYTE\_HOMEOSTASIS |  | 52 | -0.20 | -0.76 | 0.865 | 0.964 | 1.000 | 3821 | tags=33%, list=30%, signal=46% |
| 2563 | GOBP\_MITOTIC\_NUCLEAR\_DIVISION |  | 242 | -0.15 | -0.76 | 0.982 | 0.964 | 1.000 | 3055 | tags=20%, list=24%, signal=26% |
| 2564 | GOBP\_REGULATION\_OF\_ANION\_TRANSMEMBRANE\_TRANSPORT |  | 20 | -0.25 | -0.76 | 0.804 | 0.964 | 1.000 | 949 | tags=15%, list=7%, signal=16% |
| 2565 | GOBP\_ASTROCYTE\_ACTIVATION |  | 20 | -0.25 | -0.76 | 0.791 | 0.965 | 1.000 | 3362 | tags=35%, list=26%, signal=47% |
| 2566 | GOBP\_PROTEIN\_LOCALIZATION\_TO\_SYNAPSE |  | 55 | -0.19 | -0.75 | 0.886 | 0.965 | 1.000 | 2523 | tags=22%, list=20%, signal=27% |
| 2567 | GOBP\_REGULATION\_OF\_REGULATED\_SECRETORY\_PATHWAY |  | 119 | -0.17 | -0.75 | 0.938 | 0.965 | 1.000 | 3680 | tags=29%, list=29%, signal=40% |
| 2568 | GOBP\_HISTONE\_H3\_K9\_TRIMETHYLATION |  | 15 | -0.26 | -0.75 | 0.798 | 0.967 | 1.000 | 5351 | tags=67%, list=41%, signal=114% |
| 2569 | GOBP\_RELAXATION\_OF\_MUSCLE |  | 29 | -0.22 | -0.75 | 0.837 | 0.967 | 1.000 | 4684 | tags=52%, list=36%, signal=81% |
| 2570 | GOBP\_POSITIVE\_REGULATION\_OF\_STRESS\_FIBER\_ASSEMBLY |  | 51 | -0.20 | -0.75 | 0.889 | 0.967 | 1.000 | 1522 | tags=16%, list=12%, signal=18% |
| 2571 | GOBP\_ICOSANOID\_TRANSPORT |  | 46 | -0.20 | -0.75 | 0.877 | 0.966 | 1.000 | 2356 | tags=22%, list=18%, signal=27% |
| 2572 | GOBP\_POSITIVE\_REGULATION\_OF\_PROTEIN\_POLYUBIQUITINATION |  | 15 | -0.26 | -0.75 | 0.794 | 0.967 | 1.000 | 114 | tags=7%, list=1%, signal=7% |
| 2573 | GOBP\_POSITIVE\_REGULATION\_OF\_INTERLEUKIN\_1\_PRODUCTION |  | 60 | -0.19 | -0.75 | 0.895 | 0.967 | 1.000 | 3543 | tags=30%, list=27%, signal=41% |
| 2574 | GOBP\_RESPONSE\_TO\_LEAD\_ION |  | 19 | -0.25 | -0.75 | 0.807 | 0.967 | 1.000 | 354 | tags=11%, list=3%, signal=11% |
| 2575 | GOBP\_AMINE\_BIOSYNTHETIC\_PROCESS |  | 38 | -0.21 | -0.75 | 0.845 | 0.968 | 1.000 | 3437 | tags=26%, list=27%, signal=36% |
| 2576 | GOBP\_REGULATION\_OF\_MEMBRANE\_DEPOLARIZATION |  | 36 | -0.21 | -0.75 | 0.859 | 0.968 | 1.000 | 4515 | tags=36%, list=35%, signal=55% |
| 2577 | GOBP\_HETEROCHROMATIN\_ORGANIZATION |  | 66 | -0.18 | -0.75 | 0.924 | 0.968 | 1.000 | 4434 | tags=35%, list=34%, signal=53% |
| 2578 | GOBP\_DENDRITIC\_CELL\_MIGRATION |  | 29 | -0.22 | -0.75 | 0.858 | 0.968 | 1.000 | 1092 | tags=10%, list=8%, signal=11% |
| 2579 | GOBP\_POSITIVE\_REGULATION\_OF\_PEPTIDE\_SECRETION |  | 81 | -0.18 | -0.75 | 0.915 | 0.968 | 1.000 | 3742 | tags=33%, list=29%, signal=47% |
| 2580 | GOBP\_MUCOPOLYSACCHARIDE\_METABOLIC\_PROCESS |  | 73 | -0.18 | -0.75 | 0.885 | 0.968 | 1.000 | 1661 | tags=16%, list=13%, signal=19% |
| 2581 | GOBP\_PROTEIN\_LOCALIZATION\_TO\_CYTOSKELETON |  | 49 | -0.20 | -0.75 | 0.877 | 0.968 | 1.000 | 3618 | tags=29%, list=28%, signal=40% |
| 2582 | GOBP\_POSITIVE\_REGULATION\_OF\_CELL\_MORPHOGENESIS\_INVOLVED\_IN\_DIFFERENTIATION |  | 71 | -0.18 | -0.75 | 0.905 | 0.968 | 1.000 | 4156 | tags=31%, list=32%, signal=45% |
| 2583 | GOBP\_NEGATIVE\_REGULATION\_OF\_PROTEIN\_CONTAINING\_COMPLEX\_ASSEMBLY |  | 119 | -0.17 | -0.75 | 0.952 | 0.968 | 1.000 | 2491 | tags=18%, list=19%, signal=23% |
| 2584 | GOBP\_CHAPERONE\_MEDIATED\_PROTEIN\_FOLDING |  | 61 | -0.19 | -0.75 | 0.898 | 0.968 | 1.000 | 3863 | tags=34%, list=30%, signal=49% |
| 2585 | GOBP\_POSITIVE\_REGULATION\_OF\_NATURAL\_KILLER\_CELL\_MEDIATED\_IMMUNITY |  | 25 | -0.23 | -0.75 | 0.854 | 0.969 | 1.000 | 2033 | tags=20%, list=16%, signal=24% |
| 2586 | GOBP\_ADP\_METABOLIC\_PROCESS |  | 82 | -0.18 | -0.74 | 0.928 | 0.970 | 1.000 | 2148 | tags=20%, list=17%, signal=23% |
| 2587 | GOBP\_REGULATION\_OF\_DENDRITIC\_SPINE\_DEVELOPMENT |  | 42 | -0.20 | -0.74 | 0.866 | 0.970 | 1.000 | 5404 | tags=52%, list=42%, signal=90% |
| 2588 | GOBP\_REGULATION\_OF\_PHOSPHATASE\_ACTIVITY |  | 67 | -0.18 | -0.74 | 0.921 | 0.972 | 1.000 | 4366 | tags=37%, list=34%, signal=56% |
| 2589 | GOBP\_PHOSPHATIDYLINOSITOL\_3\_PHOSPHATE\_BIOSYNTHETIC\_PROCESS |  | 21 | -0.24 | -0.74 | 0.849 | 0.972 | 1.000 | 5583 | tags=62%, list=43%, signal=109% |
| 2590 | GOBP\_CALCIUM\_ION\_REGULATED\_EXOCYTOSIS |  | 49 | -0.19 | -0.74 | 0.899 | 0.972 | 1.000 | 4602 | tags=41%, list=36%, signal=63% |
| 2591 | GOBP\_EMBRYONIC\_SKELETAL\_SYSTEM\_DEVELOPMENT |  | 105 | -0.17 | -0.74 | 0.950 | 0.972 | 1.000 | 3358 | tags=26%, list=26%, signal=34% |
| 2592 | GOBP\_RESPONSE\_TO\_NITRIC\_OXIDE |  | 19 | -0.25 | -0.74 | 0.820 | 0.973 | 1.000 | 3689 | tags=32%, list=29%, signal=44% |
| 2593 | GOBP\_T\_CELL\_HOMEOSTASIS |  | 31 | -0.21 | -0.74 | 0.864 | 0.973 | 1.000 | 3730 | tags=32%, list=29%, signal=45% |
| 2594 | GOBP\_RESPONSE\_TO\_POTASSIUM\_ION |  | 15 | -0.26 | -0.74 | 0.806 | 0.974 | 1.000 | 1825 | tags=27%, list=14%, signal=31% |
| 2595 | GOBP\_REGULATION\_OF\_MYELOID\_LEUKOCYTE\_MEDIATED\_IMMUNITY |  | 57 | -0.19 | -0.74 | 0.914 | 0.974 | 1.000 | 2000 | tags=19%, list=16%, signal=23% |
| 2596 | GOBP\_ANIMAL\_ORGAN\_MATURATION |  | 27 | -0.23 | -0.74 | 0.843 | 0.974 | 1.000 | 2656 | tags=26%, list=21%, signal=33% |
| 2597 | GOBP\_REGULATION\_OF\_PHOSPHOPROTEIN\_PHOSPHATASE\_ACTIVITY |  | 48 | -0.19 | -0.74 | 0.899 | 0.973 | 1.000 | 3940 | tags=38%, list=31%, signal=54% |
| 2598 | GOBP\_PROTEIN\_LIPID\_COMPLEX\_SUBUNIT\_ORGANIZATION |  | 44 | -0.20 | -0.74 | 0.907 | 0.973 | 1.000 | 4046 | tags=39%, list=31%, signal=56% |
| 2599 | GOBP\_CARDIAC\_SEPTUM\_MORPHOGENESIS |  | 63 | -0.18 | -0.74 | 0.912 | 0.973 | 1.000 | 4102 | tags=30%, list=32%, signal=44% |
| 2600 | GOBP\_MODULATION\_BY\_HOST\_OF\_VIRAL\_PROCESS |  | 27 | -0.22 | -0.74 | 0.850 | 0.974 | 1.000 | 4048 | tags=41%, list=31%, signal=59% |
| 2601 | GOBP\_NEURON\_RECOGNITION |  | 40 | -0.20 | -0.73 | 0.886 | 0.977 | 1.000 | 2697 | tags=23%, list=21%, signal=28% |
| 2602 | GOBP\_NEURON\_CELL\_CELL\_ADHESION |  | 15 | -0.26 | -0.73 | 0.810 | 0.977 | 1.000 | 4527 | tags=40%, list=35%, signal=62% |
| 2603 | GOBP\_RIBONUCLEOSIDE\_DIPHOSPHATE\_METABOLIC\_PROCESS |  | 98 | -0.17 | -0.73 | 0.956 | 0.977 | 1.000 | 2148 | tags=18%, list=17%, signal=22% |
| 2604 | GOBP\_RESPONSE\_TO\_AMYLOID\_BETA |  | 51 | -0.19 | -0.73 | 0.898 | 0.977 | 1.000 | 3860 | tags=35%, list=30%, signal=50% |
| 2605 | GOBP\_REGULATION\_OF\_CARDIAC\_MUSCLE\_CONTRACTION |  | 67 | -0.18 | -0.73 | 0.909 | 0.976 | 1.000 | 1249 | tags=12%, list=10%, signal=13% |
| 2606 | GOBP\_CELLULAR\_GLUCAN\_METABOLIC\_PROCESS |  | 61 | -0.19 | -0.73 | 0.923 | 0.977 | 1.000 | 2072 | tags=16%, list=16%, signal=19% |
| 2607 | GOBP\_NEGATIVE\_REGULATION\_OF\_BONE\_MINERALIZATION |  | 17 | -0.24 | -0.73 | 0.848 | 0.978 | 1.000 | 1944 | tags=24%, list=15%, signal=28% |
| 2608 | GOBP\_MYELOID\_LEUKOCYTE\_MEDIATED\_IMMUNITY |  | 99 | -0.17 | -0.73 | 0.938 | 0.978 | 1.000 | 2028 | tags=18%, list=16%, signal=21% |
| 2609 | GOBP\_CYTOSOLIC\_CALCIUM\_ION\_TRANSPORT |  | 155 | -0.16 | -0.73 | 0.978 | 0.978 | 1.000 | 3081 | tags=22%, list=24%, signal=28% |
| 2610 | GOBP\_REGULATION\_OF\_INTEGRIN\_ACTIVATION |  | 15 | -0.26 | -0.73 | 0.813 | 0.978 | 1.000 | 3553 | tags=40%, list=28%, signal=55% |
| 2611 | GOBP\_NEGATIVE\_REGULATION\_OF\_DNA\_RECOMBINATION |  | 29 | -0.22 | -0.73 | 0.842 | 0.978 | 1.000 | 1170 | tags=14%, list=9%, signal=15% |
| 2612 | GOBP\_EXIT\_FROM\_MITOSIS |  | 24 | -0.23 | -0.73 | 0.848 | 0.977 | 1.000 | 1097 | tags=13%, list=9%, signal=14% |
| 2613 | GOBP\_RETINOIC\_ACID\_RECEPTOR\_SIGNALING\_PATHWAY |  | 29 | -0.22 | -0.73 | 0.859 | 0.977 | 1.000 | 2761 | tags=21%, list=21%, signal=26% |
| 2614 | GOBP\_CHRONIC\_INFLAMMATORY\_RESPONSE |  | 16 | -0.26 | -0.73 | 0.836 | 0.978 | 1.000 | 5393 | tags=50%, list=42%, signal=86% |
| 2615 | GOBP\_POSITIVE\_REGULATION\_OF\_MYELOID\_LEUKOCYTE\_CYTOKINE\_PRODUCTION\_INVOLVED\_IN\_IMMUNE\_RESPONSE |  | 22 | -0.23 | -0.73 | 0.855 | 0.978 | 1.000 | 3955 | tags=36%, list=31%, signal=52% |
| 2616 | GOBP\_POSITIVE\_REGULATION\_OF\_MEMBRANE\_PERMEABILITY |  | 37 | -0.20 | -0.73 | 0.870 | 0.977 | 1.000 | 4437 | tags=41%, list=34%, signal=62% |
| 2617 | GOBP\_THYROID\_HORMONE\_GENERATION |  | 19 | -0.24 | -0.73 | 0.831 | 0.977 | 1.000 | 2589 | tags=26%, list=20%, signal=33% |
| 2618 | GOBP\_POSITIVE\_REGULATION\_OF\_STEM\_CELL\_PROLIFERATION |  | 22 | -0.23 | -0.73 | 0.877 | 0.977 | 1.000 | 4370 | tags=41%, list=34%, signal=62% |
| 2619 | GOBP\_DOPAMINE\_RECEPTOR\_SIGNALING\_PATHWAY |  | 33 | -0.21 | -0.73 | 0.884 | 0.977 | 1.000 | 1745 | tags=15%, list=14%, signal=17% |
| 2620 | GOBP\_HISTONE\_MRNA\_METABOLIC\_PROCESS |  | 15 | -0.26 | -0.73 | 0.825 | 0.977 | 1.000 | 4498 | tags=47%, list=35%, signal=72% |
| 2621 | GOBP\_NEGATIVE\_REGULATION\_OF\_INTRACELLULAR\_TRANSPORT |  | 45 | -0.19 | -0.73 | 0.916 | 0.976 | 1.000 | 3813 | tags=33%, list=30%, signal=47% |
| 2622 | GOBP\_EPITHELIAL\_TUBE\_FORMATION |  | 107 | -0.17 | -0.73 | 0.957 | 0.976 | 1.000 | 4247 | tags=36%, list=33%, signal=53% |
| 2623 | GOBP\_REGULATION\_OF\_DEFENSE\_RESPONSE\_TO\_VIRUS |  | 57 | -0.19 | -0.73 | 0.911 | 0.976 | 1.000 | 2340 | tags=18%, list=18%, signal=21% |
| 2624 | GOBP\_POSITIVE\_REGULATION\_OF\_NATURAL\_KILLER\_CELL\_MEDIATED\_CYTOTOXICITY |  | 23 | -0.23 | -0.73 | 0.845 | 0.976 | 1.000 | 1760 | tags=17%, list=14%, signal=20% |
| 2625 | GOBP\_PROTEIN\_LOCALIZATION\_TO\_ENDOSOME |  | 20 | -0.23 | -0.73 | 0.859 | 0.976 | 1.000 | 4175 | tags=40%, list=32%, signal=59% |
| 2626 | GOBP\_NEGATIVE\_REGULATION\_OF\_MACROPHAGE\_ACTIVATION |  | 15 | -0.25 | -0.72 | 0.834 | 0.976 | 1.000 | 3872 | tags=33%, list=30%, signal=48% |
| 2627 | GOBP\_PROTEIN\_LOCALIZATION\_TO\_CHROMOSOME\_TELOMERIC\_REGION |  | 28 | -0.21 | -0.72 | 0.879 | 0.976 | 1.000 | 2311 | tags=18%, list=18%, signal=22% |
| 2628 | GOBP\_CELLULAR\_RESPONSE\_TO\_VIRUS |  | 70 | -0.18 | -0.72 | 0.941 | 0.977 | 1.000 | 3993 | tags=30%, list=31%, signal=43% |
| 2629 | GOBP\_LONG\_TERM\_SYNAPTIC\_POTENTIATION |  | 73 | -0.17 | -0.72 | 0.939 | 0.979 | 1.000 | 4351 | tags=40%, list=34%, signal=60% |
| 2630 | GOBP\_RESPONSE\_TO\_VITAMIN\_A |  | 17 | -0.24 | -0.72 | 0.851 | 0.979 | 1.000 | 3346 | tags=41%, list=26%, signal=56% |
| 2631 | GOBP\_CARDIAC\_MUSCLE\_CELL\_ACTION\_POTENTIAL |  | 63 | -0.18 | -0.72 | 0.935 | 0.979 | 1.000 | 4684 | tags=40%, list=36%, signal=62% |
| 2632 | GOBP\_POSITIVE\_REGULATION\_OF\_LIPID\_TRANSPORT |  | 76 | -0.17 | -0.72 | 0.948 | 0.981 | 1.000 | 4065 | tags=36%, list=32%, signal=52% |
| 2633 | GOBP\_POSITIVE\_REGULATION\_OF\_CELL\_JUNCTION\_ASSEMBLY |  | 82 | -0.17 | -0.72 | 0.949 | 0.981 | 1.000 | 3633 | tags=26%, list=28%, signal=35% |
| 2634 | GOBP\_NUCLEOBASE\_BIOSYNTHETIC\_PROCESS |  | 18 | -0.24 | -0.72 | 0.865 | 0.981 | 1.000 | 3427 | tags=33%, list=27%, signal=45% |
| 2635 | GOBP\_NEGATIVE\_REGULATION\_OF\_DNA\_BIOSYNTHETIC\_PROCESS |  | 31 | -0.21 | -0.72 | 0.902 | 0.982 | 1.000 | 1904 | tags=19%, list=15%, signal=23% |
| 2636 | GOBP\_POSITIVE\_REGULATION\_OF\_MYELOID\_LEUKOCYTE\_MEDIATED\_IMMUNITY |  | 19 | -0.24 | -0.71 | 0.857 | 0.982 | 1.000 | 4271 | tags=47%, list=33%, signal=71% |
| 2637 | GOBP\_CELLULAR\_RESPONSE\_TO\_COPPER\_ION |  | 20 | -0.23 | -0.71 | 0.860 | 0.982 | 1.000 | 5094 | tags=60%, list=39%, signal=99% |
| 2638 | GOBP\_PLASMA\_MEMBRANE\_ORGANIZATION |  | 111 | -0.16 | -0.71 | 0.951 | 0.982 | 1.000 | 1884 | tags=14%, list=15%, signal=17% |
| 2639 | GOBP\_RECEPTOR\_LOCALIZATION\_TO\_SYNAPSE |  | 44 | -0.19 | -0.71 | 0.909 | 0.982 | 1.000 | 2406 | tags=20%, list=19%, signal=25% |
| 2640 | GOBP\_NEUROTRANSMITTER\_TRANSPORT |  | 174 | -0.15 | -0.71 | 0.988 | 0.982 | 1.000 | 4398 | tags=36%, list=34%, signal=53% |
| 2641 | GOBP\_LIPID\_HOMEOSTASIS |  | 125 | -0.16 | -0.71 | 0.973 | 0.981 | 1.000 | 1540 | tags=14%, list=12%, signal=15% |
| 2642 | GOBP\_DETECTION\_OF\_CHEMICAL\_STIMULUS\_INVOLVED\_IN\_SENSORY\_PERCEPTION\_OF\_TASTE |  | 24 | -0.22 | -0.71 | 0.878 | 0.983 | 1.000 | 4091 | tags=33%, list=32%, signal=49% |
| 2643 | GOBP\_POSITIVE\_REGULATION\_OF\_COAGULATION |  | 22 | -0.23 | -0.71 | 0.887 | 0.983 | 1.000 | 2916 | tags=32%, list=23%, signal=41% |
| 2644 | GOBP\_MAMMARY\_GLAND\_DUCT\_MORPHOGENESIS |  | 28 | -0.21 | -0.71 | 0.880 | 0.982 | 1.000 | 4065 | tags=43%, list=32%, signal=62% |
| 2645 | GOBP\_EPITHELIAL\_STRUCTURE\_MAINTENANCE |  | 25 | -0.22 | -0.71 | 0.877 | 0.982 | 1.000 | 4475 | tags=40%, list=35%, signal=61% |
| 2646 | GOBP\_SENSORY\_PERCEPTION\_OF\_TEMPERATURE\_STIMULUS |  | 23 | -0.22 | -0.71 | 0.872 | 0.982 | 1.000 | 1229 | tags=13%, list=10%, signal=14% |
| 2647 | GOBP\_CELLULAR\_MONOVALENT\_INORGANIC\_CATION\_HOMEOSTASIS |  | 85 | -0.17 | -0.71 | 0.952 | 0.982 | 1.000 | 3479 | tags=28%, list=27%, signal=38% |
| 2648 | GOBP\_NEGATIVE\_REGULATION\_OF\_INFLAMMATORY\_RESPONSE\_TO\_ANTIGENIC\_STIMULUS |  | 23 | -0.22 | -0.71 | 0.876 | 0.982 | 1.000 | 2810 | tags=22%, list=22%, signal=28% |
| 2649 | GOBP\_GLYCEROPHOSPHOLIPID\_CATABOLIC\_PROCESS |  | 20 | -0.23 | -0.71 | 0.832 | 0.983 | 1.000 | 893 | tags=15%, list=7%, signal=16% |
| 2650 | GOBP\_SUPEROXIDE\_ANION\_GENERATION |  | 36 | -0.20 | -0.71 | 0.917 | 0.983 | 1.000 | 566 | tags=8%, list=4%, signal=9% |
| 2651 | GOBP\_VENTRICULAR\_SYSTEM\_DEVELOPMENT |  | 23 | -0.22 | -0.71 | 0.890 | 0.983 | 1.000 | 2702 | tags=26%, list=21%, signal=33% |
| 2652 | GOBP\_POSITIVE\_REGULATION\_OF\_INTERLEUKIN\_2\_PRODUCTION |  | 33 | -0.20 | -0.71 | 0.908 | 0.983 | 1.000 | 2078 | tags=18%, list=16%, signal=22% |
| 2653 | GOBP\_S\_ADENOSYLMETHIONINE\_METABOLIC\_PROCESS |  | 15 | -0.25 | -0.71 | 0.850 | 0.983 | 1.000 | 2487 | tags=20%, list=19%, signal=25% |
| 2654 | GOBP\_MAST\_CELL\_ACTIVATION\_INVOLVED\_IN\_IMMUNE\_RESPONSE |  | 51 | -0.19 | -0.71 | 0.937 | 0.983 | 1.000 | 2000 | tags=18%, list=16%, signal=21% |
| 2655 | GOBP\_NEGATIVE\_REGULATION\_OF\_INTRACELLULAR\_PROTEIN\_TRANSPORT |  | 34 | -0.20 | -0.70 | 0.916 | 0.984 | 1.000 | 3813 | tags=35%, list=30%, signal=50% |
| 2656 | GOBP\_EYE\_PHOTORECEPTOR\_CELL\_DIFFERENTIATION |  | 36 | -0.20 | -0.70 | 0.912 | 0.984 | 1.000 | 3269 | tags=25%, list=25%, signal=33% |
| 2657 | GOBP\_NEGATIVE\_REGULATION\_OF\_ADAPTIVE\_IMMUNE\_RESPONSE |  | 52 | -0.18 | -0.70 | 0.944 | 0.984 | 1.000 | 3872 | tags=33%, list=30%, signal=47% |
| 2658 | GOBP\_REGULATED\_EXOCYTOSIS |  | 192 | -0.15 | -0.70 | 0.996 | 0.984 | 1.000 | 3691 | tags=28%, list=29%, signal=39% |
| 2659 | GOBP\_LIPID\_TRANSLOCATION |  | 41 | -0.19 | -0.70 | 0.917 | 0.984 | 1.000 | 4046 | tags=32%, list=31%, signal=46% |
| 2660 | GOBP\_PERIPHERAL\_NERVOUS\_SYSTEM\_DEVELOPMENT |  | 71 | -0.17 | -0.70 | 0.958 | 0.985 | 1.000 | 827 | tags=8%, list=6%, signal=9% |
| 2661 | GOBP\_PEPTIDYL\_ARGININE\_MODIFICATION |  | 22 | -0.22 | -0.70 | 0.875 | 0.986 | 1.000 | 4998 | tags=59%, list=39%, signal=96% |
| 2662 | GOBP\_CELLULAR\_RESPONSE\_TO\_AMYLOID\_BETA |  | 42 | -0.19 | -0.70 | 0.904 | 0.986 | 1.000 | 3577 | tags=33%, list=28%, signal=46% |
| 2663 | GOBP\_ACTIN\_FILAMENT\_SEVERING |  | 15 | -0.25 | -0.70 | 0.858 | 0.985 | 1.000 | 5024 | tags=53%, list=39%, signal=87% |
| 2664 | GOBP\_ALPHA\_BETA\_T\_CELL\_PROLIFERATION |  | 33 | -0.20 | -0.70 | 0.933 | 0.986 | 1.000 | 2831 | tags=24%, list=22%, signal=31% |
| 2665 | GOBP\_REGULATION\_OF\_DENDRITE\_EXTENSION |  | 18 | -0.23 | -0.69 | 0.887 | 0.988 | 1.000 | 4182 | tags=44%, list=32%, signal=66% |
| 2666 | GOBP\_CARDIAC\_MUSCLE\_CELL\_MEMBRANE\_REPOLARIZATION |  | 31 | -0.20 | -0.69 | 0.913 | 0.988 | 1.000 | 3951 | tags=35%, list=31%, signal=51% |
| 2667 | GOBP\_CHAPERONE\_COFACTOR\_DEPENDENT\_PROTEIN\_REFOLDING |  | 28 | -0.20 | -0.69 | 0.900 | 0.988 | 1.000 | 4415 | tags=43%, list=34%, signal=65% |
| 2668 | GOBP\_NEGATIVE\_REGULATION\_OF\_ENDOTHELIAL\_CELL\_PROLIFERATION |  | 38 | -0.19 | -0.69 | 0.924 | 0.989 | 1.000 | 4762 | tags=39%, list=37%, signal=62% |
| 2669 | GOBP\_REGULATION\_OF\_SMOOTHENED\_SIGNALING\_PATHWAY |  | 61 | -0.18 | -0.69 | 0.955 | 0.988 | 1.000 | 3177 | tags=28%, list=25%, signal=37% |
| 2670 | GOBP\_POSITIVE\_REGULATION\_OF\_INTERLEUKIN\_8\_PRODUCTION |  | 57 | -0.18 | -0.69 | 0.959 | 0.988 | 1.000 | 949 | tags=12%, list=7%, signal=13% |
| 2671 | GOBP\_POSITIVE\_REGULATION\_OF\_ACTIN\_CYTOSKELETON\_REORGANIZATION |  | 17 | -0.23 | -0.69 | 0.885 | 0.989 | 1.000 | 745 | tags=12%, list=6%, signal=12% |
| 2672 | GOBP\_SINGLE\_STRANDED\_VIRAL\_RNA\_REPLICATION\_VIA\_DOUBLE\_STRANDED\_DNA\_INTERMEDIATE |  | 18 | -0.23 | -0.69 | 0.901 | 0.988 | 1.000 | 3088 | tags=28%, list=24%, signal=36% |
| 2673 | GOBP\_NEUTRAL\_LIPID\_BIOSYNTHETIC\_PROCESS |  | 27 | -0.21 | -0.69 | 0.903 | 0.988 | 1.000 | 3870 | tags=37%, list=30%, signal=53% |
| 2674 | GOBP\_NATURAL\_KILLER\_CELL\_ACTIVATION |  | 74 | -0.17 | -0.69 | 0.968 | 0.988 | 1.000 | 4654 | tags=39%, list=36%, signal=61% |
| 2675 | GOBP\_DOPAMINE\_SECRETION |  | 28 | -0.21 | -0.69 | 0.907 | 0.988 | 1.000 | 3436 | tags=32%, list=27%, signal=44% |
| 2676 | GOBP\_SPHINGOID\_METABOLIC\_PROCESS |  | 15 | -0.24 | -0.69 | 0.848 | 0.988 | 1.000 | 1742 | tags=27%, list=14%, signal=31% |
| 2677 | GOBP\_FACULTATIVE\_HETEROCHROMATIN\_ASSEMBLY |  | 26 | -0.21 | -0.69 | 0.897 | 0.989 | 1.000 | 1379 | tags=12%, list=11%, signal=13% |
| 2678 | GOBP\_RETINA\_MORPHOGENESIS\_IN\_CAMERA\_TYPE\_EYE |  | 43 | -0.19 | -0.69 | 0.929 | 0.989 | 1.000 | 2511 | tags=19%, list=19%, signal=23% |
| 2679 | GOBP\_REGULATION\_OF\_LEUKOCYTE\_MEDIATED\_CYTOTOXICITY |  | 75 | -0.17 | -0.69 | 0.961 | 0.989 | 1.000 | 2833 | tags=21%, list=22%, signal=27% |
| 2680 | GOBP\_RNA\_PHOSPHODIESTER\_BOND\_HYDROLYSIS\_EXONUCLEOLYTIC |  | 30 | -0.20 | -0.69 | 0.919 | 0.989 | 1.000 | 2498 | tags=23%, list=19%, signal=29% |
| 2681 | GOBP\_NEGATIVE\_REGULATION\_OF\_PROTEIN\_POLYMERIZATION |  | 66 | -0.17 | -0.68 | 0.970 | 0.989 | 1.000 | 3697 | tags=29%, list=29%, signal=40% |
| 2682 | GOBP\_NEURAL\_TUBE\_FORMATION |  | 84 | -0.16 | -0.68 | 0.978 | 0.989 | 1.000 | 4523 | tags=40%, list=35%, signal=62% |
| 2683 | GOBP\_REGULATION\_OF\_EXOCYTOSIS |  | 174 | -0.14 | -0.68 | 0.993 | 0.989 | 1.000 | 3081 | tags=23%, list=24%, signal=30% |
| 2684 | GOBP\_CATECHOL\_CONTAINING\_COMPOUND\_BIOSYNTHETIC\_PROCESS |  | 21 | -0.22 | -0.68 | 0.894 | 0.989 | 1.000 | 626 | tags=10%, list=5%, signal=10% |
| 2685 | GOBP\_PHOTORECEPTOR\_CELL\_MAINTENANCE |  | 25 | -0.21 | -0.68 | 0.908 | 0.989 | 1.000 | 2665 | tags=24%, list=21%, signal=30% |
| 2686 | GOBP\_REGULATION\_OF\_NEUTROPHIL\_MIGRATION |  | 41 | -0.18 | -0.68 | 0.936 | 0.989 | 1.000 | 3526 | tags=29%, list=27%, signal=40% |
| 2687 | GOBP\_CELLULAR\_MODIFIED\_AMINO\_ACID\_CATABOLIC\_PROCESS |  | 18 | -0.22 | -0.68 | 0.882 | 0.988 | 1.000 | 3825 | tags=33%, list=30%, signal=47% |
| 2688 | GOBP\_LUNG\_EPITHELIUM\_DEVELOPMENT |  | 33 | -0.20 | -0.68 | 0.928 | 0.988 | 1.000 | 4147 | tags=36%, list=32%, signal=53% |
| 2689 | GOBP\_REGULATION\_OF\_PROTEIN\_DEPOLYMERIZATION |  | 75 | -0.16 | -0.68 | 0.978 | 0.988 | 1.000 | 2886 | tags=21%, list=22%, signal=27% |
| 2690 | GOBP\_NEUROMUSCULAR\_SYNAPTIC\_TRANSMISSION |  | 21 | -0.22 | -0.68 | 0.890 | 0.988 | 1.000 | 2607 | tags=24%, list=20%, signal=30% |
| 2691 | GOBP\_AMYLOID\_PRECURSOR\_PROTEIN\_METABOLIC\_PROCESS |  | 68 | -0.17 | -0.68 | 0.975 | 0.988 | 1.000 | 4136 | tags=35%, list=32%, signal=52% |
| 2692 | GOBP\_POLYOL\_METABOLIC\_PROCESS |  | 95 | -0.16 | -0.68 | 0.983 | 0.988 | 1.000 | 1486 | tags=13%, list=12%, signal=14% |
| 2693 | GOBP\_REGULATION\_OF\_TRANSCRIPTION\_INVOLVED\_IN\_G1\_S\_TRANSITION\_OF\_MITOTIC\_CELL\_CYCLE |  | 18 | -0.23 | -0.68 | 0.901 | 0.989 | 1.000 | 1050 | tags=11%, list=8%, signal=12% |
| 2694 | GOBP\_REGULATION\_OF\_INTRINSIC\_APOPTOTIC\_SIGNALING\_PATHWAY\_IN\_RESPONSE\_TO\_DNA\_DAMAGE\_BY\_P53\_CLASS\_MEDIATOR |  | 15 | -0.24 | -0.68 | 0.878 | 0.991 | 1.000 | 1463 | tags=13%, list=11%, signal=15% |
| 2695 | GOBP\_MAMMARY\_GLAND\_MORPHOGENESIS |  | 39 | -0.19 | -0.67 | 0.948 | 0.992 | 1.000 | 4711 | tags=49%, list=37%, signal=77% |
| 2696 | GOBP\_NEGATIVE\_REGULATION\_OF\_BLOOD\_CIRCULATION |  | 23 | -0.21 | -0.67 | 0.914 | 0.992 | 1.000 | 5119 | tags=57%, list=40%, signal=94% |
| 2697 | GOBP\_EXOCYTOSIS |  | 285 | -0.14 | -0.67 | 0.999 | 0.991 | 1.000 | 3691 | tags=27%, list=29%, signal=37% |
| 2698 | GOBP\_HOMOLOGOUS\_CHROMOSOME\_SEGREGATION |  | 40 | -0.18 | -0.67 | 0.947 | 0.991 | 1.000 | 4169 | tags=35%, list=32%, signal=52% |
| 2699 | GOBP\_L\_GLUTAMATE\_TRANSMEMBRANE\_TRANSPORT |  | 26 | -0.20 | -0.67 | 0.933 | 0.991 | 1.000 | 336 | tags=8%, list=3%, signal=8% |
| 2700 | GOBP\_CENTROMERE\_COMPLEX\_ASSEMBLY |  | 26 | -0.20 | -0.67 | 0.926 | 0.991 | 1.000 | 3591 | tags=31%, list=28%, signal=43% |
| 2701 | GOBP\_AMINOGLYCAN\_BIOSYNTHETIC\_PROCESS |  | 60 | -0.17 | -0.67 | 0.974 | 0.991 | 1.000 | 1274 | tags=13%, list=10%, signal=15% |
| 2702 | GOBP\_NEGATIVE\_REGULATION\_OF\_DEFENSE\_RESPONSE\_TO\_VIRUS |  | 25 | -0.20 | -0.67 | 0.919 | 0.992 | 1.000 | 3210 | tags=28%, list=25%, signal=37% |
| 2703 | GOBP\_REGULATION\_OF\_T\_HELPER\_CELL\_DIFFERENTIATION |  | 31 | -0.19 | -0.67 | 0.936 | 0.992 | 1.000 | 2819 | tags=23%, list=22%, signal=29% |
| 2704 | GOBP\_KERATAN\_SULFATE\_METABOLIC\_PROCESS |  | 15 | -0.24 | -0.67 | 0.885 | 0.992 | 1.000 | 3557 | tags=47%, list=28%, signal=64% |
| 2705 | GOBP\_CARDIAC\_VENTRICLE\_MORPHOGENESIS |  | 67 | -0.17 | -0.67 | 0.967 | 0.992 | 1.000 | 1069 | tags=9%, list=8%, signal=10% |
| 2706 | GOBP\_VENTRICULAR\_CARDIAC\_MUSCLE\_TISSUE\_DEVELOPMENT |  | 53 | -0.18 | -0.67 | 0.971 | 0.991 | 1.000 | 1050 | tags=9%, list=8%, signal=10% |
| 2707 | GOBP\_CYTOPLASMIC\_MICROTUBULE\_ORGANIZATION |  | 45 | -0.18 | -0.67 | 0.953 | 0.991 | 1.000 | 4668 | tags=42%, list=36%, signal=66% |
| 2708 | GOBP\_CILIUM\_MOVEMENT |  | 91 | -0.15 | -0.67 | 0.988 | 0.992 | 1.000 | 3097 | tags=24%, list=24%, signal=32% |
| 2709 | GOBP\_HISTONE\_MONOUBIQUITINATION |  | 20 | -0.21 | -0.66 | 0.893 | 0.992 | 1.000 | 2516 | tags=20%, list=20%, signal=25% |
| 2710 | GOBP\_REGULATION\_OF\_TRIGLYCERIDE\_METABOLIC\_PROCESS |  | 29 | -0.20 | -0.66 | 0.927 | 0.992 | 1.000 | 4020 | tags=38%, list=31%, signal=55% |
| 2711 | GOBP\_RESPONSE\_TO\_LECTIN |  | 20 | -0.22 | -0.66 | 0.911 | 0.991 | 1.000 | 2810 | tags=20%, list=22%, signal=26% |
| 2712 | GOBP\_POSITIVE\_REGULATION\_OF\_SMAD\_PROTEIN\_SIGNAL\_TRANSDUCTION |  | 17 | -0.22 | -0.66 | 0.893 | 0.991 | 1.000 | 4355 | tags=41%, list=34%, signal=62% |
| 2713 | GOBP\_NEGATIVE\_REGULATION\_OF\_EXOCYTOSIS |  | 33 | -0.19 | -0.66 | 0.947 | 0.992 | 1.000 | 3238 | tags=27%, list=25%, signal=36% |
| 2714 | GOBP\_REGULATION\_OF\_CHOLESTEROL\_EFFLUX |  | 32 | -0.19 | -0.66 | 0.929 | 0.991 | 1.000 | 4065 | tags=38%, list=32%, signal=55% |
| 2715 | GOBP\_EYE\_PHOTORECEPTOR\_CELL\_DEVELOPMENT |  | 28 | -0.20 | -0.66 | 0.941 | 0.991 | 1.000 | 3959 | tags=32%, list=31%, signal=46% |
| 2716 | GOBP\_REGULATION\_OF\_CELL\_KILLING |  | 92 | -0.16 | -0.66 | 0.991 | 0.991 | 1.000 | 2842 | tags=20%, list=22%, signal=25% |
| 2717 | GOBP\_ORGANIC\_HYDROXY\_COMPOUND\_CATABOLIC\_PROCESS |  | 67 | -0.16 | -0.66 | 0.978 | 0.991 | 1.000 | 2725 | tags=19%, list=21%, signal=24% |
| 2718 | GOBP\_REGULATION\_OF\_CALCINEURIN\_MEDIATED\_SIGNALING |  | 31 | -0.19 | -0.66 | 0.928 | 0.992 | 1.000 | 3860 | tags=32%, list=30%, signal=46% |
| 2719 | GOBP\_PYRIMIDINE\_NUCLEOSIDE\_METABOLIC\_PROCESS |  | 19 | -0.21 | -0.66 | 0.911 | 0.992 | 1.000 | 1387 | tags=16%, list=11%, signal=18% |
| 2720 | GOBP\_MYOTUBE\_CELL\_DEVELOPMENT |  | 29 | -0.20 | -0.66 | 0.948 | 0.992 | 1.000 | 3330 | tags=28%, list=26%, signal=37% |
| 2721 | GOBP\_REGULATION\_OF\_STRIATED\_MUSCLE\_CONTRACTION |  | 82 | -0.16 | -0.66 | 0.992 | 0.992 | 1.000 | 1249 | tags=11%, list=10%, signal=12% |
| 2722 | GOBP\_REGULATION\_OF\_NEUTROPHIL\_CHEMOTAXIS |  | 30 | -0.19 | -0.66 | 0.936 | 0.992 | 1.000 | 2204 | tags=20%, list=17%, signal=24% |
| 2723 | GOBP\_PROTEIN\_LOCALIZATION\_TO\_POSTSYNAPSE |  | 28 | -0.19 | -0.65 | 0.941 | 0.992 | 1.000 | 2406 | tags=25%, list=19%, signal=31% |
| 2724 | GOBP\_LIPOPROTEIN\_LOCALIZATION |  | 16 | -0.23 | -0.65 | 0.909 | 0.993 | 1.000 | 3362 | tags=31%, list=26%, signal=42% |
| 2725 | GOBP\_NEGATIVE\_REGULATION\_OF\_RECEPTOR\_MEDIATED\_ENDOCYTOSIS |  | 26 | -0.20 | -0.65 | 0.931 | 0.993 | 1.000 | 1825 | tags=19%, list=14%, signal=22% |
| 2726 | GOBP\_CARTILAGE\_DEVELOPMENT\_INVOLVED\_IN\_ENDOCHONDRAL\_BONE\_MORPHOGENESIS |  | 28 | -0.19 | -0.65 | 0.946 | 0.993 | 1.000 | 4977 | tags=54%, list=39%, signal=87% |
| 2727 | GOBP\_DEFINITIVE\_HEMOPOIESIS |  | 17 | -0.22 | -0.65 | 0.914 | 0.993 | 1.000 | 4279 | tags=41%, list=33%, signal=62% |
| 2728 | GOBP\_MAST\_CELL\_ACTIVATION |  | 63 | -0.16 | -0.65 | 0.979 | 0.994 | 1.000 | 2000 | tags=16%, list=16%, signal=19% |
| 2729 | GOBP\_AMYLOID\_PRECURSOR\_PROTEIN\_CATABOLIC\_PROCESS |  | 52 | -0.17 | -0.65 | 0.975 | 0.994 | 1.000 | 1381 | tags=13%, list=11%, signal=15% |
| 2730 | GOBP\_NEUTRAL\_LIPID\_CATABOLIC\_PROCESS |  | 28 | -0.19 | -0.64 | 0.939 | 0.995 | 1.000 | 4528 | tags=46%, list=35%, signal=71% |
| 2731 | GOBP\_POSITIVE\_REGULATION\_OF\_SYNAPTIC\_TRANSMISSION\_GLUTAMATERGIC |  | 21 | -0.20 | -0.64 | 0.921 | 0.995 | 1.000 | 1284 | tags=14%, list=10%, signal=16% |
| 2732 | GOBP\_GLYCEROLIPID\_CATABOLIC\_PROCESS |  | 46 | -0.17 | -0.64 | 0.969 | 0.996 | 1.000 | 4528 | tags=41%, list=35%, signal=63% |
| 2733 | GOBP\_POSITIVE\_REGULATION\_OF\_FATTY\_ACID\_TRANSPORT |  | 24 | -0.20 | -0.64 | 0.933 | 0.995 | 1.000 | 2197 | tags=21%, list=17%, signal=25% |
| 2734 | GOBP\_SNRNA\_METABOLIC\_PROCESS |  | 45 | -0.17 | -0.64 | 0.966 | 0.995 | 1.000 | 2401 | tags=22%, list=19%, signal=27% |
| 2735 | GOBP\_REGULATION\_OF\_SYSTEMIC\_ARTERIAL\_BLOOD\_PRESSURE\_BY\_CIRCULATORY\_RENIN\_ANGIOTENSIN |  | 15 | -0.22 | -0.64 | 0.919 | 0.995 | 1.000 | 4455 | tags=47%, list=35%, signal=71% |
| 2736 | GOBP\_REGULATION\_OF\_MEMBRANE\_LIPID\_DISTRIBUTION |  | 42 | -0.18 | -0.64 | 0.958 | 0.995 | 1.000 | 4046 | tags=31%, list=31%, signal=45% |
| 2737 | GOBP\_MEMBRANE\_DEPOLARIZATION\_DURING\_ACTION\_POTENTIAL |  | 32 | -0.19 | -0.64 | 0.945 | 0.996 | 1.000 | 4684 | tags=41%, list=36%, signal=64% |
| 2738 | GOBP\_POSITIVE\_REGULATION\_OF\_TYPE\_2\_IMMUNE\_RESPONSE |  | 17 | -0.22 | -0.64 | 0.923 | 0.995 | 1.000 | 626 | tags=12%, list=5%, signal=12% |
| 2739 | GOBP\_SELECTIVE\_AUTOPHAGY |  | 65 | -0.16 | -0.63 | 0.984 | 0.996 | 1.000 | 3478 | tags=26%, list=27%, signal=36% |
| 2740 | GOBP\_COPPER\_ION\_HOMEOSTASIS |  | 15 | -0.23 | -0.63 | 0.919 | 0.996 | 1.000 | 2361 | tags=20%, list=18%, signal=24% |
| 2741 | GOBP\_THYROID\_GLAND\_DEVELOPMENT |  | 25 | -0.19 | -0.63 | 0.949 | 0.996 | 1.000 | 4319 | tags=36%, list=33%, signal=54% |
| 2742 | GOBP\_BRANCHING\_INVOLVED\_IN\_SALIVARY\_GLAND\_MORPHOGENESIS |  | 19 | -0.21 | -0.63 | 0.951 | 0.996 | 1.000 | 798 | tags=11%, list=6%, signal=11% |
| 2743 | GOBP\_STEROL\_HOMEOSTASIS |  | 74 | -0.15 | -0.63 | 0.993 | 0.996 | 1.000 | 3028 | tags=20%, list=23%, signal=26% |
| 2744 | GOBP\_SOMATIC\_DIVERSIFICATION\_OF\_IMMUNOGLOBULINS\_INVOLVED\_IN\_IMMUNE\_RESPONSE |  | 43 | -0.17 | -0.63 | 0.974 | 0.996 | 1.000 | 542 | tags=7%, list=4%, signal=7% |
| 2745 | GOBP\_AMINE\_TRANSPORT |  | 88 | -0.15 | -0.63 | 0.995 | 0.995 | 1.000 | 4816 | tags=41%, list=37%, signal=65% |
| 2746 | GOBP\_REGULATION\_OF\_T\_HELPER\_17\_TYPE\_IMMUNE\_RESPONSE |  | 18 | -0.20 | -0.63 | 0.936 | 0.996 | 1.000 | 197 | tags=6%, list=2%, signal=6% |
| 2747 | GOBP\_NCRNA\_CATABOLIC\_PROCESS |  | 27 | -0.19 | -0.63 | 0.957 | 0.996 | 1.000 | 3278 | tags=26%, list=25%, signal=35% |
| 2748 | GOBP\_CHOLESTEROL\_EFFLUX |  | 47 | -0.17 | -0.62 | 0.981 | 0.996 | 1.000 | 4065 | tags=34%, list=32%, signal=50% |
| 2749 | GOBP\_MEIOTIC\_CELL\_CYCLE\_PROCESS |  | 139 | -0.14 | -0.62 | 0.999 | 0.997 | 1.000 | 4169 | tags=31%, list=32%, signal=45% |
| 2750 | GOBP\_POSITIVE\_REGULATION\_OF\_PROTEIN\_LOCALIZATION\_TO\_NUCLEUS |  | 76 | -0.15 | -0.62 | 0.987 | 0.997 | 1.000 | 3553 | tags=32%, list=28%, signal=43% |
| 2751 | GOBP\_VOCALIZATION\_BEHAVIOR |  | 16 | -0.21 | -0.62 | 0.956 | 0.997 | 1.000 | 3046 | tags=25%, list=24%, signal=33% |
| 2752 | GOBP\_CARDIAC\_MUSCLE\_CELL\_ACTION\_POTENTIAL\_INVOLVED\_IN\_CONTRACTION |  | 45 | -0.16 | -0.62 | 0.986 | 0.998 | 1.000 | 4358 | tags=33%, list=34%, signal=50% |
| 2753 | GOBP\_MEMBRANE\_REPOLARIZATION\_DURING\_CARDIAC\_MUSCLE\_CELL\_ACTION\_POTENTIAL |  | 18 | -0.21 | -0.62 | 0.948 | 0.998 | 1.000 | 1739 | tags=17%, list=13%, signal=19% |
| 2754 | GOBP\_POSITIVE\_REGULATION\_OF\_T\_CELL\_RECEPTOR\_SIGNALING\_PATHWAY |  | 15 | -0.21 | -0.61 | 0.959 | 0.999 | 1.000 | 4972 | tags=53%, list=39%, signal=87% |
| 2755 | GOBP\_RESPONSE\_TO\_MISFOLDED\_PROTEIN |  | 21 | -0.20 | -0.61 | 0.959 | 0.999 | 1.000 | 2415 | tags=24%, list=19%, signal=29% |
| 2756 | GOBP\_REGULATION\_OF\_CIRCADIAN\_SLEEP\_WAKE\_CYCLE |  | 17 | -0.20 | -0.61 | 0.939 | 0.998 | 1.000 | 5928 | tags=65%, list=46%, signal=120% |
| 2757 | GOBP\_KINETOCHORE\_ASSEMBLY |  | 15 | -0.22 | -0.61 | 0.949 | 0.998 | 1.000 | 4079 | tags=40%, list=32%, signal=58% |
| 2758 | GOBP\_NEGATIVE\_REGULATION\_OF\_RESPONSE\_TO\_ENDOPLASMIC\_RETICULUM\_STRESS |  | 35 | -0.17 | -0.61 | 0.971 | 0.998 | 1.000 | 4247 | tags=34%, list=33%, signal=51% |
| 2759 | GOBP\_POSITIVE\_REGULATION\_OF\_TRIGLYCERIDE\_METABOLIC\_PROCESS |  | 15 | -0.21 | -0.61 | 0.931 | 0.998 | 1.000 | 4020 | tags=40%, list=31%, signal=58% |
| 2760 | GOBP\_REGULATION\_OF\_NEURONAL\_SYNAPTIC\_PLASTICITY |  | 36 | -0.17 | -0.61 | 0.977 | 0.997 | 1.000 | 5196 | tags=56%, list=40%, signal=93% |
| 2761 | GOBP\_CARDIAC\_MUSCLE\_CONTRACTION |  | 121 | -0.14 | -0.61 | 0.998 | 0.997 | 1.000 | 3035 | tags=20%, list=24%, signal=26% |
| 2762 | GOBP\_TETRAHYDROFOLATE\_METABOLIC\_PROCESS |  | 16 | -0.21 | -0.61 | 0.950 | 0.998 | 1.000 | 3600 | tags=38%, list=28%, signal=52% |
| 2763 | GOBP\_FC\_RECEPTOR\_MEDIATED\_STIMULATORY\_SIGNALING\_PATHWAY |  | 31 | -0.17 | -0.61 | 0.970 | 0.997 | 1.000 | 3846 | tags=32%, list=30%, signal=46% |
| 2764 | GOBP\_PHOTORECEPTOR\_CELL\_DEVELOPMENT |  | 38 | -0.17 | -0.60 | 0.975 | 0.997 | 1.000 | 3269 | tags=24%, list=25%, signal=32% |
| 2765 | GOBP\_CARDIAC\_CONDUCTION |  | 82 | -0.14 | -0.60 | 0.998 | 0.997 | 1.000 | 3035 | tags=24%, list=24%, signal=32% |
| 2766 | GOBP\_REGULATION\_OF\_INFLAMMATORY\_RESPONSE\_TO\_ANTIGENIC\_STIMULUS |  | 37 | -0.17 | -0.60 | 0.985 | 0.997 | 1.000 | 2981 | tags=22%, list=23%, signal=28% |
| 2767 | GOBP\_REGULATION\_OF\_MEMBRANE\_REPOLARIZATION |  | 28 | -0.18 | -0.60 | 0.955 | 0.997 | 1.000 | 3951 | tags=32%, list=31%, signal=46% |
| 2768 | GOBP\_PROTEIN\_QUALITY\_CONTROL\_FOR\_MISFOLDED\_OR\_INCOMPLETELY\_SYNTHESIZED\_PROTEINS |  | 22 | -0.19 | -0.60 | 0.961 | 0.998 | 1.000 | 2415 | tags=23%, list=19%, signal=28% |
| 2769 | GOBP\_POSITIVE\_REGULATION\_OF\_GLYCOPROTEIN\_METABOLIC\_PROCESS |  | 22 | -0.19 | -0.60 | 0.957 | 0.998 | 1.000 | 4136 | tags=36%, list=32%, signal=53% |
| 2770 | GOBP\_REGULATION\_OF\_CHROMATIN\_ASSEMBLY |  | 20 | -0.19 | -0.60 | 0.952 | 0.998 | 1.000 | 4434 | tags=35%, list=34%, signal=53% |
| 2771 | GOBP\_VENTRICULAR\_SEPTUM\_MORPHOGENESIS |  | 36 | -0.17 | -0.59 | 0.983 | 0.998 | 1.000 | 5086 | tags=42%, list=39%, signal=69% |
| 2772 | GOBP\_PROTEIN\_K11\_LINKED\_UBIQUITINATION |  | 22 | -0.19 | -0.59 | 0.963 | 0.998 | 1.000 | 3958 | tags=36%, list=31%, signal=52% |
| 2773 | GOBP\_RESPONSE\_TO\_PROSTAGLANDIN\_E |  | 21 | -0.18 | -0.59 | 0.975 | 0.999 | 1.000 | 4243 | tags=38%, list=33%, signal=57% |
| 2774 | GOBP\_REGULATION\_OF\_CHOLESTEROL\_BIOSYNTHETIC\_PROCESS |  | 19 | -0.19 | -0.59 | 0.966 | 0.998 | 1.000 | 1622 | tags=16%, list=13%, signal=18% |
| 2775 | GOBP\_PHOSPHATIDYLGLYCEROL\_METABOLIC\_PROCESS |  | 22 | -0.19 | -0.59 | 0.962 | 0.999 | 1.000 | 2473 | tags=18%, list=19%, signal=22% |
| 2776 | GOBP\_ACTIN\_FILAMENT\_BASED\_MOVEMENT |  | 114 | -0.13 | -0.58 | 1.000 | 0.999 | 1.000 | 3951 | tags=28%, list=31%, signal=40% |
| 2777 | GOBP\_SYNAPTONEMAL\_COMPLEX\_ORGANIZATION |  | 19 | -0.19 | -0.58 | 0.971 | 1.000 | 1.000 | 4099 | tags=37%, list=32%, signal=54% |
| 2778 | GOBP\_MULTICELLULAR\_ORGANISMAL\_SIGNALING |  | 140 | -0.13 | -0.58 | 1.000 | 1.000 | 1.000 | 4430 | tags=35%, list=34%, signal=53% |
| 2779 | GOBP\_TRANSMISSION\_OF\_NERVE\_IMPULSE |  | 64 | -0.14 | -0.57 | 0.998 | 1.000 | 1.000 | 4430 | tags=36%, list=34%, signal=54% |
| 2780 | GOBP\_STRIATED\_MUSCLE\_ADAPTATION |  | 39 | -0.16 | -0.57 | 0.995 | 1.000 | 1.000 | 1966 | tags=15%, list=15%, signal=18% |
| 2781 | GOBP\_PROSTATE\_GLAND\_MORPHOGENESIS |  | 23 | -0.18 | -0.57 | 0.976 | 1.000 | 1.000 | 1554 | tags=13%, list=12%, signal=15% |
| 2782 | GOBP\_NEGATIVE\_REGULATION\_OF\_POTASSIUM\_ION\_TRANSPORT |  | 27 | -0.17 | -0.57 | 0.967 | 1.000 | 1.000 | 4005 | tags=33%, list=31%, signal=48% |
| 2783 | GOBP\_REGULATION\_OF\_PROTEIN\_LOCALIZATION\_TO\_SYNAPSE |  | 16 | -0.20 | -0.57 | 0.969 | 1.000 | 1.000 | 2169 | tags=19%, list=17%, signal=23% |
| 2784 | GOBP\_LOW\_DENSITY\_LIPOPROTEIN\_PARTICLE\_REMODELING |  | 16 | -0.19 | -0.56 | 0.969 | 1.000 | 1.000 | 3435 | tags=31%, list=27%, signal=43% |
| 2785 | GOBP\_FEMALE\_GENITALIA\_DEVELOPMENT |  | 17 | -0.19 | -0.56 | 0.970 | 1.000 | 1.000 | 311 | tags=6%, list=2%, signal=6% |
| 2786 | GOBP\_POSITIVE\_REGULATION\_OF\_ALPHA\_BETA\_T\_CELL\_ACTIVATION |  | 62 | -0.14 | -0.56 | 0.997 | 1.000 | 1.000 | 2819 | tags=19%, list=22%, signal=25% |
| 2787 | GOBP\_CARDIAC\_MUSCLE\_CELL\_CONTRACTION |  | 64 | -0.14 | -0.56 | 0.997 | 1.000 | 1.000 | 4684 | tags=36%, list=36%, signal=56% |
| 2788 | GOBP\_NEUROTRANSMITTER\_UPTAKE |  | 42 | -0.15 | -0.56 | 0.990 | 1.000 | 1.000 | 2725 | tags=21%, list=21%, signal=27% |
| 2789 | GOBP\_CELL\_DIFFERENTIATION\_IN\_SPINAL\_CORD |  | 33 | -0.16 | -0.55 | 0.986 | 1.000 | 1.000 | 2196 | tags=15%, list=17%, signal=18% |
| 2790 | GOBP\_MEMBRANE\_REPOLARIZATION\_DURING\_ACTION\_POTENTIAL |  | 22 | -0.18 | -0.55 | 0.982 | 0.999 | 1.000 | 1739 | tags=14%, list=13%, signal=16% |
| 2791 | GOBP\_REGULATION\_OF\_CELL\_MATURATION |  | 17 | -0.19 | -0.55 | 0.981 | 1.000 | 1.000 | 10511 | tags=100%, list=81%, signal=539% |
| 2792 | GOBP\_RESPONSE\_TO\_ANTIBIOTIC |  | 42 | -0.14 | -0.54 | 0.987 | 1.000 | 1.000 | 737 | tags=7%, list=6%, signal=8% |
| 2793 | GOBP\_CHROMOSOME\_ORGANIZATION\_INVOLVED\_IN\_MEIOTIC\_CELL\_CYCLE |  | 47 | -0.15 | -0.54 | 0.998 | 1.000 | 1.000 | 4169 | tags=34%, list=32%, signal=50% |
| 2794 | GOBP\_HIGH\_DENSITY\_LIPOPROTEIN\_PARTICLE\_REMODELING |  | 16 | -0.19 | -0.54 | 0.979 | 0.999 | 1.000 | 1519 | tags=19%, list=12%, signal=21% |
| 2795 | GOBP\_REGULATION\_OF\_CARDIOCYTE\_DIFFERENTIATION |  | 21 | -0.17 | -0.54 | 0.987 | 0.999 | 1.000 | 3103 | tags=29%, list=24%, signal=38% |
| 2796 | GOBP\_SYNAPTIC\_TRANSMISSION\_CHOLINERGIC |  | 28 | -0.16 | -0.54 | 0.989 | 0.999 | 1.000 | 4868 | tags=46%, list=38%, signal=74% |
| 2797 | GOBP\_DETECTION\_OF\_MECHANICAL\_STIMULUS |  | 40 | -0.15 | -0.54 | 0.997 | 0.999 | 1.000 | 3001 | tags=20%, list=23%, signal=26% |
| 2798 | GOBP\_FATTY\_ACID\_DERIVATIVE\_BIOSYNTHETIC\_PROCESS |  | 35 | -0.15 | -0.53 | 1.000 | 0.999 | 1.000 | 3766 | tags=31%, list=29%, signal=44% |
| 2799 | GOBP\_POSITIVE\_REGULATION\_OF\_BONE\_RESORPTION |  | 15 | -0.19 | -0.53 | 0.973 | 1.000 | 1.000 | 355 | tags=7%, list=3%, signal=7% |
| 2800 | GOBP\_PROTEIN\_LOCALIZATION\_TO\_CHROMATIN |  | 24 | -0.16 | -0.52 | 0.993 | 1.000 | 1.000 | 4434 | tags=38%, list=34%, signal=57% |
| 2801 | GOBP\_GLUCOSE\_CATABOLIC\_PROCESS |  | 18 | -0.18 | -0.52 | 0.988 | 1.000 | 1.000 | 10642 | tags=100%, list=82%, signal=570% |
| 2802 | GOBP\_REGULATION\_OF\_CHROMATIN\_ASSEMBLY\_OR\_DISASSEMBLY |  | 25 | -0.16 | -0.52 | 0.988 | 0.999 | 1.000 | 1833 | tags=12%, list=14%, signal=14% |
| 2803 | GOBP\_POSITIVE\_REGULATION\_OF\_PEPTIDYL\_THREONINE\_PHOSPHORYLATION |  | 22 | -0.16 | -0.51 | 0.987 | 1.000 | 1.000 | 3358 | tags=27%, list=26%, signal=37% |
| 2804 | GOBP\_MODULATION\_OF\_PROCESS\_OF\_ANOTHER\_ORGANISM |  | 19 | -0.17 | -0.51 | 0.983 | 0.999 | 1.000 | 1686 | tags=11%, list=13%, signal=12% |
| 2805 | GOBP\_REGULATION\_OF\_THE\_FORCE\_OF\_HEART\_CONTRACTION |  | 25 | -0.16 | -0.51 | 0.991 | 0.999 | 1.000 | 1934 | tags=16%, list=15%, signal=19% |
| 2806 | GOBP\_NEGATIVE\_REGULATION\_OF\_SMOOTHENED\_SIGNALING\_PATHWAY |  | 24 | -0.15 | -0.50 | 0.997 | 1.000 | 1.000 | 4065 | tags=33%, list=32%, signal=49% |
| 2807 | GOBP\_REGULATION\_OF\_MICROTUBULE\_BASED\_MOVEMENT |  | 33 | -0.14 | -0.49 | 0.998 | 1.000 | 1.000 | 4742 | tags=42%, list=37%, signal=67% |
| 2808 | GOBP\_NEGATIVE\_REGULATION\_OF\_T\_CELL\_RECEPTOR\_SIGNALING\_PATHWAY |  | 22 | -0.15 | -0.48 | 0.993 | 1.000 | 1.000 | 4136 | tags=32%, list=32%, signal=47% |
| 2809 | GOBP\_POSITIVE\_REGULATION\_OF\_ANTIGEN\_RECEPTOR\_MEDIATED\_SIGNALING\_PATHWAY |  | 19 | -0.16 | -0.48 | 0.998 | 1.000 | 1.000 | 5158 | tags=47%, list=40%, signal=79% |
| 2810 | GOBP\_RESPONSE\_TO\_HEPATOCYTE\_GROWTH\_FACTOR |  | 18 | -0.16 | -0.48 | 0.996 | 0.999 | 1.000 | 4368 | tags=39%, list=34%, signal=59% |
| 2811 | GOBP\_RIG\_I\_SIGNALING\_PATHWAY |  | 25 | -0.14 | -0.47 | 1.000 | 1.000 | 1.000 | 3474 | tags=20%, list=27%, signal=27% |
| 2812 | GOBP\_CIRCADIAN\_SLEEP\_WAKE\_CYCLE |  | 19 | -0.15 | -0.47 | 0.987 | 0.999 | 1.000 | 10929 | tags=100%, list=85%, signal=653% |
| 2813 | GOBP\_POSITIVE\_REGULATION\_OF\_MACROPHAGE\_DERIVED\_FOAM\_CELL\_DIFFERENTIATION |  | 18 | -0.16 | -0.46 | 0.998 | 0.999 | 1.000 | 3289 | tags=22%, list=25%, signal=30% |
| 2814 | GOBP\_CIRCADIAN\_SLEEP\_WAKE\_CYCLE\_SLEEP |  | 15 | -0.15 | -0.43 | 0.996 | 0.999 | 1.000 | 10929 | tags=100%, list=85%, signal=653% |
Table: Gene sets enriched in phenotype **normal (10 samples) **[plain text format]****
